# Programming inactive RNA-binding small molecules into bioactive degraders

**DOI:** 10.1038/s41586-023-06091-8

**Published:** 2023-05-24

**Authors:** Yuquan Tong, Yeongju Lee, Xiaohui Liu, Jessica L. Childs-Disney, Blessy M. Suresh, Raphael I. Benhamou, Chunying Yang, Weimin Li, Matthew G. Costales, Hafeez S. Haniff, Sonja Sievers, Daniel Abegg, Tristan Wegner, Tiffany O. Paulisch, Elizabeth Lekah, Maison Grefe, Gogce Crynen, Montina Van Meter, Tenghui Wang, Quentin M. R. Gibaut, John L. Cleveland, Alexander Adibekian, Frank Glorius, Herbert Waldmann, Matthew D. Disney

**Affiliations:** 1 Department of Chemistry, The Scripps Research Institute & The Herbert Wertheim UF Scripps Institute for Biomedical Innovation & Technology, Jupiter, FL USA; 2 Department of Tumor Biology, Moffitt Cancer Center & Research Institute, Tampa, FL USA; 3 Max Planck Institute of Molecular Physiology, Dortmund, Germany; 4 Compound Management and Screening Center, Dortmund, Germany; 5 Organisch-Chemisches Institut, University of Münster, Münster, Germany; 6 Bioinformatics and Statistics Core, The Scripps Research Institute and The Herbert Wertheim UF Scripps Institute for Biomedical Innovation & Technology, Jupiter, FL USA; 7 Histology Core, The Scripps Research Institute and The Herbert Wertheim UF Scripps Institute for Biomedical Innovation & Technology, Jupiter, FL USA; 8 Department of Chemistry and Chemical Biology, TU Dortmund University, Dortmund, Germany

**Keywords:** RNA, Small molecules, RNA

## Abstract

Target occupancy is often insufficient to elicit biological activity, particularly for RNA, compounded by the longstanding challenges surrounding the molecular recognition of RNA structures by small molecules. Here we studied molecular recognition patterns between a natural-product-inspired small-molecule collection and three-dimensionally folded RNA structures. Mapping these interaction landscapes across the human transcriptome defined structure–activity relationships. Although RNA-binding compounds that bind to functional sites were expected to elicit a biological response, most identified interactions were predicted to be biologically inert as they bind elsewhere. We reasoned that, for such cases, an alternative strategy to modulate RNA biology is to cleave the target through a ribonuclease-targeting chimera, where an RNA-binding molecule is appended to a heterocycle that binds to and locally activates RNase L^1^. Overlay of the substrate specificity for RNase L with the binding landscape of small molecules revealed many favourable candidate binders that might be bioactive when converted into degraders. We provide a proof of concept, designing selective degraders for the precursor to the disease-associated microRNA-155 (pre-miR-155), *JUN* mRNA and *MYC* mRNA. Thus, small-molecule RNA-targeted degradation can be leveraged to convert strong, yet inactive, binding interactions into potent and specific modulators of RNA function.

## Abstract (web summary)

Small-molecule RNA-targeted degradation can be leveraged to convert strong, yet inactive, binding interactions into potent and specific modulators of RNA function.

## Main

The importance of RNA in health and disease biology is well documented^2^, affording opportunities within chemical biology to study function or intervene against dysfunction, respectively. Sequence-based targeting of RNA is often accomplished with complementary oligonucleotides that bind to and then recruit a ribonuclease to cleave the target^3^. This modality is best suited for targeting unstructured regions in an RNA, as molecular recognition occurs through base pairing^4^. However, RNA can be highly structured, and its biological function is often dictated by its structure^5,6^. Notably, such structured regions are amenable to targeting by the binding of small molecules that interact with pockets presented by an RNA fold^7^. However, the occupancy of RNA structures by small molecules alone is often not sufficient to elicit a biological effect^8^.

Here we developed a strategy that converts biologically inactive RNA-binding small molecules into potent and specific effectors of function, achieved by affixing an RNA molecular recognition element to a second compound that binds to and activates a ribonuclease to cleave the target. Our focus is threefold: (1) define binding interactions between small molecules and RNA folds; (2) convert, in a programmable manner, highly selective binding interactions that are biologically inert into inducers of targeted degradation, affording potent and selective functional inhibitors; and (3) establish a paradigm by which small molecules can eliminate RNAs.

## Identification of RNA-binding chemotypes

**Fig. 1:**
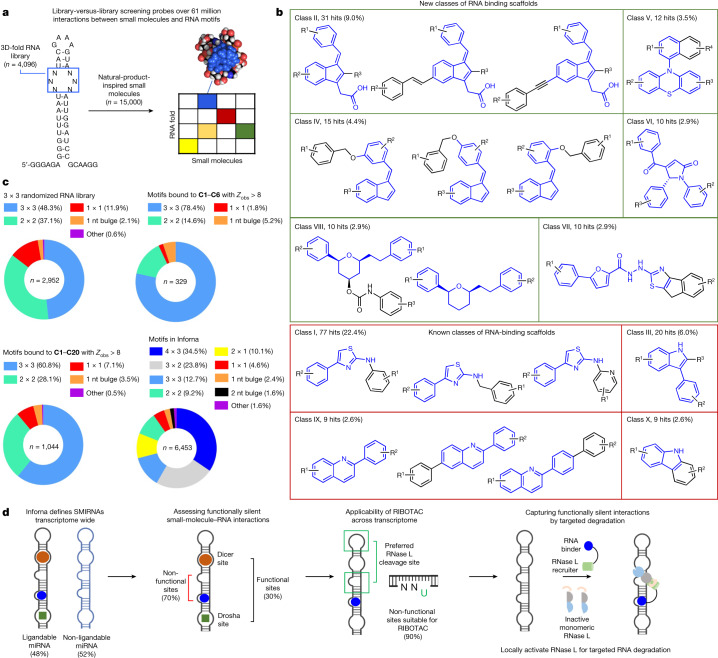
Library-versus-library screening defines new RNA-binding small molecules and druggable targets. **a**, 2DCS analysis of more than 61 million theoretical interactions, identifying new interactions between small molecules and RNA motifs. **b**, The newly identified small-molecule RNA binders (*n* = 344) included 156 different scaffolds that fall into 79 major classes based on scaffold similarities. Among the top 10 most abundant classes (collectively covering 59.6% of all hits), six are new classes (green). **c**, Motif distribution from a 3 × 3 randomized RNA library used for 2DCS screening. As expected, 3 × 3 and 2 × 2 internal loops comprise the majority (85.4% total) of the library. Motifs that bound to **C1**–**C6** showed a significant enrichment for 3 × 3 internal loops (*P* < 0.001) and one-nucleotide bulges (*P* < 0.001). A total of 1,044 motifs bound to **C1**–**C20** with *Z*_obs_ > 8. Preference for 3 × 3 internal loops and one-nucleotide bulges was collectively observed for these compounds. Of these 1,044 motifs, only 23 (2.2%) are present in highly expressed human transcripts (*n* = 2,712 total motifs), and 375 are new motifs with no previously known small-molecule binder. Inforna contains over 100,000 RNA–small molecule interactions and 6,453 unique RNA motifs of various types. **d**, Although around 6% of all miRNAs can be bound by **C1**–**C6**, only about 30% of targetable sites within them are functional (Drosha or Dicer processing site) and are therefore predicted to induce a biological effect. The other approximately 70% are unproductive interactions that are predicted to be biologically silent. We identified that 48% of miRNAs that have ligandable non-functional sites are potential substrates for RNase L, which could be targeted by RIBOTACs. Thus, biologically inert binders can be converted into bioactive RIBOTACs that provoke targeted degradation. Statistical significance referred to in **c** was calculated using two-tailed Student’s *t*-tests.

**Extended Data Fig. 1:**
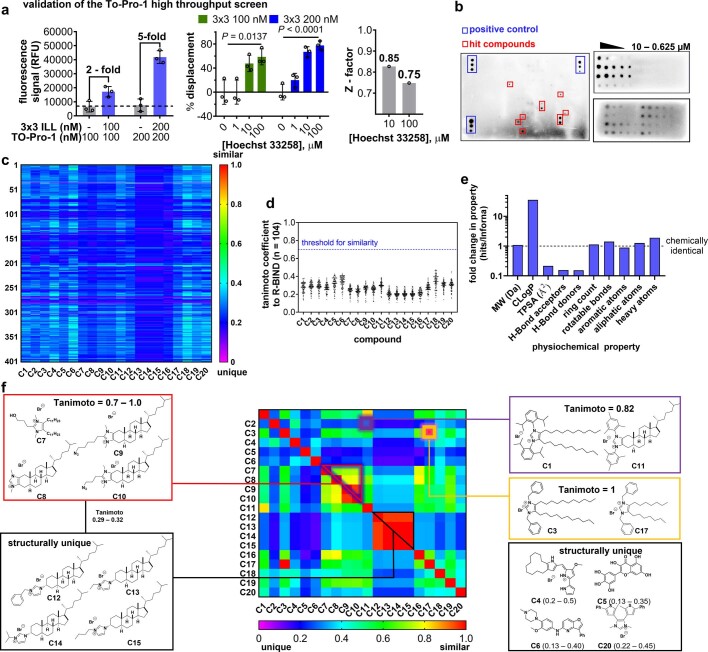
To-Pro-1 dye displacement assay validation and newly identified small molecules that bind RNA are chemically dissimilar from previously known RNA binders. **a**, To-Pro assay optimization and validation. Optimization of the signal to noise ratio of To-Pro-1 dye displacement from an RNA 3×3 internal loop library (3×3 ILL) by altering the concentration of the dye and the RNA (left; *n* = 3 independent replicates). Validation using Hoechst 33258 as a positive control to displace To-Pro-1 (middle; *n* = 3 independent replicates). Z-factor analysis of the screening conditions using 10 and 100 µM Hoechst 33258 (right). For each concentration, *n* = 690 in a single independent experiment. Data are reported as the mean ± SD. **b**, AbsorbArray screening with a 5’-^32^P-labelled 3×3 ILL. Compounds were delivered to the array surface in dose response (200 nL of 10 – 0.625 mM, log_2_ dilutions). **c**, Heat map of the Tanimoto analysis of 404 molecules found within Inforna show that **C1–C20** are chemically dissimilar to known RNA-binding matter. **d**, Average chemical similarity of **C1**–**C20** to compounds within the R-BIND database (*n* = 104 unique compounds). **e**, Comparison of the physicochemical properties of **C1–C20** and molecules housed within Inforna. The compounds identified herein are chemically distinct, having ~35-fold higher CLogP, an ~20% reduction in polar surface area (TPSA), and an ~15% reduction in the number of both H-bond donors and acceptors^15,57,58^. **f**, Unique chemical patterns indicate chemically similar compounds within the azolium scaffold. Compounds **C7–C10** have a Tanimoto scores ranging from 0.7 to >0.995 and cluster together. Compounds **C12**–**C15** are nearly chemically identical as they share the cholesterol-derived azolium core, differing only by the other N-substituent. However, when compared to the remaining compounds (**C1–C11** and **C16–C20**), their Tanimoto score ranges from 0.29–0.32, indicating they are unique among the hits obtained. **C1** and **C11** exhibit high similarity (Tanimoto coefficient = 0.82), although they appear very different structurally. This could be due to their similar spatial orientations, as both have alkyl substituted benzenes on their azolium cores. Compounds **C4**, **C5**, **C6**, and **C20** are structurally unique compared to all other hits. Source data

A 15,000-member, natural-product-like small molecule compound collection with diverse properties^9^ was investigated for binding to a library of RNA 3D folds presented in a 3 × 3 internal loop library (ILL; 61,440,000 potential binding interactions probed) (Fig. 1a). The 4,096 unique RNA folds in 3 × 3 ILL include 1 × 1, 2 × 2 and 3 × 3 internal loops as well as bulge loops and fully paired RNAs. Binding was assessed using a fluorescent-dye-displacement assay^10^, in which compounds from the library that bind to the RNA displace the dye and decrease its emission. A primary triage of the 15,000 small molecules (10 μM) yielded 1,584 hit compounds (Extended Data Fig. 1a). Secondary validation of the top 480 compounds yielded 344 binding small molecules. These diverse compounds include six RNA-binding scaffolds, such as 1-benzylidene-1-indene and phenothiazine (Fig. 1b).

The binding of these 344 small molecules was studied using an orthogonal, microarray-based method named AbsorbArray^11^, yielding 26 compounds (Extended Data Fig. 1b). Notably, AbsorbArray enables parallel affinity purification of the precise RNA members of the 3 × 3 ILL preferred by each small molecule when completed in the presence of excess unlabelled competitor oligonucleotides that mimic regions common to all library members, a selection method named two-dimensional combinatorial screening (2DCS)^12^. Of the 26 hit compounds from the primary and validation screens, six compounds (**C1**–**C6**) bound to the RNA library under these highly stringent conditions (Extended Data Fig. 1b and Supplementary Table 1). To determine the RNA 3D folds preferred by **C1**–**C6**, the frequency of each motif in the selected library was measured using RNA sequencing (RNA-seq) and compared to its frequency in the starting RNA pool. The statistical significance of the resulting enrichment was quantified by a pooled population comparison as *Z*_obs_^13^. Collectively, these studies defined the affinity landscapes for the six small molecules, that is, structure–activity relationships (SARs) between small molecules and their preferred RNA 3D folds (Supplementary Table 1).

Three lines of evidence support that the six molecules identified here are new RNA-binding molecules. First, chemical similarity was assessed by calculating Tanimoto coefficients^14^, where a threshold of <0.7 typically indicates dissimilar molecules^15^. The mean Tanimoto coefficient of the six small molecules identified here to all known RNA binders that are housed in two databases (Inforna, 404 small molecules^16^; and R-BIND, 104 small molecules^17^) was 0.25 ± 0.07 (Extended Data Fig. 1c,d). Second, a comparison of the physicochemical properties of **C1**–**C6** to known RNA-binding small molecules showed key differences in the total polar surface area (TPSA) as well as in the number of hydrogen bond donors and acceptors (Extended Data Fig. 1e). Finally, scaffold analysis revealed six new RNA-binding scaffolds, including azolium salts, bipyrrole pyrrolium salts and chromones (Extended Data Fig. 1d,f).

**Extended Data Fig. 2:**
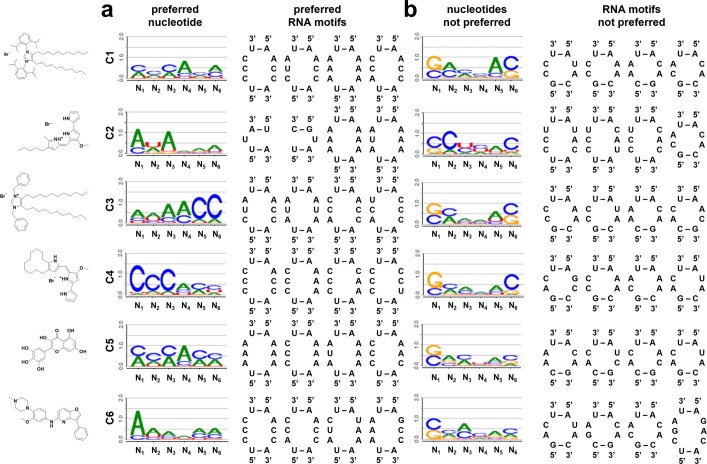
LOGO analyses for C1 – C6, showing both preferred and discriminated sequences and structures. **a**, LOGO analysis of the selected RNAs in the top 0.5% of Z_obs_ and the secondary structures of the selected RNAs with the four highest Z_obs_ values. **b**, LOGO analysis of RNAs discriminated against by each small molecule, *i.e*., in the bottom 0.5% of Z_obs_, and the secondary structures of the RNAs discriminated against with the four lowest Z_obs_ values. Interestingly, many have GC closing base pairs and are 2×2 nucleotide internal loops. Similarities and differences were found amongst the small molecules. For example, within the azolium scaffold, **C1** prefers C at positions one and three and A at positions four and six (that is, internal loops with two Cs opposite two As), while **C3**, which differs by the substituents on the azolium core, prefers A at positions one, three and four, but C at positions six. Interestingly, **C1**–**C6**, despite their chemical differences in scaffolds, all discriminate against a 5’ GC closing base pair (G and C at positions one and six, respectively).

To define SAR, the RNA 3D folds significantly enriched for each small molecule with a *Z*_obs_ > 8—a statistical threshold considered to be bound by the small molecule^13^—were analysed (*n* = 329 unique motifs; Fig. 1c). Notably, these compounds collectively showed a preference for 3 × 3 internal loops (78.4% bound by small molecules versus 48.3% in the initial library; *P* < 0.001) and one-nucleotide bulges (5.2% compared to 2.1%; *P* < 0.001; Fig. 1c). Among the 329 unique motifs, 258 had no known small-molecule binder. LOGO analysis of the RNAs in the top 0.5% of statistically significant enriched loops (*Z*_obs_ > 8) revealed that each molecule binds to a unique RNA sequence pattern (Extended Data Fig. 2).

To generate a transcriptome-wide map of the RNA folds bound by **C1**–**C6**, we compared them to a database of folded human RNA structures^18^. The database includes structural information for all human primary (pri-) and precursor (pre-) microRNAs (miRNAs; 7,436 non-canonically paired motifs), and for highly expressed transcripts with a known structure, including 5S, 16S and 23S rRNAs, 7SL (signal-recognition particle), RNase P RNA, U4/U6 small nuclear RNA and 465 non-redundant tRNAs. Informatic mining of these structures against the RNA fold–small molecule interactions identified here showed that 13 motifs bound by **C1**–**C6** were present 114 times in 111 human miRNAs; that is, approximately 6% of human miRNAs have structures that are targetable by **C1**–**C6**. Of these targetable structures, only 33 (28.9%) are present in a functional Drosha or Dicer processing site, indicating that most interactions would afford unproductive target engagement, that is, inactivity in a biological system (Fig. 1d). Notably, only 4 out of the 329 unique motifs identified from this selection (1.2%) bound by **C1**–**C6** were present in highly expressed human transcripts (*n* = 2,712 motifs).

## Design of a pre-miR-155 RIBOTAC degrader

Given the preponderance of biologically silent interactions, the challenge is therefore how to convert these into bioactive small molecules. One strategy is to imbue the binders with the ability to cleave its specific target, by generating a ribonuclease-targeting chimera (RIBOTAC)^1^. RIBOTACs are composed of an RNA-binding compound conjugated to a second small molecule that recruits and locally activates RNase L^1^, which preferentially cleaves RNAs with a UNN pattern (with unpaired Us)^19–22^ (Fig. 1d). Notably, of the predicted functionally silent interactions, 55 targetable miRNAs (48.2%) have at least one nearby RNase L site (within 10 bp) that could therefore be sensitive to a RIBOTAC (Fig. 1d).

**Fig. 2:**
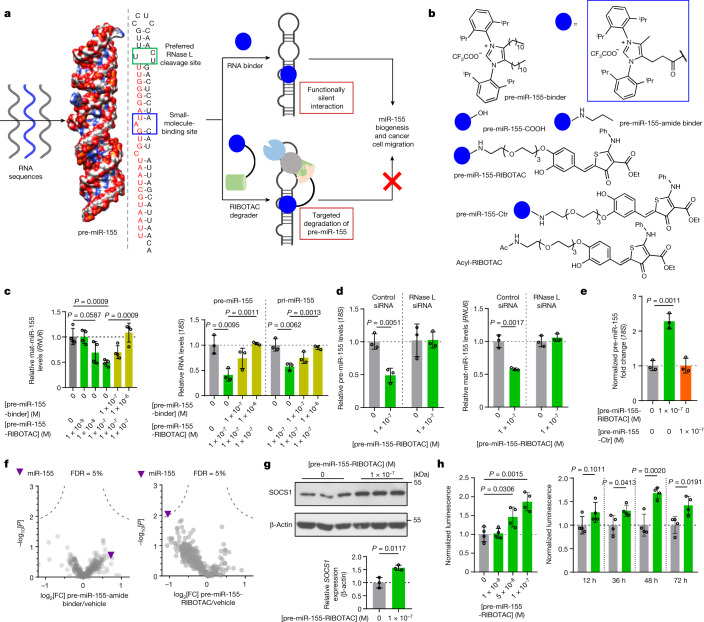
Pre-miR-155-RIBOTAC selectively degrades pre-miR-155 in an RNase-L-dependent manner in breast cancer cells. **a**, Schematic of converting an inert binder engaging pre-miR-155 into an active RIBOTAC degrader. **b**, Structures of the compounds used to target pre-miR-155. **c**, The effects of pre-miR-155-RIBOTAC on mature (mat) (*n* = 4 biological replicates), pre- (*n* = 3 biological replicates) and pri- (*n* = 3 biological replicates) miR-155 levels, competed by increasing concentrations of pre-miR-155-binder in MDA-MB-231 cells. **d**, The effect of siRNA knockdown of RNase L on pre-miR-155-RIBOTAC-mediated cleavage of pre-miR-155 in MDA-MB-231 cells, as determined using RT–qPCR. *n* = 3 biological replicates. **e**, Immunoprecipitation of pre-miR-155 using an anti-RNase L antibody in the presence of pre-miR-155-RIBOTAC in MDA-MB-231 cells (*n* = 3 biological replicates). **f**, The effect of pre-miR-155-amide-binder (left; 100 nM; *n* = 4 biological replicates) and pre-miR-155-RIBOTAC (right; 100 nM; *n* = 3 biological replicates) on the levels of the 373 miRNAs expressed in MDA-MB-231 cells. FC, fold change. **g**, Western blot analysis of SOCS1, a direct target of miR-155, after treatment of MDA-MB-231 cells with pre-miR-155-RIBOTAC (*n* = 3 biological replicates). **h**, The effect of pre-miR-155-RIBOTAC on the activity of a *SOCS1* 3′ UTR-luciferase reporter transfected into HEK293T cells, establishing both dose (left) and time dependence (right; *n* = 4 biological replicates). Data are mean ± s.d. (**c**–**e**, **g** and **h**). Statistical significance was determined using two-tailed Student’s *t*-tests (**c**–**e**, **g** and **h**) or a Wald’s test (**f**). Source data

**Extended Data Fig. 3:**
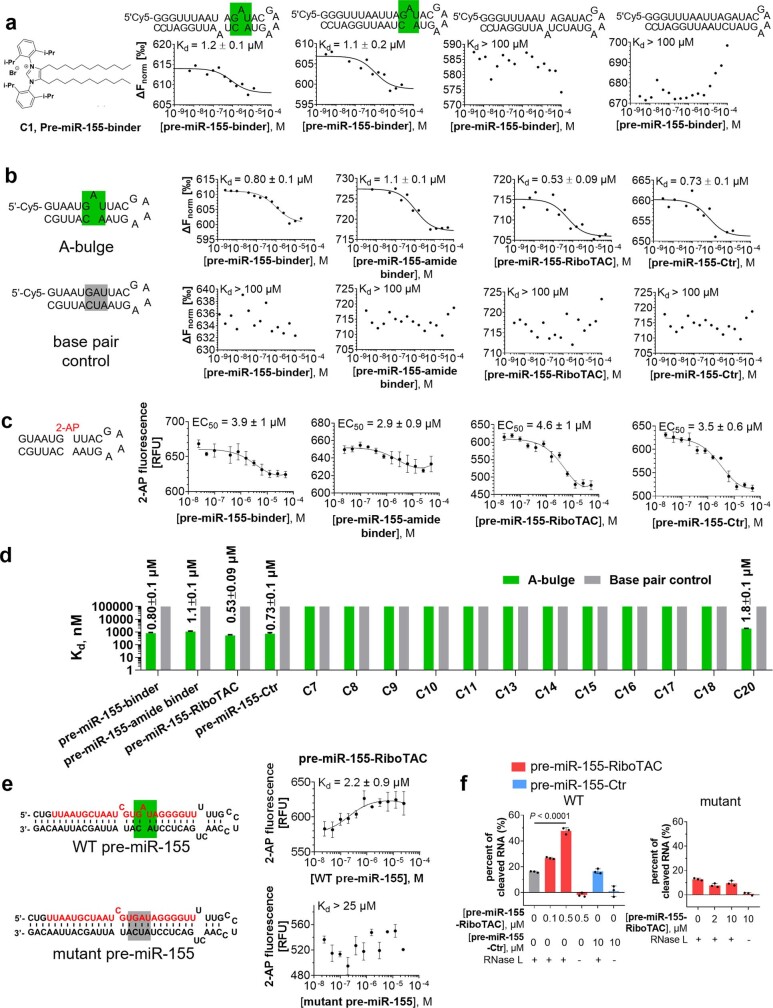
In vitro characterization of pre-miR-155-binder and its derivatives for pre-miR-155’s A bulge. **a**, Representative binding curves of **pre-miR-155-binder** and two A bulges identified from the 2DCS selection and a fully based paired control, as determined by microscale thermophoresis (MST). The bulge found in pre-miR-155 is highlighted in green. **b**, Representative binding curves for **pre-miR-155-binder** and its derivatives for a minimized A bulge construct or fully paired mutant, as determined by MST. **c**, Representative binding curves for **pre-miR-155-binder** and its derivatives and pre-miR-155’s A bulge labelled with 2-aminopurine (2AP) or a fully base paired control (*n* = 2 independent experiments). **d**, Summary of K_d_s of **pre-miR-155-binder** analogues and other imidazolium salts identified by 2DCS. The affinity of **C19** for the A bulge was undetermined due to its aggregation under assay conditions. **e**, Wild type pre-miR-155 but not the AU based paired mutant RNA can compete the binding of **pre-miR-155-RiboTAC** (5 µM) to 2AP labelled RNA (1 µM) with a K_d_ of 2.2 ± 0.9 μM (*n* = 2 independent experiments). **f**, **pre-miR-155-RiboTAC** induces in vitro RNA cleavage with wild-type (WT) pre-miR-155 construct (left), while no cleavage with mutated base pair control (right) (*n* = 3 independent experiments). All data are reported as the mean ± S.D. of independent replicates. All p-values were calculated using a two-tailed Student’s t-test. Source data

One of the predicted biologically silent interactions identified is between **C1** (described previously^23,24^) and the 5′G**A**U/3′C_A bulge of pre-miR-155, where bold indicates an unpaired or non-canonically paired nucleotide (Fig. 2a). miR-155 has been implicated in inflammation^25^, breast cancer^26^ and other diseases. The **C1**–5′G**A**U/3′C_A interaction was identified from a member of the 3 × 3 ILL that is predicted to form two bulges, rather than an internal loop (Extended Data Fig. 3a). Furthermore, the 5′G**A**U/3′C_A binding site is proximal to a 5′U**U**U/3′G**UC**A motif that is predicted to be a substrate for RNase L^20,21^ (Fig. 2a). Binding analysis by microscale thermophoresis (MST) on the 2DCS-selected RNA shows that **C1** binds only to the 5′GAU/3′C_A motif (*K*_d_ = 1.1 ± 0.2 µM), similar to an RNA with both bulges (*K*_d_ = 1.2 ± 0.1 µM) and a minimized RNA construct (*K*_d_ = 0.8 ± 0.1 µM) (Extended Data Fig. 3b). By contrast, saturable binding was not observed to the RNA that displays only the other bulge or where a point mutation in the **C1**-targeting converted the A bulge into an AU pair (Extended Data Fig. 3a,b). These data were verified in an orthogonal binding assay in which the A bulge was replaced with the fluorescent adenine mimic 2-aminopurine (2AP) (*K*_d,app_ = 3.9 ± 1 µM) (Extended Data Fig. 3c).

**Extended Data Fig. 4:**
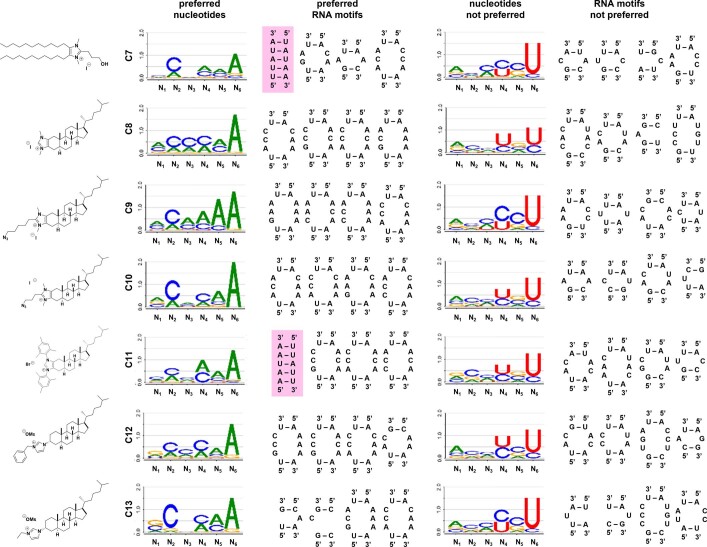
LOGO analyses for preferred and discriminated sequences for C7–C13. Interestingly, ten of the 14 azoliums share a tetradecahydro-1*H*-cyclopenta[a]phenanthrene substituent, six of which are fused to the azolium (Supplementary Table 1). In general, all 7 compounds prefer loops with Cs and As, with a strong preference for A in the sixth position, with degree of preference at each position unique to each compound. Likewise, all 7 compounds discriminate against loops with Us, particularly in the sixth position. **Left**, LOGO analysis of the selected RNAs in the top 0.5% of Z_obs_ and the secondary structures of the selected RNAs with the four highest Z_obs_ values for compounds **C7**–**C13**. Generally, these compounds prefer motifs rich in C and A residues, with position 6 almost exclusively A. Similar to **C1**–**C6**, 3×3 internal loops predominate for **C7**–**C20** (75%) (p < 0.001, as compared to its distribution in 3×3 ILL), with single nucleotide bulges and 1×1 and 2×2 internal loops comprising 14%. Interestingly, **C7** and **C11** prefer to bind AU base pairs (pink box). **Right**, LOGO analysis of RNAs discriminated against by each small molecule, *i.e*., in the bottom 0.5% of Z_obs_, and the secondary structures of the RNAs discriminated against with the four lowest Z_obs_ values for compounds **C7**–**C13**. Motifs that are discriminated against for binding are significantly enriched for single nucleotide bulges (18%) (p < 0.001, as compared to its distribution in 3×3 ILL) and 1×1 nucleotide internal loops (29%) (p < 0.001, as compared to its distribution in 3×3 ILL), with position 6 predominately U. Notably, **C1** lacks the strong preference for A at position 6.

**Extended Data Fig. 5:**
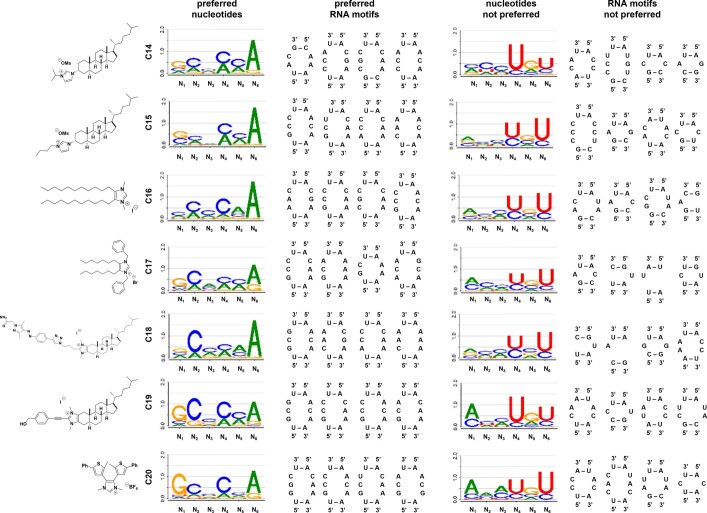
LOGO analyses for preferred and discriminated sequences for C14 – C20. **Left**, LOGO analysis of the selected RNAs in the top 0.5% of Z_obs_ and the secondary structures of the selected RNAs with the four highest Z_obs_ values for compounds **C14**–**C20**. Generally, these compounds prefer motifs rich in C and A residues, with position 6 almost exclusively A. Similar to **C1**–**C6**, 3×3 internal loops predominate for **C7**–**C20** (75%) (p < 0.001, as compared to its distribution in 3×3 ILL), with single nucleotide bulges and 1×1 and 2×2 internal loops comprising 14%. **Right**, LOGO analysis of RNAs discriminated against by each small molecule, *i.e*., in the bottom 0.5% of Z_obs_, and the secondary structures of the RNAs discriminated against with the four lowest Z_obs_ values for compounds **C1–C20**. Preferred sequences for **C14–C20** are rich in C and A, with positions 1 and 6 preferred as G and A, respectively. Two of the azoliums, **C16** and **C17**, are the most similar to **C1**; in **C16**, the substituted phenyls in **C1** are replaced with methyl groups, while in **C17**, the alkyl chains have been shortened from C_15_ to C_7_ and the 1,3-diisopropyl-2-methyphenyl groups have been replaced with benzyl groups. Like the other azolium salts, LOGO analyses of these three compounds revealed a preference for C and A nucleotides, with positional differences for all three molecules.

To our knowledge, azoliums have not been known to bind RNA. Thus, the RNA-binding ability of over 200 azolium derivatives was investigated. Of these compounds, only 14 (**C7**–**C20**) bound to RNA in the presence of competitor oligonucleotides (Supplementary Table 1). Notably, 10 of these 14 azolium salts share a cholesterol-derived sterol backbone, six of which are fused to the azolium ring. In total, 715 motifs are bound by **C7**–**C20** with *Z*_obs_ > 8, among which 117 are new with no previously known small-molecule binders (Extended Data Figs. 4 and 5 and Supplementary Table 1). Combining these with motifs bound by **C1**–**C6**, this study contributes 375 new RNA motifs to the current database of RNA–small-molecule interactions. An analysis of the RNAs selected by the azolium salts revealed that the top three small molecules predicted to bind to the 5′G**A**U/3′C_A motif in pre-miR-155 were **C19**, **C1** and **C20**, in rank order. Affinity measurements showed that only **C1** and **C20** bind to the A bulge of miR-155, while **C19** binding was undetermined due to aggregation under assay conditions (Extended Data Fig. 3d). As observed for **C1**, **C20** showed no saturable binding to an RNA with the A bulge changed to an AU pair (Extended Data Fig. 3d).

**Extended Data Fig. 6:**
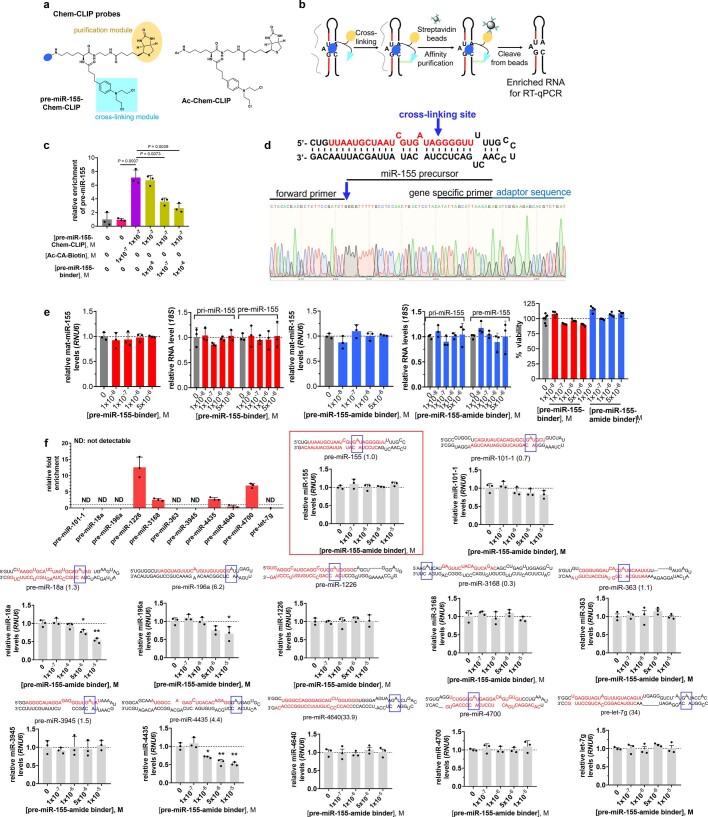
pre-miR-155-binder and derivatives directly engage pre-miR-155 and other A bulge containing miRNA precursors in MDA-MB-231 cells and in vitro. **a**, The Chem-CLIP probe for target validation studies (**pre-miR-155-Chem-CLIP**) was synthesized by conjugating **pre-miR-155-COOH** to chlorambucil (CA; cross-linking module; blue triangle) and biotin (pull-down module; yellow circle). A control Chem-CLIP probe, Ac-CA-biotin, comprises the cross-linking and pull-down module but lacks the RNA-binding module and accounts for non-selective reaction of the CA module. **b**, Chem-CLIP is a proximity-based reaction driven by the RNA-binding module. Following treatment, total RNA is isolated and purified with streptavidin beads and analysed by RT-qPCR. **c**, Results of target validation studies in MDA-MB-231 breast cancer cells. Cells were treated with either Ac-CA-Biotin (100 nM) or **pre-miR-155-Chem-CLIP** (100 nM). Enrichment of pre-miR-155 in the pulled-down fraction, as compared to the starting cell lysate, was quantified by RT-qPCR (left). Co-treatment with **pre-miR-155-Chem-CLIP** and **pre-miR-155-binder** resulted in dose dependent depletion of pre-miR-155 in the pull-down fraction, indicating that the Chem-CLIP probe and the simple binding compound **pre-miR-155-binder** occupy the same site (*n* = 3 biological replicates). **d**, **pre-miR-155-Chem-CLIP** cross-links to pre-miR-155 at a nucleotide 5 bp from the A bulge binding site (indicated with a blue arrow in the secondary structure), identified by in vitro Chem-CLIP-Map-Seq. **e**, Effect of **pre-miR-155-binder** (red; left) and **pre-miR-155-amide-binder** (blue; middle) (*n* = 3 biological replicates) on pri-, pre-, and mature miR-155 levels as well as the two compounds’ effects on cellular viability (right) (*n* = 4 biological replicates). **f**, Chem-CLIP analysis (100 nM **pre-miR-155-Chem-CLIP**) of miRNA precursors that have the same bulge as pre-miR-155 bound by **pre-miR-155-binder** (*n* = 3 biological replicates) where ND indicates that the miRNA was not detectable in the pulled down fraction. Effect of **pre-miR-155-amide-binder** (0.1 to 10 μM) on the levels of miRNAs that contain the A bulge in miR-155, as determined by RT-qPCR (*n* = 3 biological replicates). **Pre-miR-155-RiboTAC** (100 nM) has no effect on the levels of mature RNAs that share **C1**’s A bulge in miR-155 (Extended Data Fig. 6h). All data are reported as the mean ± S.D. All p-values were calculated using a two-tailed Student’s t-test. Source data

We first tested the hypothesis that **C1** (hereafter pre-miR-155-binder), engages the miR-155 precursor in cells but is biologically inert; that is, its binding to the miR-155 precursor did not inhibit biogenesis nor reduce mature miR-155 levels. To study target engagement, we used chemical cross-linking and isolation by pull-down (Chem-CLIP)^27^. In Chem-CLIP, an RNA-binding molecule is modified with a cross-linking moiety (chlorambucil (CA)) and a biotin purification module to enable pull-down with streptavidin beads (Extended Data Fig. 6a,b). To install the required modules, we hypothesized that the *n*-undecyl chains do not significantly contribute to binding. To test this, the *n*-undecyl chains were replaced with a propionic acid linker to afford compound pre-miR-155-COOH (Fig. 2b). We then amidated pre-miR-155-COOH with propylamine to mimic the reaction to generate the Chem-CLIP probe, yielding pre-miR-155-binder-amide (Fig. 2b), which binds with similar affinity to the A bulge as the pre-miR-155-binder does in both the MST- and 2AP-binding assays (Extended Data Fig. 3b,c), supporting our molecular-recognition hypothesis.

To confirm on-target binding in cells, a Chem-CLIP probe, pre-miR-155-Chem-CLIP (Extended Data Fig. 6a,b), was synthesized by installing chlorambucil and biotin through the carboxylic acid handle. A control Chem-CLIP probe was also synthesized that lacks the RNA-binding module (Ac-CA-Biotin, where Ac indicates acylated; Extended Data Fig. 6a). Pre-miR-155-Chem-CLIP (100 nM) enriched pre-miR-155 by around sevenfold in MDA-MB-231 cells, a triple-negative breast cancer cell line that aberrantly expresses miR-155^28^, whereas no enrichment was observed for Ac-CA-Biotin (Extended Data Fig. 6c). Importantly, this pull-down and enrichment of pre-miR-155 by pre-miR-155-Chem-CLIP was competed dose-dependently by co-treatment with **C1** (Extended Data Fig. 6c). Collectively, these studies demonstrate that **C1** directly binds to pre-miR-155 in cells. To affirm the precise binding site of **C1** within the miR-155 precursor, we used chemical cross-linking and isolation by pull-down to map small molecule RNA-binding sites (Chem-CLIP–Map-Seq)^29^. Indeed, in vitro, pre-miR-155-Chem-CLIP cross-linked adjacent to the A bulge-binding site (Extended Data Fig. 6d).

The effects of the pre-miR-155-binder on miR-155 biogenesis was assessed by measuring levels of pre- and mature miR-155 using quantitative PCR with reverse transcription (RT–qPCR). As expected, no effect was observed on the levels of either form of the miRNA from 0.01 to 5 µM, concentrations at which no toxicity was observed; similarly, pre-miR-155-amide-binder was also inactive (Extended Data Fig. 6e). Notably, the 5′G**A**U/3′C_A bulge found in the miR-155 precursor is also found in 11 other human miRNAs, including three miRNAs in which the A bulge is present in the Dicer processing site (miR-18a, miR-196a and miR-4435). Indeed, Chem-CLIP studies revealed that 4 out of these 11 miRNAs were directly engaged in MDA-MB-231 cells—miR-1226, miR-3168, miR-4435 and miR-4700 (Extended Data Fig. 6f). Note that six of the remaining miRNAs were not detectable in this assay, and pre-miR-4640 was not enriched. As expected, because the small molecule binding site is also a processing site, pre-miR-155-amide-binder reduced the levels of mature miR-18a, miR-196a and miR-4435 in a dose-dependent manner, but did not affect the levels of the other nine miRNAs (Extended Data Fig. 6f).

Given thatpre-miR-155-binder engages the pre-miR-155 target yet is biologically inert, we converted this RNA binder into a RIBOTAC through conjugation of pre-miR-155-COOH to an RNase-L-recruiting small molecule^1^, affording pre-miR-155-RIBOTAC (Fig. 2b). Approaches for converting functionally silent molecules into functional chimeric molecules are well studied in the protein-degradation field^30–32^, but not in the RNA-degradation field. Two control compounds were also synthesized—Ac-RIBOTAC, the RNase L-recruiting module lacking pre-miR-155-binder, and pre-miR-155-Ctr, which contains a less active RNase-L-recruiting module (Fig. 2b). Notably, the affinity of pre-miR-155-RIBOTAC for 5′G**A**U/3′C_A using the minimized binding site is similar to that of pre-miR-155 binder, whether measured by MST (*K*_d_ = 0.8 ± 0.1 µM versus 0.53 ± 0.09 µM, respectively) or using the 2AP-substituted bulge (*K*_d_ = 3.9 ± 1.2 µM versus 4.6 ± 1.2 µM, respectively) (Extended Data Fig. 3b,c). Furthermore, pre-miR-155-RIBOTAC binds to pre-miR-155 with an affinity of 2.2 ± 0.9 µM in the 2AP assay with no binding observed for a pre-miR-155 mutant in which the A bulge is mutated to an AU pair (Extended Data Fig. 3e).

**Extended Data Fig. 7:**
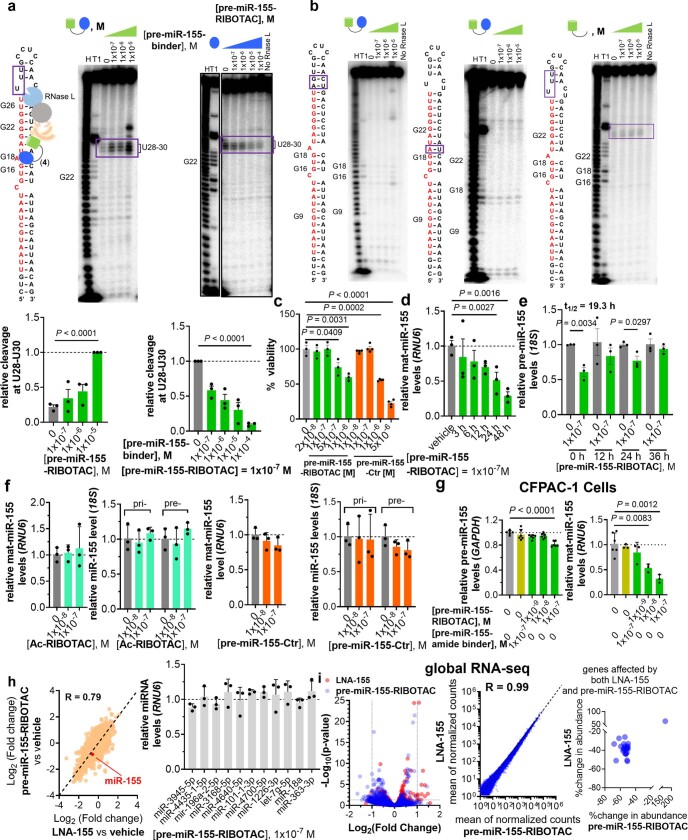
In vitro and cellular activities of pre-miR-155-RiboTAC and its derivatives. **a**, **left**: Representative gel autoradiogram of the cleavage of 5′-[^32^P]-pre-miR-155 by RNase L induced by **pre-miR-155-RiboTAC** and quantification thereof (IC_50_ = ~100 nM; *n* = 3 independent replicates); **right**: Representative gel autoradiogram of the **pre-miR-155-RiboTAC**-induced cleavage of pre-miR-155 as a function of **pre-miR-155-binder** concentration (IC_50_ = 1.9 ± 0.5 µM), demonstrating **pre-miR-155-binder** and **pre-miR-155-RiboTAC** bind the same site, and the corresponding quantification thereof (*n* = 3 independent replicates). **b**, Representative gel autoradiogram of (left to right): RNase L cleavage of cleavage site mutant of pre-miR-155 induced by **pre-miR-155-RiboTAC**; RNase L cleavage of the binding site mutant pre-miR-155 induced by **pre-miR-155-RiboTAC**; and RNase L cleavage of WT pre-miR-155 induced by **Ac-RiboTAC**, which lacks the RNA-binding module (*n* = 3 independent replicates). For panels **a** and **b**, “H” indicates a hydrolysis ladder while “T1” indicates treatment with RNase T1, which cleaves guanosine nucleotides. **c**, Effect of **pre-miR-155-RiboTAC** (*n* = 3 biological replicates) or **pre-miR-155-Ctr** (*n* = 4 biological replicates) on the viability of MDA-MB-231 cells following a 48 h treatment, as assessed by WST-1 assay. **d**, **pre-miR-155-RiboTAC** (single dose) reduces mature miR-155 levels in MDA-MB-231 cells in a time-dependent manner (*n* = 3 biological replicates). **e**, Duration of **pre-miR-155-RiboTAC**’s effect on pre-miR-155’s levels after a 48 h treatment (wash out experiment; *n* = 3 biological replicates). The compound-containing medium was removed after the treatment period, and total RNA was harvested at the indicated times post-treatment, affording a t_1/2_ = 19.3 h. The observed half-life is in accord with reported half-lives of miR-155, which range from 12.4–28 h^15^. **f**, Effect of **Ac-RiboTAC** (green), the RNase L recruiter lacking the RNA-binding module, and **pre-miR-155-Ctr** (red), a chimera with the less active regioisomer of the RNase L recruiter, on miR-155 biogenesis after a 48 h treatment as determined by RT-qPCR (*n* = 3 biological replicates). **g**, Effect of **pre-miR-155-RiboTAC** and **pre-miR-155-binder** on pre-miR-155 (left; *n* = 6 biological replicates) and mature miR-155 (right; *n* = 3 biological replicates for compound-treated cells; *n* = 6 biological replicates for vehicle-treated) levels in CFPAC-1 (pancreatic ductal adenocarcinoma) cells, as determined by RT-qPCR. **h**, Correlation of miRNome-wide changes of MDA-MB-231 cells treated with **pre-miR-155-RiboTAC** (100 nM) or **LNA-155** (50 nM) (*n* = 3 biological replicates). Comparison of normalized read counts for each miRNA between **pre-miR-155-RiboTAC** and **LNA-155** treatment (left). Effect of **pre-miR-155-RiboTAC** (100 nM, *n* = 3 biological replicates) on other miRNAs sharing the targeted A bulge (right). **i**, Correlation of transcriptome-wide changes of MDA-MB-231 cells treated with **pre-miR-155-RiboTAC** (100 nM) or **LNA-155** (50 nM) (*n* = 3 biological replicates). From left to right: (i) Volcano plot of transcriptome-wide changes of MDA-MB-231 cells treated with **pre-miR-155-RiboTAC** (100 nM) *vs*. vehicle and **LNA-155** (50 nM) *vs*. vehicle after a 48 h treatment period; (ii) comparison of normalized read counts for each gene between **pre-miR-155-RiboTAC** and **LNA-155** treatment; (iii) comparison of normalized % change for genes commonly affected by both **pre-miR-155-RiboTAC** and **LNA-155** treatment (*n* = 26 genes, right). All data are reported as the mean ± S.D. All p-values were calculated using a two-tailed Student’s t-test. Source data

In vitro, pre-miR-155-RIBOTAC recruited RNase L to pre-miR-155 and induced its cleavage at the predicted cleavage site—the 5′U**U**U/3′G**UC**A motif—in a dose-dependent manner (Extended Data Fig. 7a). Furthermore, cleavage was reduced, dose-dependently, after addition of increasing concentrations of pre-miR-155 binder (Extended Data Fig. 7a). Importantly, mutation of the **C1**-binding site or of the cleavage site abolished the activity of pre-miR-155-RIBOTAC, indicating that both sites are required for cleavage (Extended Data Fig. 7b). Similarly, Ac-RIBOTAC did not induce cleavage of pre-miR-155 (Extended Data Fig. 7b). These in vitro results, for both pre-miR-155-RIBOTAC and pre-miR-155-Ctr, were verified using a model of the pre-miR-155 A bulge labelled at the 5′ and 3′ ends with fluorescein and a black hole quencher, respectively (Extended Data Fig. 3f).

As in vitro studies suggested specific degradation of pre-miR-155 through the recruitment of RNase L by pre-miR-155-RIBOTAC, its effect on pri-, pre- and mature miR-155 levels was studied in MDA-MB-231 cells. Treatment with 100 nM of pre-miR-155-RIBOTAC reduced mature miR-155 levels in a time-dependent manner with no observed toxicity and a maximum reduction of 71 ± 10% after 48 h (Extended Data Fig. 7c,d and Supplementary Figs. 2 and 3). Furthermore, a dose-dependent reduction in all three species was observed after a 48 h treatment period, and each was rescued by co-treatment with pre-miR-155-binder (Fig. 2c), indicating that the two molecules compete for the same binding site. Moreover, removal of pre-miR-155-RIBOTAC in a wash-out study resulted in a time-dependent restoration of pre-miR-155 levels with a half-life of about 19.3 h (Extended Data Fig. 7e), in accordance with the reported turnover of miR-155 in cells^33^. Neither control molecule Ac-RIBOTAC nor pre-miR-155-Ctr affected pre- or mature miR-155 levels in cells (Extended Data Fig. 7f). Similarly, in CFPAC-1 cells—a pancreatic cancer cell line that overexpresses miR-155^34^—pre-miR-155-RIBOTAC reduced precursor and mature levels of miR-155 by around 20% and 65% respectively, whereas the pre-miR-155-binder-amide did not do so (Extended Data Fig. 7g).

To validate the mode of action for pre-miR-155-RIBOTAC, MDA-MB-231 cells were transfected with a short interfering RNA (siRNA) targeting RNase L or a control siRNA. Notably, RNase-L-siRNA ablated the ability of pre-miR-155-RIBOTAC to reduce miR-155 levels (Fig. 2d); compound activity is therefore dependent on this RNase. Furthermore, RNase L immunoprecipitation enriched pre-miR-155 by around 2.4-fold from MDA-MB-231 cells treated with pre-miR-155-RIBOTAC, but not pre-miR-155-Ctr, both as compared with treatment with vehicle (Fig. 2e). Thus, pre-miR-155-RIBOTAC directly engages the miR-155 precursor and recruits RNase L to cleave the transcript.

## pre-miR-155-RIBOTAC is selective

The selectivity of pre-miR-155-RIBOTAC for miR-155 was assessed miRNome-wide using RT–qPCR profiling. An analysis of 373 miRNAs expressed in MDA-MB-231 cells showed that pre-miR-155-RIBOTAC selectively reduced miR-155 levels in a similar manner as LNA-155 (Extended Data Fig. 7h), without significant effects on any other miRNAs (Fig. 2f), including those that have the same A bulge. Note that repression of a subset of these miRNAs was observed at pre-miR-155-amide-binder concentrations of higher than 1 μM, tenfold greater than the concentration of pre-miR-155-RIBOTAC (100 nM) (Extended Data Fig. 6f). To confirm these findings, the selectivity of pre-miR-155-RIBOTAC and LNA-155, an antagomir that targets mature miR-155-5p, was also assessed using miRNA-seq. Notably, both modalities have a similar effect across the miRNome (Extended Data Fig. 7h).

The miRNAs with the same A bulge—miR-18a, miR-101-1, miR-1226 (bound by** C1** in Chem-CLIP studies), miR-3945 and miR-4435 (bound by **C1** in Chem-CLIP studies)—also have potential RNase cleavage sites (Extended Data Fig. 6f). However, it appears that the miR-155 precursor harbours the superior RNase L cleavage site. In vitro, cleavage occurred at nucleotides U28–U30, within and adjacent to a 1 × 2 asymmetric loop juxtaposed to the apical hairpin loop. RNA secondary structure prediction indicates that this loop is probably dynamic and may form a large hairpin of single-stranded nucleotides with only a 1 kcal mol^−1^ difference in predicted free energy. Furthermore, the RNase L cleavage sites in these five other miRNAs are at different distances from the binding site than in miR-155 (7 bp), suggesting that distances between binding and degrader sites can be used to program selectivity (Extended Data Fig. 6f). Collectively, these studies show that both the RNA-binding site and the RNase L cleavage site are required for pre-miR-155-RIBOTAC to induce degradation of its target RNA.

**Extended Data Fig. 8:**
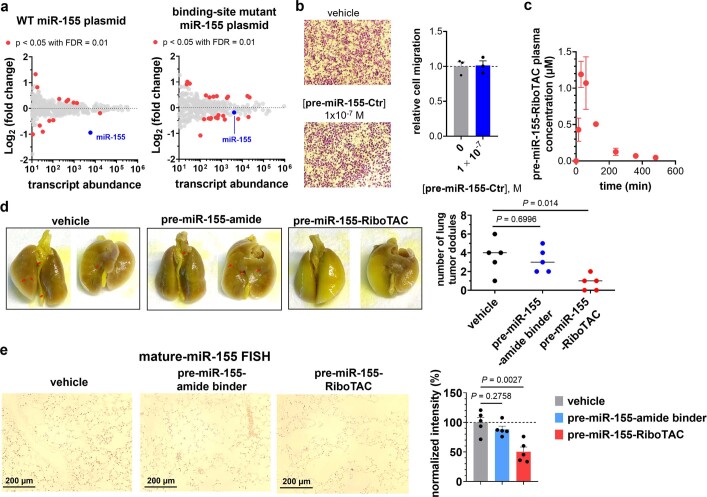
Cellular and *in vivo* activity of pre-miR-155-RiboTAC and its derivatives. **a**, Effect of **pre-miR-155-RiboTAC** (100 nM) on the miRNome of MDA-MB-231 cells forced to express WT pre-miR-155 (left; *n* = 3 biological replicates); and effect of **pre-miR-155-RiboTAC** (100 nM) on the miRNome of MDA-MB-231 cells forced to express the binding site mutant of pre-miR-155 (right; *n* = 3 biological replicates). **b**, Effect of **pre-miR-155-Ctr** on the migration of MDA-MB-231 cells (*n* = 3 biological replicates). **c**, Drug Metabolism and Pharmacokinetics (DMPK) analysis of **pre-miR-155-RiboTAC** in C57BL/6 mice (*n* = 3 mice per time point). **d**, In vivo treatment of **pre-miR-155-RiboTAC** (1 mg/kg, *q.o.d*., 30 days) decreased the number of lung nodules (nodules indicated in red) stained with Bouin’s solution (*n* = 5 mice). **e**, Lung tissue treated with **pre-miR-155-RiboTAC**, but not **pre-miR-155-amide binder**, exhibited decreased mature miR-155 levels, as determined by FISH (*n* = 5 mice). All data are reported as the mean ± S.D. Statistical significance was indicated by a Walds test (**a**) or two-tailed Student’s t-test (**b-e**). Source data

To assess the selectivity of pre-miR-155-RIBOTAC on the transcriptome, RNA-seq analysis was performed in MDA-MB-231 cells treated with either pre-miR-155-RIBOTAC or LNA-155 (Extended Data Fig. 7i). Among the 13,332 transcripts detected, 29 (0.22%) were significantly affected by pre-miR-155-RIBOTAC treatment (*P* < 0.05 and log_2_[fold change] > 1), with 1 upregulated and 28 downregulated. Notably, these 29 transcripts were also affected to a comparable extent by LNA-155 treatment (Extended Data Fig. 7i). When considering downstream targets of miR-155 predicted by TargetScanHuman (v.7.0)^35^ (*n* = 469), 307 (65%) were upregulated by pre-miR-155-RIBOTAC. A similar percentage (68%) of these 469 targets was also upregulated by LNA-155. Notably, 263 targets were upregulated by both pre-miR-155-RIBOTAC and LNA-155. A comparison of the normalized read counts for all genes between pre-miR-155-RIBOTAC and LNA-155 treatment showed a highly significant correlation, with *R* > 0.99 (Extended Data Fig. 7i). Collectively, these data indicate that pre-miR-155-RIBOTAC affects the transcriptome in similar ways to an oligonucleotide targeting miR-155 (LNA-155) and with limited off-target effects, although additional studies are needed to assess the selectivity of miRNA knockdown. Notably, pre-miR-155-RiboTAC selectively reduced miR-155 levels miRnome-wide in MDA-MB-231 cells forced to express wild-type pre-miR-155 but not those forced to express a binding site mutant (Extended Data Fig. 8a).

**Fig. 3:**
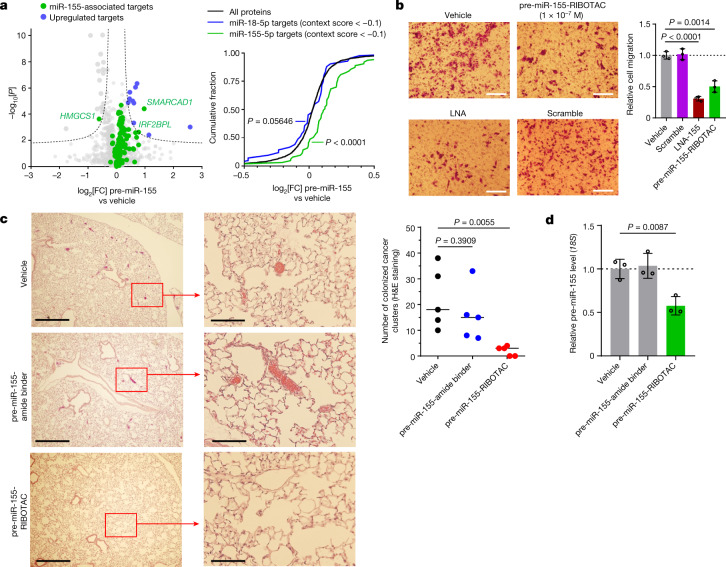
Pre-miR-155-RIBOTAC selectively degrades pre-miR-155 and reduces lung colonization in vivo. **a**, Left, proteome-wide changes in MDA-MB-231 cells treated with pre-miR-155-RIBOTAC (100 nM) versus vehicle (*n* = 3 biological replicates). Right, pre-miR-155-RIBOTAC significantly upregulated miR-155 related proteins (*n* = 98 proteins), as indicated by a Kolmogorov–Smirnov analysis (right) of their levels versus all proteins (*n* = 3 biological replicates). **b**, The effect of pre-miR-155-RIBOTAC on MDA-MB-231 cell migration (*n* = 3 biological replicates); 2 fields of view were quantified per replicate. Scale bars, 0.5 mm. **c**, pre-miR-155-RIBOTAC suppresses lung colonization in vivo, as determined by counting lung nodules (*n* = 5 mice) and by haematoxylin and eosin (H&E) staining (*n* = 5 mice; 2 fields of view were quantified per replicate). Scale bars, 1 mm (left) and 0.2 mm (right). **d**, The effect of pre-miR-155-amide-binder and pre-miR-155-RIBOTAC on pre-miR-155 levels in vivo, as determined by RT–qPCR using primers selective for human pre-miR-155 (*n* = 3 mice). Data are mean ± s.d. (**b** and **d**). Statistical significance was determined using a Wald’s test (**a**) or two-tailed Student’s *t*-tests (**b**–**d**). Source data

Upregulation of miR-155 modulates breast cancer cell migration and invasion by repressing SOCS1, which dampens cytokine signalling^26^. Indeed, pre-miR-155-RIBOTAC (100 nM) treatment increased SOCS1 levels by around 50% (Fig. 2g). These data were confirmed using a luciferase reporter fused to the SOCS1 3′ untranslated region (UTR), establishing both dose and time dependence of the RIBOTAC’s derepression of miR-155’s direct target (Fig. 2h). Global proteomics analysis confirmed that selective effects were induced by pre-miR-155-RIBOTAC. Of the 3,158 detectable proteins, 385 were upregulated and 98 of these are associated with miR-155 (Fig. 3a). Among these 98 proteins, the transcripts levels of 86 (88%) were also upregulated as determined using RNA-seq analysis. Notably, although SOCS1 was not detectable, the change in two miR-155-regulated proteins, IRF2BPL and SMARCAD1, was statistically significant (*P* < 0.01). Finally, inspection of proteins associated with pre-miR-18a, which was not affected by pre-miR-155-RIBOTAC, revealed that those that were detectable (*n* = 42 out of 264) were unaffected by treatment with this RIBOTAC (Fig. 3a).

**Extended Data Fig. 9:**
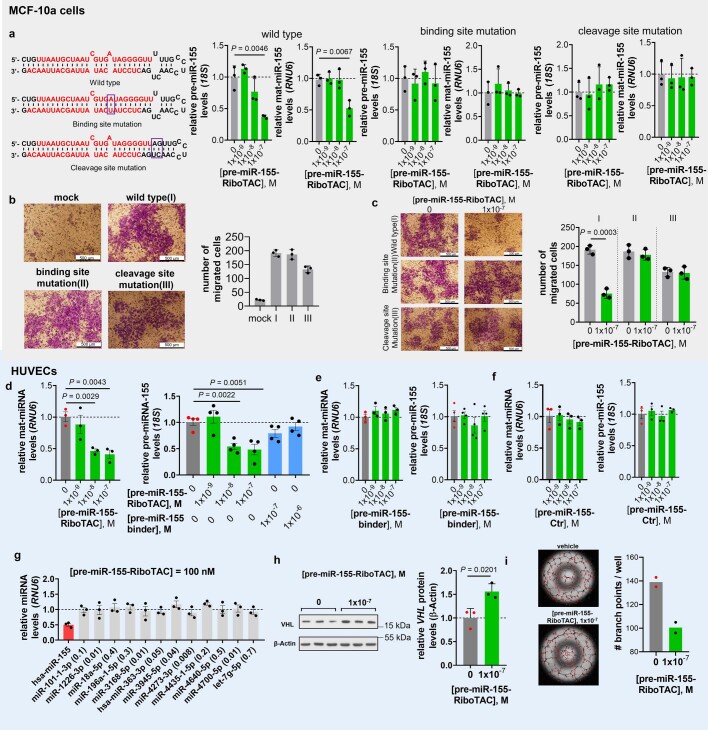
Cellular activity of pre-miR-155-RiboTAC in MCF-10a cells overexpressing miR-155 and in HUVECs. **a**, Effect of **pre-miR-155-RiboTAC** on pre- and mature miR-155 in MCF-10a cells forced to express wild-type pre-miR-155, a binding site mutant of pre-miR-155 (A bulge to AU base pair), or a RNase L cleavage site mutant (pyrimidine-rich asymmetric loop mutated to base pairs) (*n* = 3 biological replicates). **b**, Forced expression of pre-miR-155, a mutant pre-miR-155 in which **pre-miR-155-binder**’s binding site has been mutated, or a mutant pre-miR-155 in which the RNase L cleavage site has been abolished enhances the migratory nature of MCF-10a cells, a model of healthy breast epithelium (*n* = 3 biological replicates). **c**, **pre-miR-155-RiboTAC** impairs the migration of MCF-10a cells forced to express WT pre-miR-155 but not those forced express the binding site or cleavage site mutants (*n* = 3 biological replicates). **d**, **left**: Effect of **pre-miR-155-RiboTAC** on mature miR-155 levels in HUVECs, as determined by RT-qPCR (*n* = 3 biological replicates). **right**: Effect of **pre-miR-155-binder** on the cleaving capacity of **pre-miR-155-RiboTAC**, as assessed by measuring pre-miR-155 levels by RT-qPCR (*n* = 4 biological replicates). **e**, Effect of **pre-miR-155-binder** on mature and pre-miR-155 levels in HUVECs, as determined by RT-qPCR (*n* = 3 biological replicates for mature miR-155; *n* = 4 biological replicates for pre-miR-155). **f**, Effect of control RiboTAC **pre-miR-155-Ctr** on mature and pre-miR-155 levels in HUVECs, as determined by RT-qPCR (*n* = 3 biological replicates). **g**, Effect of **pre-miR-155-RiboTAC** (100 nM) on the levels of mature miRNAs that harbour miR-155’s A bulge in their precursors in HUVECs, as determined by RT-qPCR (*n* = 3 biological replicates). **h**, Representative Western blot and quantification thereof of the effect of **pre-miR-155-RiboTAC** on pre-miR-155’s downstream target, Von-Hippel Lindau (VHL) (*n* = 3 biological replicates). **i**, Effect of **pre-miR-155-RiboTAC** on the angiogenic capacity of HUVECs (*n* = 2 biological replicates). All data are reported as the mean ± S.D. All p-values were calculated using a two-tailed Student’s t-test. Source data

The cleavage of pre-miR-155 and subsequent upregulation of SOCS1 suggested that pre-miR-155-RIBOTAC may inhibit the migration of MDA-MB-231 cells. Indeed, treatment with pre-miR-155-RIBOTAC, but not with pre-miR-155-Ctr, reduced MDA-MB-231 cell migration by around 50% (Fig. 3b and Extended Data Fig. 8b). To confirm that these effects of pre-miR-155-RIBOTAC are due to abrogation of the miR-155 circuit, MCF-10a cells—a model of normal breast epithelial cells—were engineered to express either wild-type pre-miR-155 or a mutant in which the **C1**-binding site was abolished by mutation of the A bulge to a base pair (Extended Data Fig. 9a). Forced expression of both pre-miRNAs increased the migratory ability of MCF-10a cells (Extended Data Fig. 9b), indicating that miR-155 indeed contributes to this phenotype as previously reported^28,36^. Notably, treatment of MCF-10a cells expressing WT, but not mutant, pre-miR-155 with pre-miR-155-RIBOTAC reduced pre-miR-155 levels as well as the migration of these cells (Extended Data Fig. 9a–c).

Complementary analysis of pre-miR-155-RIBOTAC was performed in human umbilical vein endothelial cells (HUVECs), in which miR-155 controls angiogenesis through modulation of the Von Hippel Lindau (VHL) protein^37^. Treatment with pre-miR-155-RIBOTAC (100 nM) downregulated miR-155 levels to a similar extent to that observed in MDA-MB-231 cells, whereas pre-miR-155-binder and pre-miR-155-Ctr were inactive (Extended Data Fig. 9d–f). Moreover, there were no effects of pre-miR-155-RIBOTAC on the levels of other miRNAs with pre-miR-155-binder-binding sites (Extended Data Fig. 9g). Finally, reduction of miR-155 by pre-miR-155-RIBOTAC boosted VHL protein levels by around 50% and reduced tubule branching by approximately 35%, indicating reduced angiogenic ability (Extended Data Fig. 9h,i).

To assess the ability of pre-miR-155-RIBOTAC to inhibit miR-155-mediated colonization of breast cancer to the lungs, mice were injected through the tail vein with MDA-MB-luc cells^38^. After 5 days, mice were treated with pre-miR-155-RIBOTAC (1 mg per kg, every other day) by intraperitoneal injection. The dosage was determined by drug metabolism and pharmacokinetics studies (*C*_max_ = 1.2 µM in the plasma with half-life of 1.8 h; Extended Data Fig. 8c). As evidenced by the reduced number of nodules, lung colonization was significantly inhibited in the pre-miR-155-RIBOTAC-treated group versus the vehicle- or pre-miR-155-binder-amide-treated cohorts (Fig. 3 and Extended Data Fig. 8d). Histological staining of lung tissue sections showed marked reductions in tumour burden after treatment with the RIBOTAC (Fig. 3c). As mouse pre-miR-155 has a different secondary structure lacking the binding site for pre-miR-155-RIBOTAC, fluorescence in situ hybridization (FISH) and RT–qPCR were used to assess the levels of human mature and pre-miR-155, respectively. There were marked reductions in mature (Extended Data Fig. 8e) and pre- (Fig. 3d) miR-155 in human TBNC cells in mouse lung tissues after treatment with pre-miR-155-RIBOTAC, whereas treatment with pre-miR-155-binder-amide had no significant effect. Collectively, these results demonstrated that pre-miR-155-RIBOTAC can impair miR-155-mediated tumour colonization in vivo.

## RIBOTACs targeting *JUN* and *MYC* mRNAs

**Fig. 4:**
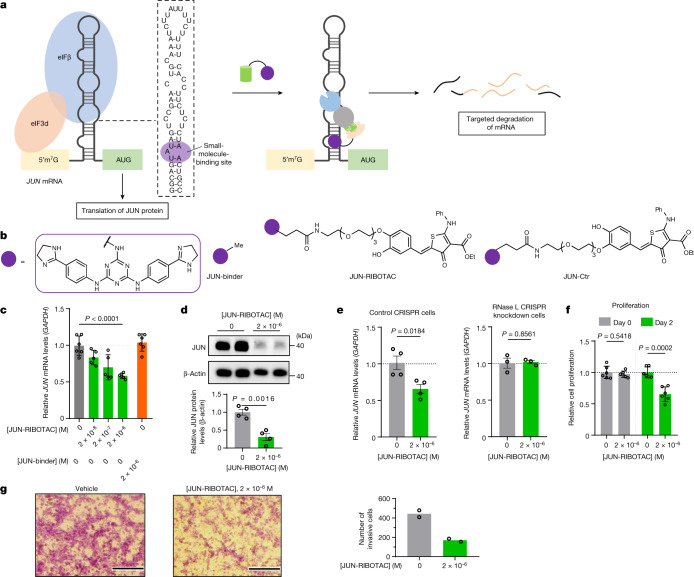
JUN-RIBOTAC impairs pancreatic tumour cell proliferation and migration by selectively degrading *JUN* mRNA. **a**, Schematic of *JUN* degradation by targeting the *JUN* IRES. **b**, The structures of compounds used to target *JUN* mRNA. **c**, The effect of JUN-RIBOTAC and JUN-binder on *JUN* mRNA levels in MIA PaCa-2 cells after treatment for 72 h, as determined using RT–qPCR (*n* = 6 biological replicates). **d**, The effect of JUN-RIBOTAC on JUN protein levels in MIA PaCa-2 cells (*n* = 4 biological replicates). **e**, The effect of JUN-RIBOTAC on *JUN* mRNA levels in MIA PaCa-2 cells in which RNase L was knocked down by CRISPR (*n* = 3 biological replicates) and in the corresponding MIA PaCa-2 control cell line in which CRISPR editing was performed using a scrambled guide RNA (*n* = 4 biological replicates), as determined using RT–qPCR. **f**, The effect of JUN-RIBOTAC on the proliferation of MIA PaCa-2 cells (*n* = 6 biological replicates). **g**, The effect of JUN-RIBOTAC on the invasiveness of MIA PaCa-2 cells, as determined using a Boyden chamber assay (*n* = 2 biological replicates; 2 fields of view were quantified per replicate). Data are mean ± s.d. (**c**–**f**). Statistical significance was determined using two-tailed Student’s *t*-tests (**d**–**f**) and one-way analysis of variance (ANOVA) adjusted for multiple comparisons (**c**). Source data

**Fig. 5:**
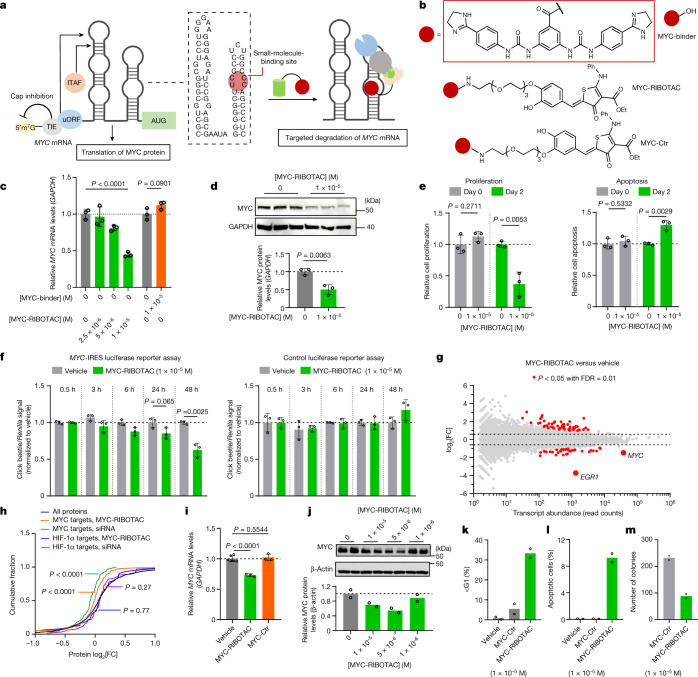
MYC-RIBOTAC selectively targets MYC in an RNase-L-dependent manner. **a**, Schematic of the targeted degradation of the *MYC* IRES. **b**, Compound structures. **c**, The effect of MYC-binder and MYC-RIBOTAC on *MYC* mRNA levels in HeLa cells, as determined using RT–qPCR. *n* = 3 biological replicates. **d**, The effect of MYC-RIBOTAC on MYC protein levels in HeLa cells (*n* = 3 biological replicates). **e**, The effect of MYC-RIBOTAC on the proliferation (left) and apoptosis (right) of HeLa cells (*n* = 3 biological replicates). **f**, The effect of MYC-RIBOTAC on *MYC* IRES luciferase reporter in HEK293T cells (left) or on a control reporter lacking the IRES (right)(*n* = 3 biological replicates). **g**, Transcriptome-wide changes in HeLa cells treated with MYC-RIBOTAC (10 μM) after treatment for 48 h (*n* = 3 biological replicates). *EGR1* is a well-known downstream target of MYC^50^. **h**, Cumulative distribution analysis of the effect of MYC-RIBOTAC and a *MYC*-selective siRNA on 87 well-validated downstream targets of MYC, or on the downstream targets of HIF-1α, as indicated by a Kolmogorov–Smirnov analysis of their levels relative to all proteins (*n* = 3 biological replicates). **i**, The effect of MYC-Ctr and MYC-RIBOTAC on *MYC* mRNA levels in Namalwa Burkitt lymphoma cells (*n* = 3 biological replicates) compared with the vehicle (*n* = 6 biological replicates). **j**, The effect of MYC-RIBOTAC on MYC protein levels in Namalwa cells (*n* = 2 biological replicates). **k**, The effect of MYC-RIBOTAC on the cell cycle of Namalwa cells. *n* = 2 biological replicates. **l**, The ability of MYC-RIBOTAC or MYC-Ctr to induce apoptosis of Namalwa cells (*n* = 2 biological replicates). **m**, The effect of MYC-RIBOTAC on colony formation of Namalwa cells (*n* = 2 biological replicates). Data are mean ± s.d. (**c**–**f** and **i**). Statistical significance was determined using a one-way ANOVA adjusted for multiple comparisons (**c**), two-tailed Student’s *t*-tests (**d**–**i**), Wald’s test (**g**), or Kolmogorov–Smirnov test (**h**). Source data

To test whether our approach to convert biologically silent interactions into bioactive ones is broadly applicable, we designed RIBOTACs for two other oncogenic RNAs—*JUN* and *MYC*. Notably, both oncoproteins are intrinsically disordered and are considered to be undruggable. Their transcripts harbour an internal ribosomal entry site (IRES) in their 5′ untranslated regions (UTRs) that can drive cap-independent translation of MYC^39,40^ and cap-dependent specialized translation initiation of JUN^41,42^. To determine whether these mRNAs form targetable structures in their 5′ UTRs, we coupled Inforna with the state-of-the-art RNA structure computing program, ScanFold^43^, which identifies regions of unusual thermodynamic stability within an RNA, an indicator of function and potential evolutionary conservation. Indeed, both mRNAs form a thermodynamically stable region in their IRESs that house structures that are targetable with small molecules and that are proximal to potential RNase-L-cleavage sites (Figs. 4a and 5a).

**Extended Data Fig. 10:**
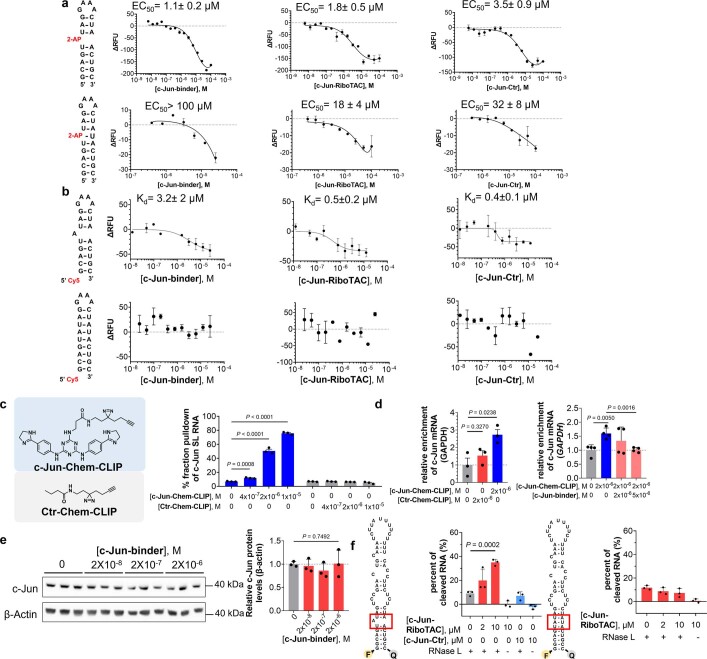
In vitro and in cellular characterization of c-Jun-binder and its derivatives. **a**, Representative binding curves to measure the affinity of **c-Jun-binder**, **c-Jun-RiboTAC**, and **c-Jun-Ctr** for a model of the c-*Jun* RNA where the A bulge has been replaced with the fluorescent adenine mimic, 2-aminopurine (2AP) (*n* = 2 independent experiments). **b**, Representative binding curves to measure the affinity of **c-Jun-binder**, **c-Jun-RiboTAC** and **c-Jun-Ctr** for a Cy5-labelled model of the c-*Jun* IRES and a fully paired RNA control (*n* = 2 independent experiments). The fully paired RNA was created by mutating the A bulge binding site to an AU pair. **c**, Chemical structure of **c-Jun-Chem-CLIP** probe and control probe. Pull-down of the c-*Jun* mRNA in vitro by **c-Jun-Chem-CLIP** or **Ctr-Chem-CLIP** (*n* = 3 replicates). **d**, **left**: Target engagement studies completed by Chem-CLIP in MIA PaCa-2 cells (*n* = 3 biological replicates). Quantification of the enrichment of c-*Jun* mRNA by **c-Jun-Chem-CLIP** or **Ctr-Chem-CLIP** in pulled-down fraction as compared to RNA not subjected to pull-down; **right**: Competitive Chem-CLIP experiments in which MIA PaCa-2 cells were co-treated with **c-Jun-Chem-CLIP** at a constant concentration and increasing concentrations of **c-Jun-binder**, which depletes c-*Jun* mRNA in the pull-down fractions in a dose dependent fashion (*n* = 4 biological replicates). **e**, Effect of **c-Jun-binder** on c-JUN protein levels in Mia-PaCa-2 cells (*n* = 3 biological replicates). **f**, **c-Jun-RiboTAC** induces in vitro RNA cleavage with WT c-*Jun* construct (left), while no cleavage of the mutated base pair control (right) was observed (*n* = 3). All data are reported as the mean ± S.D. All p-values were calculated using a two-tailed Student’s t-test. Source data

The *JUN* IRES comprises a hairpin with various internal loops and bulges embedded in its stem that is recognized by eukaryotic initiation factor 3 (eIF3) (Fig. 4a). Inforna identified a small-molecule candidate for the A bulge (5′U**A**U/3′A_A), named JUN-binder (Fig. 4b). Notably, this A bulge is adjacent to a pyrimidine-rich internal loop (5′G**UCC**G/3′C**UCU**C), a probable substrate for RNase L. Using the 2AP-binding assay, JUN-binder binds to this loop with a half-maximal effective concentration (EC_50_) of 1.1 ± 0.2 µM, with no observable binding to a control RNA in which the bulged nucleotide was converted to a base pair (EC_50_ > 50 µM) (Extended Data Fig. 10a). These data were corroborated by an orthogonal binding assay using Cy-5-labelled RNAs (Extended Data Fig. 10b). Target engagement was validated both in vitro and in the pancreatic cancer cell line MIA PaCa-2 (Extended Data Fig. 10c,d) using Chem-CLIP and its competitive variant. Finally, although in vitro binding assays and target validation confirmed engagement of the *JUN* IRES with JUN-binder, this molecule was biologically inert, with no effects on *JUN* mRNA or protein levels up to concentrations of 2 µM (Fig. 4c and Extended Data Fig. 10e).

Given these findings, the RIBOTAC strategy was applied, affording JUN-RIBOTAC and the corresponding control molecule with the less active RNase L-recruiting regioisomer, JUN-Ctr (Fig. 4b). The binding affinities of these molecules were studied in the same manner as for the JUN-binder, using 2AP- or Cy5-labelled RNAs. JUN-RIBOTAC and JUN-Ctr bind to the *JUN* IRES similarly in the Cy5 assay with *K*_d_ values of 0.5 ± 0.2 µM and 0.4 ± 0.1 µM, respectively, whereas there was no saturable binding to a fully paired control RNA (Extended Data Fig. 10b). To test whether JUN-RIBOTAC induced cleavage of the *JUN* IRES by recruiting RNase L, the *JUN* IRES was labelled with a fluorophore and quencher at its 5′ and 3′ ends, respectively (Extended Data Fig. 10f). JUN-RIBOTAC increased fluorescence intensity, suggesting its ability to induce RNase L cleavage of the *JUN* IRES, while having no effect on a mutant in which the A bulge was converted to an AU pair; furthermore, JUN-Ctr did not induce cleavage of the wild-type IRES (Extended Data Fig. 10f).

In MIA PaCa-2 cells, JUN-RIBOTAC reduced *JUN* mRNA levels in a dose-dependent manner up to about 40% and reduced protein levels by around 75% at a dose of 2 µM (Fig. 4c,d and Supplementary Fig. 4), whereas JUN-binder was inactive (Extended Data Fig. 10e). To confirm the mode of action, we evaluated the activity of JUN-RIBOTAC in MIA PaCa-2 cells in which RNase L was knocked down by CRISPR (Supplementary Fig. 5). As predicted, JUN-RIBOTAC was inactive in the RNase L CRISPR cell line, whereas it showed around a 40% decrease in *JUN* mRNA level in the CRISPR control cell line (Fig. 4e). JUN contributes to proliferative and invasive phenotypes in different tumour types, including pancreatic cancer^44–46^. The reduction in JUN protein levels by JUN-RIBOTAC in MIA PaCa-2 cells was sufficient to inhibit both cell proliferation (~40% at 2 µM; Fig. 4f) and invasion (~60% at 2 µM; Fig. 4g).

**Extended Data Fig. 11:**
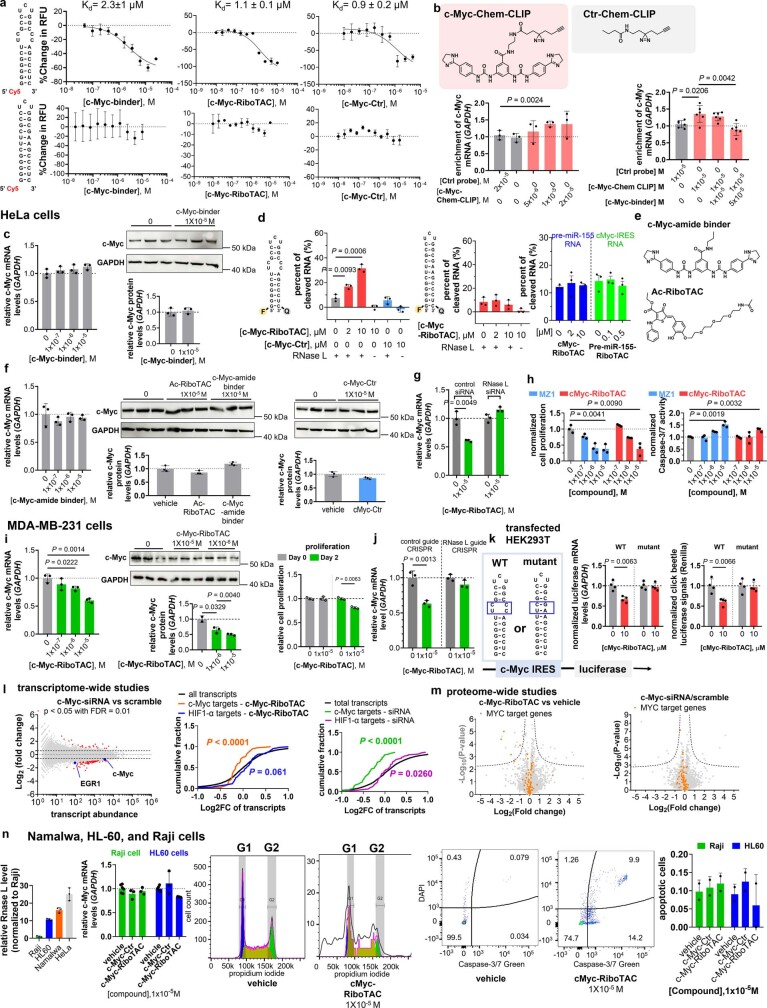
In vitro and cellular characterization of c-Myc-binder and its derivatives. **a**, Representative binding curves to measure the affinity of **c-Myc-binder**, **c-Myc-RiboTAC** and **c-Myc-Ctr** for a Cy5-labelled model of the c-Myc IRES 5’UUCG/3’ACCC 2×2 internal loop or a fully paired RNA control (*n* = 2 independent experiments). **b**, Chemical structures of **c-Myc-Chem-CLIP** and **Ctr-Chem-CLIP** probes (top) and target validation studies in HeLa cells (bottom). **c-Myc-Chem-CLIP** dose dependently enriches *MYC* mRNA (lower left; *n* = 3 biological replicates), which is competed by **c-Myc-binder** (lower right; *n* = 6 biological replicates). **c**, Effect of **c-Myc-binder** on *MYC* mRNA and protein levels in HeLa cells (*n* = 3 biological replicates). **d**, **c-Myc-RiboTAC** induces in vitro cleavage of the WT *MYC* IRES (left), while no cleavage of a mutated base pair control was observed (middle). **Pre-miR-155-RiboTAC** and **c-Myc-RiboTAC** have no effect on the non-cognate RNA (right) (*n* = 3 biological replicates for all panels). Concentrations were selected based on the cellular activity for the cognate target. **e**, Chemical structures of **c-Myc-amide-binder** and **Ac-RiboTAC**. **f**, Effect of **c-Myc-amide-binder**, **Ac-RiboTAC**, and **c-Myc-Ctr** on *MYC* mRNA and protein levels in HeLa cells (*n* = 3 biological replicates). **g**, Effect of siRNA knock-down of RNase L (~85%) on the activity of **c-Myc-RiboTAC** in HeLa cells (*n* = 3 biological replicates). **h**, Effects of **c-Myc-RiboTAC** and BRD4 degrader **MZ1** on the proliferation and apoptosis of HeLa cells upon 48 h treatment (*n* = 3 biological replicates). **i**, Effect of **c-Myc-RiboTAC** on *MYC* mRNA levels (left; n = 3 biological replicates), protein abundance (middle; (n = 2 biological replicates for vehicle (note that the third lane was excluded from quantification due to air bubble) and n = 3 biological replicates for compound-treated sampled), and proliferation (right; *n* = 3 biological replicates) in MDA-MB-231 cells. **j**, Effect of siRNA knockdown of RNase L on **c-Myc-RiboTAC**-mediated degradation of *MYC* mRNA in MDA-MB-231 cells (*n* = 3 biological replicates). **k**, Effect of **c-Myc-RiboTAC** on a *c-Myc*-IRES-luciferase reporter or a binding site mutant thereof in transfected HEK293T cells (*n* = 4 biological replicates). **l**, Transcriptome-wide changes observed in HeLa cells treated with vehicle, **c-Myc-RiboTAC**, scrambled siRNA, or *MYC* siRNA (*n* = 3 biological replicates). Volcano plots of transcriptome-wide changes of HeLa cells treated with *MYC*-siRNA (1 nM) *vs*. scrambled siRNA (1 nM) after a 48 h treatment period (left). Cumulative curves of *MYC* targets and HIF-1α targets in HeLa cells treated with **c-Myc-RiboTAC** (10 μM) *vs*. vehicle (middle) or c-Myc-siRNA (1 nM) *vs*. scrambled siRNA (1 nM) (right). **m**, Volcano plots of proteome-wide changes of HeLa cells treated with **c-Myc-RiboTAC** (10 μM) *vs*. vehicle (left) or *MYC*-siRNA (1 nM) *vs*. scrambled siRNA (1 nM) (right) after a 48 h treatment period (*n* = 3 biological replicates). **n**, Abundance of RNase L mRNA in various cell lines (left; *n* = 3 biological replicates). Effect of **c-Myc-Ctr** (10 μM) and **c-Myc-RiboTAC** (10 μM) on *MYC* mRNA levels in lymphoma or leukaemia cells, Raji, and HL-60 cells, respectively (middle left; *n* = 6 biological replicates for vehicle and *n* = 3 biological replicates for compound treatment) and on apoptosis (middle right and right; *n* = 2 biological replicates). Interestingly, these data suggest that RiboTAC activity is correlated with RNase L expression. All data are reported as the mean ± S.D. of biologically independent replicates. Statistical significance was calculated using a two-tailed Student’s t-test (**b-h, n**), Wald’s test (**i, left and m**), or Kolmogorov–Smirnov test (**i, middle and right**). Source data

The oncogenic transcription factor MYC coordinately regulates the cell cycle, proliferation and metabolism in many cancer types, including in cervical cancer as exemplified by HeLa cells^47,48^. Notably, the *MYC* IRES also forms a thermodynamically stable structure comprising two hairpins and three internal loops (Fig. 5a)^19–22^ and, fortuitously, one internal loop, 5′U**UC**G/3′A**CC**C, is targetable with a molecule from our database, named MYC-binder (Fig. 5b). Moreover, this loop is adjacent to a pyrimidine-rich hairpin loop (Fig. 5a). The phenylene bis-phenylurea group of MYC-binder interacts with the internal loop with a *K*_d_ of 2.3 ± 1 µM, as determined using a fluorescence-based binding assay with a Cy5-labelled RNA; no binding was observed to a fully paired RNA (Extended Data Fig. 11a). Furthermore, the derived MYC-Chem-CLIP probe engaged endogenous *MYC* mRNA in HeLa cells in a dose-dependent manner (about 50% enrichment at 20 µM; Extended Data Fig. 11b), and this enrichment was depleted by addition of competing concentrations of MYC-binder (Extended Data Fig. 11b). Finally, although the *MYC* IRES is a functional site, engagement by MYC-binder did not affect MYC protein levels or *MYC* mRNA levels (Fig. 5c and Extended Data Fig. 11c).

As MYC-binder was biologically inert, it was appended with the heterocyclic RNase-L-recruiter to afford MYC-RIBOTAC, or with the less active regioisomer to afford MYC-Ctr (Fig. 5b). MYC-RIBOTAC and MYC-Ctr bind to the *MYC* 5′U**UC**G/3′A**CC**C internal loop similarly in the Cy5 assay with *K*_d_ values of 1.1 ± 0.1 µM and 0.9 ± 0.2 µM, respectively, whereas no saturable binding was observed to a fully paired control RNA (Extended Data Fig. 11a). Akin to the dual-labelled *JUN* IRES model (Extended Data Fig. 9f), an in vitro fluorescent reporter was created to study whether MYC-RIBOTAC could recruit RNase L and induce *MYC* IRES degradation. Indeed, MYC-RIBOTAC but not MYC-Ctr recruited RNase L to the *MYC* IRES, as evidenced by the resulting increase in fluorescence, and again no change in fluorescence was observed after incubation of the fully paired RNA with either molecule (Extended Data Fig. 11d). Notably, pre-miR-155-RIBOTAC had no effect on the *MYC* IRES RNA and vice versa (Extended Data Fig. 11d), supporting the selectivity of the RIBOTAC approach with different RNA-binding modules.

In contrast to MYC-binder, MYC-RIBOTAC decreased the abundance of *MYC* mRNA in HeLa cells in a dose-dependent manner, up to around 50% at a 10 µM dose (Fig. 5c and Supplementary Figs. 6 and 7) with a concomitant reduction in MYC protein levels (Fig. 5d). A reduction in MYC protein was associated with reduced HeLa cell proliferation and the induction of apoptosis (Fig. 5e). Notably, MYC-Ctr and two control molecules in which the RNase-L-recruiting module (Ac-RIBOTAC) or the RNA-binding module was conjugated to propylamine (MYC-amide-binder) were inactive (Extended Data Fig. 11e,f). To confirm that reduction of *MYC* mRNA levels by MYC-RIBOTAC was dependent on RNase L, we knocked down RNase L in HeLa cells with an siRNA. As expected, the effect of MYC-RIBOTAC on *MYC* mRNA levels was ablated in cells after RNase L knockdown (Extended Data Fig. 11g). Notably, the proteolysis-targeting chimera (PROTAC) MZ-1 targets the protein bromodomain containing 4 (BRD4), a known regulator of *MYC* transcription; its degradation by MZ1 therefore leads to downregulation of *MYC* mRNA and protein levels^49^ (Supplementary Fig. 8). We therefore assessed the antiproliferative and apoptotic effects of MZ1 and MYC-RIBOTAC from 0.1 to 10 µM in HeLa cells. The two chimeras inhibited proliferation to a similar extent at the 10 µM dose. However, MZ1 is more potent at provoking cell death—1 µM MZ1 led to reductions in cell viability that were observed in cells treated with 10 µM of MYC-RIBOTAC (Extended Data Fig. 11h). Finally, MYC-RIBOTAC showed a similar effect on the reduction of *MYC* mRNA and protein levels as well as cellular phenotype in MDA-MB-231 breast cancer cells (Extended Data Fig. 11i). Similarly, the effect of MYC-RIBOTAC on *MYC* mRNA levels was ablated in MDA-MB-231 cells after knockdown of RNase L (Extended Data Fig. 11j). To verify these results, we studied the effect of MYC-RIBOTAC on a *MYC* IRES-luciferase reporter assay in transfected HEK293T cells. MYC-RIBOTAC reduced luciferase levels (~40%), yet had no effect on a control luciferase reporter lacking the *MYC* IRES (Fig. 5f). Furthermore, mutating the IRES in the luciferase reporter such that the 5′U**UC**G/3′A**CC**C binding site forms base pairs (5′U**UC**G/3′A**AG**C) rendered the RIBOTAC inactive at both the mRNA and protein levels (Extended Data Fig. 11k).

To evaluate the selectivity of MYC-RIBOTAC transcriptome-wide in HeLa cells, we performed global RNA-seq analysis. Among 21,027 transcripts detected, 84 (0.40%) were significantly affected, with 51 transcripts upregulated and 33 downregulated (Fig. 5g and Extended Data Fig. 11l). Notably, the most significantly downregulated transcript was *EGR1*, a known downstream target of MYC^50^ (Fig. 5g). Similar results were obtained from the transcriptome-wide analysis of the *MYC* siRNA, which significantly affected 90 (0.38%) transcripts among the 23,741 detected (Extended Data Fig. 11l). Cumulative distribution analysis of 837 well-validated MYC-target genes^51–53^ demonstrated a significant decrease in these transcripts after treatment with MYC-RIBOTAC (*P* < 0.001) or the *MYC* siRNA (*P* < 0.001) (Extended Data Fig. 11l). By contrast, the same analysis of 63 downstream targets of HIF-1α, another transcription factor that recognizes a similar DNA sequence (ACGTG)^54^ to MYC (CCACGTG)^55^, showed no significant change by MYC-RIBOTAC (Extended Data Fig. 11l).

The selectivity of MYC-RIBOTAC across the transcriptome was mirrored in the proteome. Only 28 (1.0%) out of 2,769 detectable proteins were significantly affected (16 downregulated and 12 upregulated; Extended Data Fig. 11m). Notably, 4 out of the 16 downregulated proteins are direct downstream targets of *MYC*. Eight of the 16 downregulated proteins are associated with the ribosome pathway, one of the main functional pathways of MYC-target genes^53^. Cumulative distribution analysis of the proteome data showed results consistent with those on the transcriptome, where MYC-RIBOTAC treatment significantly downregulated the protein levels of downstream targets of *MYC*, but not those of HIF-1α (Fig. 5h and Extended Data Fig. 11m). Collectively, these results confirm that MYC-RIBOTAC is selective transcriptome- and proteome-wide.

To further evaluate MYC-RIBOTAC in other relevant tumour cell lines, the human Burkitt lymphoma cell lines Namalwa and Raji and the leukaemia cell line HL-60 were tested, in which MYC is overexpressed due to translocations or amplification. Notably, the RNase L expression level varies among the cell lines by 25-fold—it is highly expressed in HeLa cells and has low expression in Raji Burkitt lymphoma cells (Extended Data Fig. 11n). The activity of MYC-RIBOTAC tracked with RNase L expression: *MYC* mRNA levels were reduced by around 50% in HeLa cells (Fig. 5c), 25% in Namalwa cells (Fig. 5i), 10% in HL-60 cells and there was no reduction in Raji cells (Extended Data Fig. 11n). As expected, MYC-Ctr was inactive in Namalwa Burkitt lymphoma cells (Fig. 5i). By contrast, MYC-RIBOTAC reduced *MYC* protein levels by around 50% after treatment with 5 µM (Fig. 5j), induced cell cycle arrest and provoked apoptosis (Fig. 5k,l, Extended Data Fig. 11n and Supplementary Fig. 9) and reduced colony formation by about 50% (Fig. 5m). Notably, MYC-Ctr, which cannot degrade *MYC* mRNA, had no effect in any of these assays (Fig. 5k–m).

## Programming small-molecule RNA degraders

Here we have demonstrated that inactive, biologically inert small molecules engaging an RNA target can be programmed into bioactive degraders. Applying this strategy to a miRNA precursor demonstrated that the RIBOTAC degrader reduced the levels of the miRNA in multiple cell lines covering two diseases and in vivo. This degradation inhibited cellular phenotypes and colonization processes that are driven by this miRNA. As a further proof of concept, this strategy was extended to convert biologically silent interactions into bioactive ones for two additional oncogenic targets, *JUN* and *MYC* mRNAs. Their RIBOTAC degraders, derived from binders designed by Inforna, degraded the desired targets and disabled transcriptional and proteomic programs driven by these oncoproteins.

Collectively, this study provides examples of converting functionally silent binders into potent degraders that can selectively downregulate disease-causing RNAs. To our knowledge, all previously reported bioactive small molecules that target miRNAs bind in or nearby functional sites, which in this study constituted only around 30% of human miRNAs. Much less is known about functional sites in mRNAs, making the RIBOTAC strategy perhaps even more important for this class of targets. Complementarily, about 50% of a given RNA target is unstructured, providing ideal target sites for designer oligonucleotides. Small molecules provide a means to target the remaining structured regions, which is probably no longer limited to functional regions using the RIBOTAC approach.

## Methods

### General methods

All DNA templates and primers were purchased from Integrated DNA Technologies (IDT) and used without further purification. Chemically synthesized RNA oligonucleotides and oligonucleotide competitors were obtained from Dharmacon and deprotected according to the manufacturer’s recommended protocol. After deprotection, the RNAs were desalted using a PD-10 Sephadex column (GE Healthcare) according to the manufacturer’s protocol. All oligonucleotide concentrations were determined by their absorption at 260 nm at 90 °C and the corresponding extinction coefficient provided by the manufacturer. The compound MZ1 was purchased from MedChemExpress.

All autoradiographical images and ethidium bromide staining images were obtained on the Typhoon FLA9500 variable mode imager (GE Healthcare). The Western blot images were obtained using the AFP Imaging Mini-Medical/90 system. These images were quantified using Image J (v.1.8.0_112). All numerical calculations were performed using Microsoft Excel (Office 365) and GraphPad 8 (v.8.0.2).

### Statistics and reproducibility

Experimental results shown as representative blots or gel autoradiograms were successfully replicated two or more times to ensure the reproducibility of the reported findings. The number and type of replicates as well as statistical significance are provided in the figure legends.

### In vitro Chem-CLIP binding site mapping

Pre-miR-155 RNA (500 pmol) was folded in 1× folding buffer (FB; 20 mM HEPES, pH 7.5, 150 mM NaCl, and 5 mM KCl) by heating at 95 °C for 30 s followed by cooling on ice for 5 min. Next, 50 nM of pre-miR-155-Chem-CLIP was added to the folded pre-miR-155, and the sample was incubated at 37 °C overnight. Cross-linked pre-miR-155 was captured on Dynabeads and eluted according to the manufacturer’s protocol. Reverse transcription of the pulled-down RNA was performed using a gene-specific primer (5′-CAGACGTGCTCTTCCGATCTCTGTTAATGCTAATAT-3′) and Superscript III (SSIII) according to the manufacturer’s protocol. The RT reaction was processed using RNAClean XP (Beckman Coulter) according to the manufacturer’s protocol. A ssDNA adaptor (5′phosphate-NNNAGATCGGAAGAGCGTCGTGTAG-3C spacer) was ligated to the purified RT product using T4 RNA ligase I (New England BioLabs (NEB)) and then RNA Clean XP was added according to the manufacturer’s protocol. The ligated cDNA was amplified using Phusion polymerase (NEB) using the following primers: 5′-CTACACGACGCTCTTCCGATCT-3′ and 5′-CAGACGTGCTCTTCCGAT-3′. The PCR reaction was purified with a denaturing 10% acrylamide gel and ethanol precipitation. The purified PCR product was cloned into NEB’s pminiT 2.0 vector according to the manufacturer’s protocol and sequenced by Eton Biosciences.

### Tissue culture

MDA-MB-231 triple-negative breast cancer cells (HTB-26; ATCC) were cultured in RPMI medium with l-glutamine and 25 mM HEPES (Corning; 10-041) supplemented with 10% (v/v) fetal bovine serum (FBS; Sigma-Aldrich; F2442) and 1× penicillin–streptomycin solution (Gibco; 15140122).

MCF-10a breast epithelial cells (CRL-10317; ATCC) were cultured in DMEM/F12 50/50 medium with l-glutamine and 15 mM HEPES (Corning; 10-092-CV) containing 20% (v/v) FBS, 1× antibiotic–antimycotic solution (Corning; 30-004-CI), 20 ng ml^−1^ human epidermal growth factor (Pepro Tech; GMP100-15), 100 μg ml^−1^ insulin and 0.5 mg ml^−1^ hydrocortisone (Sigma-Aldrich; H0888).

CFPAC1 ductal adenocarcinoma epithelial cells (CRL-1918; ATCC) were cultured in 1× IMDM medium (Gibco, 12440053) with 10% (v/v) FBS and 1× antibiotic–antimycotic solution.

HUVECs (Lonza, CC-2517) were cultured in EGM prepared using the EGM-2 bullet kit (Lonza; CC-3162) according to the manufacturer’s protocol.

HeLa cells (CCL-2, ATCC) and HEK293T cells (CRL-3216, ATCC) were cultured in 1× DMEM medium with 4.5 g l^−1^ glucose (Gibco, 11965092) supplemented with 2 mM glutamine or 1% Glutagro (Corning; 25-015-CI), 1× penicillin–streptomycin solution and 10% (v/v) FBS.

MIA PaCa-2 pancreatic adenocarcinoma cells (CRL-1420; ATCC) and CRISPR lines derived from MIA PaCa-2 pancreatic adenocarcinoma cells (Supplementary Fig. 6) were cultured in 1× DMEM medium with 4.5 g l^−1^ glucose supplemented with 1% Glutagro, 1× penicillin–streptomycin solution and 10% (v/v) FBS.

Namalwa (CRL-1432) and Raji (CCL-86) Burkitt lymphoma cells, and HL-60 (CCL-240) myeloid leukaemia cells, were obtained from ATCC and were cultured in RPMI medium with l-glutamine and 25 mM HEPES supplemented with 10% (v/v) FBS and 1× penicillin–streptomycin solution.

The passage number for all cell lines was <20, except for HUVECs (<6) and HeLa cells (<30). All cells were checked to be free of mycoplasma contamination before conducting experiments using a PCR Mycoplasma Test Kit (PromoCell).

### Forced expression of pre-miR-155 in MCF-10a cells

The plasmid to overexpress wild-type pre-miR-155 were purchased from Genecopoeia (HmiR0358) and the mutant plasmids were custom synthesized by GeneScript. MCF-10a cells were seeded at around 70% confluency in 60 mm dishes and transfected with a plasmid (2 µg per dish) encoding either wild-type or mutant pre-miR-155 (Extended Data Fig. 9a) using Lipofectamine 3000 (Invitrogen) according to the manufacture’s protocol. The cells were incubated in the transfection cocktail for 6 h followed by changing to fresh growth medium and incubated for an additional 16 h. Cells were then trypsinized and seeded for biological experiments as described in the following sections.

### Cellular Chem-CLIP and competitive Chem-CLIP analysis of pre-miR-155, MYC and JUN

#### General protocol for cellular Chem-CLIP

After compound treatment as specified for each target below, cells were washed once with 1× DPBS and irradiated by ultraviolet light for 15 min. Total RNA was extracted using the Zymo Quick-RNA MiniPrep Kit according to the manufacturer’s protocol. The input RNA (10 µg) was incubated with azide-modified agarose beads (Click Chemistry Tools) according to the manufacturer’s protocol, followed by adding a total of 45 µl reaction solution with 1:1:1 of ascorbic acid (250 mM), CuSO_4_ (10 mM), THPTA (tris-hydroxypropyltriazolylmethylamine; 50 mM). After incubating at 37 °C for 2 h, the beads were washed six times with washing buffer (10 mM Tris-HCl, pH 7, 4 M NaCl, 1 mM EDTA and 0.1% Tween-20) and resuspended in 100 µl release solution containing 200 mM TCEP and 400 mM K_2_CO_3_. The mixture was incubated at 37 °C for 30 min, followed by adding 50 µl of 800 mM iodoacetamide and incubating for another 30 min. The supernatant containing RNA was carefully transferred to a clean tube and purified by RNAClean XP beads (Beckman Coulter) according to the manufacturer’s protocol. The purified RNA was analysed using RT–qPCR as described in the ‘RT–qPCR analysis of mRNAs and pri-, pre- and mature miRNA levels’ section.

#### Pre-miR-155 Chem-CLIP

MDA-MB-231 cells, grown to about 80% confluency in 100-mm-diameter dishes, were treated with DMSO (vehicle; 0.1% (v/v), the final concentration in all compound-treated cells), 100 nM pre-miR-155-Chem-CLIP or 100 nM control probe Ac-Chem-CLIP for 6 h. Competitive Chem-CLIP was performed by pretreating MDA-MB-231 cells with pre-miR-155-amide (0–1 μM as indicated) for 2 h followed by addition of pre-miR-155-Chem-CLIP (100 nM) and incubating the cells for 16 h.

#### JUN Chem-CLIP

MIA PaCa-2 cells, grown to about 90% confluency in 100-mm-diameter dishes, were treated with DMSO (vehicle), JUN-Chem-CLIP or Ctrl-Chem-CLIP at the indicated concentrations for 6 h. Competitive Chem-CLIP was performed by pretreating MIA PaCa-2 cells with JUN-binder at the indicated concentrations for 2 h followed by the addition of JUN-Chem-CLIP and incubating cells for another 5 h. After cross-linking and RNA isolation as described in the ‘General protocol for cellular Chem-CLIP’, the RNA samples were supplemented with a click reaction mixture consisting of disulfide biotin azide (Click Chemistry Tools, 1168, 0.5 µl, 100 mM), CuSO_4_ (1 µl, 10 mM), THPTA (1 µl, 50 mM) and sodium ascorbate (1 µl, 250 mM). The reaction was incubated at 37 °C for 3 h. After the incubation period, 100 µl of Streptavidin beads (Dynabeads MyOne Streptavidin C1 beads; Thermo Fisher Scientific) was added, and the samples were incubated at room temperature for 1 h. The washing and elution steps were identical to those described in the ‘General protocol for cellular Chem-CLIP’ section.

#### MYC Chem-CLIP

HeLa cells, grown to about 80% confluency in 60-mm-diameter dishes, were treated with vehicle, MYC-Chem-CLIP or control probe Ctrl-Chem-CLIP for 5 h. MYC competitive Chem-CLIP was performed by pretreating HeLa cells with MYC-binder at the indicated concentration for 2 h followed by addition of MYC-Chem-CLIP (10 μM) and incubating the cells for 5 h. The remaining steps were performed as described in the ‘General protocol for cellular Chem-CLIP’ section.

### RT–qPCR analysis

#### General Protocol for RT–qPCR analysis

Total RNA was extracted using the Zymo Quick-RNA MiniPrep Kit according to the manufacturer’s protocol. For pri- and pre- miRNAs and mRNAs, RT was performed using 200 ng of RNA with a Qscript cDNA Synthesis Kit (QuantaBio). For mature miRNAs, the RT reaction was performed using 200 ng of RNA with the miScript II RT Kit in a total volume of 20 μl (Qiagen). Subsequent qPCR analysis (see Supplementary Table 2 for primer sequences and Supplementary Figs. 2–7 for primer validation) using Power SYBR Green Master Mix (Life Technologies) or a TaqMan assay and a Applied Biosystems QS5 384-well PCR system (software v.1.3.0). For mature miR-155 levels, TaqMan assays were performed using the ipu-miR-155 Taqman Assay (Thermo Fisher Scientific, 467534). Data were analysed using the ΔΔ*C*_t_ method as described previously^13^.

#### RT–qPCR analysis of mRNA and pri-, pre- and mature miRNA levels

MDA-MB-231 cells or MCF-10a cells were seeded in 12-well plates at around 60% confluency. Cells were treated with compound, LNA-155 miRCURY LNA Power Inhibitor (Qiagen; 5′UUAAUGCUAAUCGUGAUAGGGGU) or vehicle (0.1% (v/v) DMSO, the final concentration in all compound-treated samples) at the indicated concentrations for 48 h in growth medium (unless noted otherwise; Extended Data Fig. 6e). For washout experiments, MDA-MB-231 cells (around 40% confluency) were treated with pre-miR-155-RIBOTAC at the indicated concentration in growth medium for 48 h. After the treatment period, the cells were washed with 1× DPBS and fresh growth medium without the compound was added. Total RNA was then collected from the cells at the indicated timepoints after treatment (12, 24 and 36 h). For competitive experiments with the binder and the RIBOTAC, both compounds were added to the growth medium at the indicated concentrations, applied to the cells and incubated for 48 h. For miRNA profiling, cells were treated with pre-miR-155-RIBOTAC (100 nM) or **LNA-155** (50 nM) in growth medium for 48 h.

CFPAC cells were seeded in 12-well plates at about 50% confluency and treated with compounds at the indicated concentrations for 48 h. HUVECs cells were seeded in 12-well plates at around 60% confluency and treated with compound prepared in growth medium at the indicated concentrations for 48 h. The competitive experiment with the binder and the RIBOTAC was performed by adding both compounds to the growth medium to the desired concentrations, applying the mixture to the cells and incubating for 48 h.

#### RT–qPCR analysis of *JUN* mRNA

MIA PaCa-2 cells, whether unmodified or CRISPR-modified cell lines, were grown to around 40% confluency in 12-well plates and then treated with vehicle (DMSO; 0.1% (v/v), the final concentration in all compound-treated samples) or compound in growth medium at the indicated concentration. The siRNA directed at *JUN* (Dharmacon; L-003268-00-0005) or a scrambled control siRNA (Dharmacon; D-001810-10-05) was transfected with Lipofectamine 3000 (Invitrogen, L3000001) according to the manufacturer’s instructions. After RT as described above, qPCR amplification was performed using the TaqMan gene expression assay (*GAPDH*: Hs03929097_g1, 4331182; *JUN*: Hs00277190_s1, 4331182) and the TaqMan Fast Advanced MasterMix. The same protocol was used for MIA PaCa-2 cells in which RNase L was knocked down by CRISPR and cells for which a control guide RNA was used.

#### RT–qPCR analysis of *MYC* mRNA

HeLa cells and MDA-MB-231 cells (whether unmodified or CRISPR-modified cell lines), grown to around 30% or 50% confluency in 12-well plates, respectively, were treated with vehicle (DMSO; 0.1% (v/v), the final concentration in all compound-treated samples) or compound of interest in growth medium for 24 h. After 24 h, the medium was removed, and the cells were treated with fresh medium containing compound for additional 24 h. For *MYC* siRNA studies, cells were seeded in 12-well plates and 1 nM of *MYC* siRNA (SantaCruz, sc-29226) or scrambled control (SantaCruz, sc-37007) was transfected with Lipofectamine 3000 according to the manufacturer’s recommended protocol. Namalwa (0.4 × 10^6^ cells per ml, 2 ml), HL-60 (0.5 × 10^6^ cells per ml, 2 ml) and Raji (0.7 × 10^6^ cells per ml, 2 ml) cells were treated with vehicle, MYC-RIBOTAC or MYC-Ctr for 48 h. For Namalwa, HL-60 and Raji cells, RNase L mRNA levels were also measured using RT–qPCR. For studies performed in MCF-10a and MDA-MB-231 cells, TaqMan assays were also performed using the TaqMan gene expression assay (*GAPDH*: Hs03929097_g1, 4331182; *MYC*: Hs00153408_m1, 4331182) and the TaqMan Fast Advanced MasterMix.

#### RT–qPCR analysis of *MYC* IRES-luciferase mRNA

HEK293T cells were transfected and treated as described in the ‘MYC luciferase reporter assay’ section except in 12-well instead of 96-well plates. After 48 h, the total RNA was extracted and RT–qPCR was performed as described for mRNAs in the ‘General protocol for RT–qPCR analysis’ section.

### Western blot analysis

#### General protocol for western blotting

After treatment as specified for each target below, total protein was collected using M-PER Extraction Reagent (Thermo Fisher Scientific), and protein concentration was measured using the Pierce Micro BCA Protein Assay Kit (Thermo Fisher Scientific) according to the manufacturer’s protocol. Approximately 20 μg of total protein, except for analysis of SOCS1 protein (which used 40 μg due to its low abundance), was resolved on a 10% SDS–polyacrylamide gel. After transferring to a PVDF membrane and blocking the membrane with 1× tris-buffered saline supplemented with 0.1% (v/v) Tween-20 (TBST; 50 mM Tris-Cl, pH 7.5, 150 mM NaCl and 0.1% (v/v) Tween-20) with 5% (w/v) milk, the membrane was incubated with the primary antibody (see below for each target) in 1× TBST with 5% (w/v) milk at 4 °C for 16 h. The membrane was washed three times with 1× TBST (5 min per wash) at room temperature. The blot was then incubated with IgG horseradish-peroxidase (HRP) secondary antibody conjugate in 1× TBST with 5% milk at room temperature for 1 h. After washing five times with 1× TBST (5 min per wash), the target protein was detected by using SuperSignal West Pico Chemiluminescent Substrate (Pierce Biotechnology) and quantified using ImageJ. The membrane was then stripped (200 mM glycine, pH 2.2, 4 mM SDS, 1% (v/v) Tween-20) at room temperature for 30 min and blotted again with either β-actin or GAPDH for normalization.

#### Western blot analysis of SOCS1

MDA-MB-231 cells were seeded in six-well plates at about 60% confluency and treated with pre-miR-155-RIBOTAC at the indicated concentrations for 48 h. The anti-SOCS1 primary antibody (Cell Signaling Technology (CST), 3950S) was used at 1:2,500 dilution followed by anti-rabbit IgG-HRP secondary antibody conjugate (CST, 7074S) at 1:5,000 dilution. After stripping, β-actin primary antibody (CST, 3700S) was used at 1:5,000 dilution, followed by anti-mouse IgG HRP secondary antibody conjugate (CST, 7076S) at 1:10,000 dilution.

#### Western blot analysis of VHL

HUVECs (around 60% confluency) were seeded in six-well plates and treated with pre-miR-155-RIBOTAC at the indicated concentration for 48 h. The anti-VHL primary antibody (CST, 68547S) was used at 1:1,000 dilution followed by anti-rabbit IgG HRP (CST, 7074S) at 1:5,000 dilution. β-Actin was detected as described above in the ‘Western blot analysis of SOCS1’ section.

#### Western blot analysis of JUN and RNase L

MIA PaCa-2 cells were seeded in six-well plates and treated with vehicle (0.1% (v/v) DMSO; the final concentration in all compound-treated samples) or compound at the indicated concentrations in growth medium for 72 h once they reached around 40% confluency. The anti-JUN primary antibody (CST, 9165S) or anti-RNase L antibody (CST, D4B4J) was used at 1:1,000 dilution followed by anti-rabbit IgG HRP (CST, 7074S) at 1:10,000 dilution. β-Actin was detected as described above in the ‘Western blot analysis of SOCS1’ section.

#### Western blot analysis of MYC

HeLa cells in six-well plates (around 30% confluency) or MDA-MB-231 cells (about 60% confluency) were seeded in six-well plates and treated with vehicle or compound at the indicated concentrations in growth medium for 24 h. The compound-containing growth medium was removed and replaced with fresh growth medium containing compound. The cells were incubated for an additional 24 h at which point total protein was collected as described above. Namalwa cells (0.4 × 10^6^ cells per ml, 10 ml) were treated with vehicle, MYC-RIBOTAC or MYC-Ctr for 48 h (single dose), and then total protein was collected. The anti-MYC antibody (CST, 5605S) was used at 1:1,000 dilution followed by anti-rabbit IgG HRP (CST, 7074S) at 1:5,000 dilution. The anti-GAPDH primary antibody (CST, 51332S) was used at 1:3,000 dilution followed by direct imaging (no secondary antibody needed as this antibody is an HRP conjugate). The membrane was not stripped and was directly blotted with anti-GAPDH antibodies for normalization.

#### Western blot analysis of BRD4

The same procedure was followed as described in the ‘Western blot analysis of MYC’ section except that the primary antibody was anti-BRD4 (Cell Signaling Technologies, 13440S) at 1:1,000 dilution. The effect of MZ1 on BRD4 and MYC protein levels was validated and shown in Supplementary Fig. 8.

### SOCS1 luciferase reporter assay (pre-miR-155)

HEK293T cells in 60-mm-diameter dishes (around 80% confluency) were co-transfected with 4 µg of a plasmid encoding SOCS1 fused to luciferase (Genecopoeia, HmiT021399-MT06) and 1 µg of the plasmid expressing wild-type pre-miR-155 using jetPRIME (Polypuls, 101000027) according to the manufacturer’s recommended protocol. The cells were seeded to 96-well plates (7,000 cells per well) after transfection. After incubating for 12 h, the cells were treated with DMSO (vehicle, 0.1% (v/v)) or pre-miR-155-RIBOTAC. A dose–response was measured by treating pre-miR-155-RIBOTAC at 0, 10 and 100 nM for 48 h and a time course was measured by treating pre-miR-155-RIBOTAC (100 nM) for 6, 24, 48 and 72 h. The Dual-Glo Luciferase Assay System (Promega, E2920) was used to measure luciferase activity, according to the manufacturer’s instructions.

### MYC luciferase reporter assay

The *MYC* IRES luciferase plasmid was custom-synthesized by GenScript (U6617GC310) by inserting the *MYC* IRES sequence between the two Click Beetle luciferases (*CBR* and *CBG*) within the pcDNA5 vector. HEK293T cells in 60-mm-diameter dishes (around 80% confluency) were co-transfected with 2 µg of a plasmid and 500 ng of a plasmid expressing Renilla luciferase (normalization) using jetPRIME (Polyplus, 101000027) according to the manufacturer’s recommended protocol. Control experiments were performed by co-transfecting 2 µg of a plasmid encoding luciferase lacking the IRES (GenScript) and 500 ng of a plasmid expressing *Renilla* luciferase (for normalization). The cells were trypsinized and seeded into 96-well plates (7,000 cells per well). After incubating for 12 h, the cells were treated with vehicle or MYC-RIBOTAC (10 µM) for the indicated time. Click Beetle (which uses the same substrate as firefly luciferase) and *Renilla* luciferase activities were measured using the Dual-Glo Luciferase Assay System (Promega, E2920) according to the manufacturer’s instructions.

To construct a mutant *MYC* IRES plasmid in which the small-molecule-binding motif was mutated into base pairs, the Q5 Site-Directed Mutagenesis Kit (NEB, E0554S) was used according to the manufacturer’s recommendations. The primers used to create the mutant were: 5′CGCCTCTGGC**GA**AGCCCTCCCG and 5′AAGCCCCCTATTCGCTCC (IDT), where the bold nucleotide indicates the point mutation. The mutagenesis product was transformed into chemically competent *Escherichia coli* (included in the kit) and selected using ampicillin-agar plates. The mutant plasmid sequence was confirmed by Sanger sequencing, and the plasmid was tested in HeLa cells according to the same procedure as described above.

### Methylcellulose colony assays

Namalwa Burkitt lymphoma cells (1,000 cells per ml, 3 ml) were treated with MYC-Ctr (10 µM) or MYC-RIBOTAC (10 µM) for 48 h (single dose). The cells were then collected by centrifugation for 5 min at 1,000 rpm, and 1 × 10^3^ viable cells were resuspended in 400 µl of IMDM medium containing 2% (v/v) FBS. The cells were then added to 4 ml of Methocult (STEMCELL Technology, H4230), and 1.1 ml was carefully spread into a SmartDish (STEMCELL Technology, 27370). Cells were incubated for 7–10 days, and colonies were counted manually.

### Cell cycle analysis

Namalwa (0.4 × 10^6^ cells per ml, 2 ml), HL-60 (0.5 × 10^6^ cells per ml, 2 ml) and Raji (0.7 × 10^6^ cells per ml, 2 ml) cells were treated with vehicle, MYC-RIBOTAC (10 µM) or MYC-Ctr (10 µM) for 48 h (single dose). To assess the percentage of cells at each stage of the cell cycle, 1 × 10^6^ cells were fixed dropwise with 70% (v/v) ethanol at room temperature, and then stored for 24 h at −20 °C. Fixed cells were washed twice with 1× PBS, and then treated with 50 µl of 100 µg ml^−1^ RNase A (Sigma-Aldrich, R-6148) for 5 min room temperature. The cells were then stained with 200 µl of 50 µg ml^−1^ propidium iodide; Sigma-Aldrich, P4170) prepared in 1× PBS. The stained cells were analysed for the percentage in the G1, S and G2 phases of the cell cycle in a BD LSRII (BD Biosciences) flow cytometer. Cell doublets were excluded from analysis.

### Drug metabolism and pharmacokinetic studies (pre-miR-155)

Female C57Bl/6 mice (*n* = 3) were treated once with either pre-miR-155-RIBOTAC or pre-miR-155-amide-binder at 1 mg per kg (10:10:80 of DMSO:Tween-80:H_2_O) by i.p. injection. The plasma was then collected at 0, 15, 30, 60, 120, 240, 360, 480 and 1,440 min to determine the concentration in plasma.

### In vivo studies to assess lung colonization of breast cancer cells (pre-miR-155)

Mice were housed in individually ventilated (IVC), JAG 75 cages with micro-isolator lids. HEPA filtered air was supplied into each cage at a rate of 60 air exchanges per h. The dark–light cycle was set for 08:00 (on)–20:00 (off). The temperature was maintained at 72 ± 2 °F (22.2 ± 1.1 °C). Humidity was maintained at 30–70%. Female NOD/SCID mice (*n* = 8 per group; 5–7 weeks; The Jackson Laboratory) were used for breast cancer studies as described previously^56^, and all studies were approved by The Scripps Research Institute IACUC (protocol no. 16-025). The maximal tumour size allowable under this protocol is 500 mm^3^ or 2 cm in diameter.

In brief, mice were intravenously injected (tail vein) with MDA-MB-231-Luc cells (0.8 × 10^6^ cells per mouse). Compound treatment began 5 days later as determined by luciferase activity, where the averaged luciferase signal in the lung tissue is ten times higher than background measured from neighbouring tissues. Luciferase activity was measured by i.v. injection of luciferin (150 mg per kg) every other day as measured by LagoX (Spectral Instruments). Mice were split into two groups: vehicle group (10:10:80 of DMSO:Tween-80:H_2_O) or 1 mg per kg of either pre-miR-155-RIBOTAC or pre-miR-155-amide in the same formulation. Compound or vehicle was delivered by i.p. injection every other day.

After 30 days, mice were euthanized by affixiation with CO_2_ in accordance with guidelines provided by the American Veterinarian Medical Association. For each group, the lungs from five mice were perfused with 1× PBS, collected and immediately fixed in Bouin’s solution (Sigma-Aldrich, HT10132-1L) to image nodules. After imaging, the lungs were sectioned and stained with H&E staining or analysed using FISH to assess pre-miR-155 levels. The FISH probe and qPCR primers are specific to human mature and pre-miR-155; mouse pre-miR-155 has a different secondary structure lacking the binding site for pre-miR-155-RIBOTAC. Human and mouse SOCS1 protein cannot be distinguished by available antibodies; therefore, SOCS1 protein levels were not determined. For the remaining three mice from each group, the lungs were collected and snap-frozen for extracting total RNA to measure mature-miR-155 levels by RT–qPCR.

To image lung nodules, lungs were fixed in 60 ml of Bouin’s solution for 24 h followed by briefly rinsing in 10% neutral buffered formalin solution (Harleco). After counting nodules, the lungs were washed with 60 ml of 10% (v/v) formalin solution four times (8 h per wash with gentle shaking at room temperature). Paraffin-embedded sections were generated by the Histology Core at The Scripps Research Institute using a Sakura Tissue-Tek VIP5 paraffin processor. H&E staining was performed on the Leica ST5010 Auto-Stainer XL at The Scripps Research Institute’s Histology Core.

For FISH analysis to assess mature miR-155 levels, the sections were incubated at 60 °C for 15 min followed by deparaffination with xylene. The sections were rinsed once in each of the following solutions: 95%, 80%, 50% and 0% ethanol in water. The sections were then treated with 20 µg ml^−1^ proteinase K in 50 mM Tris, pH 7.5, for 10–20 min at 37 °C, followed by rinsing in water five times. The sections were then treated with ice-cold 20% (v/v) acetic acid for 20 s to permeabilize cells, followed by rinsing with Nanopure water three times. The sections were dehydrated by rinsing in 100% ethanol. The sections were then prepared for FISH analysis by rinsing the sections in 40% formamide/2× SSC (0.3 M sodium chloride and 0.03 M sodium citrate, pH 7.0) buffer for 5 min. The slides were then incubated with 40% formamide/2× SSC buffer containing 0.2 µM FITC-labelled DNA probe (5′-AACCCCTATCACGATTAGCATTAA), 2 µg ml^−1^ BSA, 10 nM tRNA (Roche, 10109517001) in a humidified chamber at 37 °C for 16 h. The slides were then rinsed three times with 2× SSC, and once with 1× DPBS, followed by addition of an anti-FITC antibody (Abcam, 6656; 1:500 dilution in 1×TBST buffer containing 1% (v/v) goat serum (Sigma-Aldrich)). After incubation at room temperature for 2 h, the slides were rinsed three times with 1× DPBS and visualized using a DAB Substrate Kit (Abcam, 64238) according to the manufacturer’s protocol. Images were acquired using a bright-field microscope (Leica DM800) and quantified using ImageJ.

The lungs from the other three mice were frozen at −80 °C and homogenized using tissue plastic homogenizing probes (Omni International, 10062-782). The RNA extraction and RT–qPCR analysis were performed the same as described above in the ‘RT–qPCR analysis of mRNAs and pri-, pre- and mature miRNA levels’ section.

### Reporting summary

Further information on research design is available in the Nature Portfolio Reporting Summary linked to this article.

## Online content

Any methods, additional references, Nature Portfolio reporting summaries, source data, extended data, supplementary information, acknowledgements, peer review information; details of author contributions and competing interests; and statements of data and code availability are available at 10.1038/s41586-023-06091-8.

## Supplementary information

Supplementary Information Supplementary Tables 1 and 2; Supplementary Figures 1–9; Supplementary Methods; and synthetic methods and characterizations.

Reporting Summary

## Data Availability

All data and materials are available from the corresponding author on reasonable request. The data for global proteomics and RNA-seq studies are available at Mendeley Data (10.17632/xgr83xy8pm.1). The sequencing data for plasmids used in this study are available at Mendeley Data (10.17632/9zgvv67j7s.1). The raw sequencing data have been deposited at the Sequence Read Archive under BioProject ID PRJNA914317. The R-Bind database^17^ is publicly accessible (https://rbind.chem.duke.edu). The Inforna database is available online (https://rnainforna.com). Access is freely granted for academic users after completion of a software license agreement (https://disney.scripps.ufl.edu/wp-content/uploads/2020/05/software-licensing-agreement-1.pdf). Source data are provided with this paper.

## Source data

Source Data Fig. 2
Source Data Fig. 3
Source Data Fig. 4
Source Data Fig. 5
Source Data Extended Data Fig. 1
Source Data Extended Data Fig. 3
Source Data Extended Data Fig. 6
Source Data Extended Data Fig. 7
Source Data Extended Data Fig. 8
Source Data Extended Data Fig. 9
Source Data Extended Data Fig. 10
Source Data Extended Data Fig. 11

## Code availability

No unique code was used in the described data analyses.
