# Supplementary material for: Programming inactive RNA-binding small molecules into bioactive degraders
Source: Nature. 2023 May 24;618(7963):169–79. doi: 10.1038/s41586-023-06091-8 (PMC10232370; doi:10.1038/s41586-023-06091-8)
Supplement: Supplementary file 1 — Supplementary Tables 1 and 2; Supplementary Figures 1–9; Supplementary Methods; and synthetic methods and characterizations. [file 41586_2023_6091_MOESM1_ESM.pdf]

nature portfolio

https://doi.org/10.1038/s41586-023-06091-8

Supplementary information

Programming inactive RNA-binding small molecules into bioactive degraders

In the format provided by the authors and unedited

nature portfolio

https://doi.org/10.1038/s41586-023-06091-8

Supplementary information

Programming inactive RNA-binding small molecules into bioactive degraders

In the format provided by the authors and unedited

Supplementary Table 1. Hit compounds from 2DCS studies and the unique sequences and secondary structures of selected RNAs having no previously known small molecule binder.

Compound Structure	Compound	Scaffold	Number of motifs bound ($Z_{obs} > 8$)	Motifs unique to compound
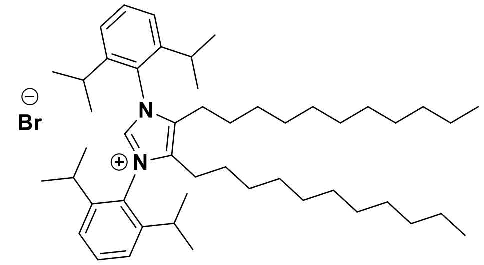	**C1**	AZS	58	5’UACGU/3’AAUAA<br>5’UCUAU/3’AAUGA<br>5’UAAUU/3’ACACA<br>5’UCUGU/3’AAUAA<br>5’UUCGU/3’AUAAA<br>5’UUCGU/3’ACUAA<br>5’UCCG/AAUU<br>5’UUCUU/3’ACUCA
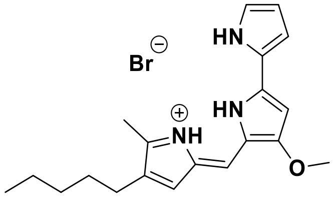	**C2**	BPS	238	5’UUAU/3’ACAA<br>5’UUAG/3’ACCC<br>5’UAUAU/3’ACAGA<br>5’UAAA/3’ACGU
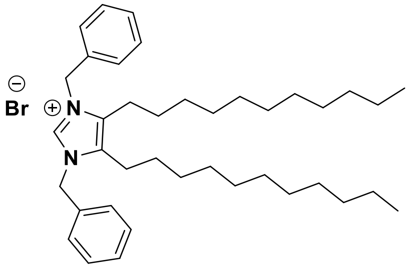	**C3**	AZS	221	5’UAGAU/3’ACACA<br>5’UAUU/3’ACCA<br>5’UCAU/3’ACCA<br>5’UUCAU/3’ACACA<br>5’UUGUU/3’ACCCA<br>5’UAUAU/3’AGGGA<br>5’UCUAU/3’AUAAA<br>5’UCGUU/3’ACCCA
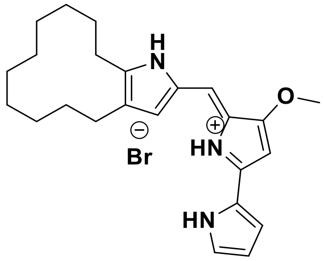	**C4**	AZS	137	5’UUUCU/3’AUUAA<br>5’UAUU/3’ACCG
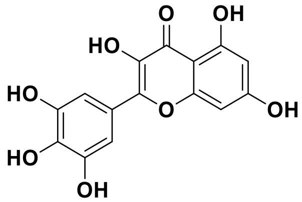	**C5**	CHR	121	No unique motifs
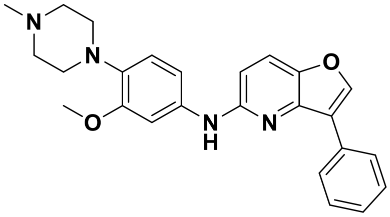	**C6**	3PPA	69	5’UUUCU/3’AUUAA<br>5’UAUU/3’ACCG

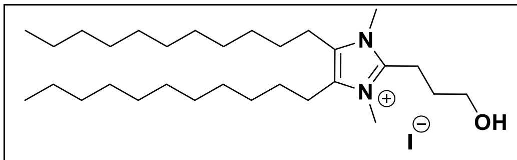	**C7**	AZS	162	No unique motifs
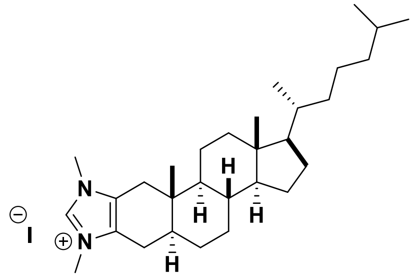	**C8**	AZS	433	No unique motifs
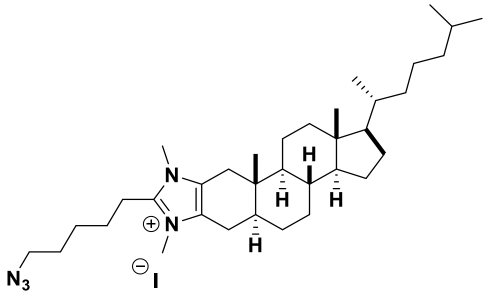	**C9**	AZS	411	5’UAAU/3’AAAG<br>5’UUGCU/3’AUAAA<br>5’UAUGU/3’AGAAA<br>5’UAGUU/3’AAAUA<br>5’UCAGU/3’AAAAA<br>5’UAAU/3’GAGA<br>5UCCU/3’ACUA<br>5’UAACU/3’AACUA<br>5’UCUAU/3’AAGCA<br>5’UAGGU/3’AAAAA<br>5’CACU/3’GAAA<br>5’UCAUU/3’AACUA<br>5’UAUGU/3’AAGAA<br>5’UCAAU/3’AAUAA<br>5’UAAU/3’GCGA
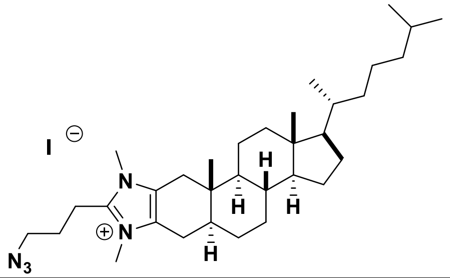	**C10**	AZS	332	5’UAAUU/3’AAACA<br>5’UGAU/3’GAAA<br>5’UAAU/3’AACG<br>5’UGUC/3’AAUG
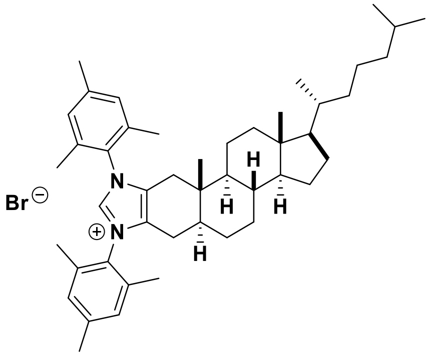	**C11**	AZS	306	5’UCUUU/3’AAACA<br>5’UCCU/3’AUAA

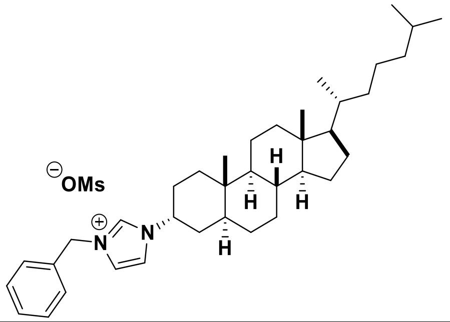	**C12**	AZS	176	No unique motifs
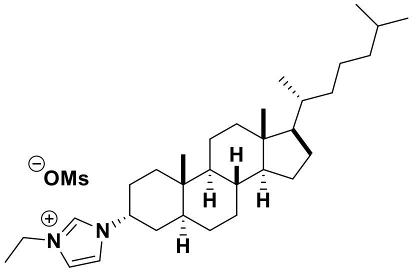	**C13**	AZS	340	5’UGAUU/3’AAGUA<br>5’UUCU/3’ACCA
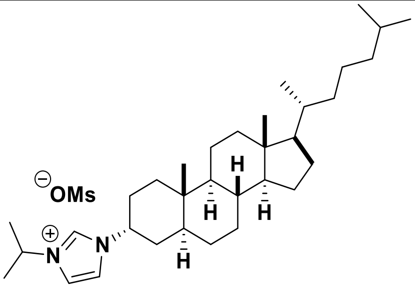	**C14**	AZS	378	5’UCGA/3’AAGU<br>5’UCGUU/3’AAUCA<br>5’CGGU/3’GAAA<br>5’UGAG/3’AACU<br>5’UGUCU/3’AACUA<br>5’CAGU/3’GCAA<br>5’CUGU/3’GCAA
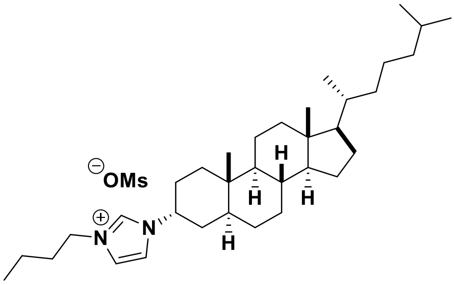	**C15**	AZS	248	5’UAGU/3’GCGA
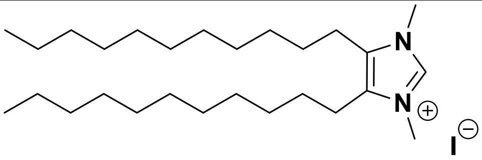	**C16**	AZS	253	5’UCUU/3’ACCA
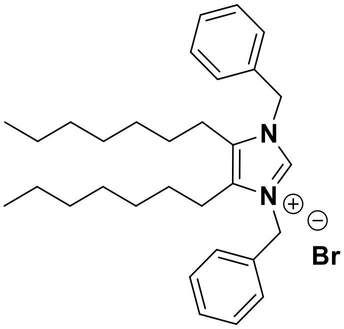	**C17**	AZS	390	5’UAAGU/3’AAGGA<br>5’UGGU/3’AGAG<br>5’UGUG/3’AACU<br>5’UCGCU/3’AUAAA<br>5’UAGU/3’ACGA<br>5’UAAGU/3’AACGA<br>5’UGAAU/3’AGAGA<br>5’UGUU/3’AGCG

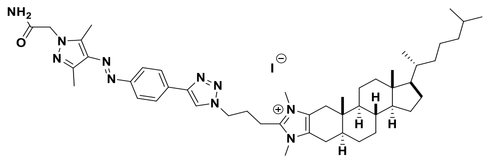	**C18**	AZS	334	5'UAAU/3'AGCG<br>5'UAGU/3'GCAA<br>5'UACAU/3'AAGGA
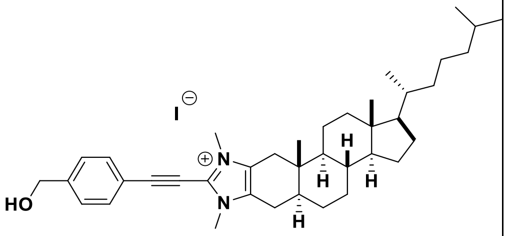	**C19**	AZS	473	5'UGGGU/3'AACGA<br>5'UGAU/3'AGAA<br>5'UAGU/3'AGAA<br>5'UCGAU/3'AAUGA<br>5'CAAU/3'GCCA<br>5'UCGCU/3'AAUCA<br>5'UGCUU/3'AAGUA<br>5'UGGU/3'AAGA<br>5'CGU/3'GAG<br>5'UACGU/3'ACAGA<br>5'UGCG/3'AACU<br>5'UGCAU/3'AAUCA<br>5'UGAGU/3'AGGAA<br>5'UCGCU/3'AAUAA<br>5'UCGGU/3'AAGAA<br>5'UGUAU/3'AGACA<br>5'UGAUU/3'AGACA<br>5'UGGGU3/AAGGA<br>5'UGAU/3'AGAA<br>5'UGGU/3'AAGG<br>5'UGGU/3'AAGA
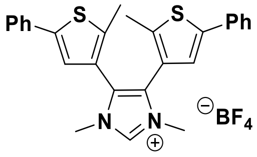	**C20**	AZS	305	5'UGGC/3'AAGG<br>5'UGGAU/3'AGGAA<br>5'UGUUU/3'AGUCA<br>5'UACGU/3'AAGGA<br>5'UU/3'AGCA

AZS, azolium salts; BPS, bipyrrole pyrrolium salts; Chr, chromone; 3PPA, 3-phenylfuro[3,2-b]pyridine-5-amines.

Supplementary Table 2: Sequences of oligonucleotides and primers used in these studies.

Oligonucleotide	Sequence 5′ to 3′	Experiment	Supplier
RNU6	ACACGCAAATTCGTGAAGCGTTC	RT-qPCR	IDT
miR-155-5p Fwd.	TTAATGCTAATCGTGATAGGGGTT	RT-qPCR	IDT
miR-4273-5p Fwd.	GTGTTCTCTGATGGACAG	RT-qPCR	IDT
miR-1226-3p Fwd.	TCACCAGCCCTGTGTTCCCTAG	RT-qPCR	IDT
miR-101-1-3p Fwd.	TACAGTACTGTGATAACTGAA	RT-qPCR	IDT
miR-4435-1-5p Fwd.	ATGGCCAGAGCTCACACAGAGG	RT-qPCR	IDT
miR-196a-5p Fwd.	TAGGTAGTTTCATGTTGTTGGG	RT-qPCR	IDT
miR-3945-5p Fwd.	AGGGCATAGGAGAGGGTTGATAT	RT-qPCR	IDT
miR-4640-3p Fwd.	CACCCCCTGTTTCCTGGCCCAC	RT-qPCR	IDT
miR-3168-5p Fwd.	GAGTTCTACAGTCAGAC	RT-qPCR	IDT
miR-4700-5p Fwd.	TCTGGGGATGAGGACAGTGTGT	RT-qPCR	IDT
let-7g-5p Fwd.	TGAGGTAGTAGTTTGTACAGTT	RT-qPCR	IDT
miR-18a-5p Fwd.	TAAGGTGCATCTAGTGCAGATAG	RT-qPCR	IDT
miR-363-3p Fwd.	AATTGCACGGTATCCATCTGTA	RT-qPCR	IDT
Universal Reverse	GAATCGAGCACCAGTTACGC	RT-qPCR	IDT
pri-miR-155 Fwd.	GTGTATGATGCCTGTTACTAGCA	RT-qPCR	IDT
pri-miR-155 Rev.	GCCTGAAGTCTAAGTTTATCCAGC	RT-qPCR	IDT
pre-miR-155 Fwd.	TGCTAATCGTGATAGGGGTTTT	RT-qPCR	IDT
pre-miR-155 Rev.	TGCTAATATGTAGGAGTCAGTTGGA	RT-qPCR	IDT
pre-miR-155 binding site mutation Fwd.	TGCTAATCGTGATAGGGGTTT	RT-qPCR	IDT
pre-miR-155 binding site mutation Rev.	TGCTAATATGATAGGAGTCAGTTGGA	RT-qPCR	IDT
pre-miR-155 cleavage site mutation Fwd.	TGCTAATCGTGATAGGGGTTAG	RT-qPCR	IDT
pre-miR-155 cleavage site mutation Rev.	TGCTAATATGTAGGAGTCAGTTGGA	RT-qPCR	IDT
pre-miR-4435 Fwd.	ATGGCCAGAGCTCACACAGA	RT-qPCR	IDT
pre-miR-4435 Rev	CACACTGCAGGTGAAGTGCACT	RT-qPCR	IDT
pre-miR-3168 Fwd.	GAGTTCTACAGTCAGACAGCCTG	RT-qPCR	IDT
pre-miR-3168 Rev.	GCAAGAAGTGAAGATGAGCCTCC	RT-qPCR	IDT
pre-miR-4640 Fwd.	TGGGCCAGGGAGCAGCT	RT-qPCR	IDT
pre-miR-4640 Rev	GTGGGTGGGATGGAGTCCA	RT-qPCR	IDT
pre-miR-4700 Fwd.	TCTGGGGATGAGGACAGTGTGT	RT-qPCR	IDT
pre-miR-4700 Rev.	GGGTGAGGAGTCAGTCCTGTGAATTT	RT-qPCR	IDT
pre-miR-1226 Fwd.	GTGAGGGCATGCAGGCCTG	RT-qPCR	IDT
pre-miR-1226 Rev.	TGGTGAGGCCACCCTTTTGG	RT-qPCR	IDT
MYC-Luciferase Fwd.	GTGATCTACGGCCCAGAAC	RT-qPCR	IDT
MYC-Luciferase Rev.	CTTTGGGCCAACAGCACA	RT-qPCR	IDT
pre-miR-155 Template	CTGTTAATGCTAATCGTGATAGGGGTTTTTGCCTC CAACTGACTCCTACATATTAGCATTAACAG	In vitro cleavage	IDT
pre-miR-155 +U49 Mutant Template	CTGTTAATGCTAATCGTGATAGGGGTTTTTGCCTC CAACTGACTCCTA**T**CATATTAGCATTAACAG	In vitro cleavage	IDT
pre-miR-155 U27 AG Mutant Template	CTGTTAATGCTAATCGTGATAGGGGTT**AG**TTGCCT CCAACTGACTCCTACATATTAGCATTAACAG	In vitro cleavage	IDT

pre-miR-155 T7 Fwd.	GGCCGGATCCTAATACGACTCACTATAGGCTGTTAATGCTAATCGTGATAGGG	In vitro cleavage	IDT
pre-miR-155 +U49 Rev.	CTGTTAATGCTAATATG**A**TAGGAGTCAG	In vitro cleavage	IDT
pre-miR-155 Rev.	CTGTTAATGCTAATATGTAGGAGTCAG	In vitro cleavage	IDT
Cy5-2DCS model with two A bulges	GGGUUUAAUAGAUACGAAAGUACUAAUUGGAUCC	MST Binding	Dharmacon
Cy5-2DCS model with 5'GAU/A_C	GGGUUUAAUAGAUACGAAAGUAUCUAAUUGGAUCC	MST Binding	Dharmacon
Cy5-2DCS model with 5'U_A/UAA	GGGUUUAAUUAGAUACGAAAGUACUAAUUGGAUCC	MST Binding	Dharmacon
Cy5-2DCS model fully base paired	GGGUUUAAUUAGAUACGAAAGUAUCUAAUUGGAUCC	MST Binding	Dharmacon
Cy5-5'GAU/3'C_A mimic	Cy5-GUAAUGAUUACGAAAGUAAC AUUGC	MST Binding	Dharmacon
Cy5-5'GAU/3'CUA mimic	Cy5-GUAAUGAUUACGAAAGUAA**U** CAUUGC	MST Binding	Dharmacon
c-Myc Fwd.	TGAGGAGACACCGCCCAC	RT-qPCR	IDT
c-Myc Rev.	CAACATCGATTTCTTCCTCATCTTC	RT-qPCR	IDT
18S Fwd.	GTAACCCGTTGAACCCCATT	RT-qPCR	IDT
18S Rev.	CCATCCAATCGGTAGTAGCG	RT-qPCR	IDT
GAPDH Fwd.	GTTCGACAGTCAGCCGCATC	RT-qPCR	IDT
GAPDH Rev.	GGAATTTGCCATGGGTGGA	RT-qPCR	IDT
Control Screen Template	GGGAGAGGGTTTAATTACGAAAGTATTGGATCCGCAAGG	Screening	IDT
3×3 ILL	GGGAGAGGGTTTAAT***NNN***TACGAAAGT***NNN***ATTGGATCCGCAAGG	Screening	IDT
T7 Forward 3×3 ILL	GGCCGGATCCTAATACGACTCACTATAGGGAGAGGGTTTAAT	Screening PCR	IDT
Reverse 3×3 ILL	CCTTGCGGATCCAAT	Screening PCR	IDT
Bolded nucleotides are mutations except ***N*** which indicates any nucleotide A, T, G, or C.			

Fig. 2g

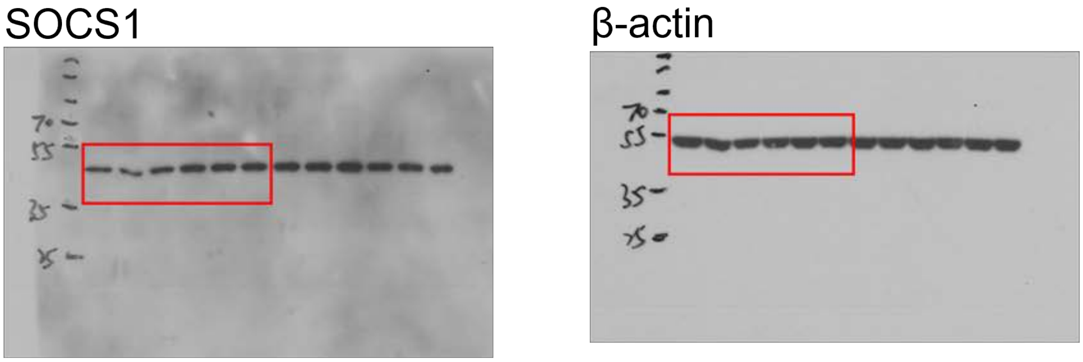

Fig. 4d

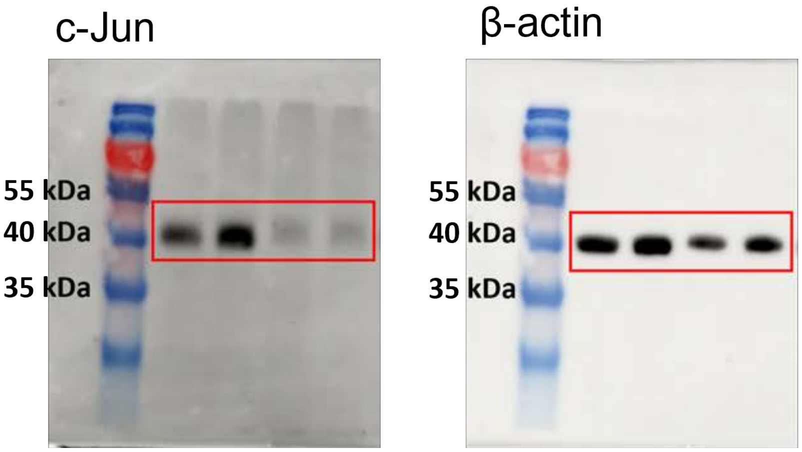

Fig. 5d

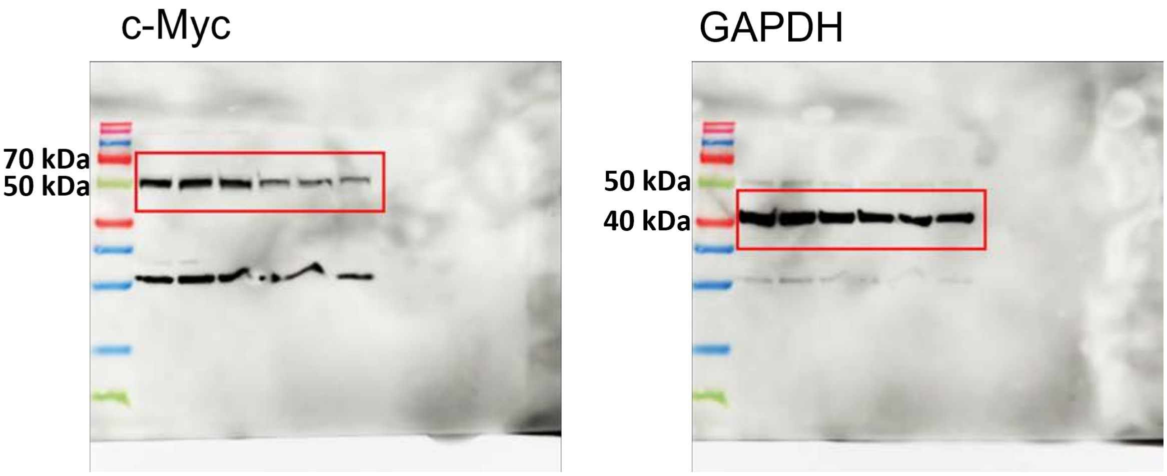

Fig. 5j

c-Myc β-actin

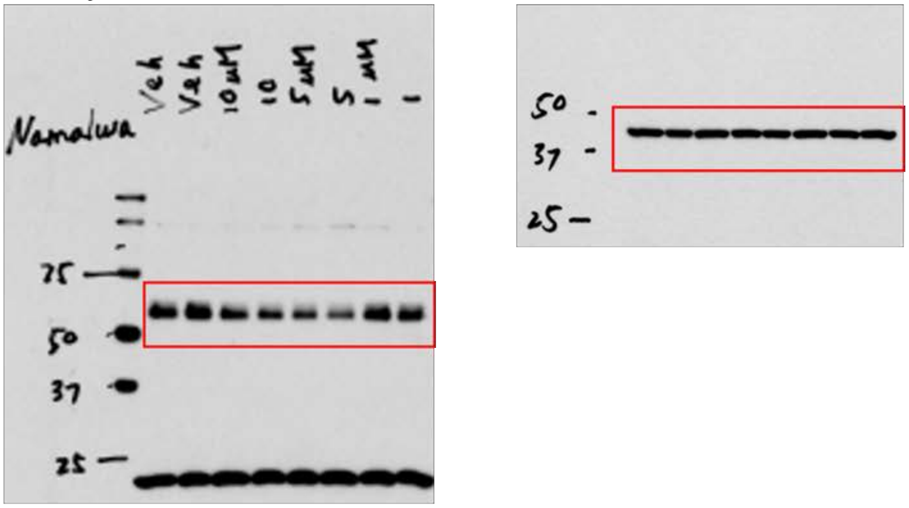

Ext. Data. Fig. 6a (left two) and 6b (right three)

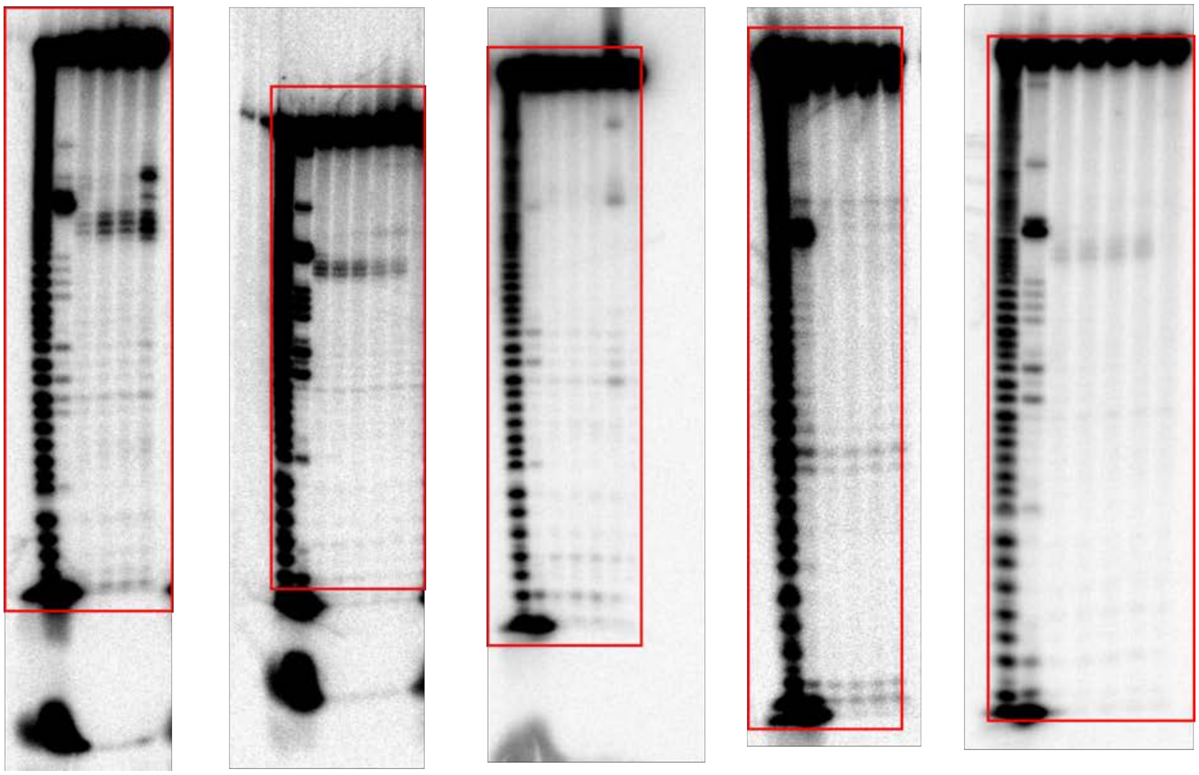

Ext. Data Fig. 8h

VHL

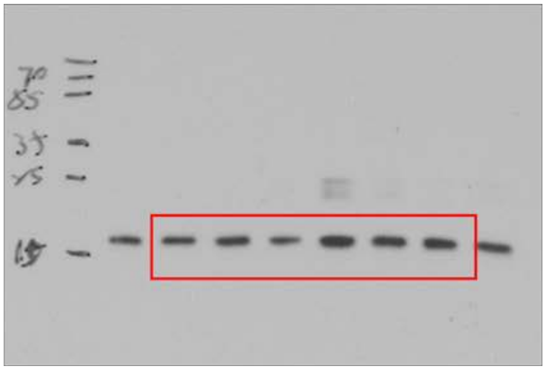

β-actin

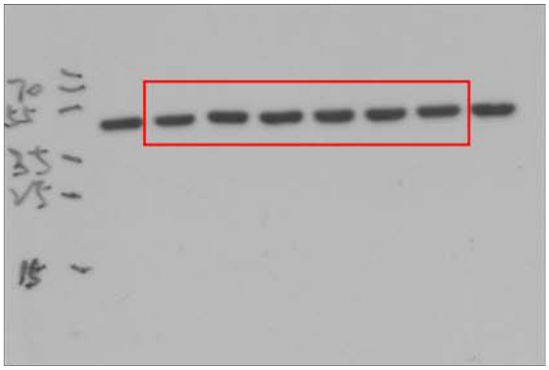

Ext. Data Fig. 9e

c-Jun

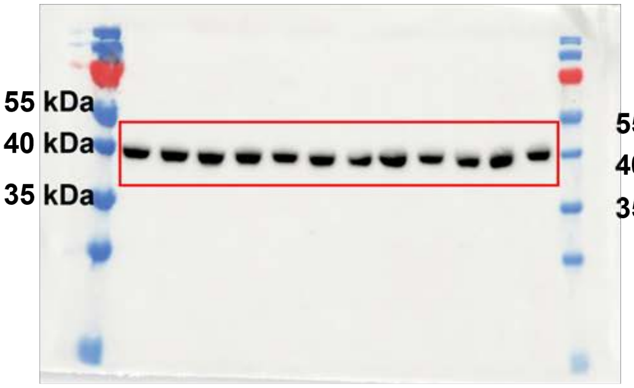

β-actin

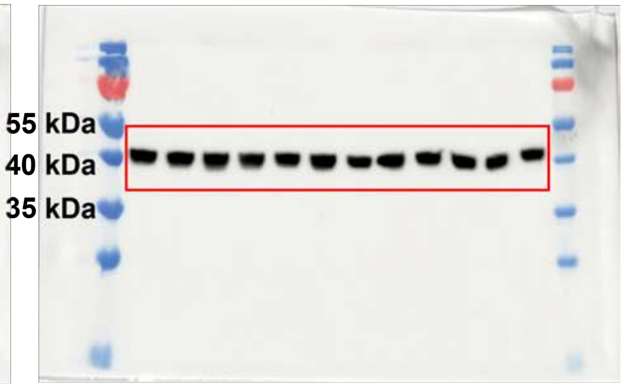

Ext. Data Fig. 10c

c-Myc

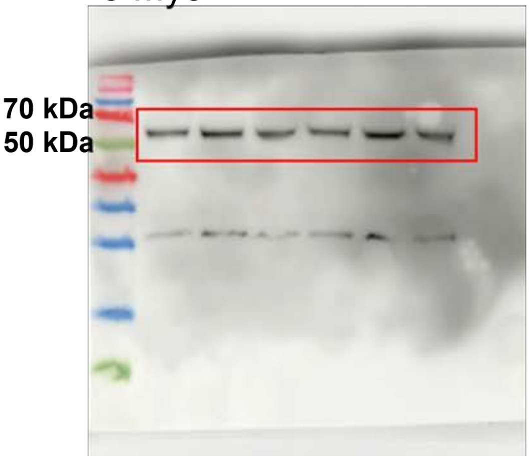

GAPDH

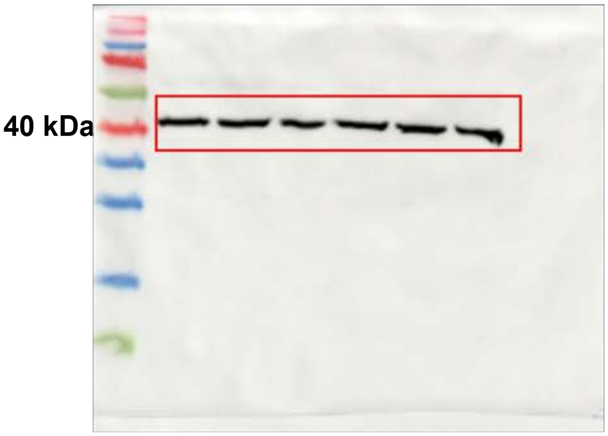

Ext. Data Fig. 10f (left panel)

c-Myc

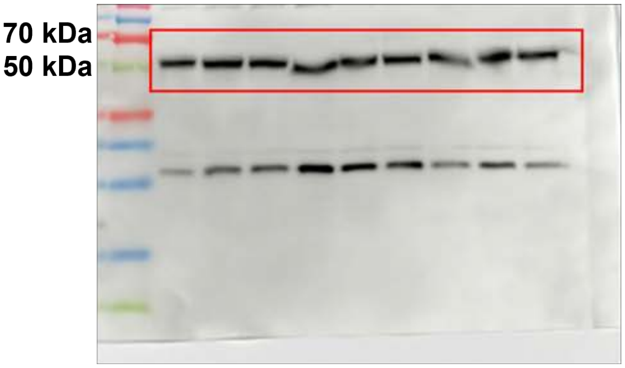

GAPDH

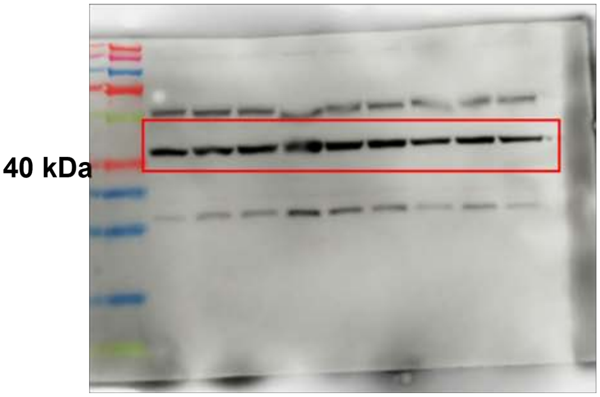

Ext. Data Fig. 10f (right panel)

c-Myc

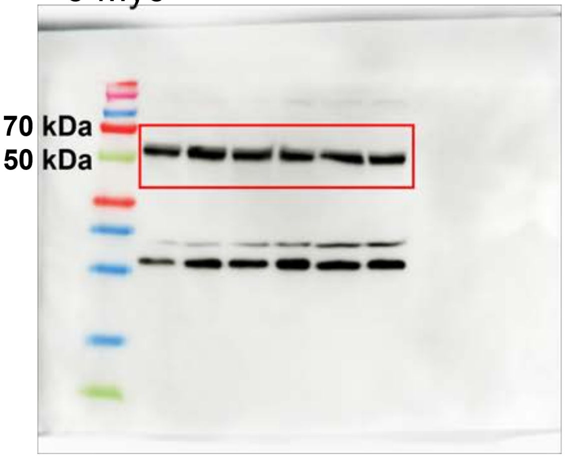

GAPDH

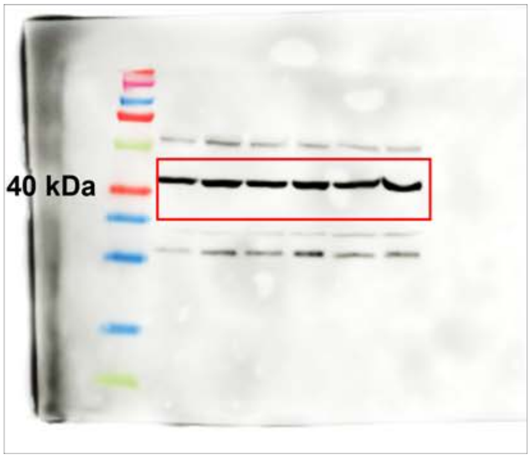

Ext. Data Fig. 10i

c-Myc

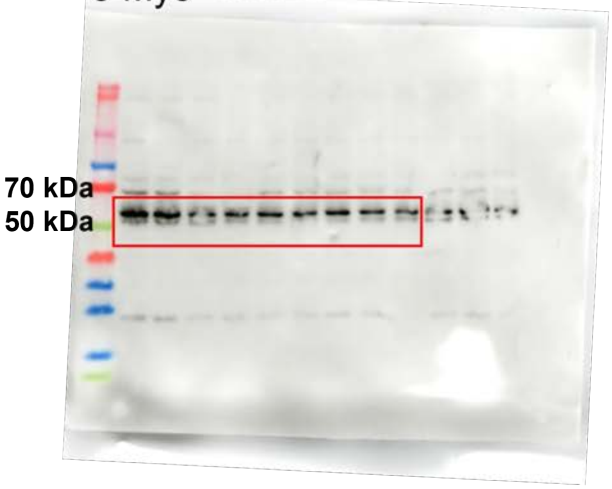

GAPDH

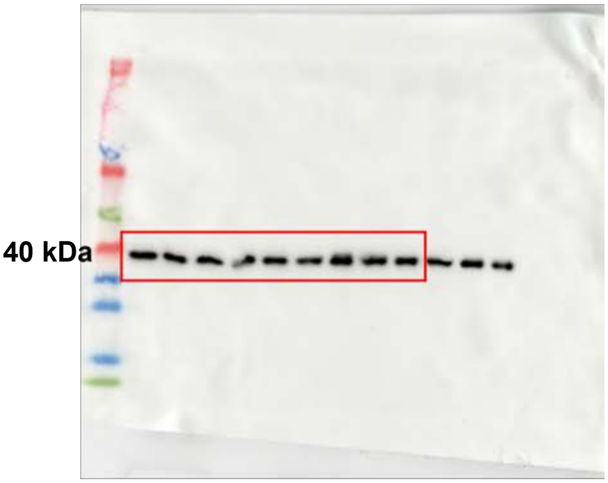

Supplementary Figure 5

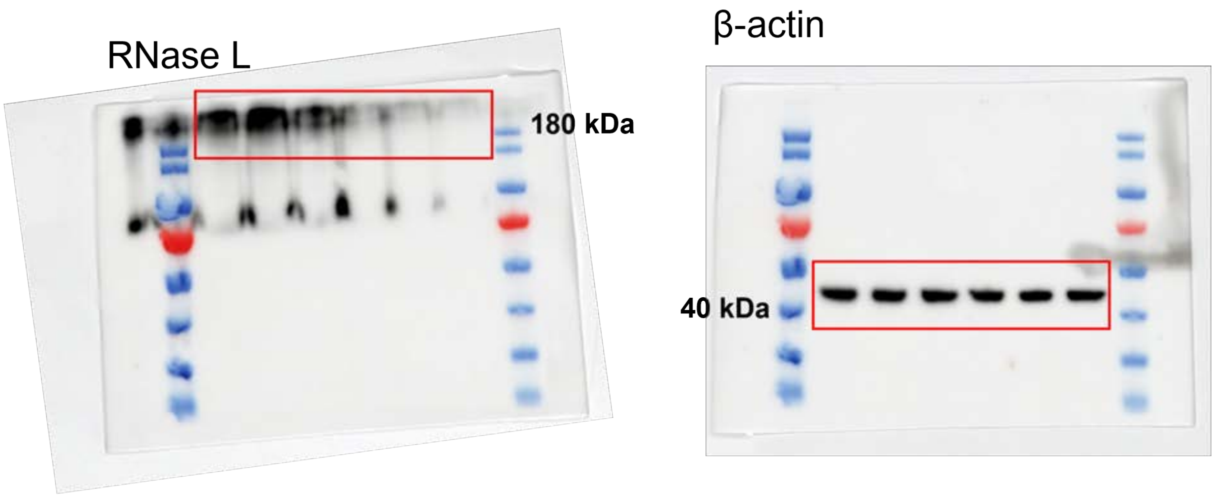

Supplementary Figure 8

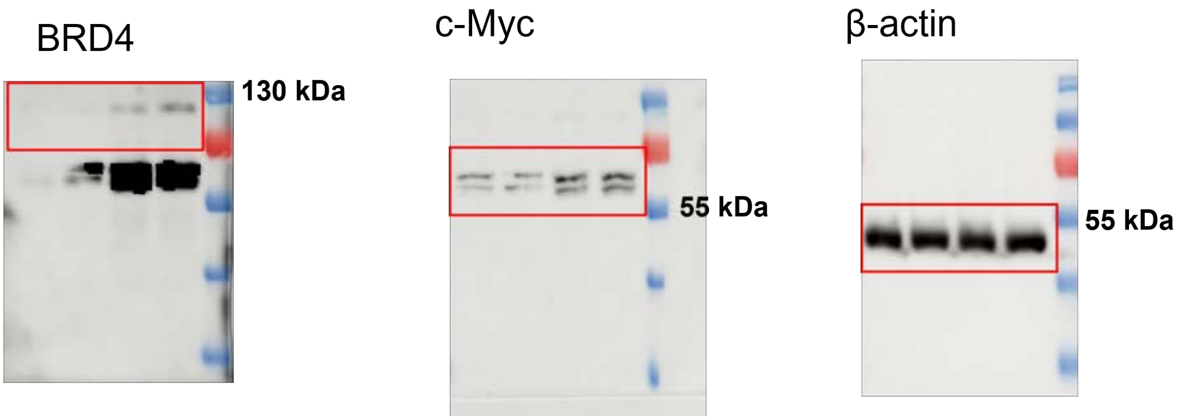

Supplementary Figure 1 | Uncropped images of gel electrophoresis.

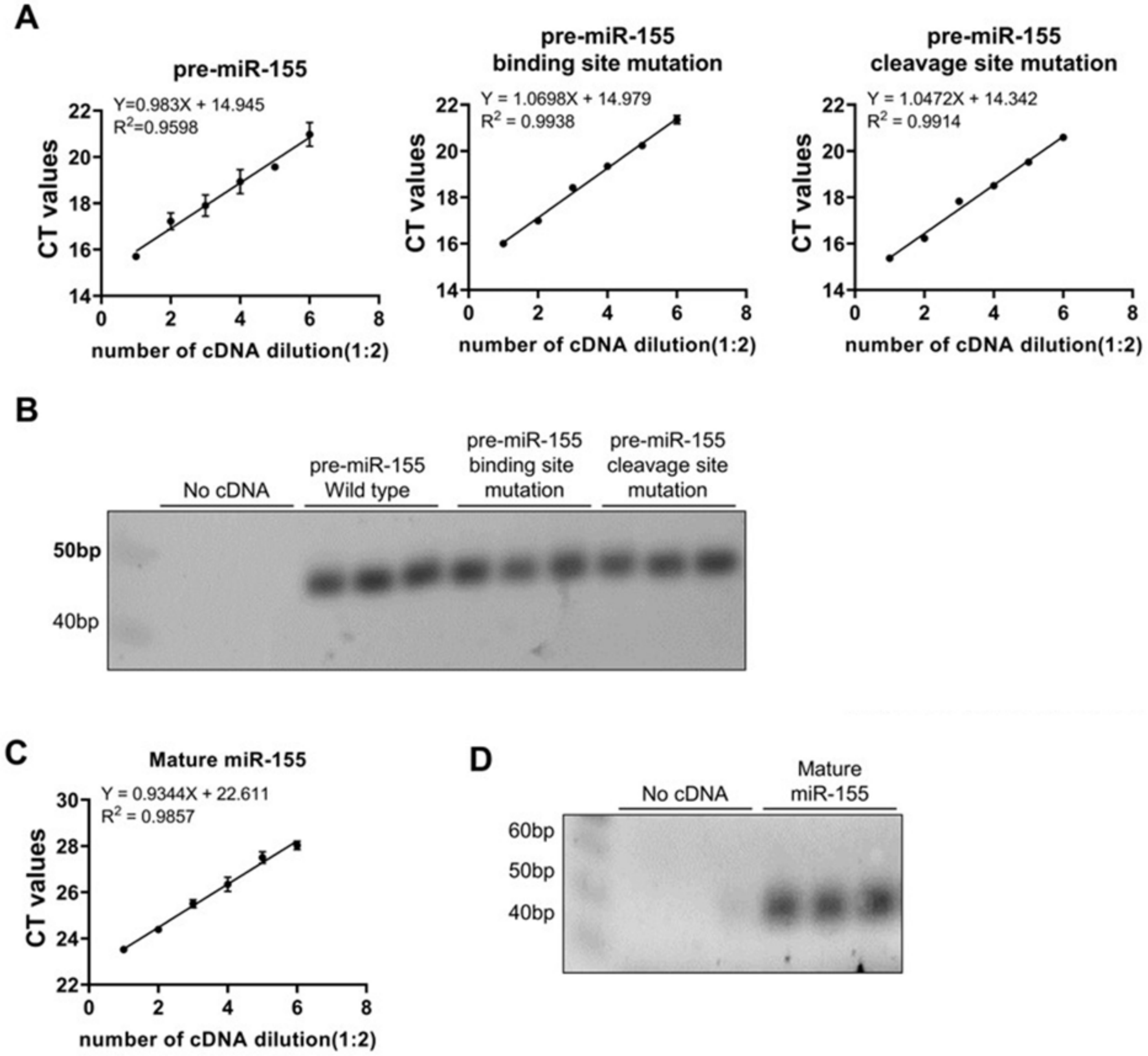

Supplementary Figure 2 | Validation of SYBR Green primers used to measure levels of mature and pre-miR-155 by RT-qPCR. **A**, Plot of C_t values as a function of 2-fold cDNA dilutions using primers for wild type pre-miR-155 (left) or mutants thereof (middle - binding site mutant; right - cleavage site mutant) (*n* = 3 biological replicates; the error bars are too small to be shown). **B**, RT-qPCR yielded single products for wild type pre-miR-155 and both mutants (*n* = 3 biological replicates). **C**, Plot of C_t values as a function of 2-fold cDNA dilutions using primers for mature miR-155, the sequence of which is the same after processing of wild type or mutant pre-miRNAs (*n* = 3 biological replicates). **D**, RT-qPCR yielded a single product for mature miR-155 (*n* = 3 biological replicates). All data are reported as the mean ± S.D. of the measured biologically independent replicates.

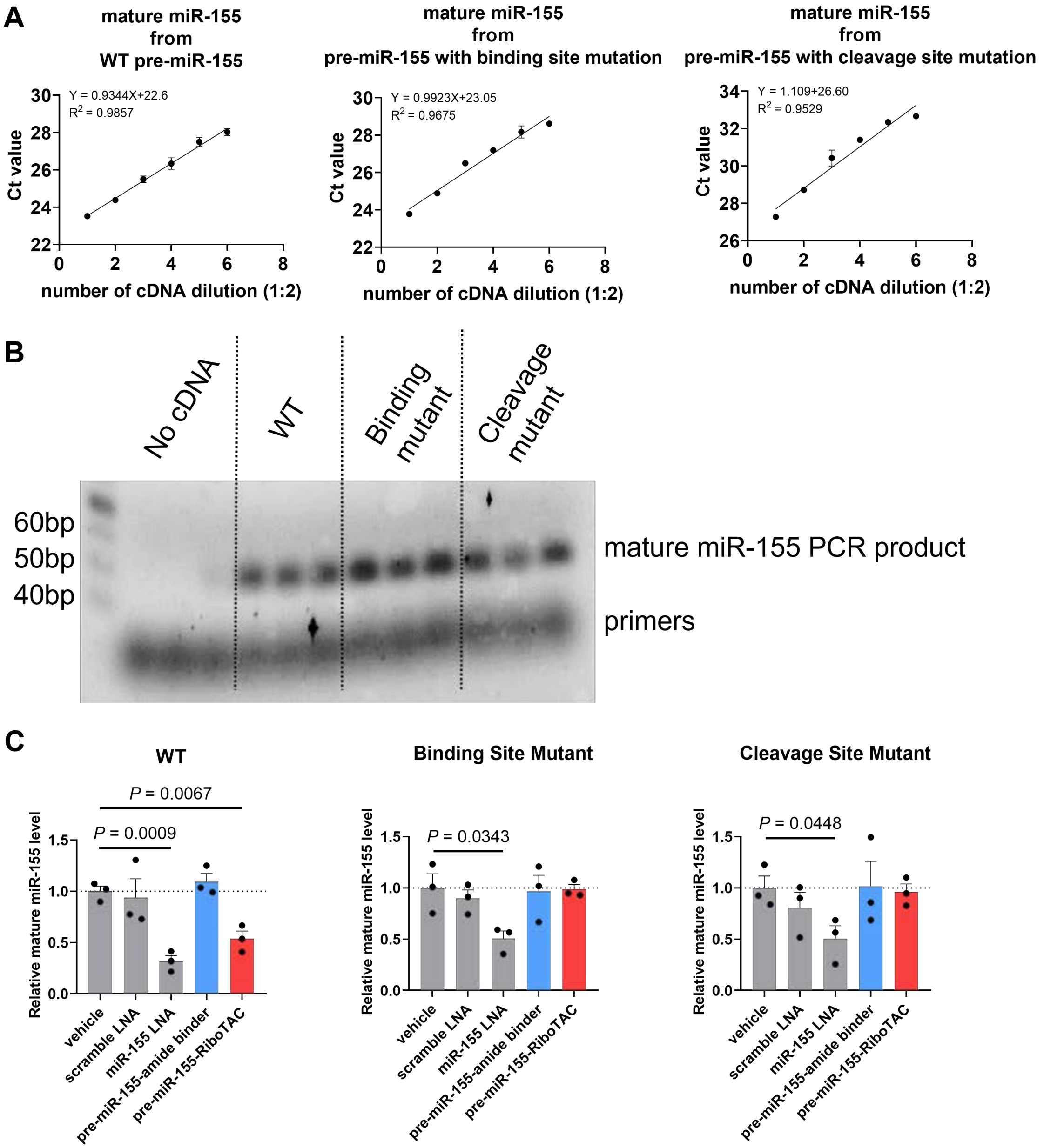

Supplementary Figure 3 | Validation of TaqMan primers used to measure levels of mature miR-155 by RT-qPCR. **A**, Plot of C_t values as a function of 2-fold cDNA dilutions using TaqMan primers for miR-155 in MCF-10a cells forced to express WT pre-miR-155 (left), the pre-miR-155 binding site mutant (middle), and the cleavage site mutant (right) (n = 3 biological replicates). **B**, Gel electrophoretic analysis of RT-qPCR products afforded a single band for WT miR-155 and both mutants (n = 3 biological replicates). **C**, Effects of **LNA-155** (50 nM), an antagomir directed at miR-155, **pre-miR-155-amide binder** (100 nM), and **pre-miR-155-RiboTAC** (100 nM) in MCF-10a cells forced to express WT pre-miR-155 (left), the pre-miR-155 binding site mutant (middle), and the cleavage site mutant (right), as assessed by RT-qPCR using TaqMan primers (n = 3 biological replicates). These results are in agreement with those obtained using SYBR Green primers (Extended

Data Fig. 9a). All data are reported as the mean ± S.D. of the measured biologically independent replicates. Statistical significance was calculated by a two-tailed Student's t-test.

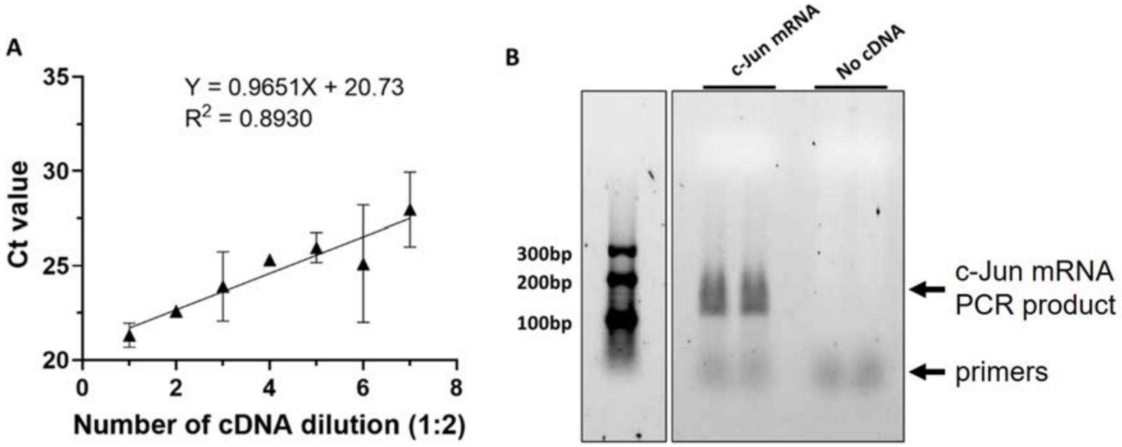

Supplementary Figure 4 | Validation of TaqMan primers used to measure levels of c-*JUN* mRNA by RT-qPCR. **A**, Plot of C_t values as a function of 2-fold cDNA dilutions by using *c-JUN* TaqMan primers (*n* = 3 biological replicates). **B**, Gel electrophoretic analysis of RT-qPCR products for *c-JUN* mRNA using TaqMan primers, as determined by agarose gel electrophoresis (*n* = 2 biological replicates). No amplification was observed when cDNA was not added (no template control). All data are reported as the mean ± S.D. of the measured biologically independent replicates.

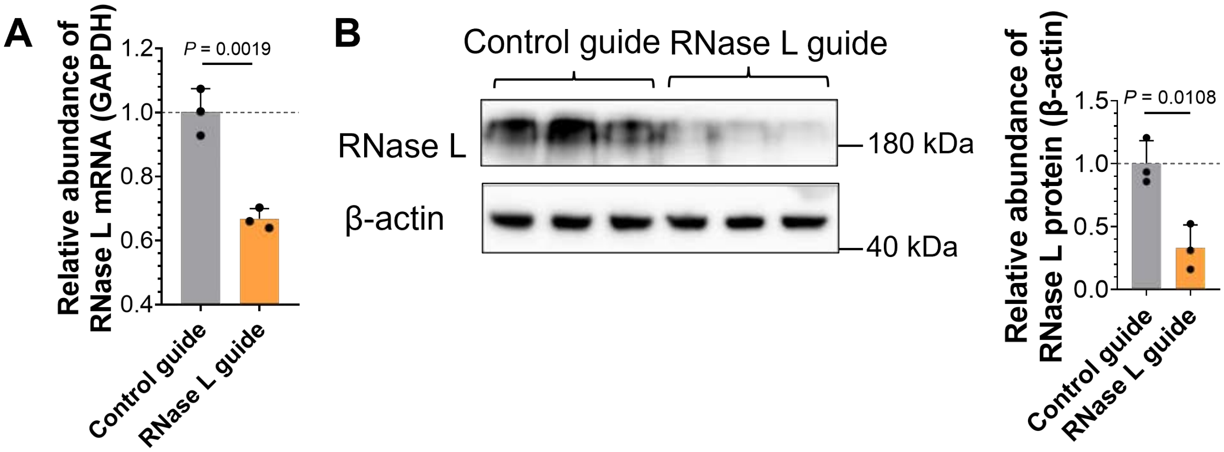

Supplementary Figure 5 | Validation of MIA PaCa-2 pancreatic adenocarcinoma cells in which of RNase L was knocked down by CRISPR. A, RNase L mRNA level in CRISPR-directed RNase L knockdown and control MIA PaCa-2 cells, as determined by RT-qPCR (n = 3 biological replicates). **B**, RNase L protein levels in RNase L knockdown and control MIA PaCa-2 cells, as determined by Western blotting (*n* = 3 biological replicates). All data are reported as the mean ± S.D. of the measured biologically independent replicates. Statistical significance was calculated by a two-tailed Student's t-test.

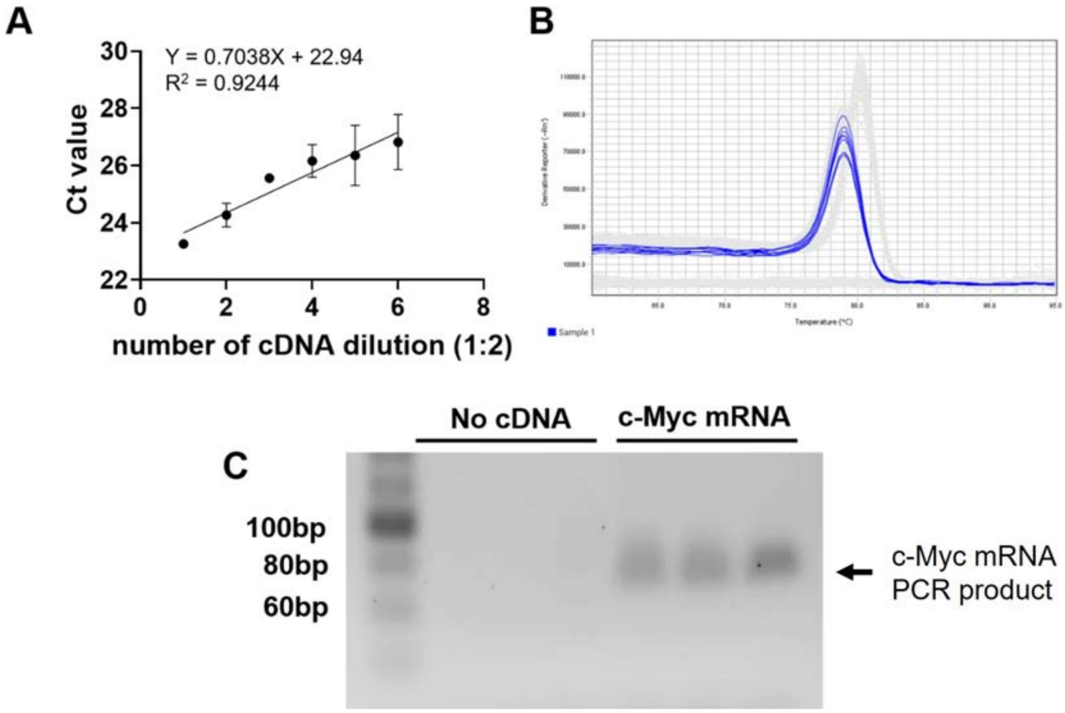

Supplementary Figure 6 | Validation of SYBR Green primers used to measure levels of *MYC* mRNA by RT-qPCR. A, Plot of C_t values as a function of 2-fold cDNA dilutions by using primers amplifying *MYC* mRNA (*n* = 3 biological replicates). **B**, Melting curve of the c-MYC RT-qPCR products (blue), completed after RT-qPCR (*n* = 3 biological replicates). **C**, Gel electrophoretic analysis of RT-qPCR products for *c-MYC* mRNA using SYBR Green primers (*n* = 3 biological replicates). All data are reported as the mean ± S.D. of the measured biologically independent replicates.

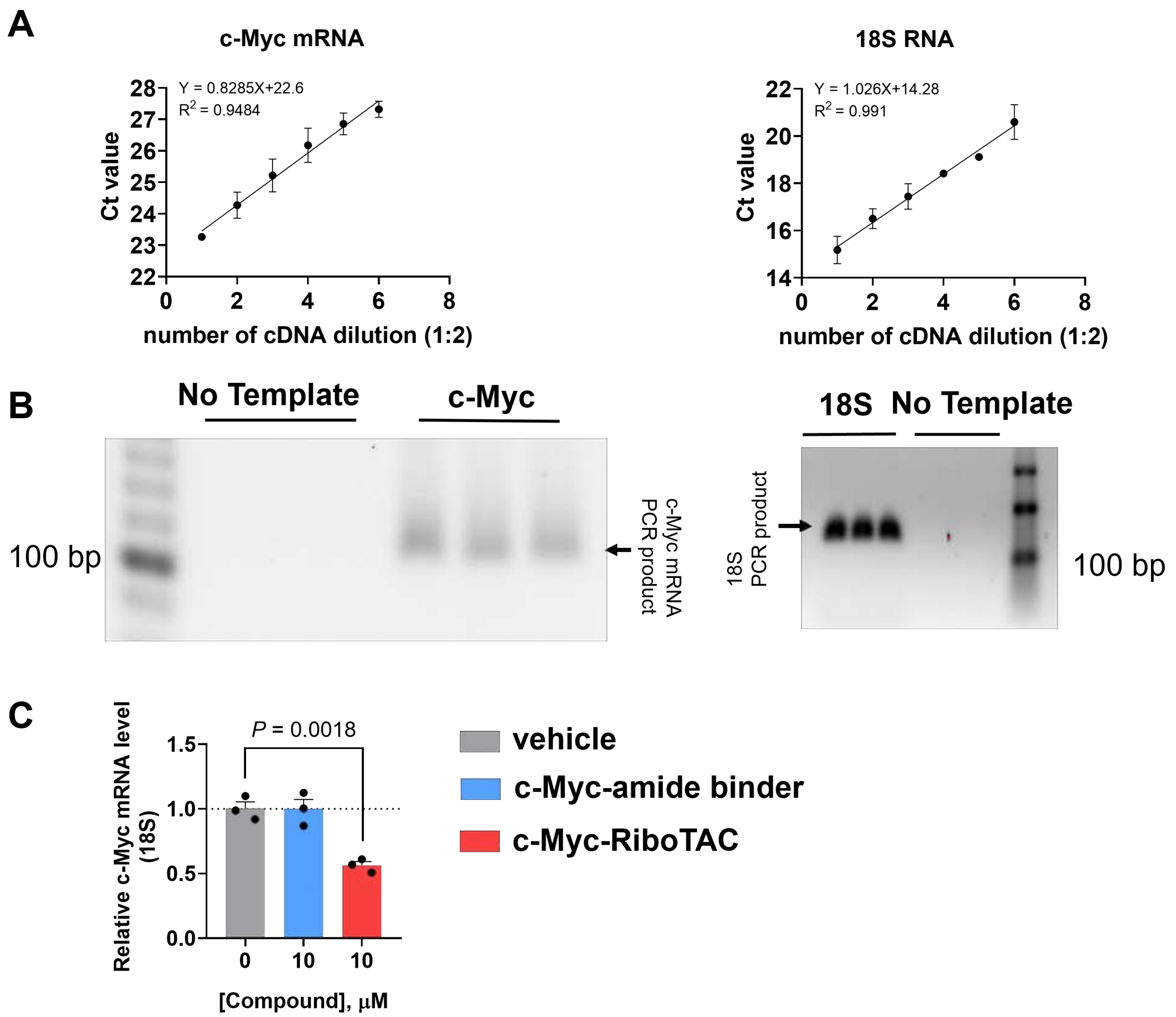

Supplementary Figure 7 | Validation of TaqMan primers used to measure levels of *MYC* mRNA by RT-qPCR. **A**, Plot of C_t values as a function of 2-fold cDNA dilutions by using TaqMan primers amplifying *MYC* mRNA and 18S RNA (*n* = 3 biological replicates). **B**, Gel electrophoretic analysis of RT-qPCR products for *c-MYC* mRNA and 18S rRNA using TaqMan primers (*n* = 3 biological replicates). **C**, Effects of **c-Myc-amide binder** (10 μM) and **c-Myc-RiboTAC** (10 μM) upon treatment of HeLa cells for 48 h (two doses at t = 0 and t = 24 h, as completed for SYBR Green primers) by TaqMan assay (*n* = 3 biological replicates). These results are in agreement with those obtained using SYBR Green primers (Fig. 5c). All data are reported as the mean ± S.D. of the measured biologically independent replicates. Statistical significance was calculated by a two-tailed Student's t-test.

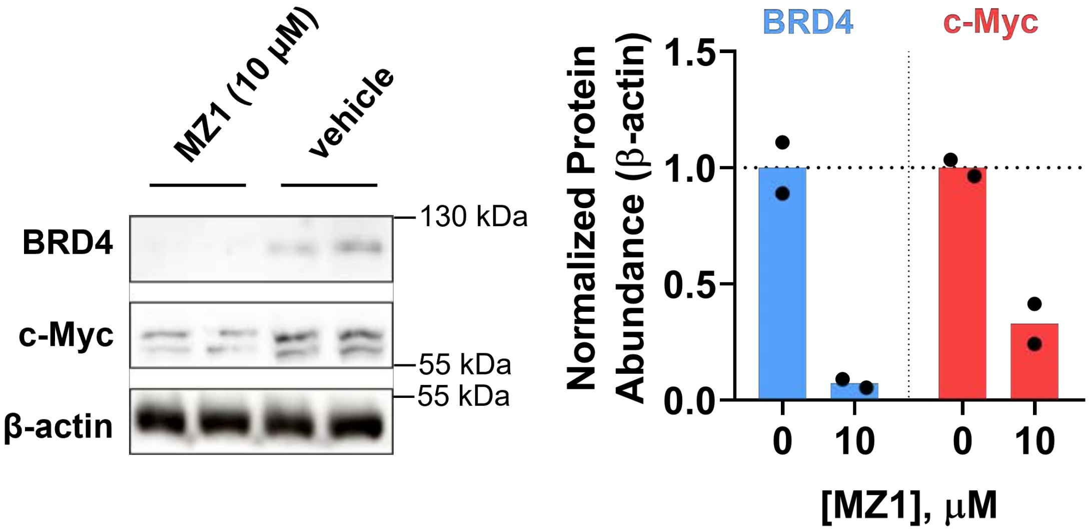

Supplementary Figure 8 | Effects of MZ1 on BRD4 and c-Myc protein abundances. BRD4 and c-Myc protein levels in HeLa cells treated with vehicle or MZ1 for 48 h, as determined by Western blotting (*n* = 2 biological replicates). The decrease of both proteins by MZ1 is consistent with previous literature reports. [49] Note that two bands of c-MYC proteins (both selected for quantification) are two expected isoforms, [59] which could merge into a single band depending on the separation of gel electrophoresis. All data are reported as the mean ± S.D. of the measured biologically independent replicates.

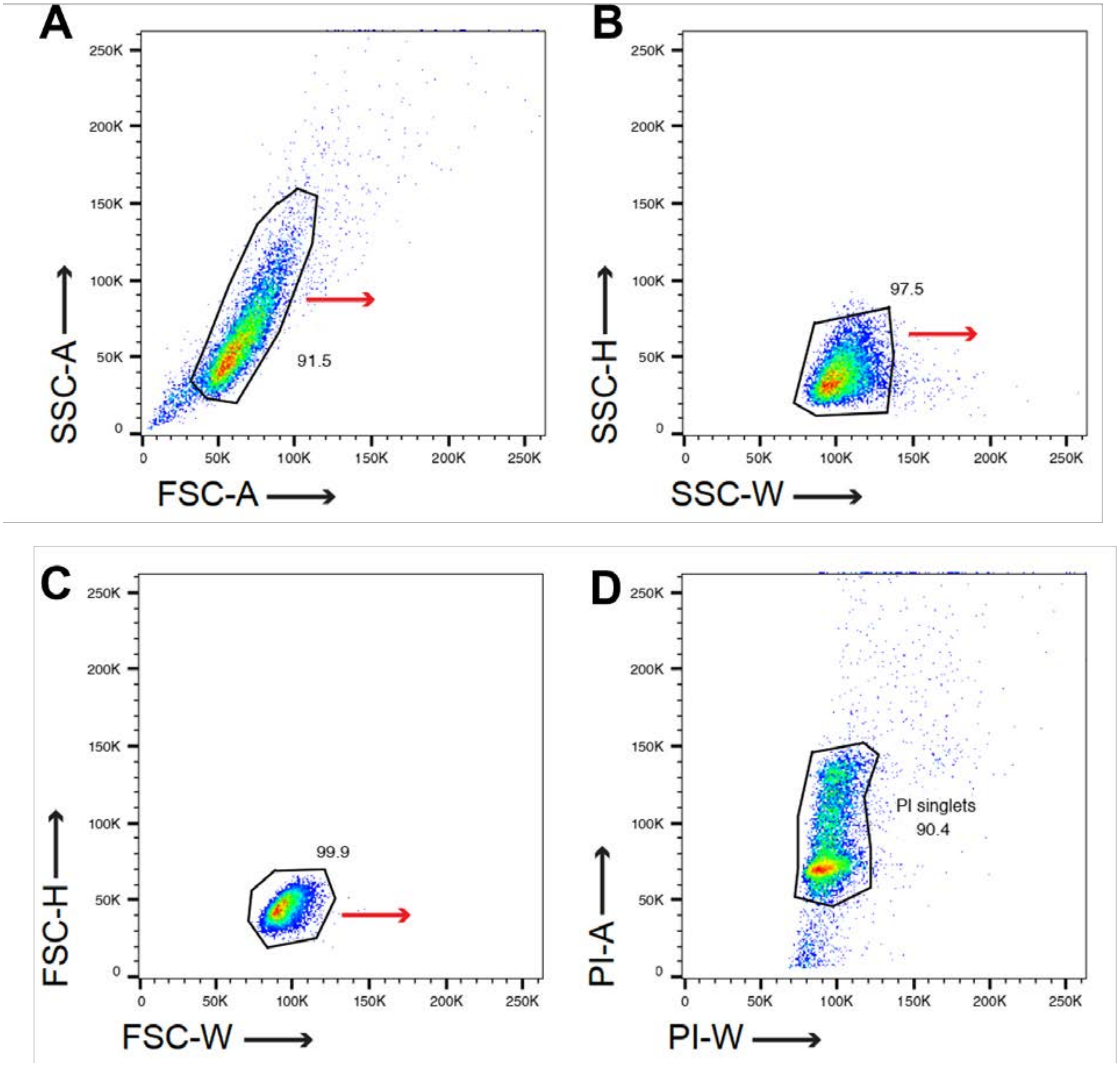

Supplementary Figure 9 | Illustration of the gating strategy used in flow cytometry. Cells were first selected for size **(A)** and subsequently gated on singlets **(B&C).** For PI-stained cells, cells were further selected for PI staining and singlets **(D)**.

SUPPLEMENTARY METHODS

Screening of natural product-like library by To-Pro-1 dye displacement

The RNA library was transcribed and purified as described.[60] The RNA was folded in 1× HTS Buffer (8 mM Na_2PO_4, pH 7.0, 185 mM NaCl, and 1 mM EDTA) by heating at 95 °C for 2 min and cooled down on ice. To-Pro-1 dye displacement assay was performed in DNase- and RNase-free water supplemented with 1× HTS Buffer following the protocol as reported previously in a 1536-well plate format. To-Pro-1 fluorescence (F_A) was measured by a Tecan Safire plate reader (Ex. 485 ± 5 nm; Em. 520 ± 5 nm) with a gain of 225, optimized to ensure at least 3-fold signal above background.

After reading, Hoechst 33258 (0, 1, 10, and 100 μM final concentration) or DMSO (0.1%, same as the compound) was added to each well using a BioMek 1536-well pin transfer tool (Beckman Coulter). After incubating for 1 h at room temperature, To-Pro-1 fluorescence (F_B) was measured as described above. The percent decrease in fluorescence was calculated using Equation (1):

$$\left(\frac{F_B - F_A}{F_B}\right) * 100 = \%\ decrease\ in\ fluorescence \qquad (1)$$

To determine this assay's amenability to HTS screening in this format, a Z-factor was calculated according to Equation (2):[61]

$$Estimated\ Z\ Factor = 1 - \frac{3(\sigma_p + \sigma_n)}{|\mu_p - \mu_n|} \qquad (2)$$

where σ_p and σ_n are the standard deviations of the percent changes of the positive (addition of Hoechst 33258) and negative (DMSO) controls, respectively, and μ_p and μ_n are the mean percent changes for the positive and negative control respectively. The Z-factor was calculated by measuring n = 690 wells per condition, which represents half of the 1536-well plate after excluding the edge wells.

Screening of the natural product-like small molecule collection

The above protocol was adapted to screen the natural product-like library using a Thermo Fisher F5 automation system, plate hotels for incubation, an Echo520 acoustic dispenser for compounds (Labcyte, Inc.), Multidrop Combi nL (ThermoFisher) dispensers for plating solutions, and an Envision plate reader (PerkinElmer) for measuring fluorescence.

In brief, the 3×3 ILL (200 nM final concentration) was folded as described above. A control RNA lacking the randomized region (Supporting Table 1) was used as a counter screen and prepared analogously. After folding, an equimolar amount of To-Pro-1 was added, and the samples were

equilibrated at room temperature for 30 min. Using the Multidrop Combi dispenser, 5 µL of the dye/RNA mixture was added to each well in a 1536-well plate (Greiner, catalog #782076). Note: control wells were also prepared that contain To-Pro-1 and 1× HTS Buffer but lacking RNA (minimum fluorescence). Fluorescence intensity was measured using a PerkinElmer EnVision® plate reader (Ex/Em: 480 ± 5 nm/535 ± 5 nm). After, 5 nL of compound was added to each well using an Echo 520 acoustic dispenser, and the plates incubated at room temperature in plate hotels for 30 min. All data were normalized to DMSO controls and then the percent decrease in fluorescence was calculated per Equation 1. The top 30% of compounds that reduced signal by > 30% were carried forward to study in dose response (n = 480).

Dose response curves were generated by using 3-fold dilutions (n = 8), with final concentrations ranging from 0.001 µM to 30 µM. Curves were fit to afford IC_{50} values using the Quatro Workflow software (Quattro-Research GmbH) (n = 3). Of the 480 compounds progressed to these studies, 344 displaced To-Pro-1 dose-dependently (Fig. 1b) and were screened by 2DCS to define their RNA binding landscapes.

Buffers used in 2DCS

Buffers and the methods for 2DCS were described previously and are as follows: [11,13] 1× Folding Buffer (FB): 20 mM HEPES, pH 7.5, 150 mM NaCl, and 5 mM KCl; 1× Hybridization Buffer (HB): 20 mM HEPES, pH 7.5, 150 mM NaCl, 5 mM KCl, 1 mM $MgCl_2$, and 40 µg/mL BSA.

2DCS: Primary screening

Microarrays were constructed as described. In brief, glass plates (L: 84 mm; W: 126 mm; H: 1 mm) were coated with 25 mL of molten 1% (w/v) agarose and left for 2 h at room temperature. Compounds (100 nL) were then delivered to the array surface using a Biomek® NX robotic pin-tool. The array was dried in a fume hood overnight and then washed with 1× FB supplemented with 0.1% (v/v) Tween-20 and twice with Nanopure water, and then air dried. The microarrays were pre-hybridized with 1× HB for 5 min, and excess buffer was removed by touching the edge with a Kimwipe. The arrays were the incubated with folded radioactively labeled 3×3 ILL[11] for 30 min at room temperature, spread evenly across the surface with a piece of parafilm. To fold the RNA library, ~200 pmol was folded in 0.5 mL of 1× FB by heating to 95 °C for 1 min and then slowly cooling to room temperature. After, $MgCl_2$ was added to a final concentration of 1 mM (*i.e.,* 1× HB), and the volume was adjusted to a total volume of 2.5 mL with 1× HB. After hybridization, the array was

washed four times with 1× HB, allowed to dry, and imaged by autoradiography. Hits were defined as compounds with radioactive signal >3-fold over background (n = 26). Mitoxantrone (100 nL of 10, 5 and 2.5 mM delivered to the surface) was used as a positive control.

2DCS selection in the presence of tRNA and competitor oligonucleotides

The 26 hits from the primary 2DCS screen, were carried forward to a 2DCS selection using radiolabeled 3×3 ILL, and unlabeled yeast tRNA and competitor oligonucleotides as described.[13] Briefly, the competitor oligonucleotides include (5'GGGUUUAAUUACGUAAUUGGAUCC; 5'GGGAGA; 5'GCAAGG; 5'CGCGAAAGCG; $d(GC)_{11}$; $d(At)_{11}$). RNAs that bound each compound were excised if the signal was >3-fold above the background radioactive signal from the array. Both the excised RNAs and the unselected, starting library was subjected to RNA-seq analysis as described.[11] After library preparation, cDNA samples were quantified on an Agilent Technologies 2100 Bioanalyzer (Model #: G1939A) and on a Qbit 2.0 (Invitrogen) fluorimeter. Sequencing was completed by an Ion Proton sequencer (Life Technologies) with > 200-fold coverage/base.

Statistical analysis of selected RNAs

High Throughput Structure-Activity Relationships Through Sequencing (HiT-StARTS) statistical analysis of the 2DCS selections was completed as described,[11] using a pooled population comparison according to Equations (3) and (4).

$$\phi = \frac{n_1 p_1 + n_2 p_2}{n_1 + n_2} \tag{3}$$

$$Z_{obs} = \frac{(p_1 - p_2)}{\sqrt{\phi(1-\phi)\left(\frac{1}{n_1} + \frac{1}{n_2}\right)}} \tag{4}$$

where n_1 is the total number of reads for all selected RNAs in the RNA-seq data; n_2 is the total number of reads for the starting library; p_1 is the proportion of the reads for a particular sequence in the total number of reads from the selected library; p_2 is the proportion of the reads for a particular sequence in the total number of reads from the starting library.

Fitness Scores were also calculated for each selected sequence (Z_{obs} > 8), calculated according to Equation (5):[13]

$$Fitness = \frac{Z_n - Z_{obs\,(8)}}{Z_{obs\,max} - Z_{obs(8)}} * 100 \tag{5}$$

where Z_n is the Z_{obs} for a particular sequence in the RNA-seq data; $Z_{obs\,(8)} = 8$;[11,13] and $Z_{obs,\,max}$ is the maximum Z_{obs} observed for the selection of interest.

Calculation of Tanimoto coefficients and physiochemical properties

Small molecule structures were analyzed for chemical similarity with the small molecules housed in Inforna[60] and R-BIND[17] databases. Using instant JChem (ChemAxon), an overlap analysis was completed using Chemically Hashed Fingerprinting to determine the structural similarity of **C1 – C20** to those small molecules contained in each database. This score was then averaged to afford the mean similarity to known RNA binders. Instant JChem was also used to calculate physicochemical properties, for structure database management, and for search and prediction [Instant JChem 19.8.0, 2019, ChemAxon (http://www.chemaxon.com)].

LOGO and DiffLOGOS RNA motif analysis

Sequences corresponding to the highest and lowest 0.5% of Z_{obs} values were used to generate LOGOS for preferred (highest) and discriminated against (lowest) RNA folds. The resulting sequences were individually converted to position weight matrix (PWM) lists using JMP®, Version 13.2.1 (SAS Institute Inc., Cary, NC, 1989-2007). The R package Difflogos,[62] which a part of Bioconductor, was utilized to create the sequence logos from PWM lists for each compound and visually compare the differences between them. DiffLOGO (version 1.36) was installed on RStudio (version 1.2.5042, RStudio Team 2020) with R 3.6.3 (R Core Team, 2014).

Analysis of ligandable sites in the human miRnome.

The analysis was performed by searching each 2DCS enriched motif against the miRNA motif database as previously published.[18] The position and distance of unpaired Us from the ligandable sites were determined manually by inspecting the secondary structures.

Binding affinity measurements by microscale thermophoresis (MST; pre-miR-155)

Binding constants were measured by MST Monolith instrument using premium capillaries (Nanotemper) using a constant concentration of RNA (10 nM) and 1:2 serial dilutions of the compound interest (50 µM to 1 nM). Both the compound of interest and RNA were prepared as 2x stocks in 1× HTS Buffer. Cy5-labeled RNAs (20 nM) were folded in 1× HTS Buffer by heating at 90 °C

for 1 min and slowly cooling to room temperature. Equal volumes (10 µL) of 2× compound and 2x RNA solutions were then mixed together, and the samples were allowed to equilibrate at room temperature for 15 min. The samples were then loaded into capillaries and analyzed with a Nanotemper Monolith NT.115 as described.[63] MST power was set to 40% and the LED power was set to 3%. The data points were fitted using Equation (6) embedded in the MO.AffinityAnalysis to obtain K_d.

$$f(c) = unbound + \frac{bound - unbound}{2} * \left(F + c + K_d - \sqrt{((F + c + K_d)^2 - 4 * F * c)}\right) + A \quad (6)$$

where F is the concentration of fluorescently labeled RNA; unbound and bound refer to the thermophoresis signal at completely unbound and bound state of RNA, respectively; c is the concentration of the compound; $f(c)$ is the thermophoresis signal at compound concentration of c; K_d is the dissociation constant; and A is a constant.

Measuring of binding affinity by monitoring 2AP fluorescence (pre-miR-155 and c-Jun)

RNAs in which the A bulge binding site, whether in pre-miR-155 or c-*Jun* (note the bulges have different closing base pairs; Extended Data Figs. 3d & 10a), were folded by heating the RNA (final concentration of 500 nM) in a 1× Assay Buffer at 60 °C for 5 min and slowly cooling to room temperature. BSA was then added to a final concentration of 40 µg/mL, and the sample was aliquoted. Different concentrations of compound or DMSO were added as indicated such that the final concentration of DMSO was 1% (v/v) in each sample. After incubating the complex at room temperature for 30 min, 2AP fluorescence intensity was measured using a Molecular Devices SpectraMax M5 plate reader using an excitation wavelength of 310 nm and an emission wavelength of 375 nm. The background fluorescence measured from a well with buffer only was subtracted, and EC_{50} values were calculated by fitting the curve to Equation 7:

$$Y = Min + \frac{(Max - Min)}{1 + (\frac{X}{EC_{50}})} \quad (7)$$

where Y is the measured fluorescence; Min and Max are minimum and maximum fluorescence measured among all data points on the curve, respectively; and X is the concentration of the compound.

For competitive binding assays with unlabeled WT or mutant pre-miR-155 RNA, the unlabeled

RNA was folded as described above to achieve a final concentration of 100 μM and then supplemented with BSA to a final concentration of 40 μg/mL. The RNA was serially diluted 2-fold (12 data points total) in 1× Assay Buffer supplemented with 40 μg/mL BSA. The compound of interest (final concentration of 5 μM) or DMSO was added to each sample such that the final concentration of DMSO was 1% (v/v). The 2AP-label RNA (1 μM) was prepared as described above and added at equal volume to each sample (500 nM final concentration) in a final volume of 200 μL. All samples were incubated at room temperature for 30 min, and 2AP fluorescence intensity was measured as described above. The competitive K_d was calculated by fitting the curve to Equation 8:

$$Y = a * \left(\frac{1}{2[F]}\right) * (K_d + \left(\frac{K_d}{EC_{50}}\right) * c + 2[F] - (K_d + \left(\frac{K_d}{EC_{50}}\right) * c + 2[F])^2 - 4[F]^2)^{0.5} + A \qquad (8)$$

where Y is the measured fluorescence; F is the concentration of 2AP-labeled RNA; c is the concentration of the compound; EC_{50} is the half maximal effective concentration calculated from Equation (7); K_d is the competitive dissociation constant; a and A are constants.

Binding affinity measurements by fluorescence quenching assay (c-Jun and c-Myc)

RNAs that model the c-*Jun* and *MYC* IRES or a fully base paired control thereof were labeled at the 5' end with Cy5 (Extended Data Figs. 9 and 10). The RNAs were folded in 1× Assay Buffer (final concentration of 50 nM) by heating at 60 °C for 5 min followed by cooling to room temperature. Upon cooling to room temperature, BSA was added to a final concentration of 40 μg/mL BSA to the folded c-*Jun* IRES but not the folded *MYC* IRES. The compound of interest or vehicle was added at the indicated concentrations such that the final concentration of DMSO in each sample was 1% (v/v). The samples were then incubated at room temperature for 30 min followed by measurement of Cy5 fluorescence intensity by a SpectraMax M5 plate reader using an excitation wavelength of 678 nm and an emission wavelength of 694 nm. For *MYC* IRES, the fluorescence intensity of compound alone at each concentration was also measured and then subtracted from the corresponding well containing RNA. Binding constants were then calculated by fitting the curve to equation 9.

$$\mathrm{Y} = \mathrm{B}_{max} * \frac{\mathrm{X^h}}{K_d^h + \mathrm{X^h}} \qquad (9)$$

where, Y is the relative fluorescence intensity, B_{max} is the maximum specific binding in the same units as Y, X is the concentration of RNA, h is the Hill slope, and K_d is the dissociation constant.

In vitro RNA cleavage by RNase L by gel electrophoresis (pre-miR-155)

The wild-type pre-miR-155 and the two mutant pre-miR-155 RNAs were prepared as described[64] using the corresponding DNA templates and primers provided in Extended Data Table 2. RNA (0.5 nmol) was dephosphorylated by using CIAP (Calf Intestinal Alkaline Phosphatase; Thermo Fischer) per manufacturer's protocol. After phenol-chloroform extraction and ethanol precipitation, the RNA was dissolved in water followed by adding PNK Kinase buffer (NEB)to a final concentration of 1×, T4 polynucleotide kinase, and [γ-^{32}P] ATP per manufacturer's protocol. The labeled RNA was then purified on a denaturing 15% polyacrylamide gel and isolated by extraction into 300 mM NaCl and ethanol precipitation. To perform cleavage assay, the purified, radiolabeled RNA (5 pmol per reaction) was folded in 1× FB by heating at 65 °C for 5 min and cooling to room temperature. The solution was supplemented with the following (final concentrations): 10 mM $MgCl_2$, 7 mM β-mercaptoethanol, 10 µM yeast tRNA (Roche), 50 µM ATP, and compound of interest at the indicated concentration. After incubating at room temperature for 15 min, RNase L was added to a final concentration of 25 nM, and the samples were incubated at room temperature for an additional 12 h. The fragments were separated by a denaturing 15% polyacrylamide gel. The gel was imaged by using a Typhoon FLA9500 imager (GE Healthcare) and quantified by ImageJ.

In vitro Chem-CLIP to study the binding of c-Jun-binder

The c-Jun SL-1 RNA (Dharmacon) was radiolabeled with [γ-^{32}P] ATP, and the RNA (~2000 CPM) was folded, both as described above. The RNA solution was then incubated with **c-Jun-Chem-CLIP** or **Ctr-Chem-CLIP** for 30 min at room temperature at varying concentrations. The samples were irradiated with UV light (365 nm) in a UVP crosslinker (analyticjena, 40W) for 10 min. To each reaction was then added a click reaction mixture [PEG3 biotin azide (Click chemistry tools, Cat#: AZ104, 0.8 µL, 10 mM), CuSO4 (0.5 µL, 10 mM), Tris(3-hydroxypropyltriazolylmethyl)amine (THPTA; 0.1 µL, 50 mM) and sodium ascorbate (0.1 µL, 250 mM)]. The samples were incubated at 37 °C for 3 h. After incubation, 8 µL of streptavidin beads (Dynabeads MyOne Streptavidin C1 beads; Thermo Scientific) was added to the mixture. Crosslinked RNA and unreacted RNA was separated by placing the reaction tubes on a magnetic separation rack per manufacturer's recommendations. The beads were then washed twice with 1× Wash Buffer (10 mM Tris-HCl, pH 7.0, 1 mM EDTA, 4 M NaCl, and 0.2% Tween). The radioactive signal associated with the beads and in the supernatant was measured by liquid scintillation counting and the fraction pulldown was calculated.

***In vitro* RNA cleavage by RNase L using a fluorescence-based assay (pre-miR-155, c-Jun, c-Myc)**

RNAs labeled with 5'-fluorescein and Black Hole Quencher at the 3' end were purchased from Dharmacon (Extended Data Table 2). The RNA of interest (final concentration of 100 nM) was folded 1× FB and then incubated with the RiboTAC of interest and RNase L as described above. After incubating the samples at room temperature for 12 h, fluorescein fluorescence intensity (Ex 484 nm; Em 525 nm) was measured by using a SpectraMax M5 plate reader. Background fluorescence of the compound was subtracted by measuring a well with same reaction composition but lacking RNA.

Construction of RNase L knockdown cells

To establish the optimal conditions for lentiviral transductions for knockdown of RNase L in MIA PaCa-2 cells, the cells were plated at three different densities (1×10^5, 2×10^5, and 4×10^5 cells per 9.6 cm^2 well). After 48 h, the cells were then treated with six different concentrations of puromycin (0 to 4 μg/mL) using Puromycin Solution (GenDEPOT, cat# CR026-001). The cells were cultured for an additional 72 h and counted using a TC20 automated cell counter (Bio-Rad Laboratories, Inc.) to determine the optimal plating density and IC_{95} for puromycin.

Lentiviral constructs containing Cas9 and gRNAs against RNASEL or a negative control were purchased from Transomic Technologies, Inc. (TEVH-1249025-pCLIP-ALL-hCMV-Puro, TEVH-1181883-pCLIP-ALL-hCMV-Puro, TEVH-1114741-pCLIP-ALL-hCMV-Puro, and TELA1015). These constructs are packaged by transfecting HEK293T cells (ATCC, CRL-11268) using Lipofectamine 2000 transfection reagent (Invitrogen, 11668500) and Cellecta packaging mix (Cellecta, CPCP-K2A) in Opti-MEM (Gibco, 31985070), following Invitrogen's recommended protocol. Virus supernatants were harvested 72 h after transfection, filtered with 0.45 μm PES filters (Mdi Membrane Technologies Inc., SYPL0602MNXX204), and used to transduce MIA PaCa-2 cells in the presence of 6 μg/mL polybrene (Millipore, TR-1003-G). Knockdown of RNase L mRNA and protein levels were validated by RT-qPCR and Western blotting, respectively (**Supplementary Figure 4**).

Cell viability assays (small molecules and RiboTACs targeting pre-miR-155)

MDA-MB-231 cells were plated in 96-well plates (2.5×10^4 cells/well). After reaching ~60% confluency, the compound of interest (**miR-155-binder**; **miR-155-amide binder**; **miR-155 RiboTAC**) or DMSO was added in growth medium, and the cells were incubated for an additional 48 h. The final concentration of DMSO in all samples was 0.1% (v/v). Cell viability was measured by using WST-1 assay (Sigma, catalog # 5015944001) according to the manufacturer's protocol.

siRNA knockdown of RNase L (pre-miR-155 and c-Myc)

The RNase L-targeting siRNA-2 (SMARTpool ON-TARGET plus) and control siRNA-2 (ON-TARGET plus) were purchased from Dharmacon. The siRNA (100 nM) was transfected with Lipofectamine RNAiMAX reagent (Invitrogen) per the manufacturer's protocol for siRNA transfection. For MDA-MB-231 cells, the cells were seeded to 12-well plates and grown until ~60% confluency before transfection. HeLa cells were seeded to 12-well plates and grown until at ~50% confluency before transfection. Cells were incubated with the transfection cocktail for 6 h, followed by recovery in fresh growth medium for 18 h, and then treated with vehicle (DMSO, 0.1%v/v) or compounds as described above.

Immunoprecipitation of RNase L (pre-miR-155)

RNase L immunoprecipitation studies were performed according to published procedures.[13] MDA-MB-231 cells (~60% confluency) were treated with vehicle, 100 nM of **pre-miR-155-RiboTAC**, or 100 nM of **pre-miR-155-Ctr** (RiboTAC control) in growth medium for 48 h. Samples were then harvested, followed by RNase L immunoprecipitation as previously described.[1] The amount of pre-miR-155 in the pulled down fraction and in the starting lysate were measured by RT-qPCR as described in "*RT-qPCR analysis of mRNAs and pri-, pre-, and mature miRNA levels*" in the manuscript's Methods section.

Cellular migration assay (pre-miR-155)

MDA-MB-231 and MCF-10a cells, the latter either mock-transfected or transfected with a plasmid encoding WT or mutated pre-miR-155, were pre-treated with compound at indicated concentrations in growth medium for 12 h. This medium was replaced with growth medium lacking FBS for another 12 h with the same concentration of the compound. The cells were then seeded to the ThinCert inserts with 8 μm pores (GBO, 657638) at 50,000 cells per well in a total volume of 100 μL of growth medium. The inserts were then placed in 24-well plates holding 650 μL of fresh growth medium containing FBS so that the bottom of the insert is covered by the fresh medium. After incubating for 24 h, all growth medium was removed, and cells were washed by 1× DPBS twice. The cells were then fixed by 4% (w/v) paraformaldehyde in 1× DPBS (400 μL per well) at room temperature for 30 min, followed by washing with 1× DPBS twice. The cells were stained by of 0.1% (w/v) crystal violet (400 μL per well) in 4:1 water/methanol and washed by 1× DPBS twice. A cotton swab was used to gently remove the non-migratory cells on the top side of the insert membrane. The remaining migratory

cells were then imaged by an optical microscope (two views captured per well) and counted manually.

Measuring angiogenic capacity in HUVECs (pre-miR-155)

Angiogenic tubule formation assays were performed by using a Culturex In Vitro Angiogenesis Assay Kit (Trevigen) as previously reported.[15] Briefly, approximately 1 × 10^5 HUVEC cells were seeded in a 96-well plate as a single cell suspension in growth medium (Lonza). Cells were treated with **pre-miR-155-RiboTAC** (100 nM) for 48 h. Images of cells were taken on a BioTek Lionheart FX automated microscope (20 field views per well). Image stitching was carried out directly on the Biotek imaging suite and the number of branch points were counted.

Cellular invasion assay (c-Jun)

ThinCert inserts with 8 μm pores (GBO, 657638) were coated with 100 μL of 0.3 mg/mL (w/v) Matrigel (Fisher Scientific; CB40234) per well and then incubated at 37 °C for 1 h. MIA PaCa-2 cells were prepared in growth medium lacking FBS supplemented with 2 μM **c-Jun-RiboTAC** or DMSO (vehicle; 0.1% (v/v), the final concentration in all compound-treated samples) and added to the Matrigel-coated inserts (50,000 cells per insert). The inserts were then placed in 24-well plates holding 650 μL of fresh growth medium containing FBS so that the bottom of the insert was covered by the fresh growth medium. After incubating for 24 h, the compound-containing medium was removed from the inserts, which were then washed with 1× DPBS twice. The remaining steps for fixing, staining, and imaging are identical as described for cellular migration assays.

Cellular proliferation assay (c-Jun and c-Myc)

Cell proliferation assays were completed using CellTiter 96® AQueous One Solution Cell Proliferation Assay Solution (Promega, G3580) per manufacturer's protocol. HeLa cells were seeded into 96-well white, clear bottom tissue culture plates (Corning, 3610). Upon reaching ~30% confluency, the cells were either treated with 0.1 - 10 μM of **c-Myc-RiboTAC, MZ1**, or DMSO (vehicle; 0.1% (v/v), the final concentration in all compound-treated samples) for 48 h. After 48 h incubation, 20 μL of the proliferation assay solution was added to each well, and the samples were incubated at 37 °C for 30 min. The absorbance at 490 nm was then measured using a SpectraMax M5 fluorescence plate reader. Similarly, the effect of **c-Jun-RiboTAC** (2 μM) on the proliferation of MIA PaCa-2 were measured by seeding at ~40% confluency and treating for 48 h.

Caspase 3/7 assay to measure apoptosis

HeLa cells were seeded into 96-well white, clear bottom tissue culture plates (3×10^4 cells per well). Upon reaching ~30% confluency, the cells were treated with 0.1 - 10 µM of **c-Myc-RiboTAC**, **MZ1,** or DMSO (vehicle; 0.1% (v/v), the final concentration in all compound-treated samples) for 48 h. Caspase 3/7 activity was measured using Caspase-Glo 3/7 Assay Kit (Promega) according to the manufacturer's instructions. Namalwa (0.4×10^6 cells/mL, 1 mL), HL-60 (0.5×10^6 cells/mL, 1 mL), and Raji (0.7×10^6 cells/mL, 1 mL) were treated with vehicle (0.1% (v/v) DMSO; the final concentration in all compound-treated samples), **c-Myc-RiboTAC** (10 µM), or **c-Myc-Ctr** (10 µM) for 48 h. For Namalwa, HL-60, and Raji cells, apoptosis was measured by CellEvent Caspase-3/7 Green ReadyProbes Reagent (ThermoFisher, C10423) and DAPI (0.1 µg/mL, Molecular Probe) following the manufacturer's protocol. Dye was directly added to flow cytometry analysis buffer (1× DPBS, 10% (v/v) FBS). Samples were analyzed in a BD LSRII (BD Biosciences) flow cytometer.

Global proteomics profiling by using LC-MS/MS

For pre-miR-155: MDA-MB-231 cells were seeded in 60 mm dishes at ~60% confluency and treated with DMSO (0.1% (v/v); the final concentration in all compound-treated samples), **LNA-155** (100 nM; added directly to the cells in growth medium) or **pre-miR-155-RiboTAC** (100 nM) for 48 h. For MYC: HeLa cells were grown in 100 mm dish (~30% confluency) and treated with DMSO (vehicle; (0.1% (v/v); the final concentration in all compound-treated samples)) or **c-Myc-RiboTAC** (10 µM) for 24 h and then redosed with fresh medium containing compound for additional 24 h. HeLa cells were also grown in 100 mm dish (~40% confluency) and transfected with c-Myc siRNA (1 nM) or scrambled siRNA (1 nM) as described by using Lipofectamine 3000 per manufacturer's protocol. The total protein was harvested 48 h later.

Protein samples were harvested following protocol as reported previously.[65] Protein samples (~20 µg) were denatured with 6 M urea in 50 mM NH_4HCO_3, pH 8, reduced with 10 mM tris(2-carboxyethyl)phosphine hydrochloride (TCEP) for 30 min, and then alkylated with 25 mM iodoacetamide for 30 min in the dark. The samples were diluted to 2 M urea solution with 50 mM NH_4HCO_3, pH 8, and then digested with trypsin (1 µL of 0.5 µg/µL) in the presence of 1 mM $CaCl_2$ for 12 h at 37 °C. The digested samples were acidified with acetic acid, added to a final concentration of 5% (v/v), desalted over a self-packed C18 spin column, and dried. Samples were analyzed by LC-MS/MS by using a Q Exactive HF-X mass spectrometer and the MS data were processed with MaxQuant (v2.3.0.0) as described.[65]

Analysis of global proteomics data

Global proteomics data were acquired as described.[65] Data obtained from the LC-MS-MS run were analyzed as follows: MS data were analyzed with MaxQuant[66] (V1.6.1.0) and searched against the human proteome (Uniprot) and a common list of contaminants (included in MaxQuant). The peptide search tolerance was set at 20 ppm, 10 ppm was used for the main peptide search, and fragment mass tolerance was set to 0.02 Da. The false discovery rate for peptides, proteins, and sites identification was set to 1%. The minimum peptide length was set to 6 amino acids, and peptide re-quantification and label-free quantification (MaxLFQ) were enabled. The minimal number of peptides per protein was set to 2. Methionine oxidation was searched as a variable modification, and carbamidomethylation of cysteines was searched as a fixed modification.

To compare differences of protein $\log_2$(fold changes) of the downstream protein targets of c-Myc, HIF1-α, miR-155-5p, or miR-18a-5p targets versus all proteins, a Kolmogorov-Smirnov test was used to compare the distance between two cumulative fractions and compute the corresponding p-value. The miR-155-5p downstream targets were identified by using TargetScanHuman 7.0[35] (only targets with conserved sites), affording 469 targets, 63 of which were detected by proteomics analysis. The same searching for miR-18a-5p downstream targets afforded 264 targets, 42 of which were detected by proteomics analysis. Myc downstream targets were identified from previous reports[51-53]. Among 404 targets, 83 proteins were detected by proteomics. HIF-1α downstream targets were identified from a previous report[67], affording 476 targets, 63 of which were detected by proteomics analysis.

RNA-seq analysis

For pre-miR-155: MDA-MB-231 cells were seeded into 12-well plates and treated with DMSO (vehicle), **pre-miR-155-RiboTAC** (100 nM), or **LNA-155** (100 nM; added to the cells in growth medium; no transfection required) for 48 h.

For MYC: HeLa cells were seeded into 12-well plates and treated with DMSO (vehicle) or **c-Myc-RiboTAC** (10 μM) for 24 h. Then, cells were re-treated with fresh growth medium containing compound for an additional 24 h. HeLa cells were transfected with *MYC* siRNA (1 nM) or scrambled siRNA (1 nM) as described above, and total RNA has harvested 48 h later. Total RNA was extracted using the Zymo Quick-RNA MiniPrep Kit according to the manufacturer's protocol.

Total RNA was then isolated with miRNeasy Kit (QIAGEN), and quantified using a Qubit 2.0 Fluorometer (Invitrogen, Carlsbad, CA). RNA quality was evaluated by using an Agilent 2100 Bioanalyzer RNA nano chip (Agilent Technologies, Santa Clara, CA). Approximately 200 ng of total

RNA was depleted of ribosomal RNA using NEBNext rRNA depletion module (E6310L, NEB) according to manufacturer's recommendations. Library preparation from the rRNA-depleted RNA was performed using NEBNext Ultra II Directional RNA kit (E7760, NEB), also per manufacturer's protocol. The final libraries were validated by a Bioanalyzer analysis, pooled to equimolar concentrations, and loaded onto the NextSeq 500 v2.5 flow cell. Libraries were sequenced with 2 x 40 bp paired-end chemistry. Approximately 20 million reads pass filter (base quality score > Q30) for each sample. Raw sequence reads were aligned to the human genome by STAR (v2.5.2a).[68] Raw counts were extracted from .bam files by featurecounts (v2.0.0),[69] and differential gene expression was analyzed by DESeq2 (v3.13).[70]

To compare differences of $\log_2$(fold changes) of the downstream RNA targets of c-Myc and HIF-1α targets versus all transcripts, we used a Kolmogorov-Smirnov test to compare the distance between two cumulative fractions and compute the corresponding p-value. The same downstream transcripts of Myc and HIF-1α described above for protein targets were evaluated.

Synthetic Methods and Compound Characterization

General Methods

Chemicals were procured from the following sources: acetic acid, Avantor Performance Materials; N-Boc-ethylenediamine Boc-Lys(Ac)-OH, *N*-(2-α-aminoethyl)biotinamide, and Boc-Lys-OH, Combi-Blocks; 2,3-butanedione, Fisher Scientific; *n*-Butyl Lithium (*n*-BuLi), Sigma-Aldrich; 5-carboxyfluorescein, Combi-Blocks; chlorambucil acid, Alfa Aesar; 2,6-diisopropylaniline, VWR; *N,N*-diisopropylethylamine, Sigma-Aldrich; *N*, *N*-dimethylformamide (DMF), Fisher Chemical; ethyl bromoacetate, Fisher Scientific; HATU, Oakwood Products; propylamine, Alfa Aesar; and tetrahydrofuran, Fisher Scientific. All chemicals were used as received without further purification.

The progress of all reactions was monitored by LC-MS or thin layer chromatography (TLC), where compounds were visualized by using 254 nm UV light. All small molecules were purified on a Waters 2487 or 1525 HPLC by reverse phase chromatography (19 × 150 mm SunFire® Prep C18 OBDTM 5 μm column). A flow rate of 5 mL/min was used with a gradient of 100% H_2O (+ 0.1% trifluoroacetic acid (TFA)) to 100% MeOH (+ 0.1% TFA) over 60 min unless otherwise noted. The purity of products was determined using analytical HPLC by equipping a 4.6 × 150 mm SunFire® C18 3.5 μM column to either instrument above. Analytical HPLC traces were collected by using a Waters 1525 HPLC by reverse phase chromatography (4.6 × 150 mm SunFire® Analytical C18 3.5 μm

column), monitoring absorbance at 220 nm and 255 nm. A liner gradient of 100% H_2O + 0.1% TFA to 100% MeOH + 0.1% TFA over 60 min was applied unless specified otherwise.

The identify of reaction products was determined by ^{1}H NMR and ^{13}C NMR spectroscopy. Spectra were acquired on a 400 UltraShield™ (Bruker) (400 MHz for ^{1}H and 100 MHz for ^{13}C) or an Ascend™ 600 (Bruker) (600 MHz for ^{1}H and 150 MHz for ^{13}C). All chemical shifts are reported in ppm. Residual solvent peaks were used as an internal standard. Coupling constants are reported in Hz. Identity was further confirmed by high resolution mass spectrometry, obtained with an Agilent 1260 Infinity LC system coupled to an Agilent 6230 TOF (HR-ESI). The LC system was equipped with a Poroshell 120 EC-C18 column (Agilent, 50 mm × 4.6 mm, 2.7 μm). MALDI-TOF analysis was performed on a 4800 Plus MALDI TOF/TOF analyzer. All compounds were fully soluble at concentrations and conditions specified for each experiment.

Synthetic Methods and Characterizations

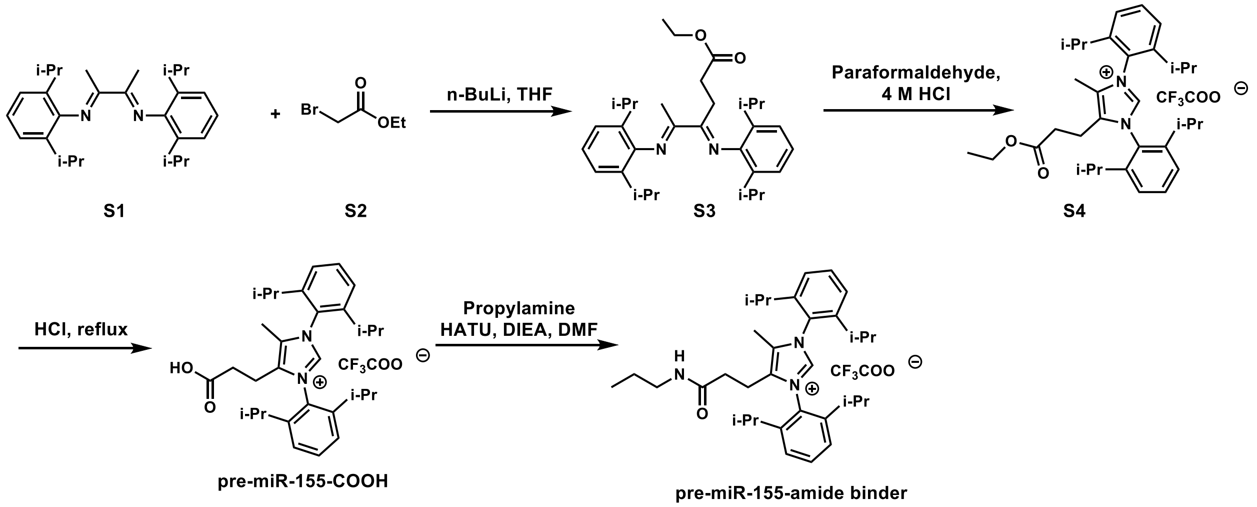

Scheme S1. **Synthesis of pre-miR-155-amide binder**.

Compound S4. Compound **S1** was synthesized as previously reported.[71] To a solution of **S1** (404 mg, 1 mmol) in dry tetrahydrofuran (THF) at −80 °C was added 500 µL of *n*-BuLi (2 M, 500 µL, 1 mmol), and the mixture was stirred at −80 °C for 20 min and then at room temperature for 20 min. The mixture was then cooled to −80 °C again, followed by the addition of ethyl bromoacetate (167 mg, 1 mmol). The reaction was allowed to warm to room temperature and stirred for another 4 h. The mixture was then concentrated *in vacuo*, and the residue was re-suspended in 4 M HCl in dioxane, followed by addition of paraformaldehyde (58 mg, 2 mmol). The reaction mixture was stirred overnight at room temperature and concentrated *in vacuo*. The residue was purified by HPLC as described in the **General Methods** to give **S4** as a white solid (100 mg, 0.162 mmol, 16%). **^{1}H NMR** (400 MHz, $CDCl_3$) δ (ppm) 9.38 (s, 1H), 7.60 (m, 2H) 7.38 (m, 4H) 4.09 (q, *J*=7.1 Hz, 2H), 2.83 (t, *J*=7.0 Hz, 2H), 2.38 (t, *J*=7.04Hz, 2H), 2.27 (m, 4H), 2.17 (s, 3H), 1.33 (d, *J*=6.8 Hz, 6H), 1.25 (d, *J*=6.8 Hz, 6H), 1.22 (d, *J*=7.1 Hz, 3H), 1.17 (m, 12H); **^{13}C NMR** (100 MHz, $CDCl_3$) δ (ppm) 171.0, 145.2, 145.1, 136.7, 132.6, 132.4, 130.9, 130.9, 127.5, 125.2, 125.0, 61.1, 29.2, 29.1, 25.8, 24.7, 23.2, 22.5, 18.8, 14.1, 9.2.

Pre-miR-155-COOH. A solution of **S4** (100 mg, 0.162 mmol) in 3 mL of concentrated HCl was stirred under reflux overnight. The mixture was concentrated *in vacuo* to give compound **pre-miR-155-COOH** as a white solid, which was used without further purification.

Pre-miR-155-amide binder. A solution of **pre-miR-155-COOH** (3 mg, 0.005 mmol), propylamine (0.3 mg, 0.015 mmol), HATU (3.8 mg, 0.01 mmol), and *N,N*-diisopropylethylamine (DIPEA, 1.9 mg, 0.015 mmol) in DMF was stirred at room temperature for 2 h. **Pre-miR-155-amide binder** was purified by HPLC as described in the **General Methods**, affording a white solid (2.1 mg, 0.0033 mmol, 66%). **^{1}H NMR** (400 MHz, $CDCl_3$) δ (ppm) 8.04 (s, 1H), 7.65 (br, 1H), 7.63 (m, 2H), 7.40 (m, 4H), 3.11 (m, 2H), 2.91 (t, *J*=7.8 Hz, 2H), 2.50 (t, *J*=7.8 Hz, 2H), 2.43 (m, 2H), 2.34 (m, 2H), 2.21 (s, 3H), 1.49 (m, 2H), 1.35 (d, *J*=6.7 Hz, 6H), 1.30 (d, *J*=6.8 Hz, 6H), 1.16 (d, *J*=6.9 Hz, 6H), 1.13 (d, *J*=6.9 Hz, 6H), 0.87 (t, *J*=7.4 Hz, 3H); **^{13}C NMR** (150 MHz, $CDCl_3$) δ (ppm) 170.8, 145.8, 145.7, 133.6, 133.0, 132.9, 132.7, 131.7, 127.5, 125.5, 125.3, 41.6, 33.3, 29.1, 29.1, 26.1, 25.3, 23.3, 22.8, 22.5, 19.9, 11.5, 9.2; **HRMS** (m/z): calculated for $C_{34}H_{50}N_3O$ $[M]^+$: 516.3948, found: 516.3978.

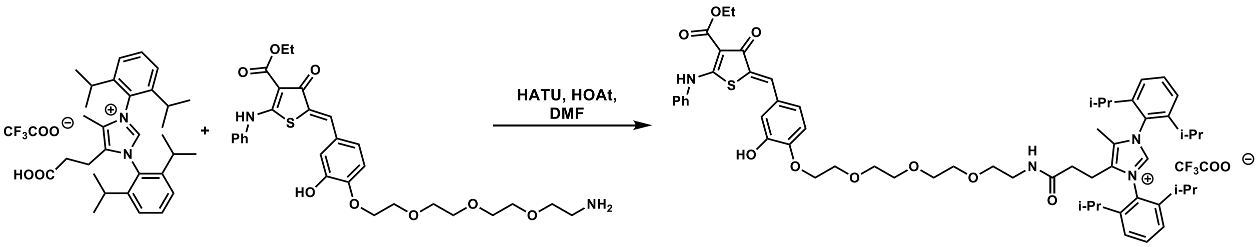

C1-COOH S5 pre-miR-155-RiboTAC

Scheme S2. Synthesis of pre-miR-155-RiboTAC.

Pre-miR-155-RiboTAC. A solution of **pre-miR-155-COOH** (3 mg, 0.005 mmol), HATU (3.8 mg, 0.01 mmol), and DIPEA (1.9 mg, 0.015 mmol) in 0.2 mL of DMF was stirred at room temperature for 20 min. **S5**, synthesized as previously reported,[1] (5.7 mg, 0.01 mmol) was then added, and the mixture was stirred at room temperature for another 2 h. The product was purified by HPLC as described in the **General Methods** to afford **pre-miR-155-RiboTAC** (1.3 mg, 0.0012 mmol, 23%). **^{1}H NMR** (600

MHz, CD_3OD) δ (ppm) 9.75 (s, 1H), 7.64-7.68 (m, 3H), 7.45-7.57 (m, 9H), 6.98-7.02 (m, 2H), 4.39 (q, *J*= 7.1 Hz, 2H), 4.16-4.22 (m, 2H), 3.84-3.88 (m, 2H), 3.68-3.72 (m, 2H), 3.63-3.66 (m, 2H), 3.59-3.63 (m, 2H), 3.54-3.58 (m, 2H), 3.48 (t, *J*= 5.4 Hz, 2H), 3.28 (t, *J*=5.4 Hz, 2H), 2.81 (t, *J*=7.4 Hz, 2H), 2.41-2.47 (m, 2H), 2.35-2.41 (m, 2H), 2.32 (t, *J*=7.5 Hz, 2H), 2.13 (s ,3H), 1.39 (t, *J*=7.1 Hz, 3H), 1.34 (d, *J*=6.7 Hz, 6H), 1.29 (d, *J*=6.8 Hz, 6H), 1.20 (d, *J*=6.9 Hz, 6H), 1.17 (d, *J*=6.9 Hz, 6H); **^{13}C NMR** (100 MHz, CD_3OD) δ (ppm) 184.5, 178.1, 172.5, 167.1, 150.2, 148.5, 147.1, 147.0, 138.8, 138.5, 133.7, 133.5, 133.3, 133.0, 132.1, 131.0, 129.5, 129.3, 129.3, 128.5, 126.4, 126.3, 126.2, 124.8, 117.5, 114.5, 99.0, 71.6, 71.6, 71.5, 71.2, 70.6, 70.4, 69.4, 61.4, 40.5, 33.8, 30.2, 20.2, 26.2, 25.4, 23.4, 22.9, 20.4, 14.8, 9.4. **HRMS** (m/z): calculated for $C_{59}H_{75}N_4O_9S$ $[M]^+$: 1015.5249, found: 1015.5256.

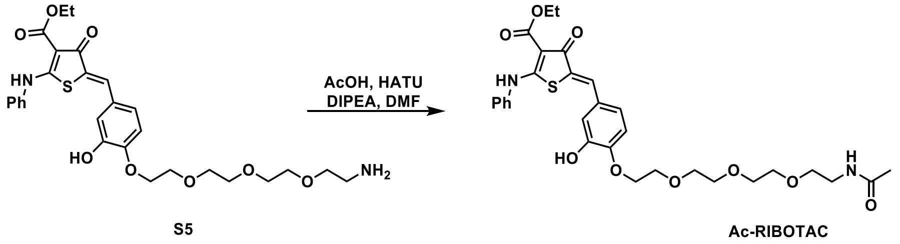

Scheme S3. Synthesis of control compound Ac-RiboTAC.

Ac-RiboTAC. To a solution of HATU (5.7 mg, 0.015 mmol) and DIPEA (5 μL, 0.03 mmol) in 0.5 mL of DMF was added 10 μL of acetic acid solution (1 M AcOH in DMF). After stirring at room temperature for 10 min, **S5** (5.6 mg, 0.01 mmol) was added to the mixture. The reaction was stirred at room temperature overnight and then purified by HPLC as described in the **General Methods** to give **Ac-RiboTAC** as a TFA salt (3.4 mg, 0.0056 mmol, 56%). **^{1}H NMR** (600 MHz, CD_3OD) δ (ppm) 7.66 (s, 1H), 7.53-7.58 (m, 2H), 7.44-7.53 (m, 3H), 6.99-7.07 (m, 3H), 4.38 (q, *J*=7.1 Hz, 2H), 4.18-4.23 (m, 2H), 3.82-3.90 (m, 2H), 3.69-3.74 (m, 2H), 3.65-3.68 (m, 2H), 3.56-3.60 (m, 2H), 3.50 (t, *J*=5.6 Hz, 2H), 1.91 (s, 3H), 1.39 (t, *J*=7.1 Hz, 3H). **^{13}C NMR** (150 MHz, CD_3OD) δ (ppm) 183.1, 176.9, 172.0, 148.8, 147.1, 137.4, 131.7, 129.6, 128.1, 127.1, 125.0, 124.9, 123.4, 116.1, 113.0, 97.5, 70.2, 70.1, 69.8, 69.2, 69.1, 67.9, 60.0, 39.1, 21.1, 13.4. **HRMS** (m/z): calculated for $C_{30}H_{37}N_2O_9S$ $[M+H]^+$: 601.2214, found: 601.2214.

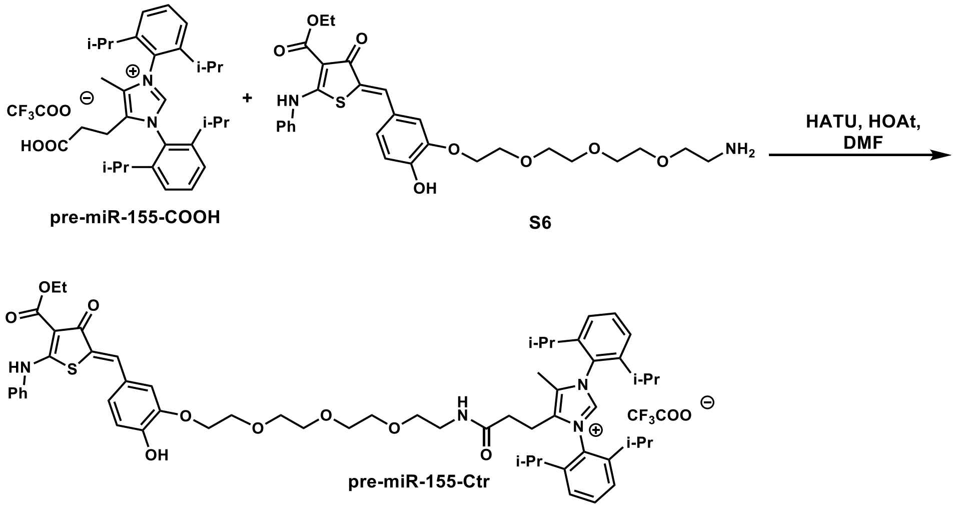

Scheme S4. Synthesis of control compound pre-miR-155-Ctr.

Pre-miR-155-Ctr. A solution of **pre-miR-155-COOH** (3 mg, 0.005 mmol), HATU (3.8 mg, 0.01 mmol), and DIPEA (1.9 mg, 0.015 mmol) in 0.2 mL of DMF was stirred at room temperature for 20 min. Then, **S6** (5.7 mg, 0.01 mmol), synthesized as previously reported,[1] was added, and the mixture was stirred at room temperature for another 2 h. The product was purified by HPLC as described in the **General Methods** to afford the **pre-miR-155-Ctr** as a TFA salt (1.5 mg, 0.0014 mmol, 27%). **^{1}H NMR** (600 MHz, CD_3OD) δ (ppm) 9.75 (s, 1H), 7.64-7.70 (m, 3H), 7.43-7.5 8(m, 9H), 7.15 (d, J=2.0 Hz, 1H), 7.07 (dd, J=8.3 Hz, 2 Hz, 1H), 6.83 (d, J=8.3 Hz, 1H), 4.38 (q, J=7.1 Hz, 2H), 4.13-4.18 (m, 2H), 3.81-3.86 (m, 2H), 3.66-3.70 (m, 2H), 3.62-3.65 (m, 2H), 3.58-3.62 (m, 2H), 3.54-3.58 (m, 2H), 3.48 (t, J=5.4 Hz, 2H), 3.28 (t, J=5.2 Hz, 2H), 2.81 (t, J=7.4 Hz, 2H), 2.41-2.50 (m, 2H), 2.34-2.41 (m, 2H), 2.32 (t, J=7.4 Hz, 2H), 2.13 (s, 3H), 1.39 (t, J=7.1 Hz, 3H), 1.34(d, *J*=6.8 Hz, 6H), 1.29(d, *J*=6.8Hz, 6H), 1.19 (d, *J*=6.8 Hz, 6H), 1.17 (d, *J*=6.8 Hz, 6H); **^{13}C NMR** (150 MHz, CD_3OD) δ (ppm) 184.5, 178.0, 172.5, 167.1, 151.0, 148.4, 147.1, 147.0, 138.8, 138.5 133.6, 133.5, 133.3, 133.3, 132.1, 131.0, 129.5, 129.3, 129.3, 126.9, 126.4, 126.3, 126.2, 125.7, 125.7, 117.4, 99.0, 71.6, 71.6, 71.5, 70.7, 70.4, 69.7, 61.4, 40.5, 33.8, 30.2, 30.2, 26.2, 25.4, 23.4, 22.9, 20.4, 14.8, 9.4. **HRMS** (m/z): calculated for $C_{59}H_{75}N_4O_9S$ $[M]^{+}$: 1015.5249, found: 1015.5267.

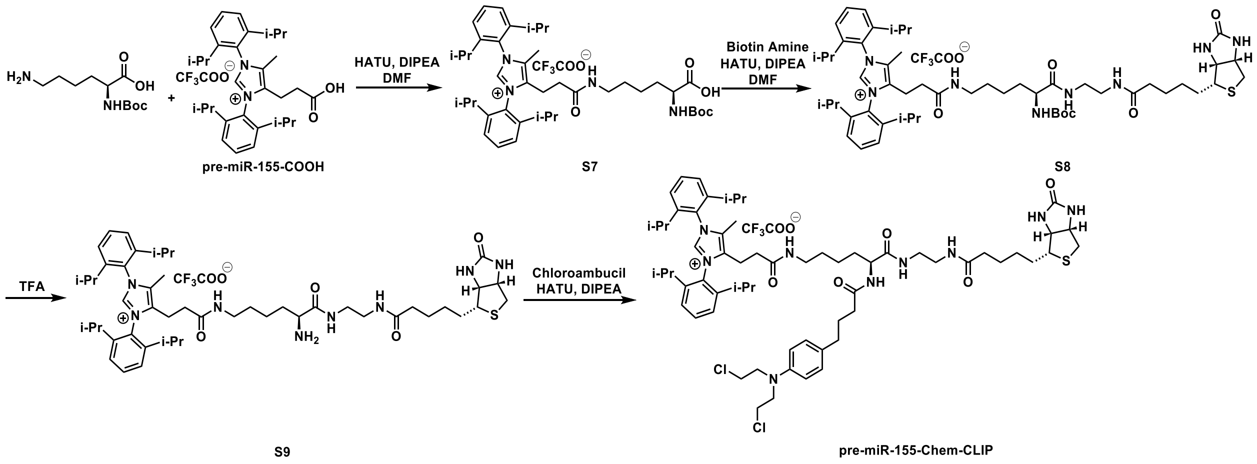

Scheme S5: Synthesis of pre-miR-155-Chem-CLIP.

Pre-miR-155-Chem-CLIP. A solution of **C1-COOH** (3 mg, 0.005 mmol), HATU (2.3 mg, 0.006 mmol), and DIPEA (2 mg, 0.015 mmol) in 0.2 mL of DMF was stirred at room temperature for 30 min. Boc-Lys-OH (6.2 mg, 0.025 mmol) was then added, and the mixture was stirred for another 30 min at room temperature. The crude mixture was purified by HPLC as described in the **General Methods** to give **S7**, which was proceeded to the next step without further purification. A solution of **S7**, biotin-amine (2.9 mg, 0.01 mmol), HATU (3.8 mg, 0.01 mmol) and DIPEA (2 mg, 0.015 mmol) in 0.2 mL of DMF was stirred at room temperature for 2 h. The reaction was then subjected to HPLC purification as described in the **General Methods** to afford **S8**, which was proceeded to the next step without further purification. A solution of **S8** in 30% (v/v) TFA in dichloromethane (DCM) was stirred at room temperature for 30 min and concentrated *in vacuo*. The residue was directly used in the next step without further purification. A solution of chlorambucil acid (3 mg, 0.01 mmol), HATU (3.8 mg, 0.01 mmol), and DIPEA (2 mg, 0.015 mmol) in 0.2 mL DMF was stirred at room temperature for 20 min, and then the amine obtained above was added in 0.1 mL of DMF. The mixture was stirred at room temperature for another 30 min, and **pre-miR-155-Chem-CLIP** was obtained by HPLC purification as described in the **General Methods** (0.6 mg, 0.45 µmol). ^{1}H NMR (600 MHz, DMSO-d_6) δ (ppm) 9.94(s, 1H), 7.69(m, 2H), 7.54(dd, J=5-8 Hz, 4H), 7.00(d, J=9 Hz, 2H), 6.65(d, J=9 Hz, 2H),

4.30(dd, J=5-8 Hz, 1H), 4.11(m, 2H), 3.69(t, J=4 Hz, 8H), 3.17(s, 3H), 3.07(m, 4H), 2.95(m, 3H), 2.81(dd, J=5-13 Hz, 1H), 2.69(t, J=8 Hz, 2H), 2.56(m, 2H), 2.39(m, 5H), 2.30(quint, J=7 Hz, 2H), 2.21(t, J=8 Hz, 2H), 2.11(m, 2H), 2.06(s, 2H), 2.03(t, J=8 Hz, 2H), 1.70(m, 2H), 1.59(m, 2H), 1.46(m, 4H), 1.31(m, 8H), 1.24(d, J=7 Hz, 6H), 1.11(dd, J=7-12 Hz, 12H); ^{13}C NMR (151 MHz, DMSO-d_6) δ (ppm) 172.29, 169.42, 162.72, 158.15, 157.92, 145.33, 145.24, 144.39, 136.62, 132.18, 131.58, 129.97, 129.81, 129.30, 127.79, 127.76, 125.03, 124.98, 111.86, 61.00, 59.21, 55.39, 52.20, 48.59, 41.15, 40.43, 38.51, 35.20, 34.68, 33.65, 32.41, 31.58, 28.69, 28.43, 28.37, 28.19, 28.02, 27.35, 25.43, 25.18, 24.71, 22.94, 22.71, 22.29, 18.75, 8.67. **HRMS** (m/z): calculated for $C_{63}H_{92}Cl_2N_9O_5S$ $[M]^+$: 1156.6314, found: 1156.6311.

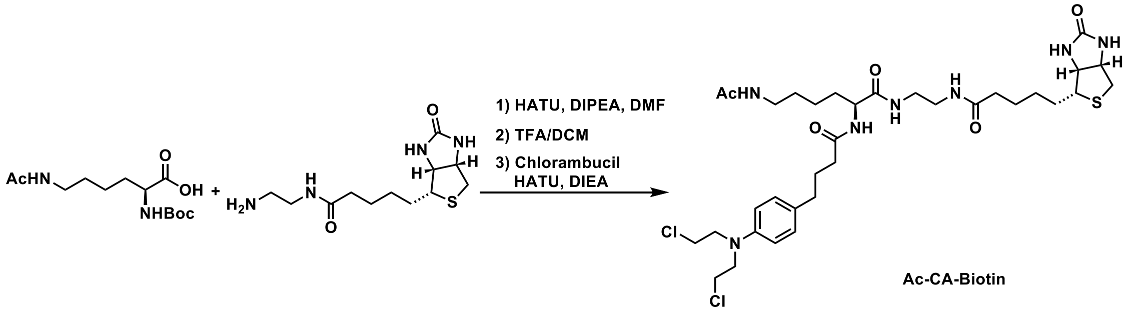

Scheme S6. Synthesis of control Chem-CLIP probe Ac-CA-Biotin.

Ac-CA-Biotin: A solution of Boc-Lys(Ac)-OH (5.7 mg, 0.02 mmol), biotin amine (5.7 mg, 0.02 mmol), HATU (8.4 mg, 0.022 mmol), and DIPEA (3.97 mg, 0.03 mmol) in 0.11 mL of DMF was stirred at room temperature for 2 h, and then the mixture was concentrated in *vacuo*. The resulting residue was re-suspended in 1 mL of 30% TFA / 70% DCM. The mixture was stirred at room temperature for another 30 min and then dried *in vacuo*. A solution of chlorambucil (12.2 mg, 0.04 mmol), HATU (22.8 mg, 0.06 mmol), and DIPEA (9.9 μL, 0.06 mmol) in 0.2 mL of DMF was stirred at room temperature for 20 min, followed by the addition of the crude primary amine. The mixture was stirred at room temperature for another 30 min and then purified by HPLC as described in the **General Methods** to

give the control Chem-CLIP probe **Ac-CA-Biotin** (0.3 mg, 0.34 μmol, 2%). **HRMS** (m/z): calculated for $C_{34}H_{54}Cl_2N_7O_5S$ $[M]^+$: 742.3279, found: 742.3276.

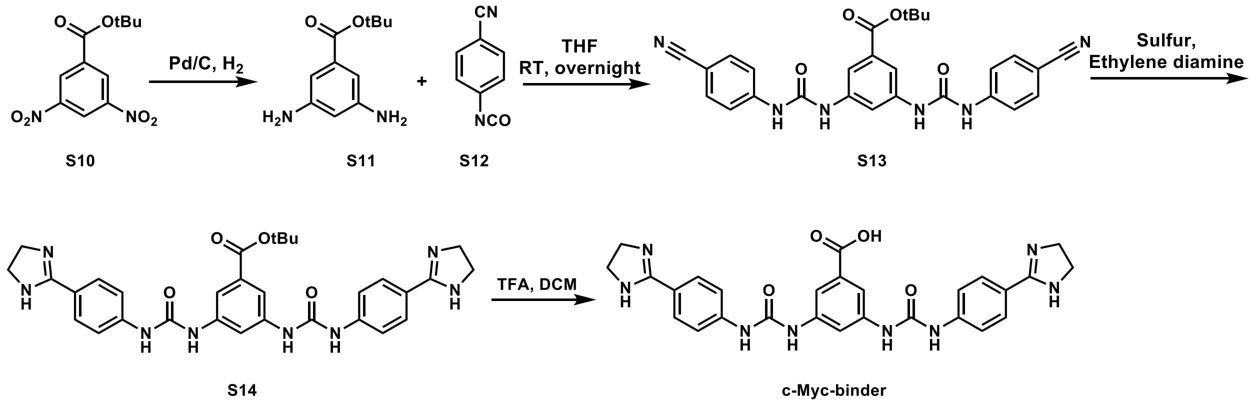

Scheme S7. Synthesis of c-Myc-binder.

Compound S13. Compound **S11** was synthesized as previously reported.[72] To a solution of **S11** (540 mg, 2.6 mmol) in THF, was added **S12** (1.12g, 7.79 mmol). The mixture was stirred at RT for overnight. Then, the precipitate was collected by filtration to give **S13** as a white solid (1.16 g, mmol, 90%). **^{1}H NMR**(400 MHz, DMSO-d_6) δ (ppm) 9.13 (d, 4H) 7.95 (s, 1H), 7.73(m, 4H), 7.65 (m, 6H), 1.54 (s, 9H); **^{13}C NMR** (100 MHz, DMSO-d_6) δ (ppm) 165.1, 152.5, 144.5, 140.3, 133.8, 132.8, 119.7, 118.9, 118.7, 113.4, 112.7, 103.9, 81.3, 28.2; **HRMS** (m/z): calculated for $C_{27}H_{24}N_6O_4$ $[M+H]^+$: 497.1859, found: 497.1856.

c-Myc-binder. To a solution of **S13** (168 mg, 0.339 mmol) in ethylenediamine (5 mL), was added sulfur (16.2 mg, 0.508 mmol). The mixture was stirred at 80 °C for 90 min with microwave irradiation using a Biotage Flash Chromatography. The reaction was added an additional 2 mL of DMSO and sulfur (10.9 mg, 0.339 mmol) to dissolve the resulting precipitate. The reaction was microwaved at 70 °C for an additional 90 min. Next, 15 mL of distilled water was added, and the precipitated product was collected by filtration to afford **S14** as a white solid (118 mg, 0.2032 mmol, 60%). **S14** was used

in next reaction without further purification. To a solution of **S14** (58.2 mg, 0.1 mmol) in DCM (200 µL) was added TFA (800 µL). The reaction mixture was stirred at room temperature for 1 h followed by removal of TFA with air. The reaction mixture was then purified by HPLC as described in the **General Methods** (13 mg, 0.025 mmol, 25%). **^{1}H NMR** (400 MHz, DMSO-d_6) δ (ppm) 10.30 (s, 4H) 9.76 (s, 2H), 9.60 (s, 2H), 7.87 (m, 7H), 7.75 (s, 2H), 7.73 (s, 2H), 3.97 (s, 8H); **^{13}C NMR** (100 MHz, DMSO-d_6) δ (ppm) 167.6, 164.9, 164.8, 152.6, 152.5, 146.2, 140.4, 132.3, 130.2, 120.2, 118.2, 117.3, 114.9, 114.4, 113.9, 112.8, 111.6, 44.6, 44.5; **HRMS** (m/z): calculated for $C_{27}H_{26}N_8O_4$ $[M+H]^+$: 527.2150, found: 527.2125.

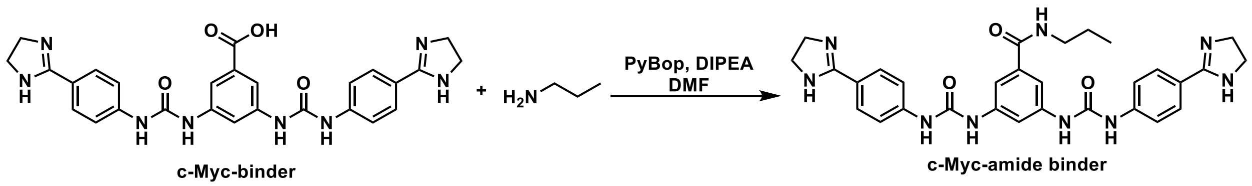

Scheme S8. Synthesis of c-Myc-amide binder.

c-Myc-amide binder. To a solution of **c-Myc-binder** (10 mg, 0.019 mmol) in DMF (500 µL) were added PyBOP (9.9 mg, 0.019 mmol) and DIPEA (12.2 µL, 0.076 mmol). The mixture was stirred at room temperature for 10 min followed by addition of propylamine (1.87 µL, 0.023 mmol). The reaction was incubated at room temperature for an additional 2 h. After reaching completion as determined by LC-MS, the reaction mixture was purified by HPLC (MeOH 20-100% in H_2O + 0.1% (v/v) TFA) to afford **c-Myc-amide binder** (2.0 mg, 19% yield). **^{1}H NMR** (600 MHz, DMSO-d_6) δ (ppm) 10.31 (s, 3H), 9.63 (s, 2H), 9.42 (s, 2H), 8.40 (m, 1H), 7.89 (d, J = 8.8 Hz, 4H), 7.81 (s, 1H), 7.74 (d, J = 8.9 Hz, 4H), 7.55 (d, J = 1.9 Hz, 2H), 3.98 (s, 8H), 3.18 (m, 2H), 1.53 (m, 2H), 0.90 (t, J = 7.4 Hz, 3H); **^{13}C NMR** (150 MHz, DMSO-d_6) δ (ppm) 164.9, 152.6, 146.2, 140.1, 137.0, 130.2, 120.5, 118.5, 118.1, 116.5, 114.9, 114.6, 112.2, 111.6, 47.2, 47.1, 46.3, 44.7, 44.5, 42.5, 41.5, 40.9, 40.5, 26.3, 22.8, 11.9; **HRMS** (m/z): calculated for $C_{30}H_{33}N_9O_3$ $[M+H]^+$: 568.2779 found: 568.2774.

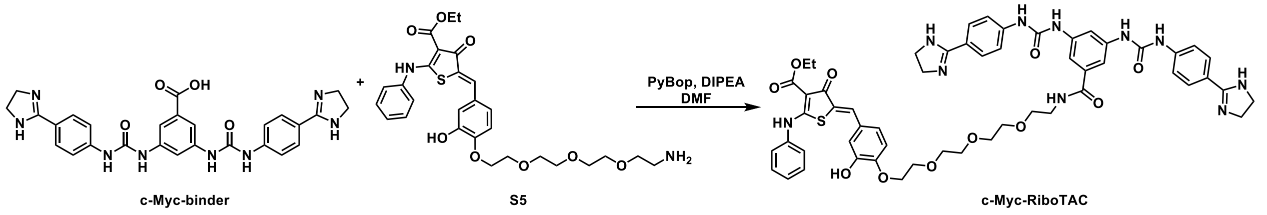

Scheme S9. Synthesis of c-Myc-RiboTAC.

c-Myc-RiboTAC. To a solution of **c-Myc-binder** (10 mg, 0.019 mmol) in DMF (500 μL) were added PyBOP (9.9 mg, 0.019 mmol) and DIPEA (12.2 μL, 0.076 mmol). The mixture was stirred at room temperature for 10 min. Then, **S5** (12.7 mg, 0.023 mmol) was added, and the mixture was stirred overnight at room temperature. After confirming the reaction went completion by LC-MS, the reaction mixture was purified by HPLC (MeOH 20-100% in H_2O + 0.1% (v/v) TFA) to obtain **c-Myc-RiboTAC** (4.1 mg, 20% yield). **^{1}H NMR** (400 MHz, DMSO-d_6) δ (ppm) 10.28 (s, 3H), 9.53 (s, 2H), 9.42 (s, 2H), 9.33 (s, 2H), 8.41 (t, J = 5.1 Hz, 1H), 7.87 (t, J = 8.9 Hz, 5H), 7.73 (d, J = 8.9 Hz, 4H), 7.52 (m, 6H), 7.46 (m, 2H), 6.96 (m, 3H), 4.26 (q, J = , 2H), 4.07 (m, 2H), 3.98 (s, 8H), 3.70 (m, 2H), 3.54 (s, 8H), 3.41 (m, 2H), 1.29 (m, 3H); **^{13}C NMR** (150 MHz, DMSO-d_6) δ (ppm) 181.5, 175.8, 167.1, 165.3, 164.8, 152.6, 149.2, 146.2, 140.1, 137.9, 136.6, 130.5, 130.2, 130.1, 128.7, 126.8, 126.1, 126.0, 125.1, 123.6, 118.5, 118.1, 116.6, 116.3, 114.9, 114.2, 112.2, 97.4, 70.3, 70.2, 70.1, 69.3, 68.4, 67.1, 60.0, 49.1, 40.8, 39.1, 14.9; **HRMS** (m/z): calculated for $C_{55}H_{58}N_{10}O_{11}S$ $[M+H]^+$: 1067.4080, found: 1067.4082.

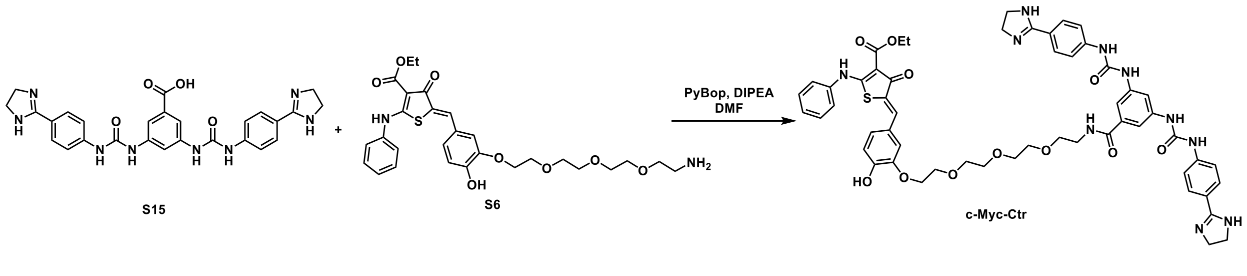

Scheme S10. Synthesis of c-Myc-Ctr.

c-Myc-Ctr. To a solution of **c-Myc-binder** (10 mg, 0.019 mmol) in DMF (500 μL) was added PyBOP (9.9 mg, 0.019 mmol) and DIPEA (12.2 μL, 0.076 mmol). After stirring the mixture at room

temperature for 10 min, **S5** (12.7 mg, 0.023 mmol) was added, and the mixture was stirred overnight at room temperature. After confirming reaction completion by LC-MS, the product was purified by HPLC (MeOH 20-100% in H_2O + 0.1% (v/v) TFA) to afford **c-Myc-Ctr** (3.5 mg, 17% yield). **^{1}H NMR** (400 MHz, DMSO-d_6) δ (ppm) 11.23 (s, 1H), 10.27 (s, 3H), 9.80 (s, 1H), 9.47 (s, 1H), 9.27 (s, 1H), 8.41 (m, 1H), 7.89 (t, *J* = 9.0, 4H), 7.73 (d, *J* = 9.0, 4H), 7.53 (m, 5H), 7.45 (m, 1H), 7.14 (d, *J* = 2.0, 1H), 6.99 (q, *J* = 3.4, 1H), 6.88 (d, *J* = 8.3, 1H), 4.27 (d, *J* = 7.1, 2H), 4.06 (m, 2H), 3.98 (s, 6H), 3.70 (m, 2H), 3.54 (s, 8H), 3.78 (s, 6H), 1.29 (t, *J* = 7.1, 3H); **^{13}C NMR** (150 MHz, DMSO-d_6) δ (ppm) 181.5, 175.6, 167.0, 165.3, 164.8, 152.5, 149.6, 147.4, 146.1, 140.0, 138.0, 136.6, 130.9, 130.2, 130.0, 128.6, 125.9, 125.3, 124.5, 123.8, 118.1, 117.1, 116.8, 115.0, 112.2, 111.5, 97.4, 70.4, 70.3, 70.2, 70.0, 69.3, 69.3, 68.6, 60.0, 44.6, 14.9; **HRMS** (m/z): calculated for $C_{55}H_{58}N_{10}O_{11}S$ [M+H]$^+$: 1067.4080, found: 1067.4096.

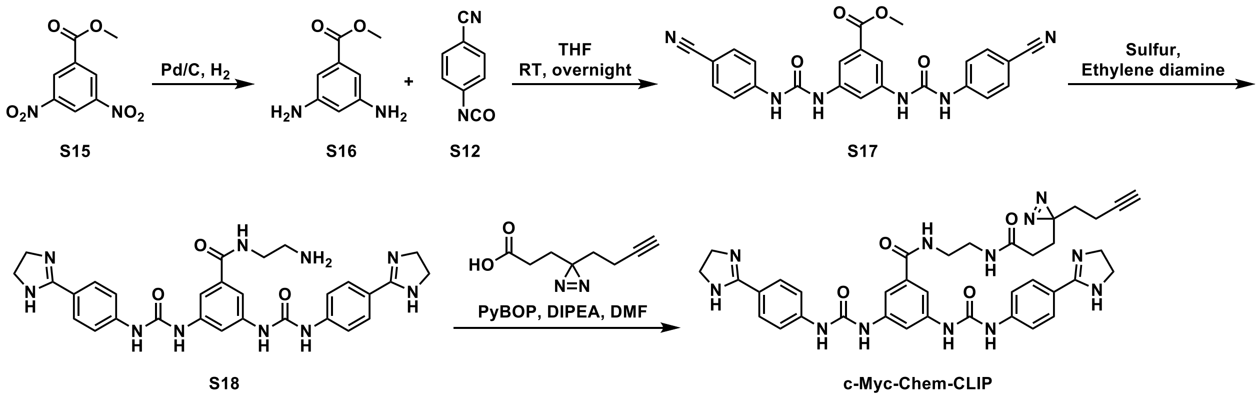

Scheme S11. Synthesis of c-Myc-Chem-CLIP.

Compound S17. Compound **S16** was synthesized as previously reported.[73] To a solution of **S16** (577 mg, 3.47 mmol) in THF, **S12** (1.00 g, 6.94 mmol) was added. The mixture was stirred overnight at room temperature. Then, the reaction mixture was filtered, and the precipitate **S17** was isolated as a white solid (1.48 g, 3.30 mmol, 95%). **^{1}H NMR** (400 MHz, DMSO-d_6) δ (ppm) 9.19 (d, *J* = 8.3 Hz, 4H), 7.82 (m, 3H), 7.75 (m, 4H), 7.65 (m, 4H), 3.86 (s, 3H); **^{13}C NMR** (150 MHz, DMSO-d_6) δ (ppm) 166.5, 152.5, 144.5, 140.5, 133.8, 131.1, 119.7, 118.7, 113.4, 112.9, 103.9, 97.6, 52.7.

Compound S18. To a solution of **S19** (300 mg, 0.66 mmol) in ethylenediamine (2 mL) was added sulfur (31.8 mg, 0.99 mmol), and the mixture was microwaved with stirring at 80 °C for 3 h and then at 70 °C for 90 min. The product was precipitated by addition of 5 mL of distilled water and collected by filtration to give **S18** as a yellow solid (198 mg, 0.35 mmol, 53%). **S18** was used in the next reaction without further purification. **^{1}H NMR** (400 MHz, DMSO-d_6) δ (ppm) 9.32 (m, *J* = 20.0 Hz, 3H), 8.35 (t, *J* = 5.3 Hz, 1H), 7.74 (m, 5H), 7.57 (m, 5H), 3.61 (s, 8H), 3.30 (q, *J* = 6.0 Hz, 2H), 2.73 (t, *J* = 6.4 Hz, 2H), 2.39 (s, 1H); **^{13}C NMR** (150 MHz, DMSO-d_6) δ (ppm) 167.4, 163.9, 152.9, 145.0, 142.6, 140.5, 136.5, 133.6, 128.5, 123.3, 118.6, 117.8, 111.9, 111.7, 111.0, 49.4, 42.3, 41.4, 41.2; **HRMS** (m/z): calculated for $C_{29}H_{32}N_{10}O_3$ $[M+H]^+$: 569.2659, found: 569.2653.

c-Myc-Chem-CLIP. To a solution of 3-(3-(but-3-yn-1-yl)-3H-diazirin-3-yl)propanoic acid (5 mg, 0.03 mmol) in DMF (500 µL), PyBOP (15.7 mg, 0.03 mmol) and DIPEA (19.3 µL, 0.12 mmol) were added, and the mixture was stirred at room temperature for 10 min. Then, **S18** (21.8 mg, 0.039 mmol) was added, and the reaction was stirred at room temperature for 2 h. After confirming the reaction had reached completion by LC-MS, the reaction mixture was purified by HPLC (MeOH 20-100% in H_2O + 0.1% (v/v) TFA) to afford **c-Myc-Chem-CLIP** (4.5 mg, 20.9% yield). **^{1}H NMR** (400 MHz, MeOD) δ (ppm) 7.80 (m, 9H), 7.61 (d, J = 2.0 Hz, 2H), 4.06 (s, 8H), 3.50 (m, 2H), 3.41 (t, J = 5.5 Hz, 2H), 2.23 (t, J = 5.9 Hz, 1H), 2.04 (m, 2H), 1.96 (m, 2H), 1.75 (m, 2H), 1.57 (t, J = 7.5 Hz, 2H); **^{13}C NMR** (100 MHz, MeOD) δ (ppm) 173.6, 168.9, 165.8, 152.9, 145.9, 139.6, 135.8, 129.2, 118.2, 114.9, 112.7, 112.6, 82.2 ,69.3, 68.9, 44.4, 39.5, 38.6, 31.9, 29.7, 28.4, 27.5, 12.4; **HRMS** (m/z): calculated for $C_{37}H_{40}N_{12}O_4$ $[M+H]^+$: 717.3368, found: 717.3361.

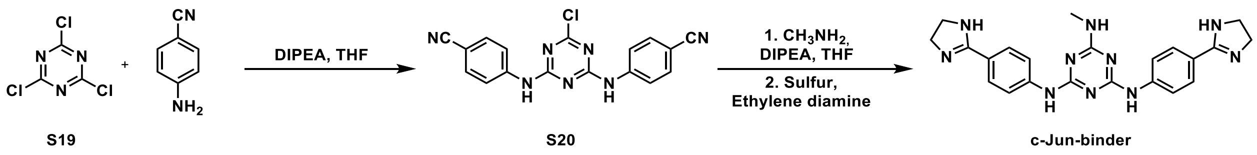

Scheme S12. Synthesis of c-Jun-binder.

Compound S20. Cyanuric chloride **S19** (500 mg, 2.7 mmol) and 4-amino benzonitrile (800 mg, 6.6 mmol) were dissolved in 2.5 mL THF, followed by the addition of DIPEA (1.6 mL, 9.4 mmol). The reaction was then heated to 60 °C with stirring for 1 h in a Biotage Initiator+ microwave reactor. The solvent was then evaporated under reduced pressure, and the product (560 mg, 53% yield) was purified by flash chromatography using Biotage Isolera one (40g silica column, 20-80% EtOAc-hexane) and isolated as a white powder. **^{1}H NMR** (600 MHz, DMSO-d_6) δ 7.86 (s, 2H), 7.43 – 7.35 (m, 4H), 6.64 – 6.58 (m, 4H). **^{13}C NMR** (151 MHz, DMSO-d_6) δ 153.5, 133.9, 133.6, 121.2, 113.9, 96.0.

c-Jun-binder. Compound **S20** (200 mg, 0.57 mmol) was dissolved in 2.5 mL of anhydrous THF followed by addition of DIPEA (213 µL, 1.15 mmol) in a 2 mL microwave vial. To the reaction mixture was added methylamine dissolved in THF (575 µL, 1.15 mmol) and the reaction was heated to 60 °C for 1 h in the microwave reactor. The solvent was then removed under reduced pressure. A portion of reaction (100 mg), without further purification, was dissolved in 2 mL of ethylene diamine followed by addition of sulfur (18 mg, 0.57 mmol). The reaction mixture was then heated at 90 °C for 1 h using a heat block while stirring. After, the solvent was removed under reduced pressure, and the product (55% yield) was obtained after purification by HPLC as described in the **General Methods**. **^{1}H NMR** (600 MHz, DMSO-d_6) δ 10.46 (s, 1H), 10.44 (s, 1H), 9.96 (s, 1H), 9.86 (s, 1H), 8.12 (d, J = 4.5 Hz, 4H), 7.92 (t, J = 9.0 Hz, 4H), 7.51 (s, 1H), 4.00 (s, 8H), 2.91 (d, J = 3.2 Hz, 3H). **^{13}C NMR** (151 MHz, DMSO-d_6) δ 166.23, 164.84 (d, J = 10.3 Hz), 164.48, 163.94, 146.59 (d, J = 8.9 Hz), 129.80, 119.42 (d, J = 25.6 Hz), 117.56, 115.62, 114.58 (d, J = 21.0 Hz), 49.00, 27.77. **HR-MS** (m/z): calculated for $C_{22}H_{24}N_{10}$ $[M+H]^+$: 429.2258, found: 429.2250.

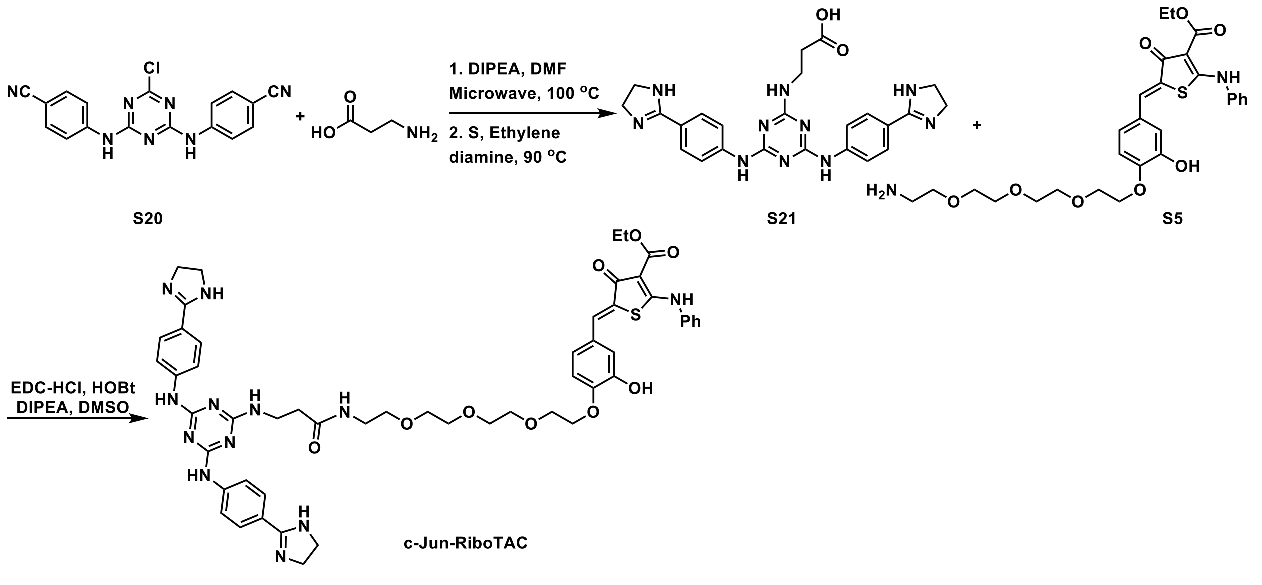

Scheme S13. Synthesis of c-Jun-RiboTAC.

Compound S21. Compound **S20** (100 mg, 0.28 mmol) was dissolved in anhydrous DMF followed by addition of DIPEA (158 µL, 0.86 mmol) in a 2 mL microwave vial. To the reaction mixture was added β-alanine (77 mg, 0.86 mmol), and the reaction was heated to 100 °C for 1 h in a Biotage Initiator+ microwave reactor. After removing the solvent under reduced pressure, without further purification, the reaction was added with 2 mL of ethylene diamine followed by addition of with sulfur (18 mg, 0.57 mmol). The reaction mixture was then heated at 90 °C for 1 h using a heat block while stirring. The solvent was removed under reduced pressure, and compound **S21** (49% yield) was obtained after purification by HPLC as described in the **General Methods**. **^{1}H NMR** (600 MHz, DMSO-d_6) δ 10.48 (d, J = 14.9 Hz, 1H), 10.39 (d, J = 10.8 Hz, 10H), 9.93 (s, 3H), 9.83 (s, 3H), 8.11 (dd, J = 8.1, 5.7 Hz, 11H), 7.89 (dd, J = 8.8, 5.1 Hz, 10H), 7.60 (s, 3H), 4.00 (d, J = 9.4 Hz, 20H), 3.58 (dd, J = 12.9, 7.0 Hz, 6H), 2.60 (t, J = 7.1 Hz, 5H); **^{13}C NMR** (151 MHz, DMSO-d_6) δ 173.42, 165.88, 164.83 (d, J = 13.1 Hz), 164.49, 164.16, 146.58 (d, J = 10.1 Hz), 129.81 (d, J = 5.2 Hz), 119.39 (d, J = 12.4 Hz), 117.92, 115.96, 114.65, 114.50, 49.05, 44.64 (d, J = 3.9 Hz), 36.97, 34.25, 31.15.

c-Jun-RiboTAC. **Compound S21** (60 mg, 0.12 mmol) was dissolved in 1 mL DMSO followed by addition of 1-ethyl-3-(3′-dimethylaminopropyl)carbodiimide·HCl (35.46 mg, 0.18 mmol), HOBt (25.18 mg, 0.18 mmol) and DIPEA (68.3 µL, 0.37 mmol). This solution was stirred for 15 min at room temperature followed by addition of **S5** (103.3 mg, 0.18 mmol). After stirring the reaction overnight at room temperature, the reaction mixture was purified by HPLC as described in the **General Methods** to obtain **c-Jun-RiboTAC** (23.6 mg, 19% yield). **^{1}H NMR** (600 MHz, DMSO-d_6): δ (ppm) 11.24 (s, 1H), 10.42-10.23 (m, 4H), 9.90 (s, 1H), 9.80 (s, 1H), 9.70-9.22 (br, 1H), 8.10 (d, *J* = 8.5 Hz, 4H), 7.98 (t, *J* = 5.6 Hz, 1H), 7.91-7.81 (4H), 7.56-7.49 (5H), 7.47 (s, 1H), 7.44 (m, 1H), 7.02-6.93 (3H), 4.27 (q, *J* = 7.1 Hz, 2H), 4.08 (m, 2H), 4.03-3.92 (m, 8H), 3.72 (m, 2H, overlapped with residual water peak), 3.59-3.52 (m, 4H), 3.52-3.44 (m, 6H), 3.39 (t, *J* = 5.9 Hz, 2H), 3.21 (q, *J* = 5.8 Hz, 2H), 2.44 (t, *J* = 7.1 Hz, 2H), 1.29 (t, *J* = 7.1 Hz, 3H); **^{13}C NMR** (150 MHz, DMSO-d_6): δ (ppm) 181.0, 175.3, 170.5, 165.4, 164.9, 164.4, 164.3, 164.1, 163.7, 148.7, 147.0, 146.2, 146.1, 137.5, 130.1, 129.6, 129.4, 129.3, 128.2, 126.3, 125.6, 124.7, 123.1, 119.0, 118.9, 115.8, 114.2, 114.0, 113.7, 96.9, 69.9, 69.8, 69.7, 69.6, 69.1, 68.9, 67.9, 59.6, 55.1, 44.2, 44.2, 40.1, 38.6, 37.0, 35.1, 14.4; **HRMS** (m/z): calculated for $C_{52}H_{59}N_{12}O_9S^+$ $[M+H]^+$: 1027.4243, found: 1027.4264.

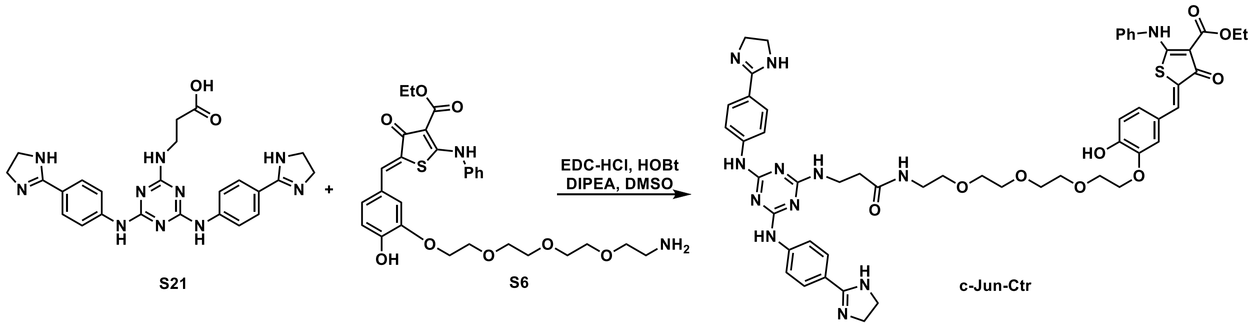

Scheme S14. Synthesis of c-Jun-Ctr.

c-Jun-Ctr. **Compound S21** (5 mg, 10.2 µmol) was dissolved in 200 µL DMSO followed by addition of DC-HCl (3.0 mg, 15.4 µmol), HOBt (2.1 mg, 15.4 µmol) and DIPEA (5.7 µL, 30.8 µmol), and the mixture was stirred for 15 min at room temperature. To the reaction was added compound **S6** (8.6 mg, 15.4

μmol), and the mixture was stirred overnight at room temperature. The reaction mixture was purified by HPLC as described in the **General Methods** to obtain **c-Jun-Ctr** (1.5 mg, 14% yield). **^{1}H NMR** (600 MHz, DMSO-d_6) δ (ppm) 11.23 (s, 1H), 10.35-10.22 (m, 4H), 9.89 (s, 1H), 9.87-9.69 (m, 2H), 8.10 (d, *J* = 7.8 Hz, 4H), 7.97 (t, *J* = 5.6 Hz, 1H), 7.91-7.80 (m, 4H), 7.58-7.48 (m, 6H), 7.43 (m, 1H), 7.14 (d, *J* = 2.0 Hz, 1H), 6.98 (dd, *J* = 8.3 Hz, 1.9, 1H), 6.88 (d, *J* = 8.3 Hz, 1H), 4.27 (q, *J* = 7.1 Hz, 2H), 4.06 (m, 2H), 4.02-3.93 (m, 8H), 3.69 (m, 2H), 3.59-3.35 (m, 14H, overlapped with residual water peak), 3.21 (m, 2H), 2.44 (t, *J* = 7.1 Hz, 2H), 1.29 (t, *J* = 7.1 Hz, 3H); **^{13}C NMR** (150 MHz, DMSO-d_6) δ (ppm) 181.0, 175.3, 170.5, 165.4, 164.9, 164.4, 164.3, 164.1, 163.7, 148.7, 147.0, 146.2, 146.1, 137.5, 130.1, 129.7, 129.4, 129.3, 128.2, 126.3, 125.6, 124.7, 123.1, 119.0, 118.9, 115.8, 114.2, 114.0, 113.7, 96.9, 69.9, 69.8, 69.7, 69.6, 69.1, 68.9, 67.9, 59.6, 44.2, 44.2, 40.1, 38.6, 37.0, 35.1, 14.4; **HRMS** (m/z): calculated for $C_{52}H_{59}N_{12}O_9S^+$ [M+H]$^+$: 1027.4243, found: 1027.4229.

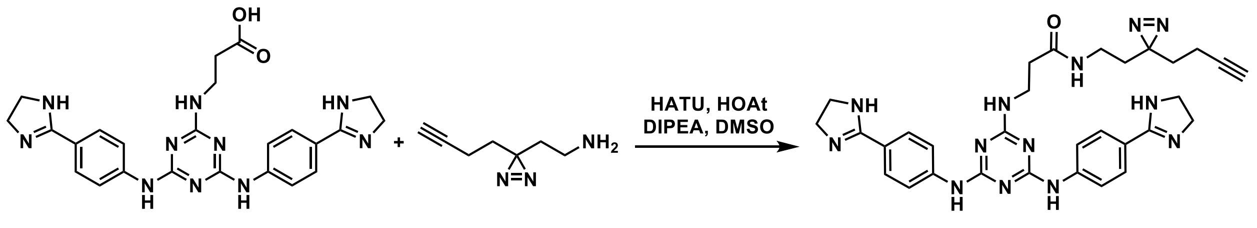

Scheme S15. Synthesis of c-Jun-Chem-CLIP.

c-Jun-Chem-CLIP. To **S21** (4.2 mg, 8.6 μmol) dissolved in 250 μL DMSO were added HATU (6.7 mg, 17.26 μmol), HOAt (2.35 mg, 17.26 μmol) and DIPEA (6.4 μL, 34.53 μmol). This solution was stirred for 15 min at room temperature after which 2-(3-(but-3-yn-1-yl)-3H-diazirin-3-yl)ethan-1-amine (2.37 mg, 17.26 μmol) was added to the solution and the reaction was stirred overnight at room temperature, protected from light. The reaction mixture was purified by HPLC as described in the **General Methods** to obtain **c-Jun-Chem-CLIP** (2.1 mg, 40% yield). **^{1}H NMR** (600 MHz, DMSO-d_6) δ (ppm) 10.33 (s, 2H), 9.91 (s, 1H), 9.80 (s, 1H), 8.11 (d, J = 8.6 Hz, 2H), 7.91 (dt, J = 16.3, 6.8 Hz, 3H), 7.52 (s, 1H), 3.99 (s, 4H), 3.57 (dd, J = 13.1, 6.8 Hz, 2H), 2.94 (dd, J = 12.9, 7.0 Hz, 1H), 2.82 (t, J = 2.6

Hz, 1H), 2.43 (t, J = 7.1 Hz, 1H), 1.97 (td, J = 7.4, 2.6 Hz, 1H), 1.58 (t, J = 7.4 Hz, 1H), 1.53 (t, J = 7.2 Hz, 1H); **^{13}C NMR** (151 MHz, DMSO-d_6) δ (ppm) 170.81, 165.89, 164.82 (d, J = 13.8 Hz), 164.50, 164.18, 146.66, 129.83 (d, J = 9.5 Hz), 119.40 (d, J = 18.2 Hz), 116.69, 114.59, 114.44, 83.60, 72.25, 44.63 (d, J = 4.9 Hz), 37.40, 35.63, 34.05, 32.44, 31.76, 27.69, 13.14. **HRMS** (m/z): calculated for $C_{31}H_{35}N_{13}O$ $[M+H]^+$: 606.3160, found: 606.3154.

NMR Spectra

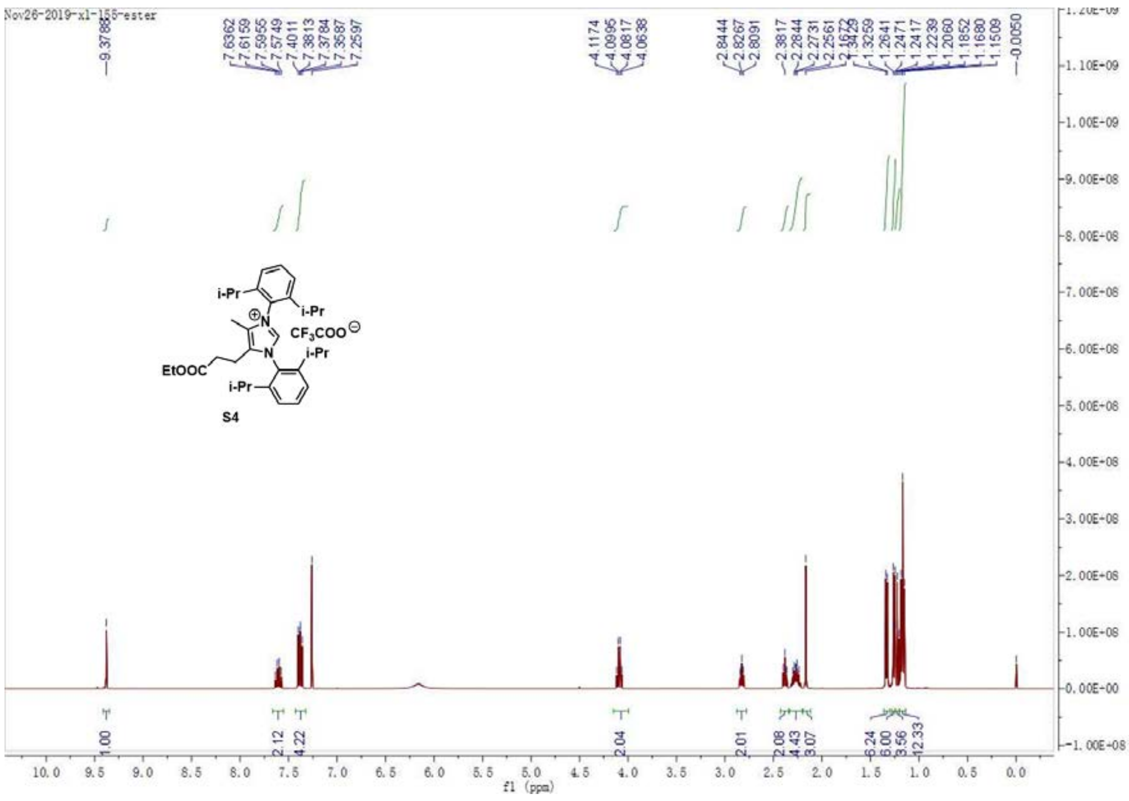
Nov26-2019-xl-155-ester
9.3785
7.6362
7.6159
7.5955
7.5749
7.4011
7.3813
7.3784
7.3587
7.2597
4.1174
4.0995
4.0817
4.0638
2.8444
2.8267
2.8091
2.3817
2.2844
2.2731
2.2561
2.1672
1.3429
1.3259
1.2641
1.2471
1.2417
1.2239
1.2060
1.1852
1.1680
1.1509
0.0050
1.00
2.12
4.22
2.04
2.01
2.08
4.43
3.07
6.24
6.00
3.56
12.33
i-Pr
i-Pr
$CF_3COO^{\ominus}$
i-Pr
EtOOC
i-Pr
S4
f1 (ppm)

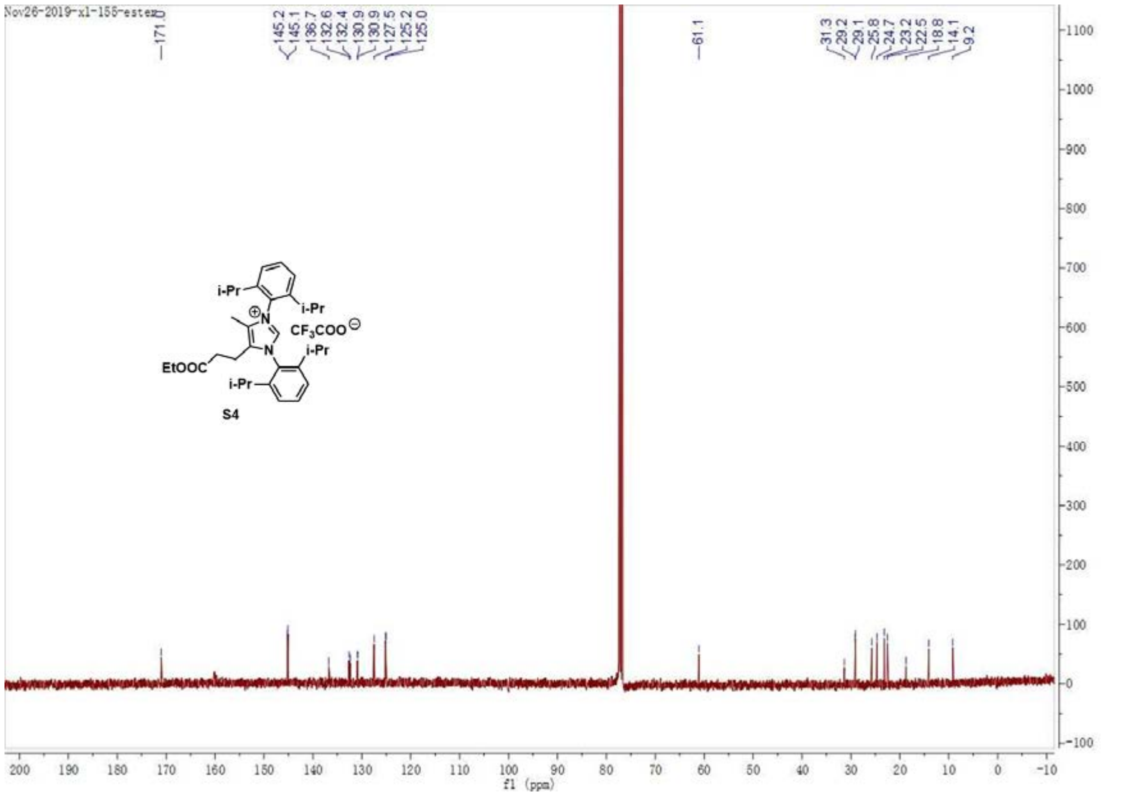
Nov26-2019-xl-155-ester
171.0
145.2
145.1
136.7
132.6
132.4
130.9
130.9
127.5
125.2
125.0
61.1
31.3
29.2
29.1
25.8
24.7
23.2
22.5
18.8
14.1
9.2
i-Pr
i-Pr
$CF_3COO^{\ominus}$
i-Pr
EtOOC
i-Pr
S4
f1 (ppm)

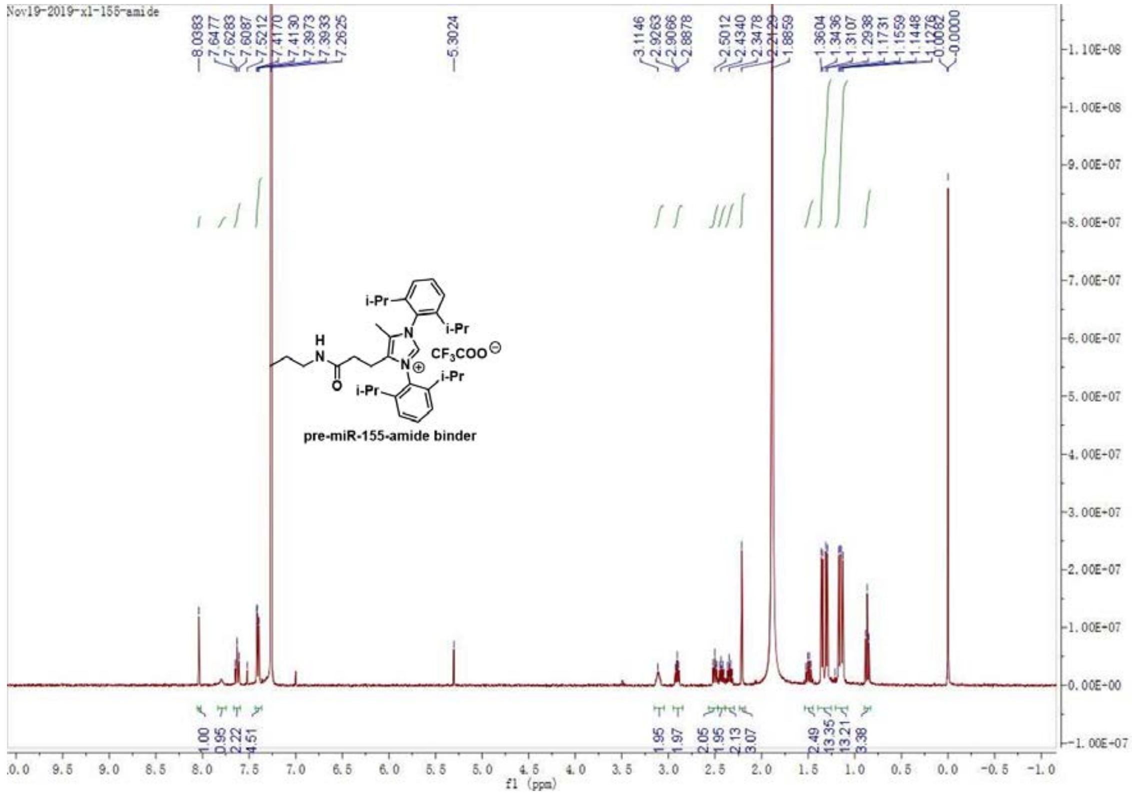
Nov19-2019-xl-155-amide
8.0383
7.6477
7.6283
7.6087
7.5212
7.4170
7.4130
7.3973
7.3933
7.2625
5.3024
3.1146
2.9263
2.9066
2.8878
2.5012
2.4340
2.3478
2.2129
1.8859
1.3604
1.3436
1.3107
1.2938
1.1731
1.1559
1.1448
1.1276
0.0000
pre-miR-155-amide binder
1.00
0.95
2.22
4.51
1.95
1.97
2.05
1.95
2.13
3.07
2.49
13.35
13.21
3.38
f1 (ppm)

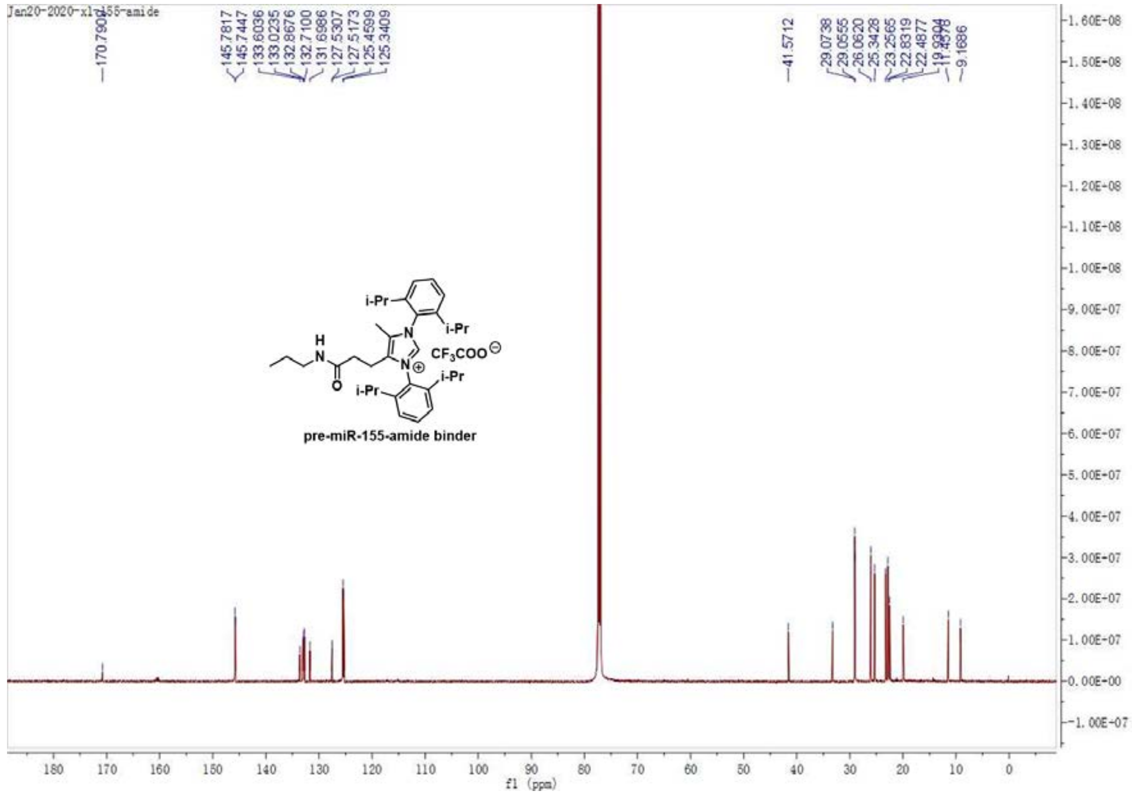
Jan20-2020-xl-155-amide
170.7900
145.7817
145.7447
133.6036
133.0235
132.8676
132.7100
131.6986
127.5307
127.5173
125.4599
125.3409
41.5712
29.0738
29.0555
26.0620
25.3428
23.2565
22.8319
22.4877
11.4578
9.1686
pre-miR-155-amide binder
f1 (ppm)

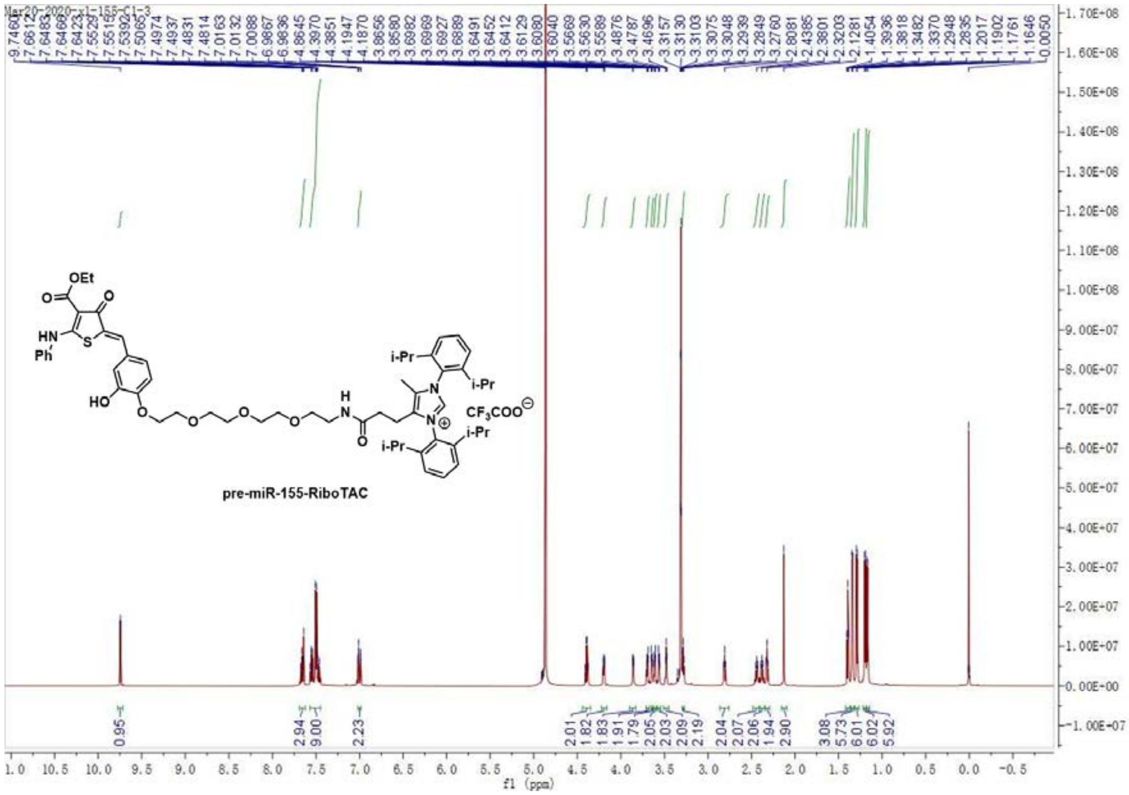
Mar20-2020-x1-155-C1-3
pre-miR-155-RiboTAC
OEt
HN
Ph
S
HO
H
N
i-Pr
CF3COO
f1 (ppm)

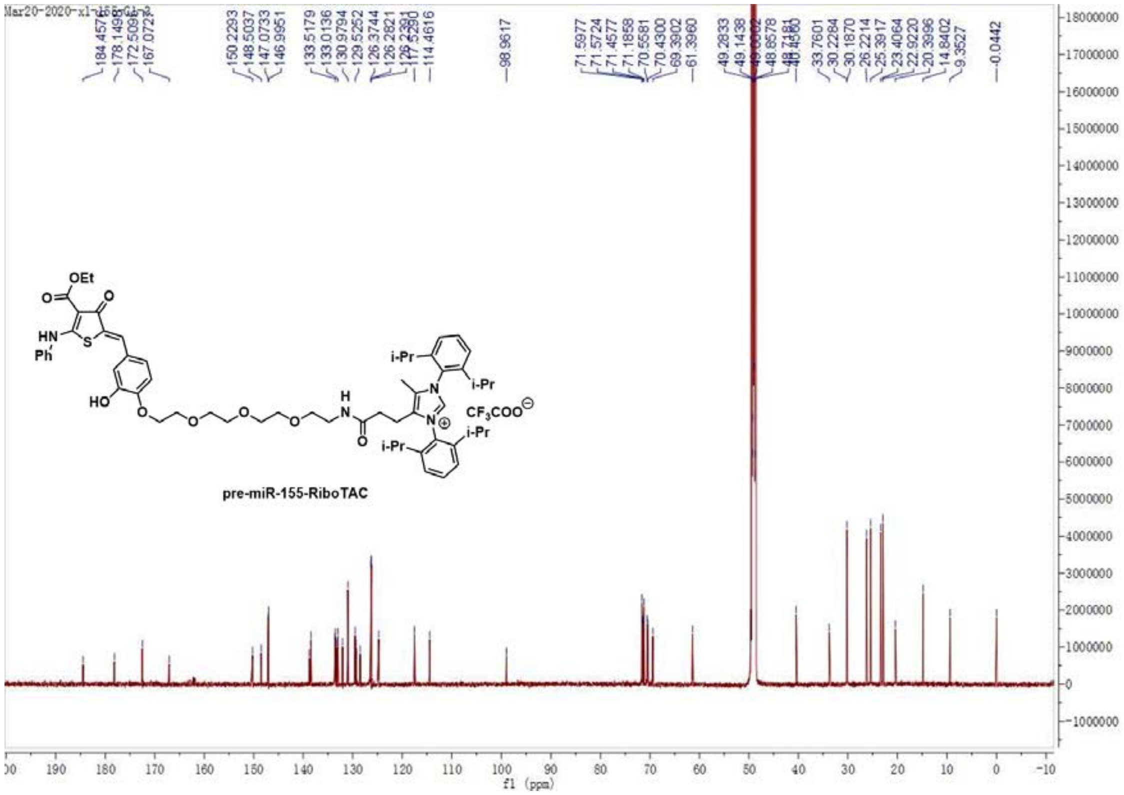
pre-miR-155-RiboTAC
OEt
HN
Ph
S
HO
H
N
i-Pr
CF3COO
f1 (ppm)

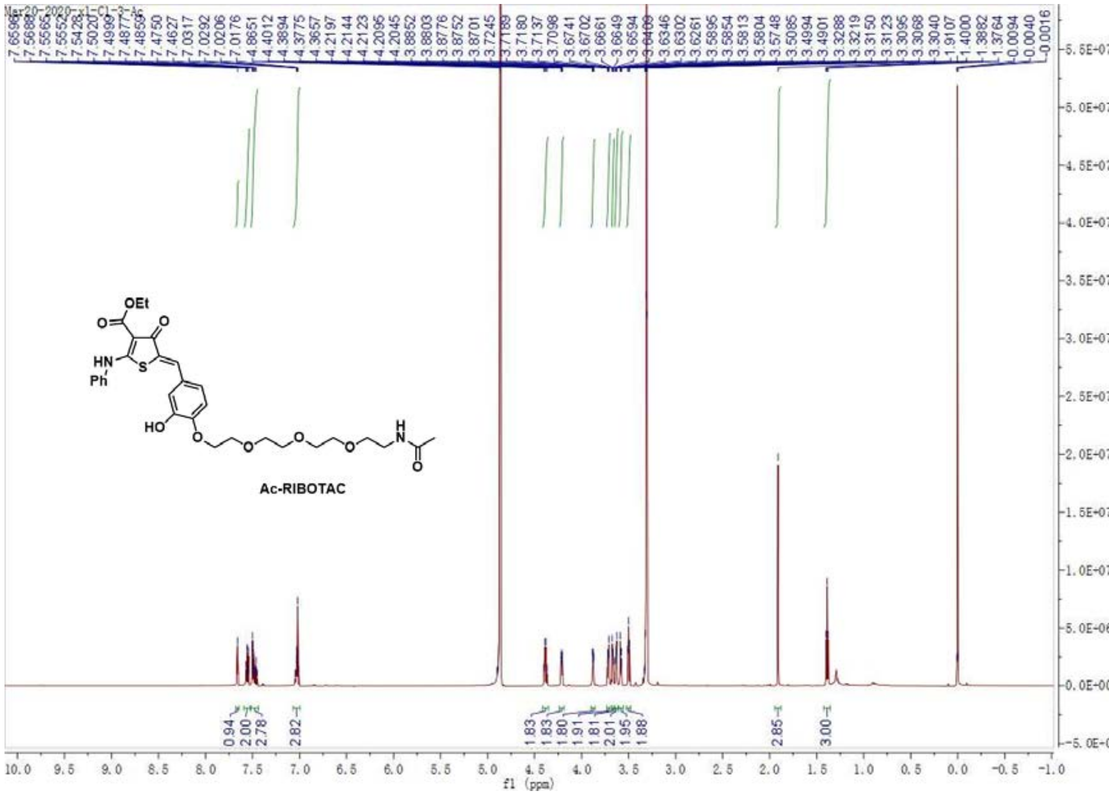

Mar20-2020-x1-C1-3-Ac
Ac-RIBOTAC
f1 (ppm)

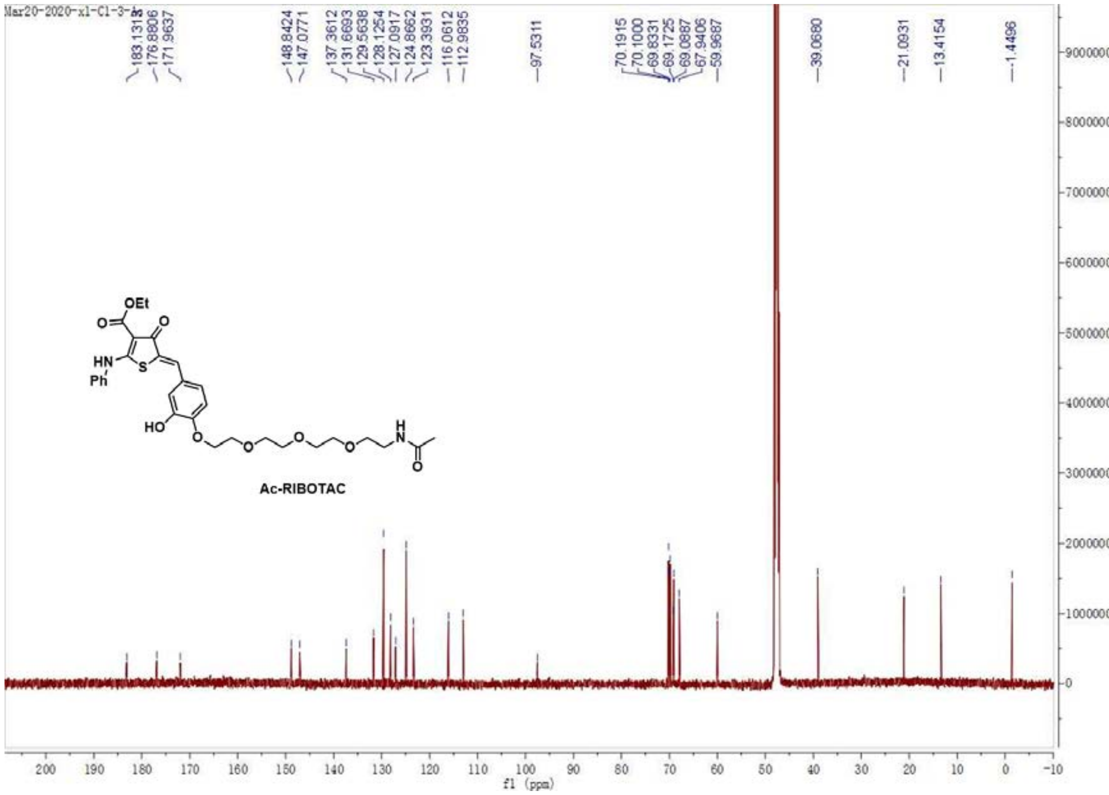

Mar20-2020-x1-C1-3-Ac
Ac-RIBOTAC
f1 (ppm)

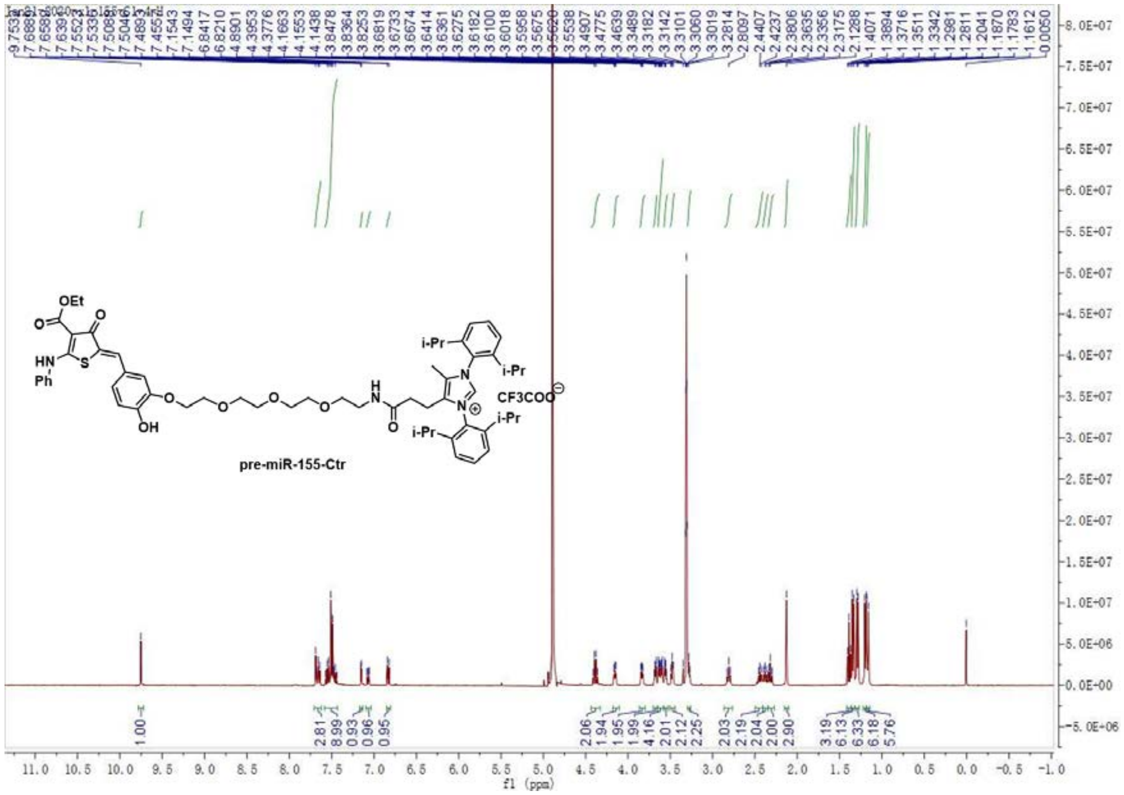

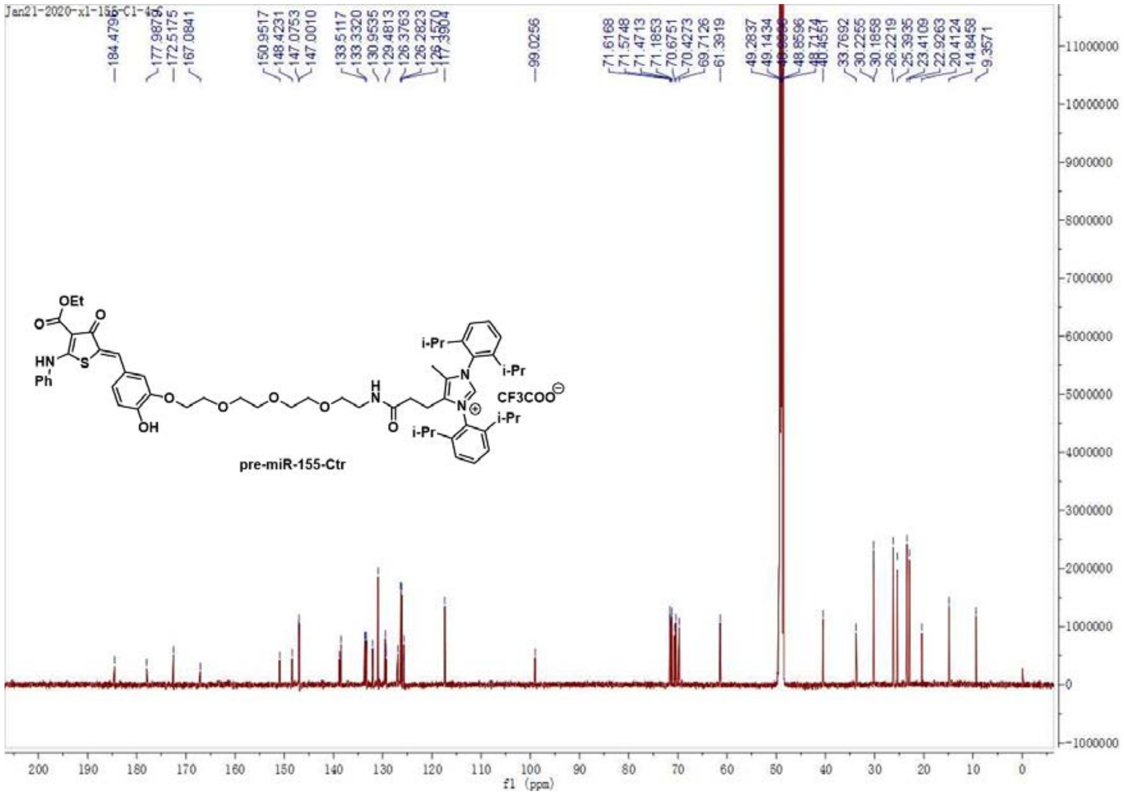

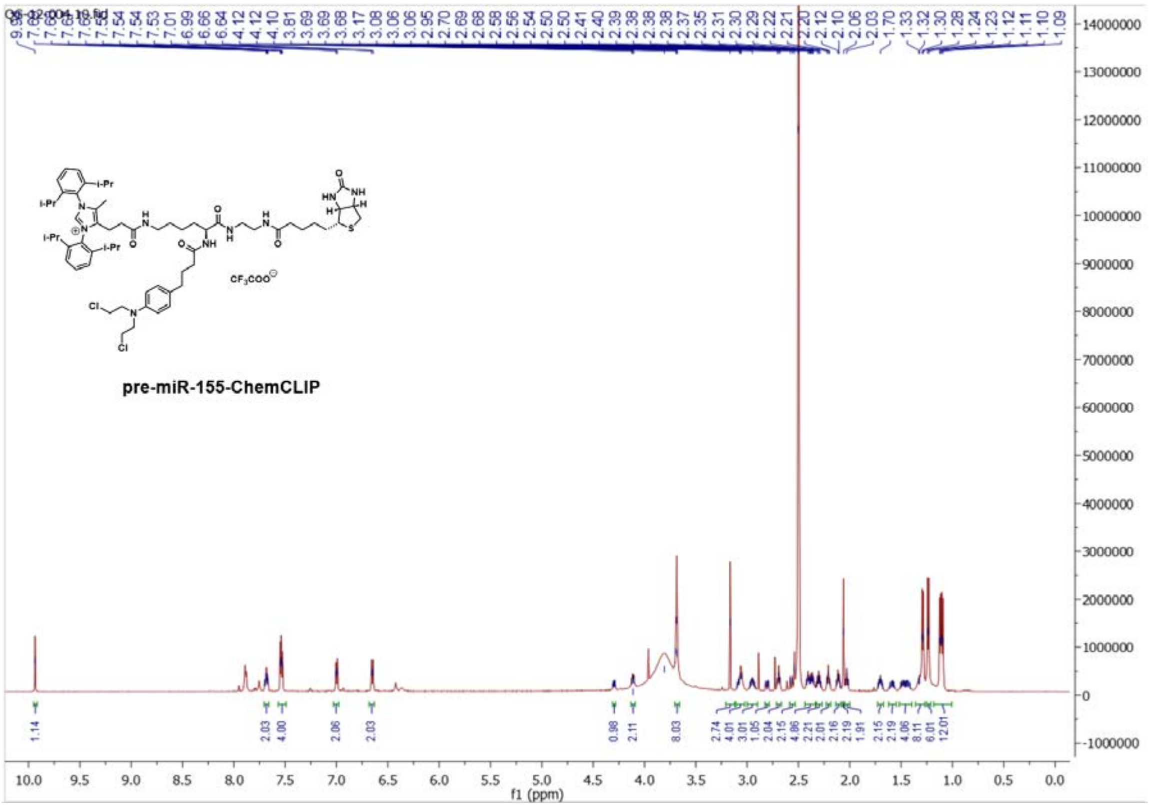

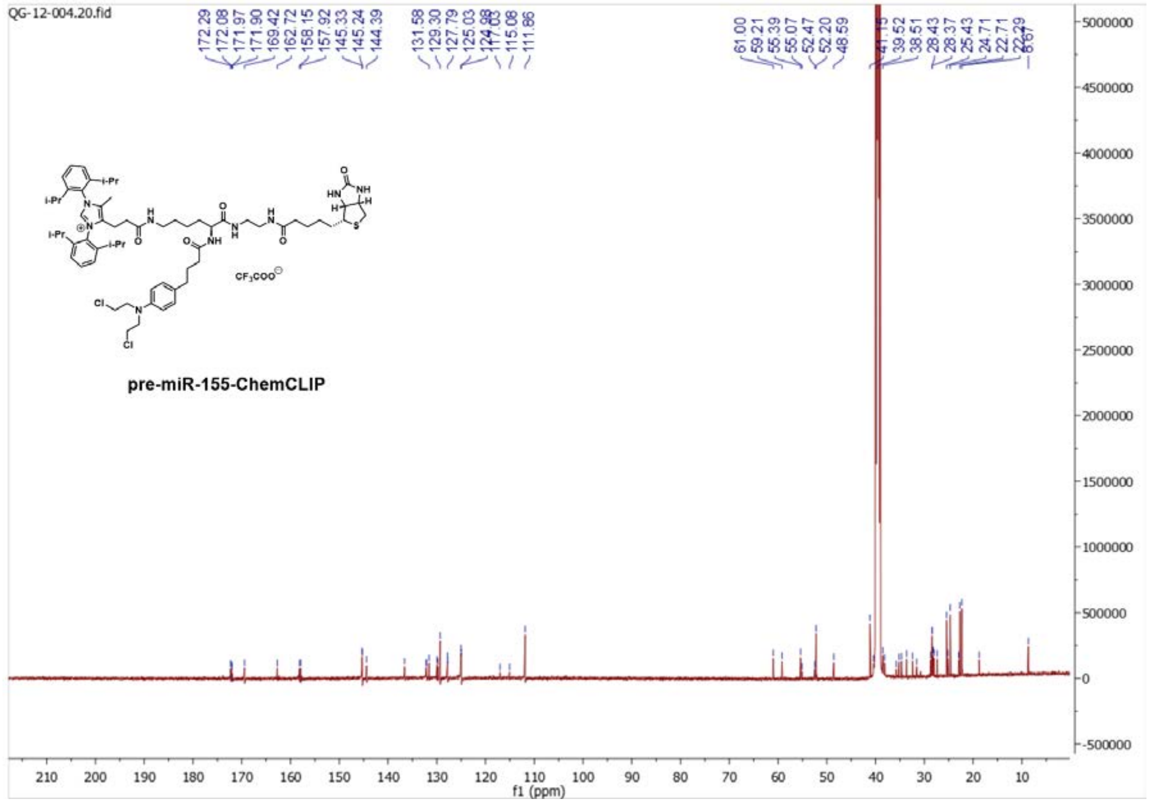

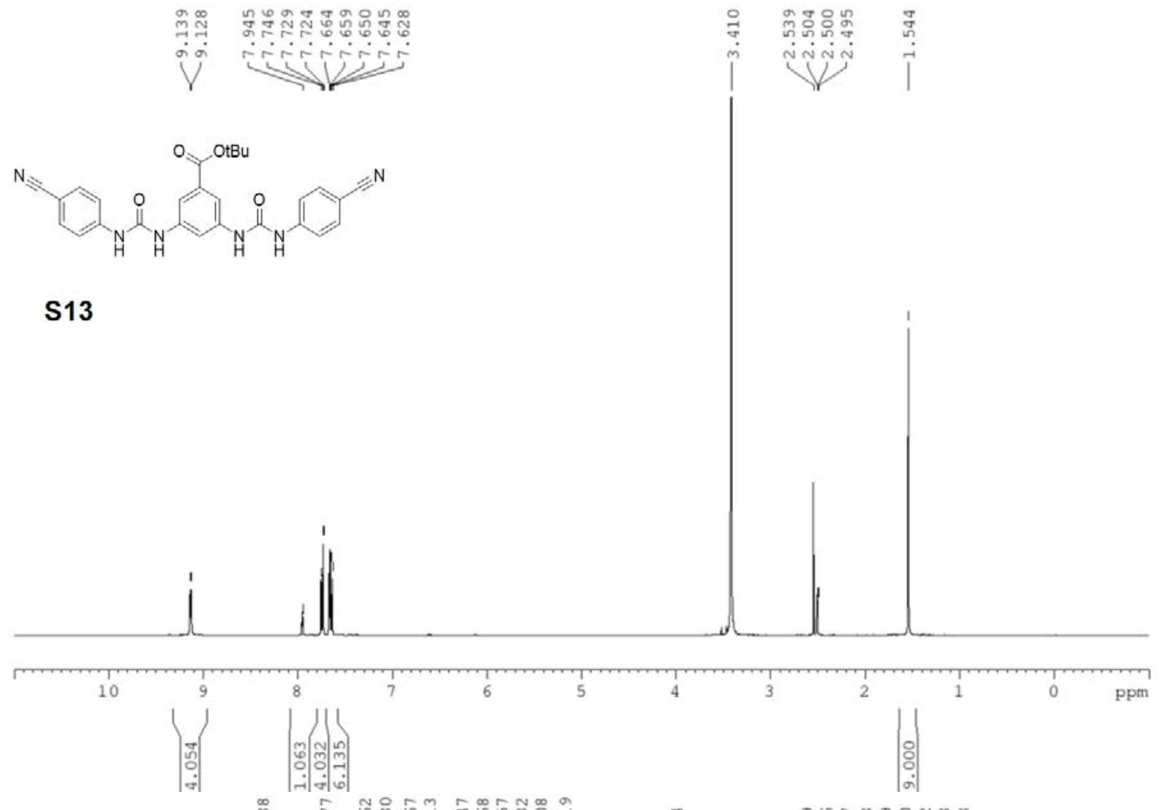
9.139
9.128
7.945
7.746
7.729
7.724
7.664
7.659
7.650
7.645
7.628
3.410
2.539
2.504
2.500
2.495
1.544
S13
ppm
4.054
1.063
4.032
6.135
9.000

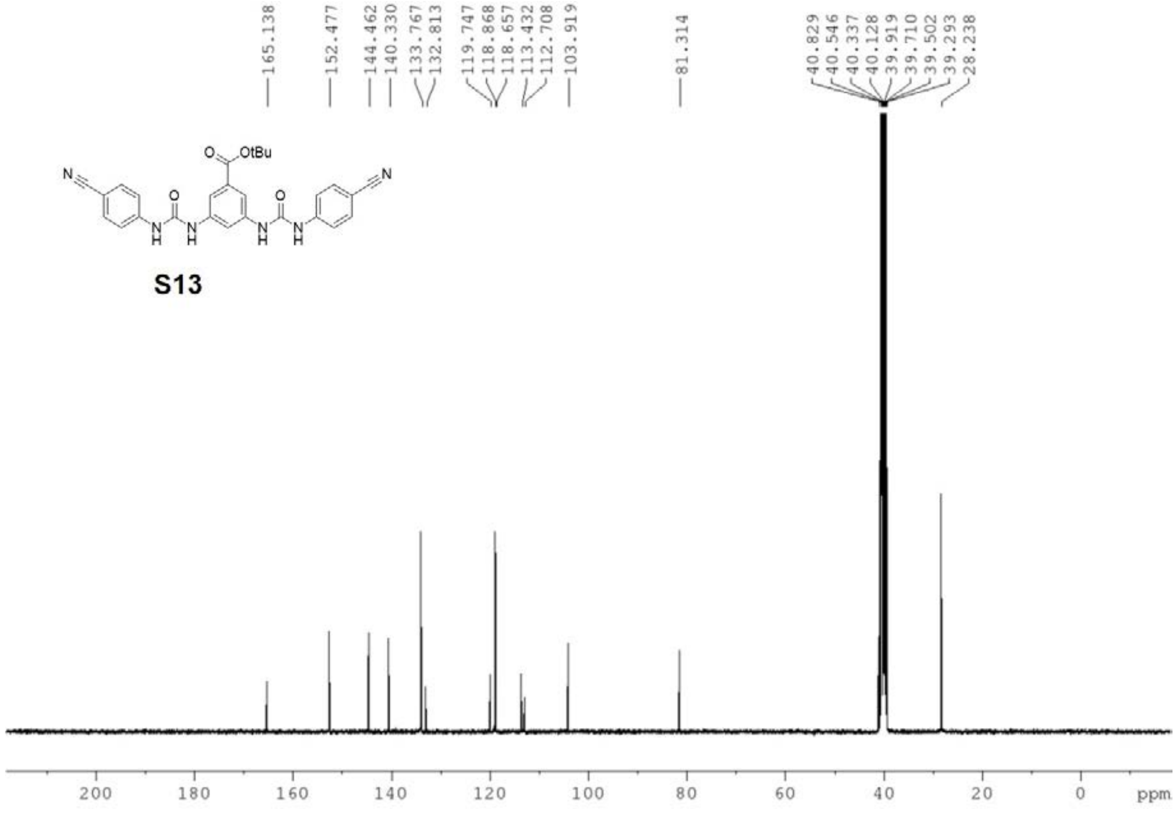
165.138
152.477
144.462
140.330
133.767
132.813
119.747
118.868
118.657
113.432
112.708
103.919
81.314
40.829
40.546
40.337
40.128
39.919
39.710
39.502
39.293
28.238
S13
ppm

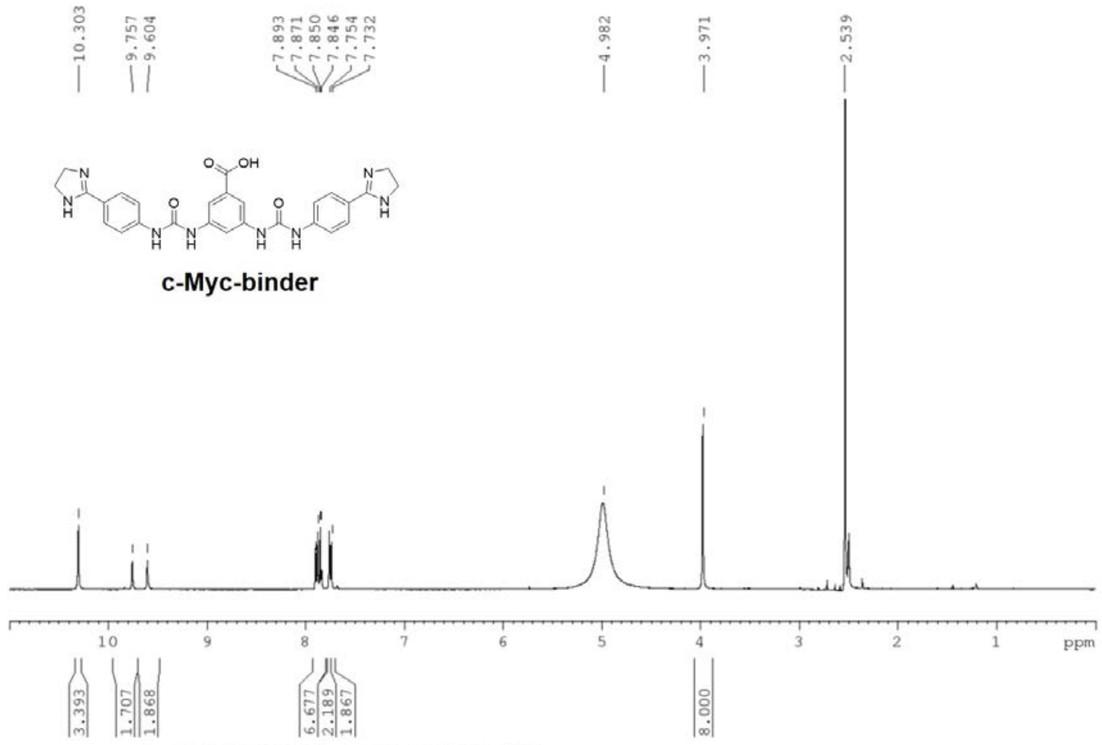
10.303
9.757
9.604
7.893
7.871
7.850
7.846
7.754
7.732
4.982
3.971
2.539
c-Myc-binder
ppm
3.393
1.707
1.868
6.677
2.189
1.867
8.000

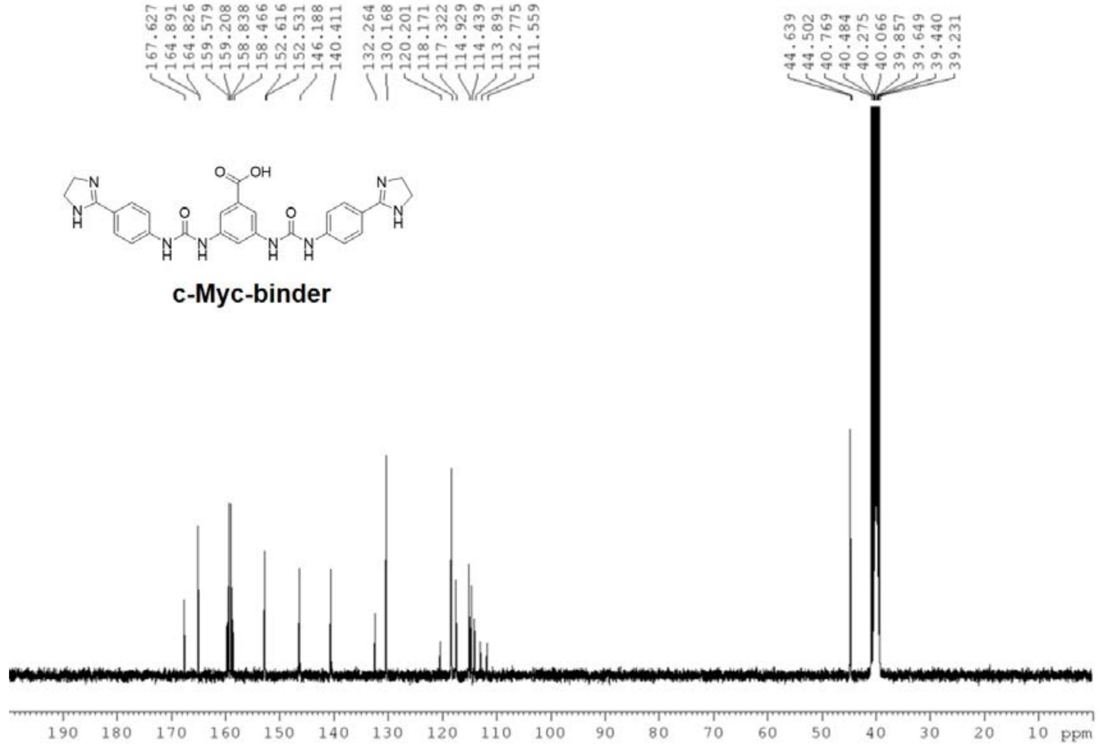
167.627
164.891
164.826
159.579
159.208
158.838
158.466
152.616
152.531
146.188
140.411
132.264
130.168
120.201
118.171
117.322
114.929
114.439
113.891
112.775
111.559
44.639
44.502
40.769
40.484
40.275
40.066
39.857
39.649
39.440
39.231
c-Myc-binder
ppm

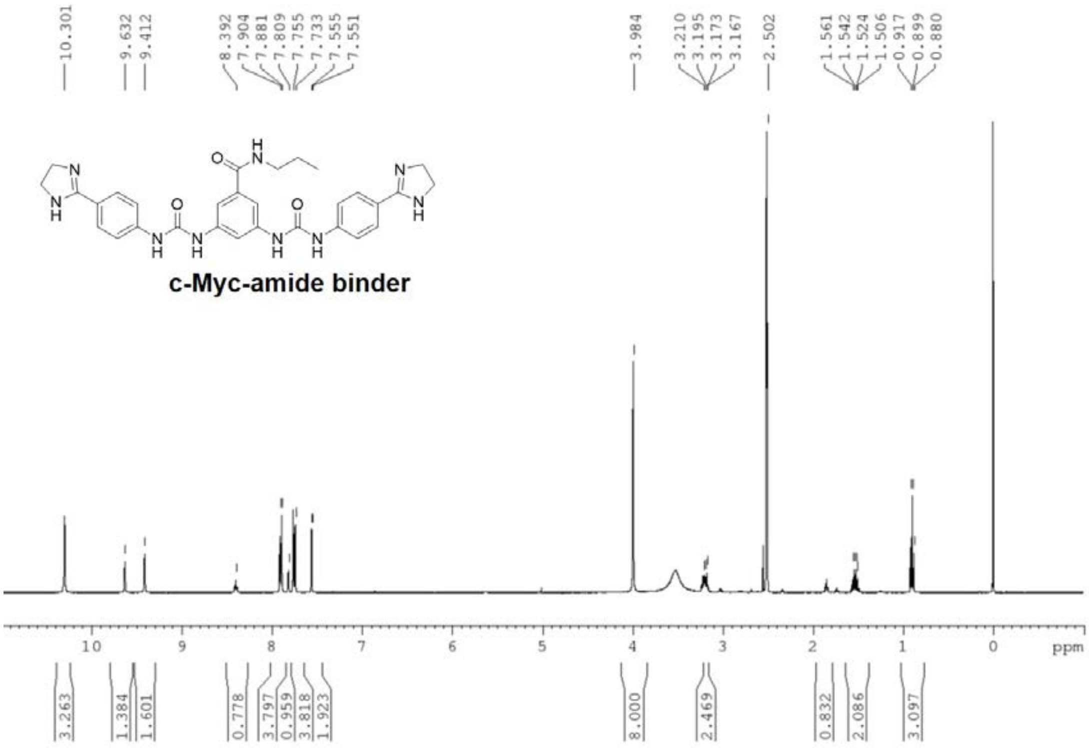
10.301
9.632
9.412
8.392
7.904
7.881
7.809
7.755
7.733
7.555
7.551
3.984
3.210
3.195
3.173
3.167
2.502
1.561
1.542
1.524
1.506
0.917
0.899
0.880
c-Myc-amide binder
10 9 8 7 6 5 4 3 2 1 0 ppm
3.263
1.384
1.601
0.778
3.797
0.959
3.818
1.923
8.000
2.469
0.832
2.086
3.097

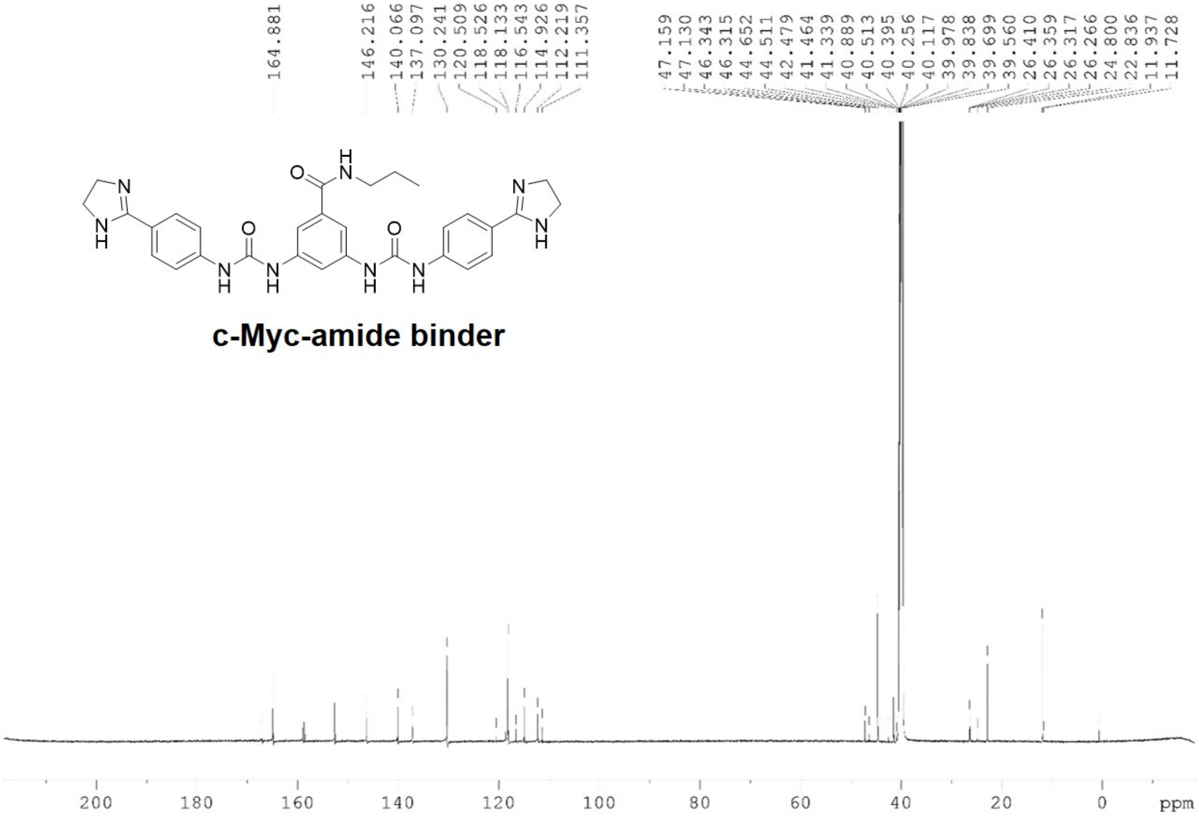
164.881
146.216
140.066
137.097
130.241
120.509
118.526
118.133
116.543
114.926
112.219
111.357
47.159
47.130
46.343
46.315
44.652
44.511
42.479
41.464
41.339
40.889
40.513
40.395
40.256
40.117
39.978
39.838
39.699
39.560
26.410
26.359
26.317
26.266
24.800
22.836
11.937
11.728
c-Myc-amide binder
200 180 160 140 120 100 80 60 40 20 0 ppm

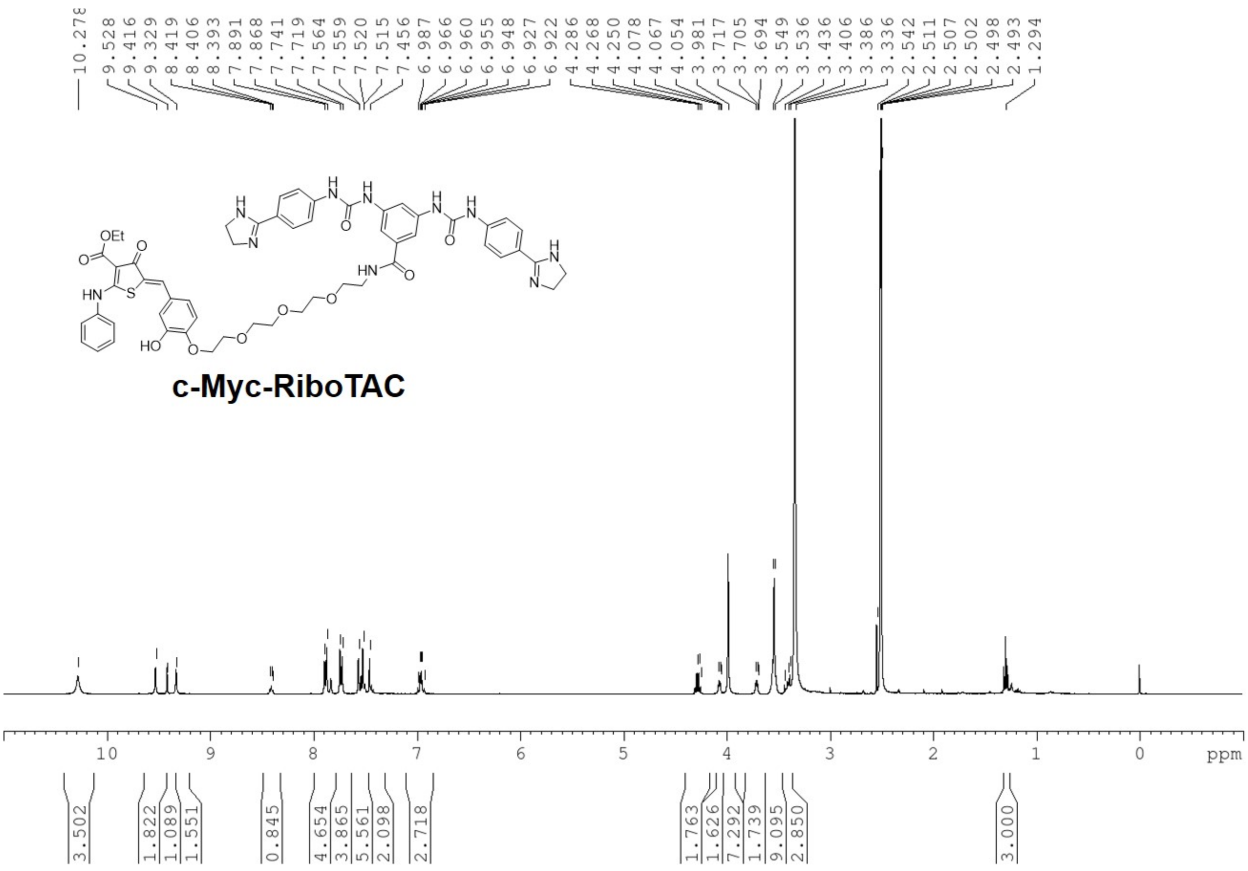
10.278
9.528
9.416
9.329
8.419
8.406
8.393
7.891
7.868
7.741
7.719
7.564
7.559
7.520
7.515
7.456
6.987
6.966
6.960
6.955
6.948
6.927
6.922
4.286
4.268
4.250
4.078
4.067
4.054
3.981
3.717
3.705
3.694
3.549
3.536
3.436
3.406
3.386
3.336
2.542
2.511
2.507
2.502
2.498
2.493
1.294
OEt
O
HN
S
HO
H
N
O
HN
c-Myc-RiboTAC
10
9
8
7
6
5
4
3
2
1
0
ppm
3.502
1.822
1.089
1.551
0.845
4.654
3.865
5.561
2.098
2.718
1.763
1.626
7.292
1.739
9.095
2.850
3.000

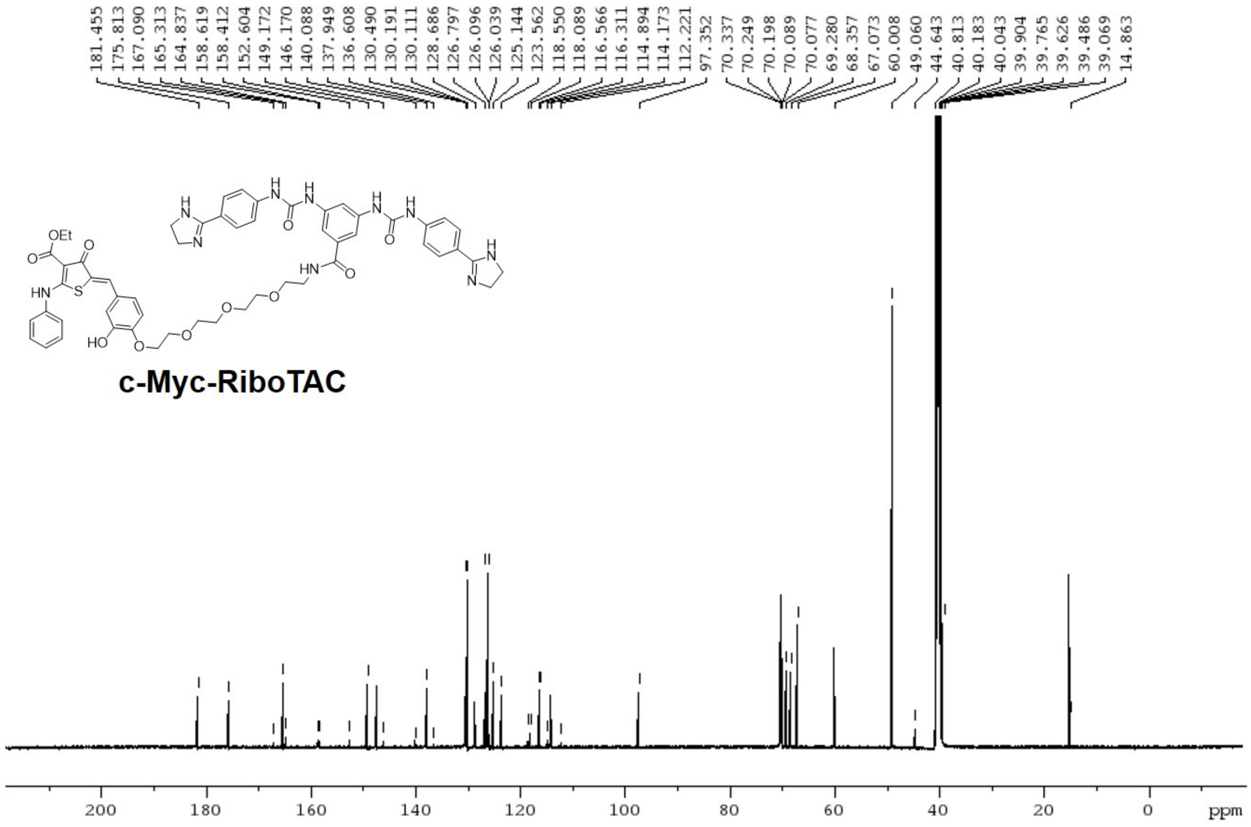
181.455
175.813
167.090
165.313
164.837
158.619
158.412
152.604
149.172
146.170
140.088
137.949
136.608
130.490
130.191
130.111
128.686
126.797
126.096
126.039
125.144
123.562
118.550
118.089
116.566
116.311
114.894
114.173
112.221
97.352
70.337
70.249
70.198
70.089
70.077
69.280
68.357
67.073
60.008
49.060
44.643
40.813
40.183
40.043
39.904
39.765
39.626
39.486
39.069
14.863
OEt
O
HN
S
HO
H
N
O
HN
c-Myc-RiboTAC
200
180
160
140
120
100
80
60
40
20
0
ppm

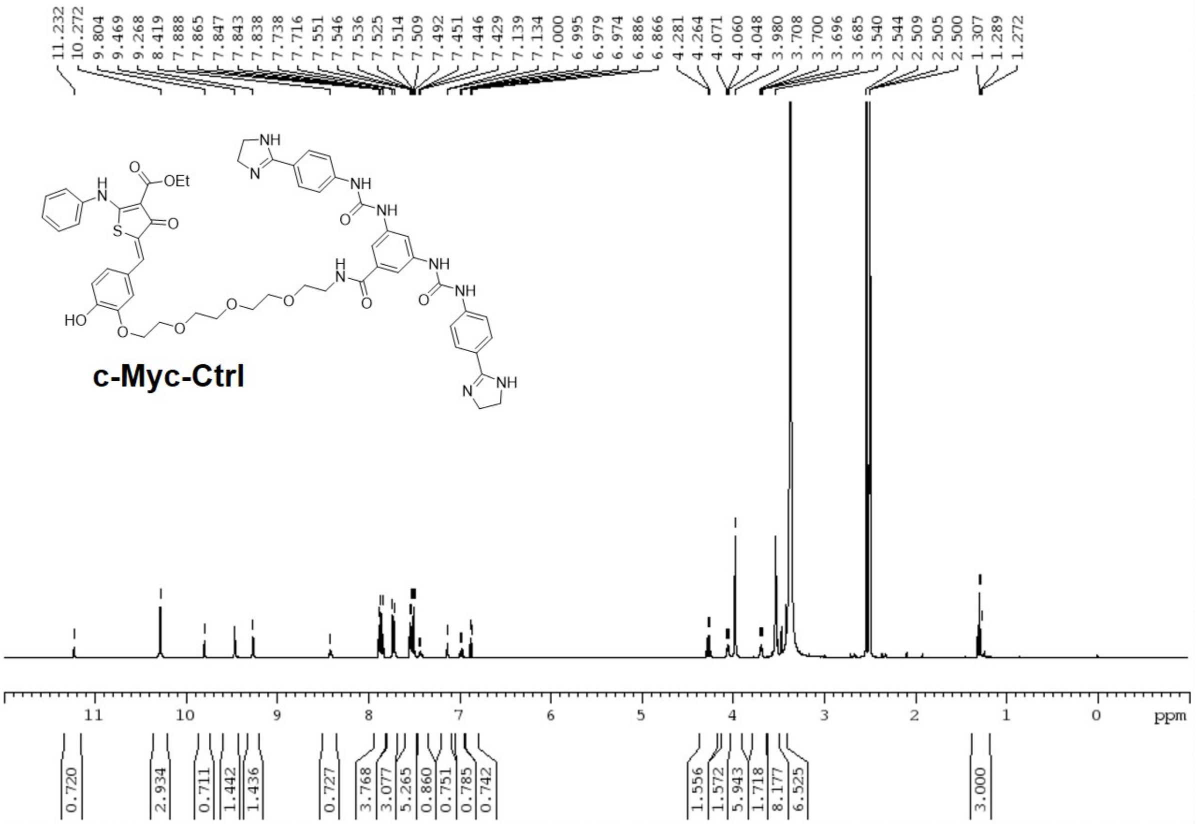
11.232
10.272
9.804
9.469
9.268
8.419
7.888
7.865
7.847
7.843
7.838
7.738
7.716
7.551
7.546
7.536
7.525
7.514
7.509
7.492
7.451
7.446
7.429
7.139
7.134
7.000
6.995
6.979
6.974
6.886
6.866
4.281
4.264
4.071
4.060
4.048
3.980
3.708
3.700
3.696
3.685
3.540
2.544
2.509
2.505
2.500
1.307
1.289
1.272
c-Myc-Ctrl
ppm
0.720
2.934
0.711
1.442
1.436
0.727
3.768
3.077
5.265
0.860
0.751
0.785
0.742
1.556
1.572
5.943
1.718
8.177
6.525
3.000
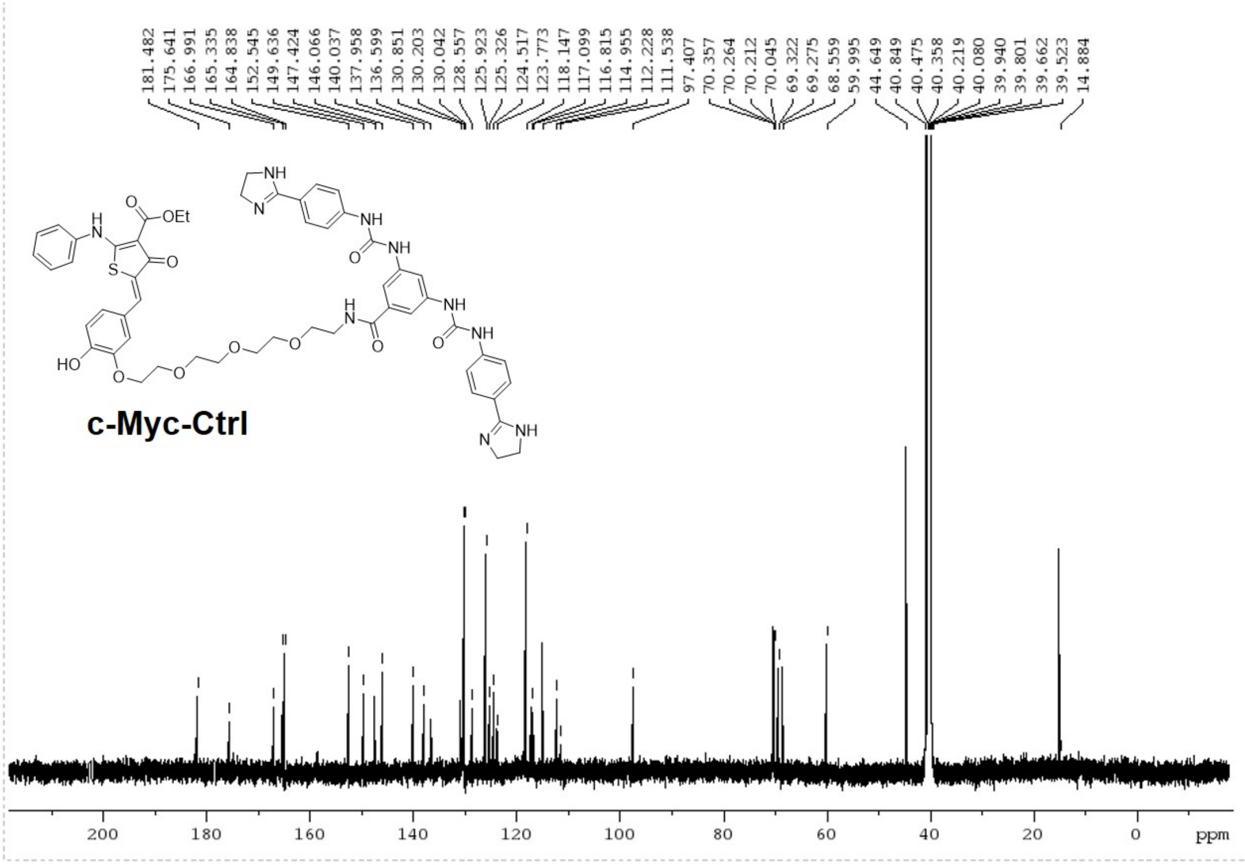
181.482
175.641
166.991
165.335
164.838
152.545
149.636
147.424
146.066
140.037
137.958
136.599
130.851
130.203
130.042
128.557
125.923
125.326
124.517
123.773
118.147
117.099
116.815
114.955
112.228
111.538
97.407
70.357
70.264
70.212
70.045
69.322
69.275
68.559
59.995
44.649
40.849
40.475
40.358
40.219
40.080
39.940
39.801
39.662
39.523
14.884
c-Myc-Ctrl
ppm

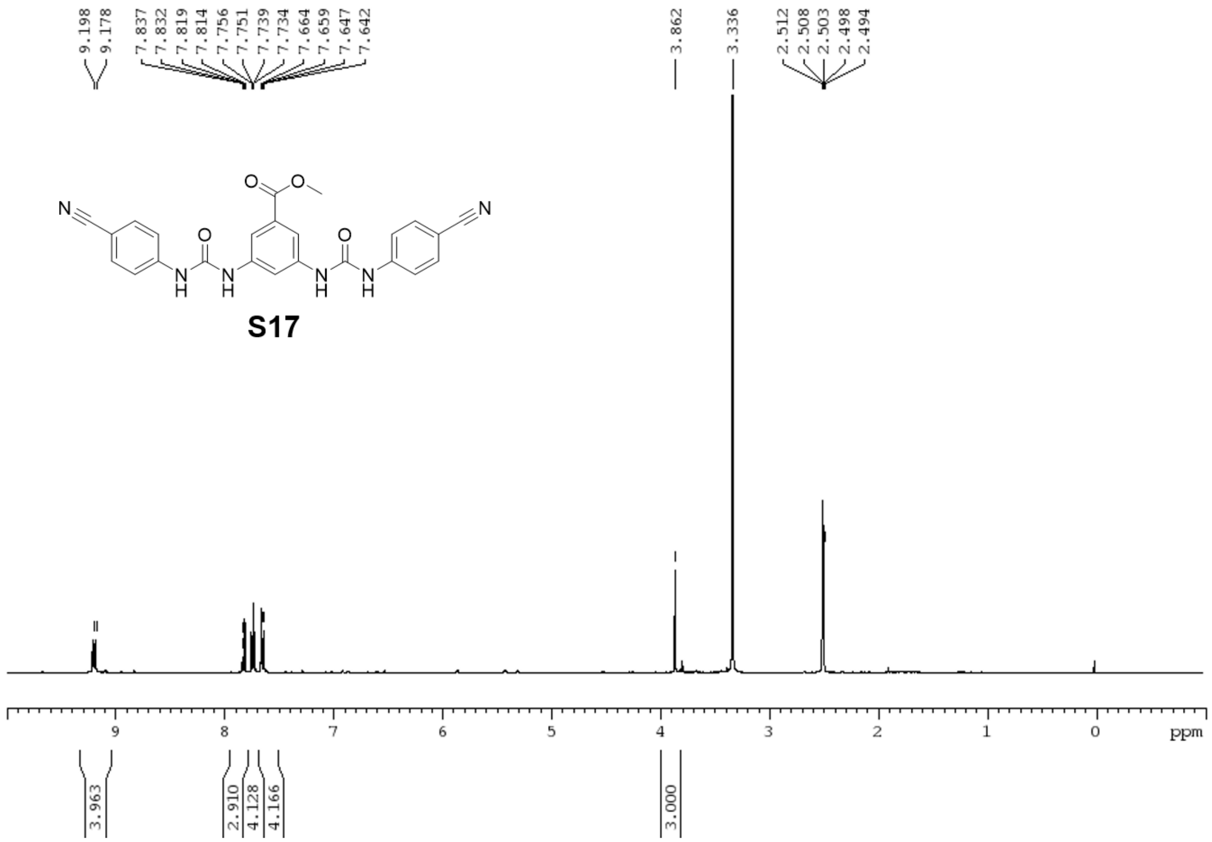
9.198
9.178
7.837
7.832
7.819
7.814
7.756
7.751
7.739
7.734
7.664
7.659
7.647
7.642
3.862
3.336
2.512
2.508
2.503
2.498
2.494
S17
ppm
3.963
2.910
4.128
4.166
3.000

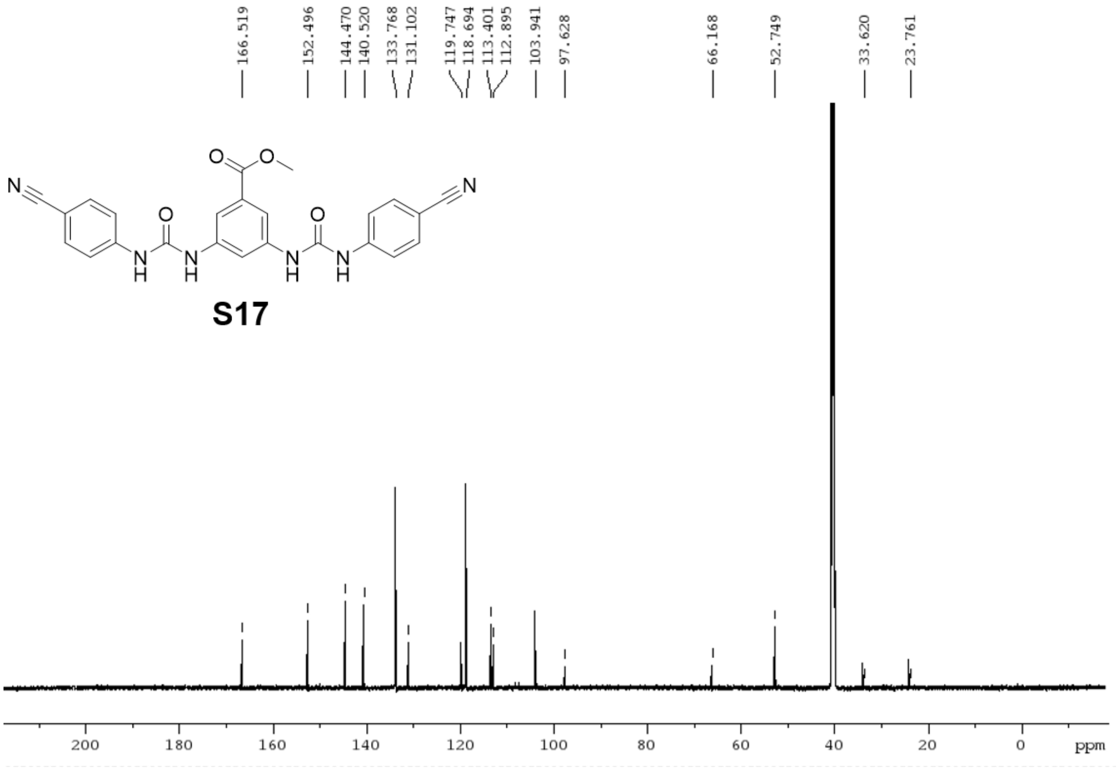
166.519
152.496
144.470
140.520
133.768
131.102
119.747
118.694
113.401
112.895
103.941
97.628
66.168
52.749
33.620
23.761
S17
ppm

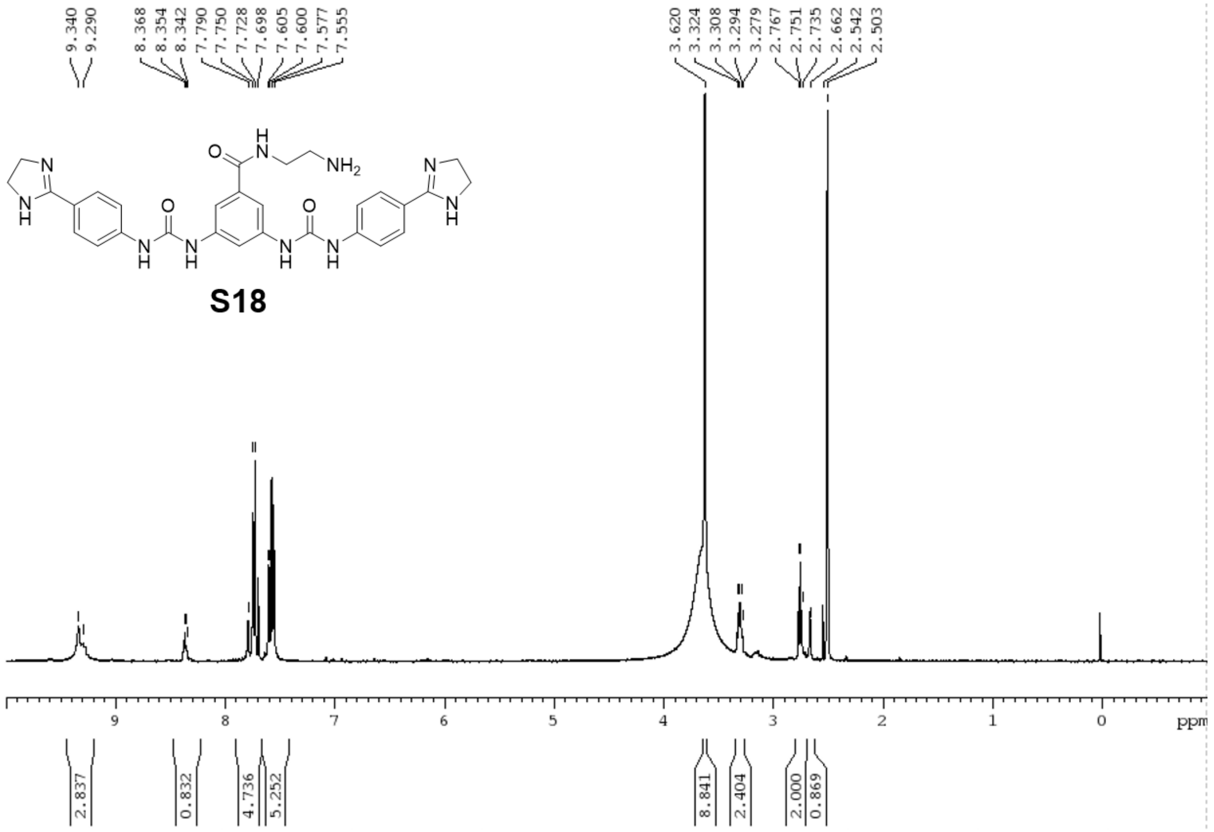
9.340
9.290
8.368
8.354
8.342
7.790
7.750
7.728
7.698
7.605
7.600
7.577
7.555
3.620
3.324
3.308
3.294
3.279
2.767
2.751
2.735
2.662
2.542
2.503
N
H
O
NH2
S18
ppm
2.837
0.832
4.736
5.252
8.841
2.404
2.000
0.869

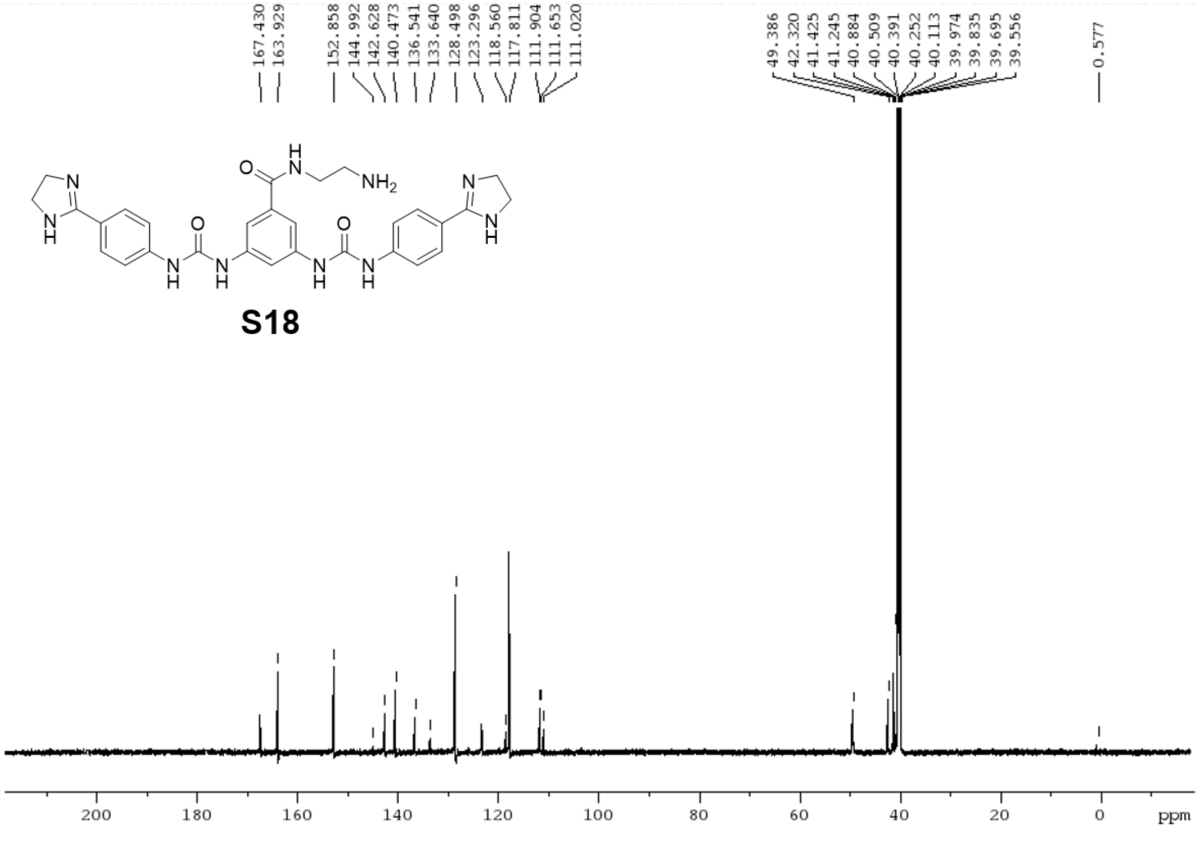
167.430
163.929
152.858
144.992
142.628
140.473
136.541
133.640
128.498
123.296
118.560
117.811
111.904
111.653
111.020
49.386
42.320
41.425
41.245
40.884
40.509
40.391
40.252
40.113
39.974
39.835
39.695
39.556
0.577
S18
ppm

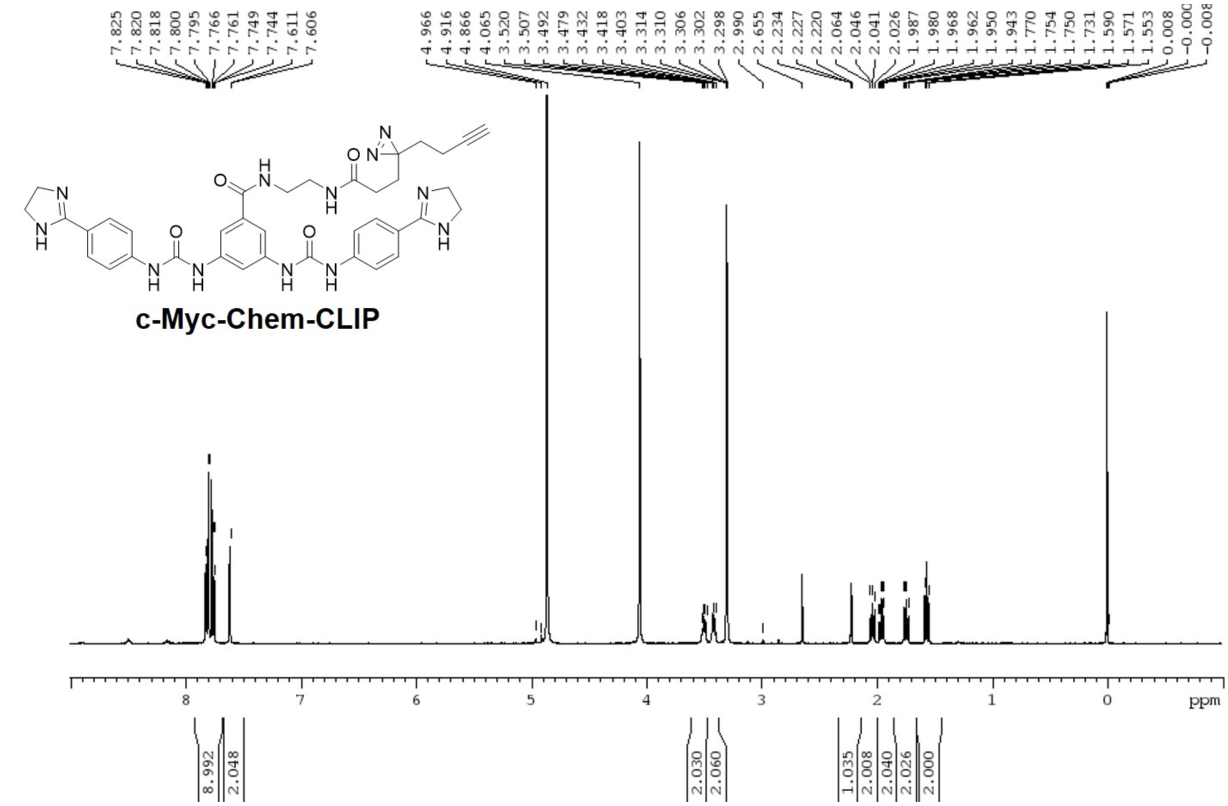
7.825 7.820 7.818 7.800 7.795 7.766 7.761 7.749 7.744 7.611 7.606
4.966 4.916 4.866 4.065 3.520 3.507 3.492 3.479 3.432 3.418 3.403 3.314 3.310 3.306 3.302 3.298 2.990 2.655 2.234 2.227 2.220 2.064 2.046 2.041 2.026 1.987 1.980 1.968 1.962 1.950 1.943 1.770 1.754 1.750 1.731 1.590 1.571 1.553 0.008 -0.000 -0.008
c-Myc-Chem-CLIP
8.992 2.048 2.030 2.060 1.035 2.008 2.040 2.026 2.000
ppm

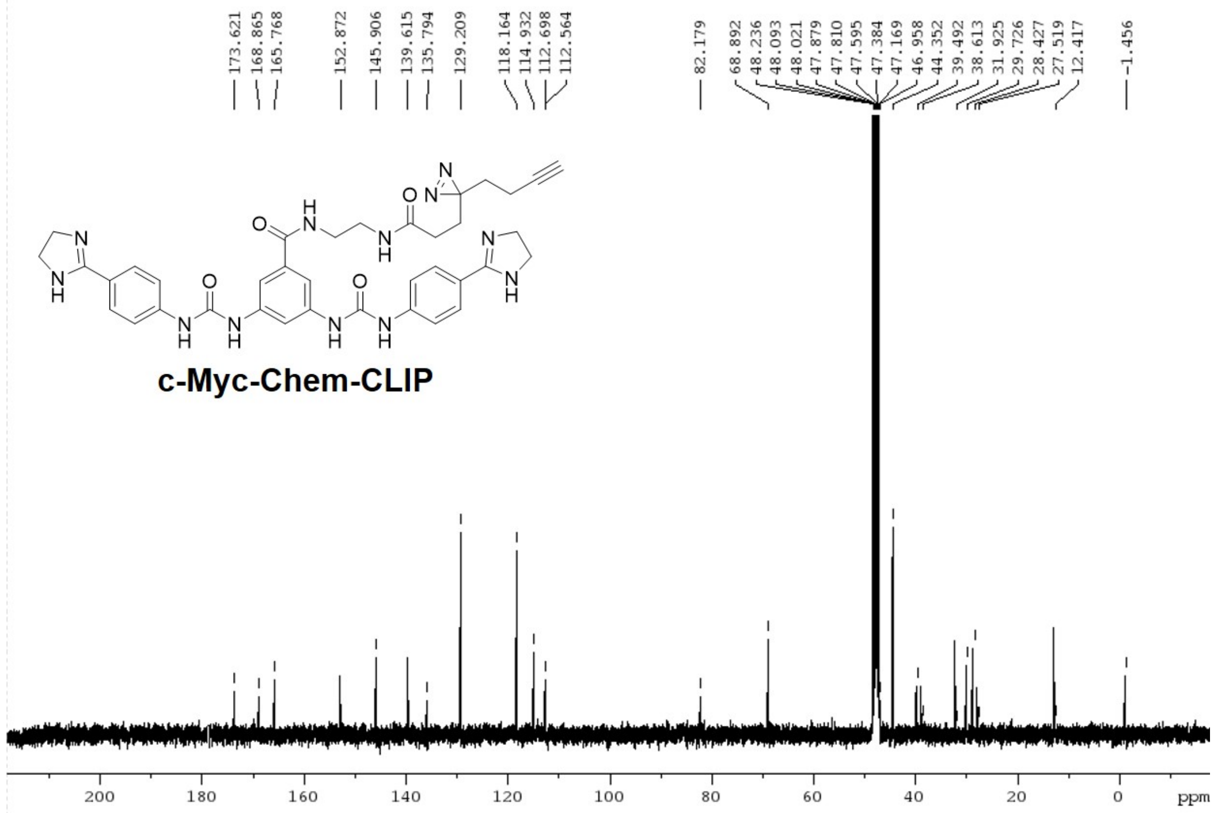
173.621 168.865 165.768 152.872 145.906 139.615 135.794 129.209 118.164 114.932 112.698 112.564 82.179 68.892 48.236 48.093 48.021 47.879 47.810 47.595 47.384 47.169 46.958 44.352 39.492 38.613 31.925 29.726 28.427 27.519 12.417 -1.456
c-Myc-Chem-CLIP
200 180 160 140 120 100 80 60 40 20 0 ppm

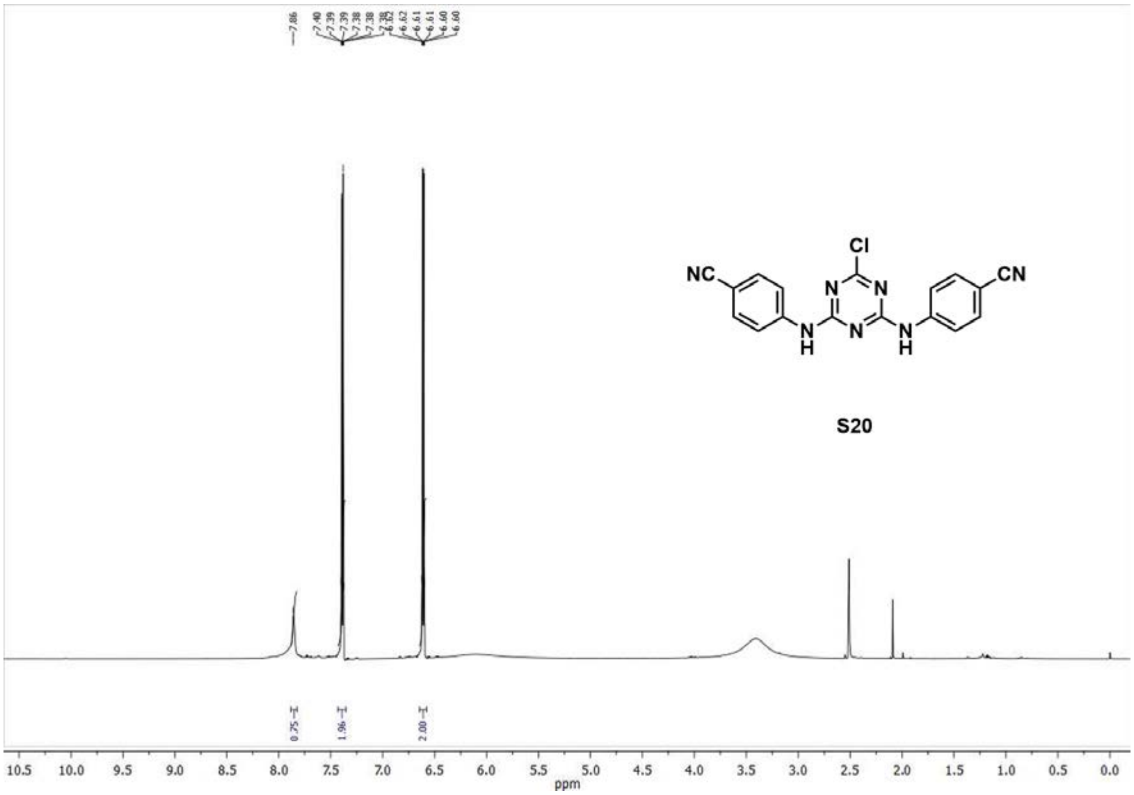

7.86
7.40
7.39
7.39
7.38
7.38
7.38
6.62
6.62
6.61
6.61
6.60
6.60
NC
Cl
N
CN
H
S20
0.75
1.96
2.00
ppm

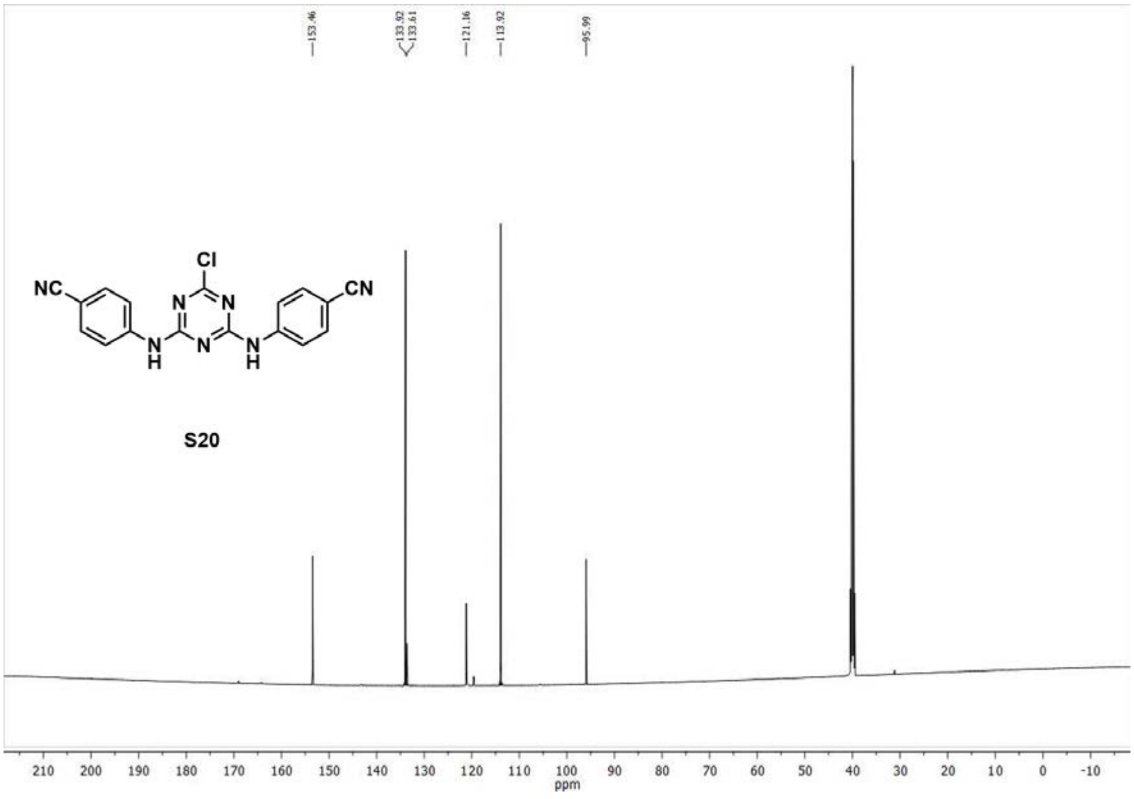

153.46
133.92
133.61
121.16
113.92
95.99
NC
Cl
N
CN
H
S20
ppm

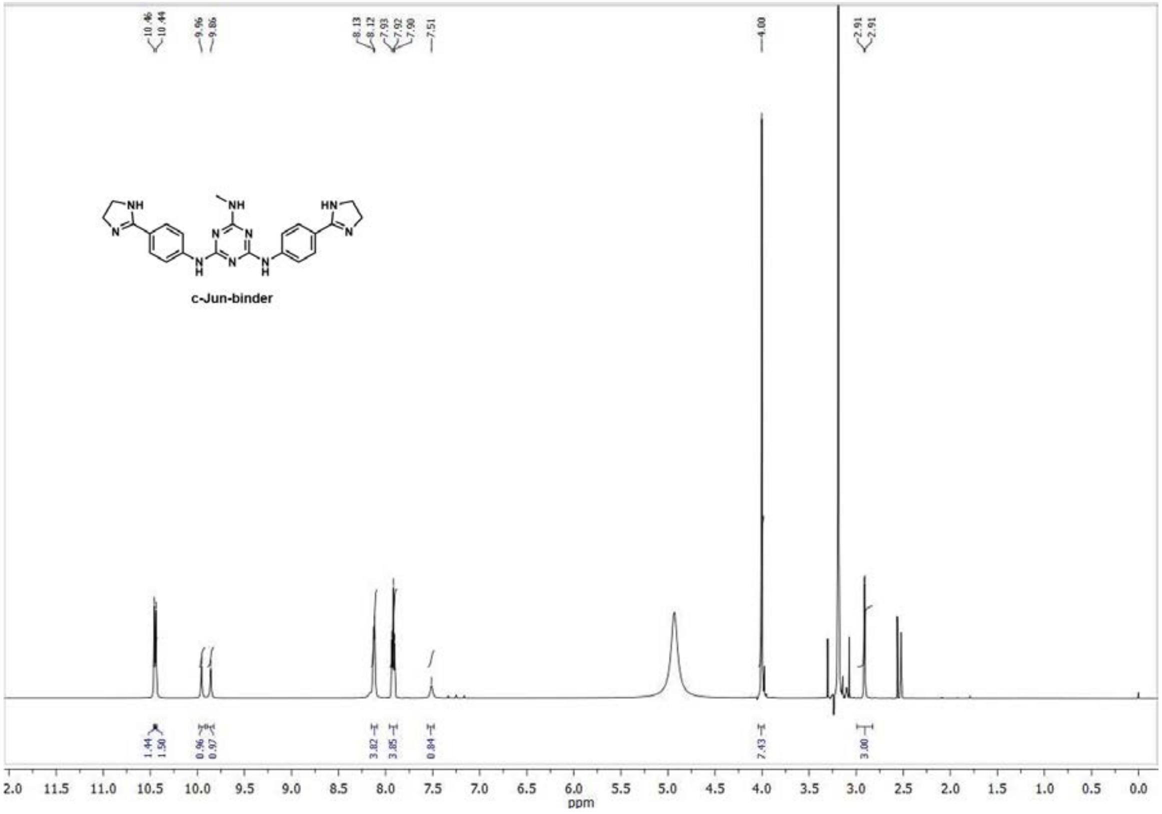

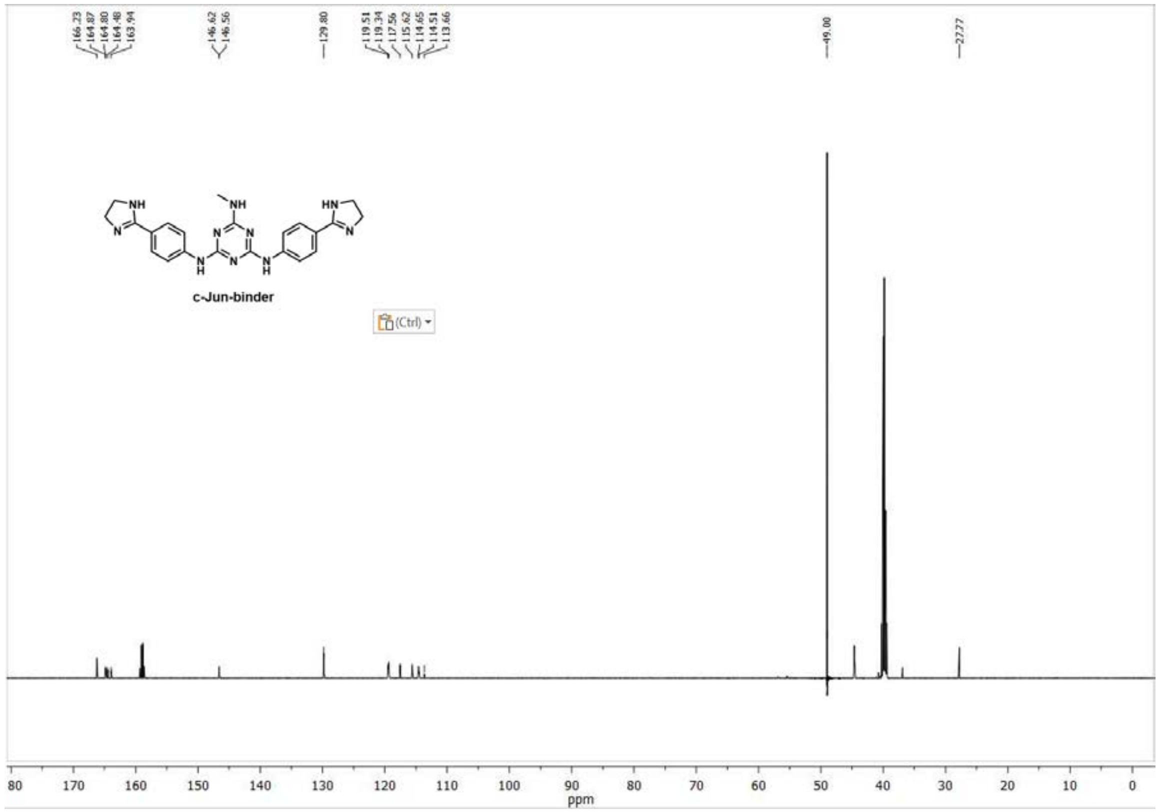

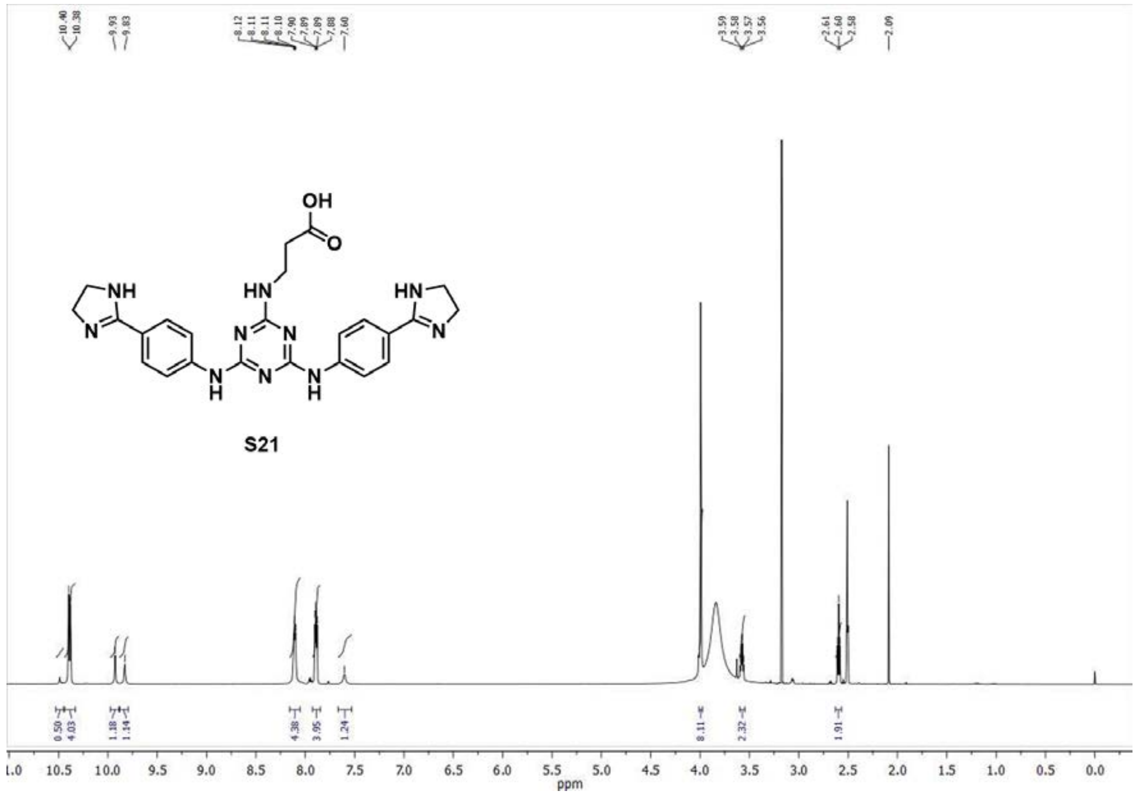
10.40
10.38
9.93
9.83
8.12
8.11
8.11
8.10
7.90
7.89
7.89
7.88
7.60
3.59
3.58
3.57
3.56
2.61
2.60
2.58
2.09
OH
O
HN
NH
N
S21
0.50
4.03
1.18
1.14
4.38
3.95
1.24
8.11
2.32
1.91
ppm

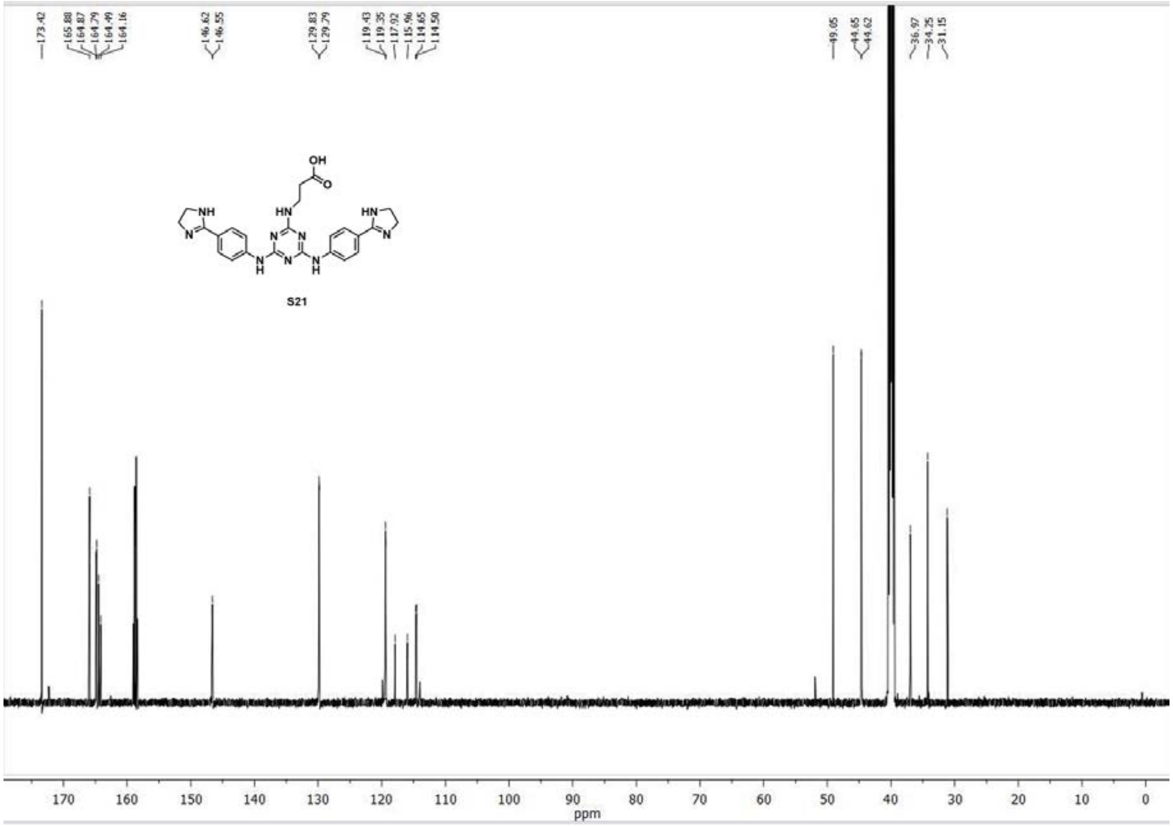
173.42
165.88
164.87
164.79
164.49
164.16
146.62
146.55
129.83
129.29
119.43
119.35
117.92
115.96
114.65
114.50
49.05
44.65
44.62
36.97
34.25
31.15
S21
ppm

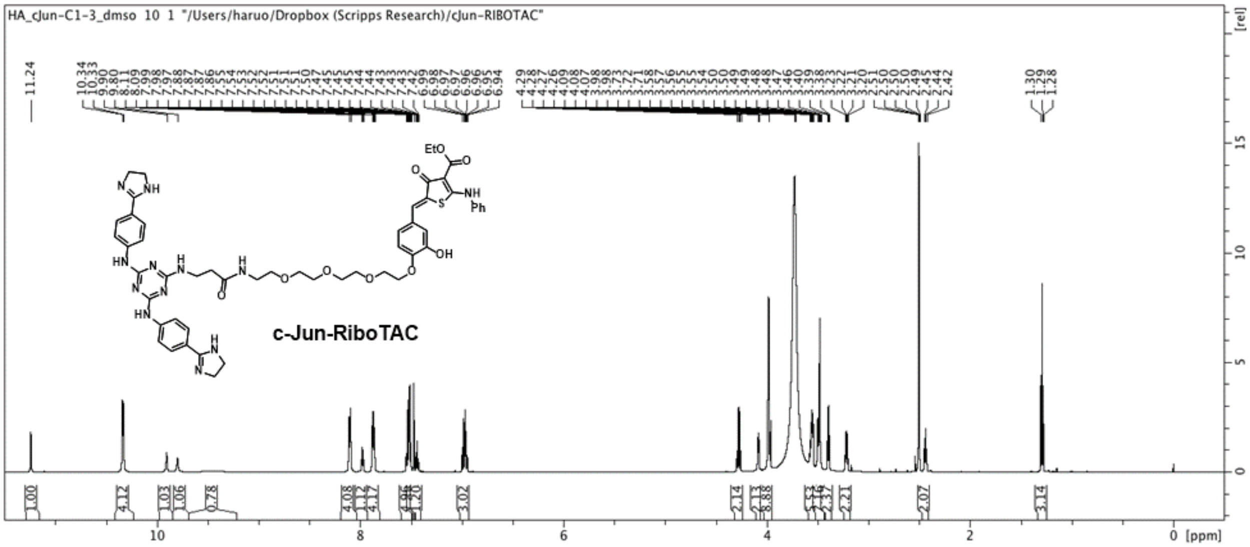

HA_cJun-C1-3_dmso 10 1 "/Users/haruo/Dropbox (Scripps Research)/cJun-RIBOTAC"
c-Jun-RiboTAC
[ppm]
[rel]

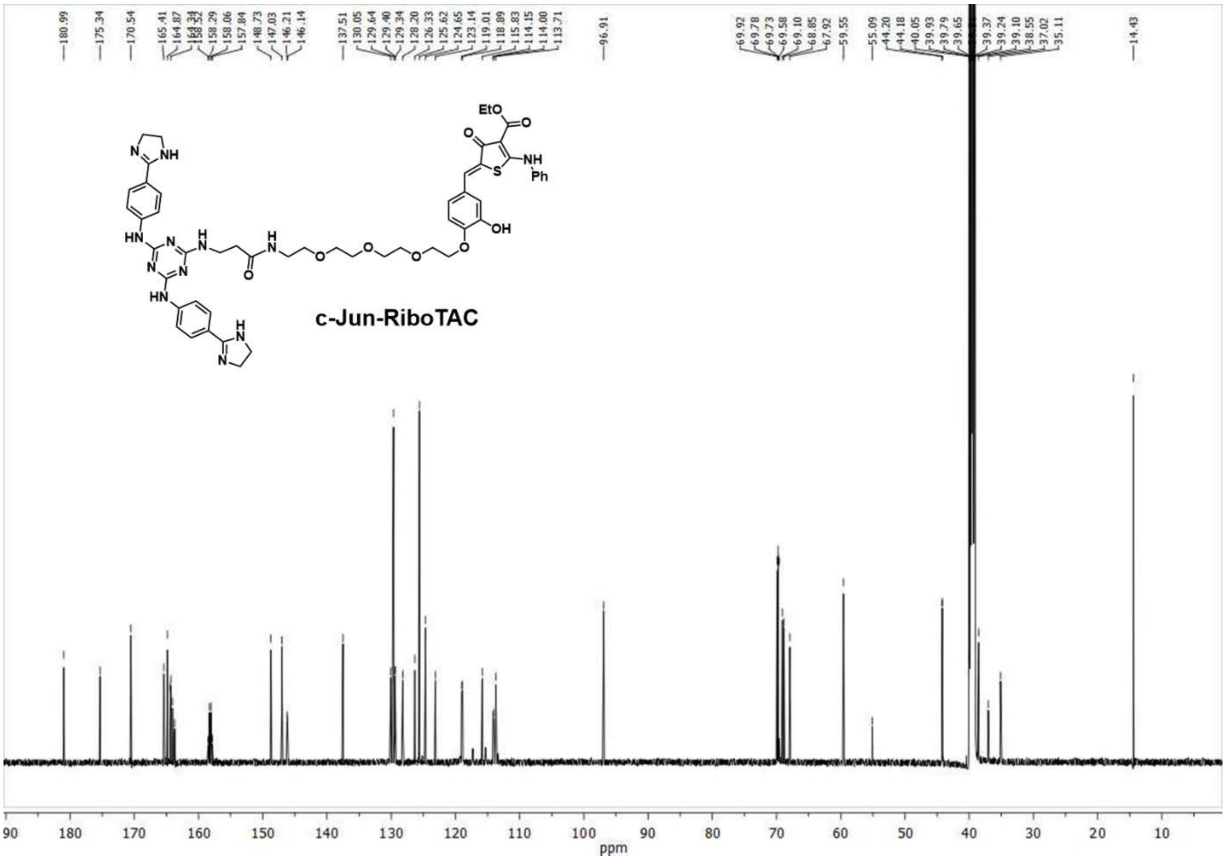

c-Jun-RiboTAC
ppm

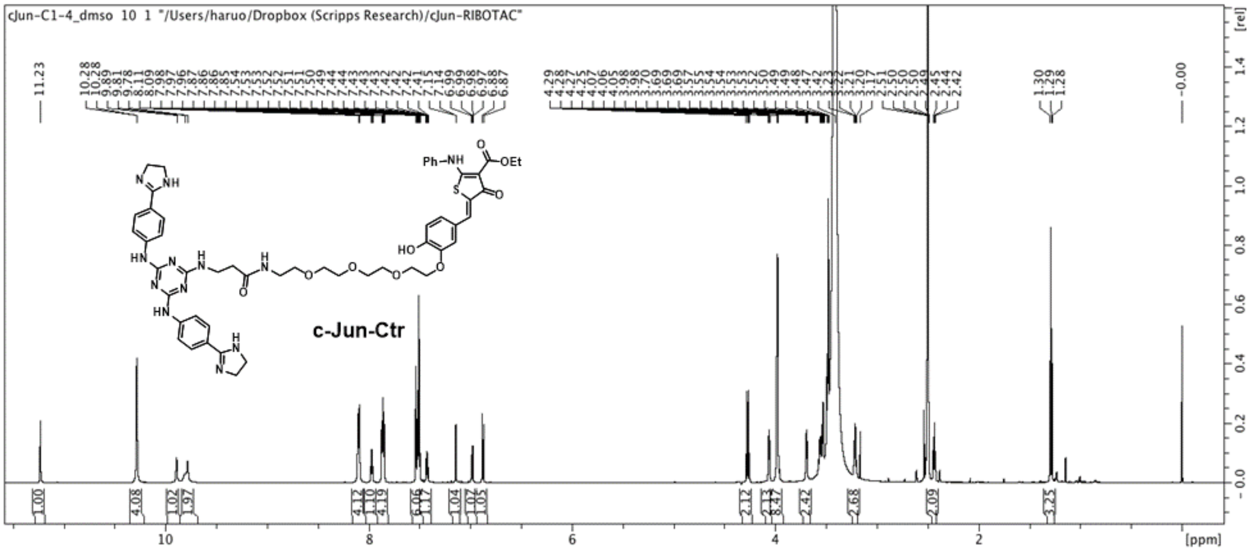
cJun-C1-4_dmso 10 1 "/Users/haruo/Dropbox (Scripps Research)/cJun-RIBOTAC"
c-Jun-Ctr
[ppm]
[rel]

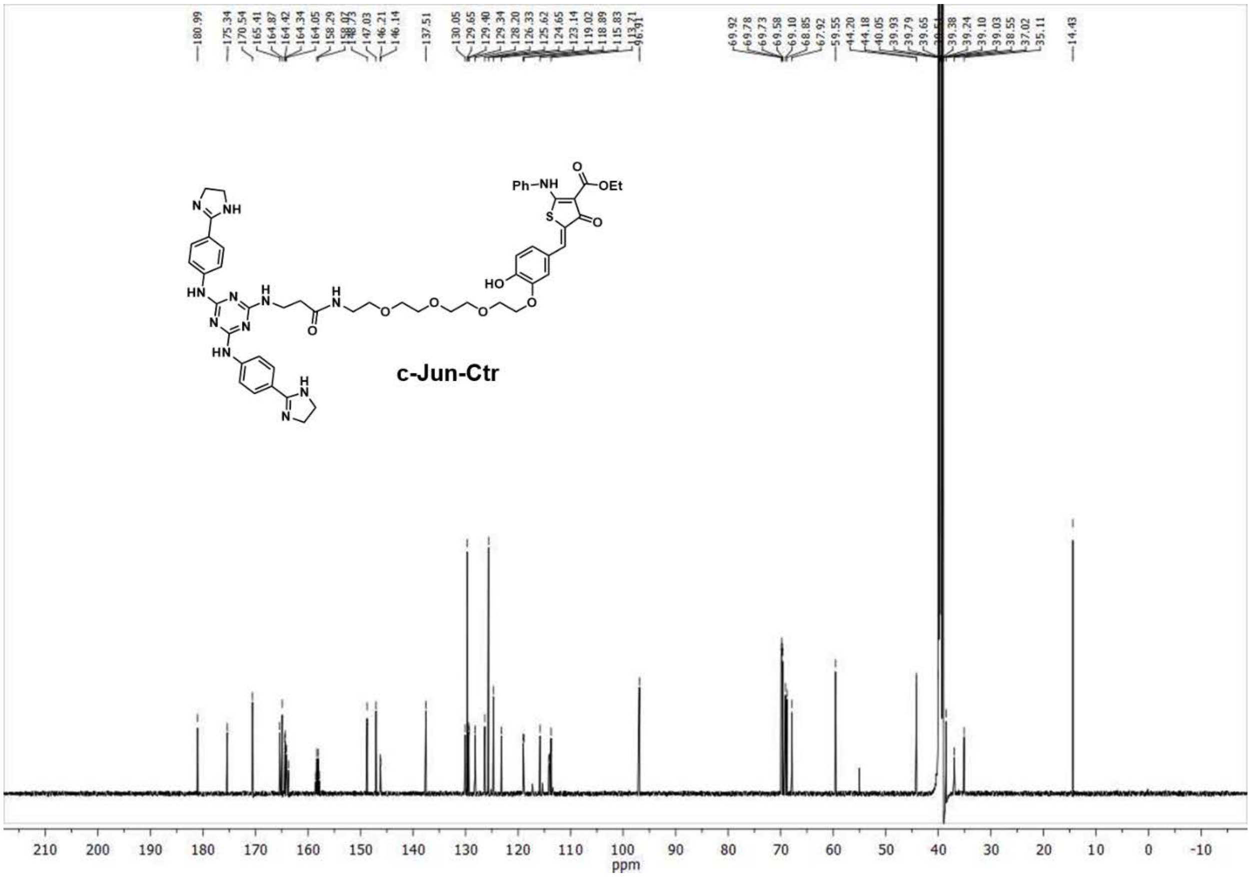
c-Jun-Ctr
ppm

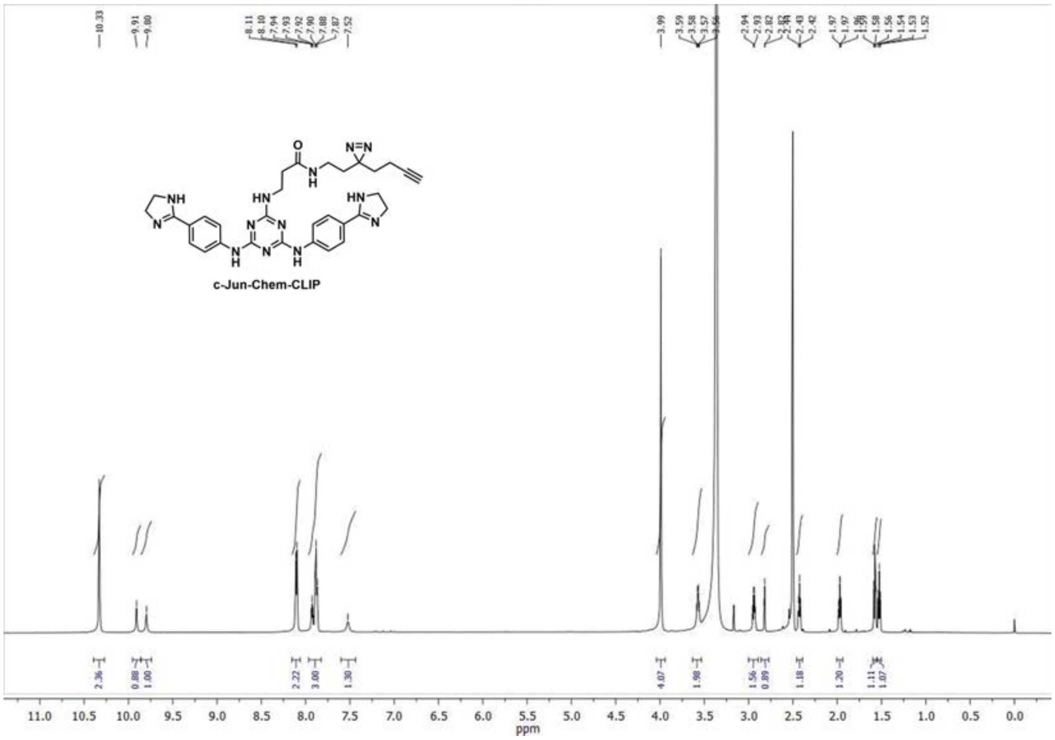
c-Jun-Chem-CLIP
10.33
9.91
9.80
8.11
8.10
7.94
7.93
7.92
7.90
7.88
7.87
7.52
3.99
2.36
0.88
1.00
2.22
3.00
1.30
4.07
1.98
1.56
0.89
1.18
1.20
1.11
1.07
ppm

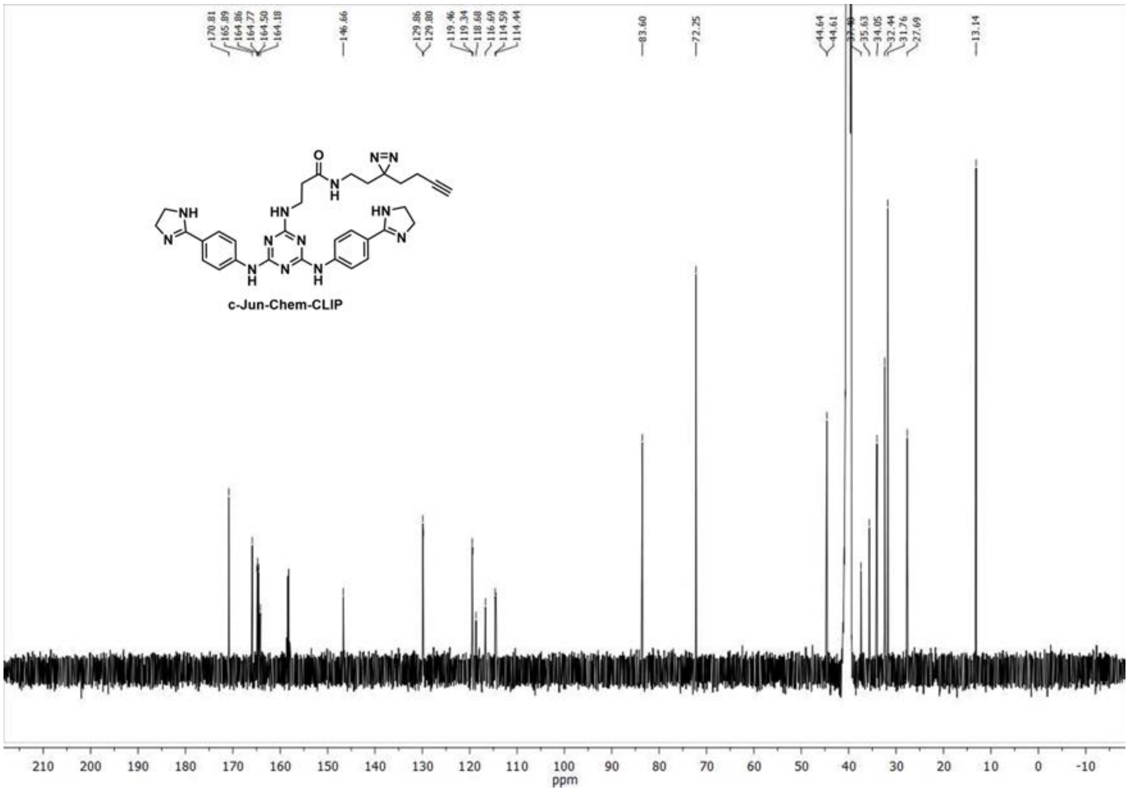
c-Jun-Chem-CLIP
170.81
165.89
164.86
164.77
164.50
164.18
146.66
129.86
129.80
119.46
119.34
118.68
116.69
114.59
114.44
83.60
72.25
44.64
44.61
35.63
34.05
32.44
31.76
27.69
13.14
ppm

HPLC Traces and HRMS:

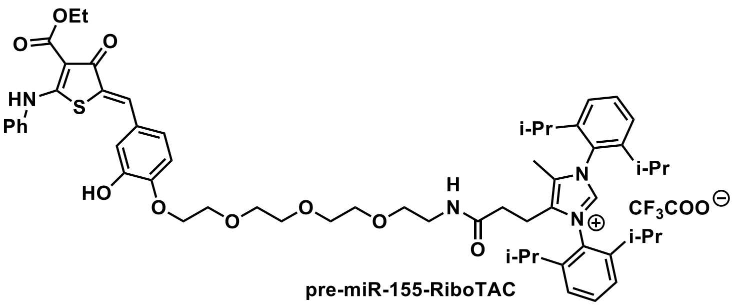

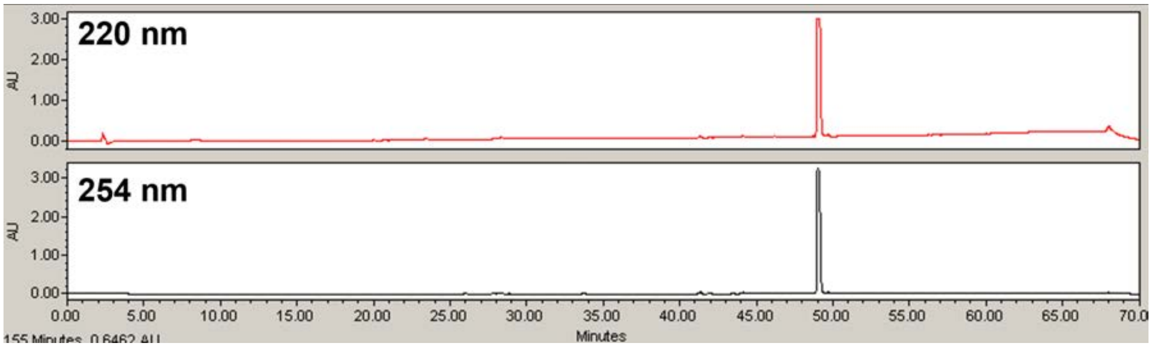

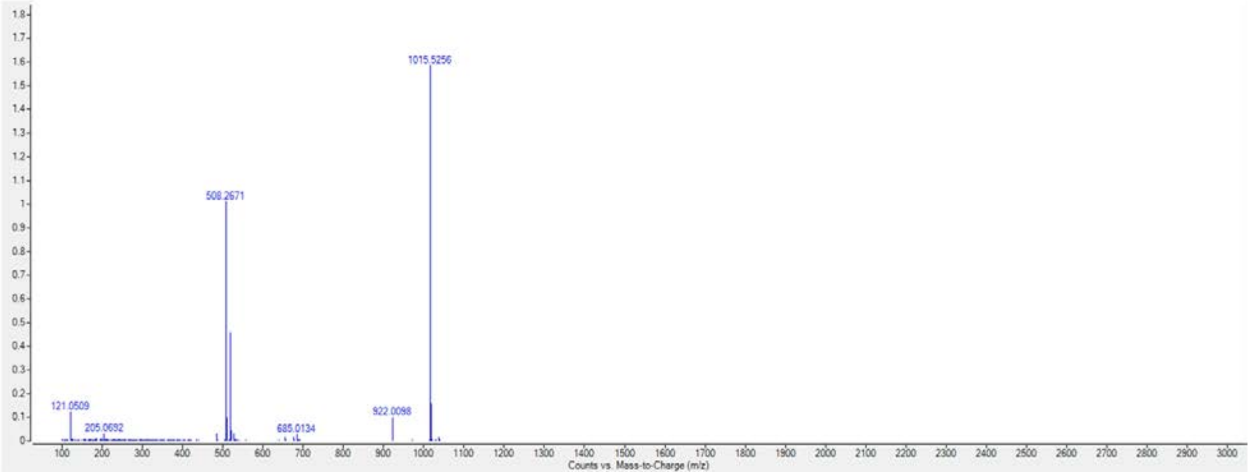

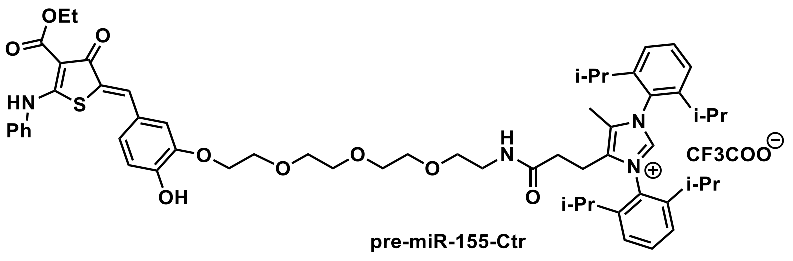
OEt
HN
Ph
S
OH
O
H
N
i-Pr
CF3COO
pre-miR-155-Ctr

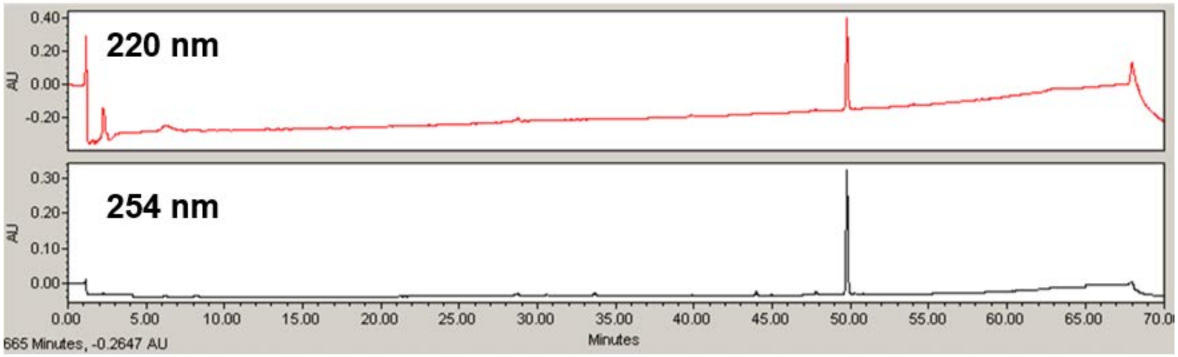
220 nm
254 nm
AU
Minutes
665 Minutes, -0.2647 AU

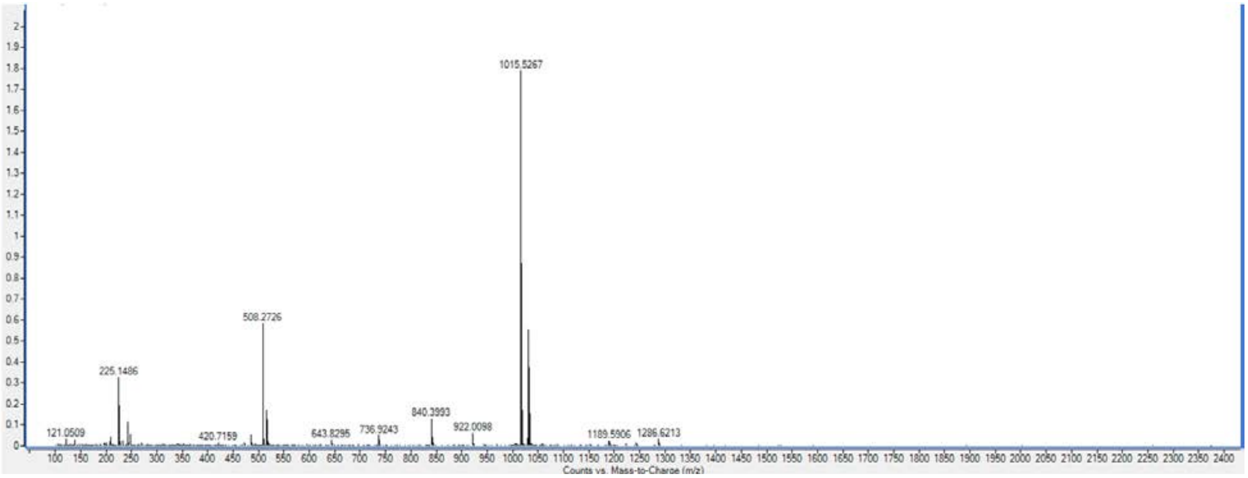
1015.5267
508.2726
225.1486
121.0509
420.7159
643.8295
736.9243
840.3993
922.0098
1189.5906
1286.6213
Counts vs. Mass-to-Charge (m/z)

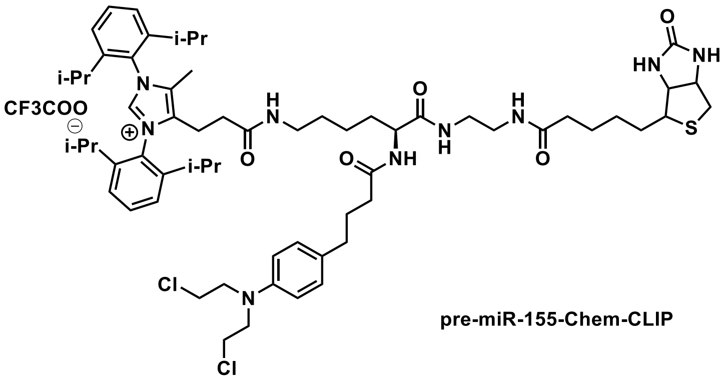

pre-miR-155-Chem-CLIP

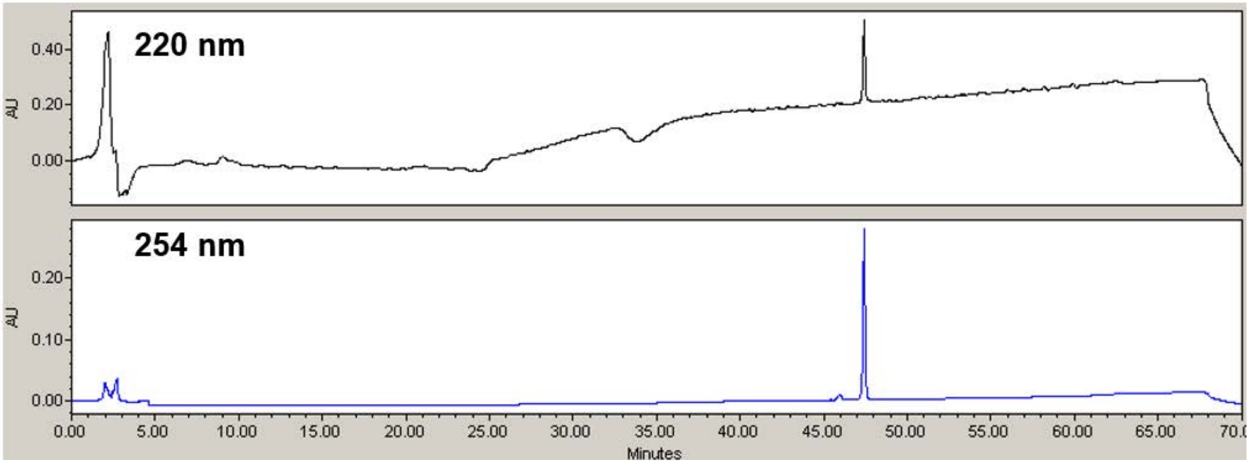

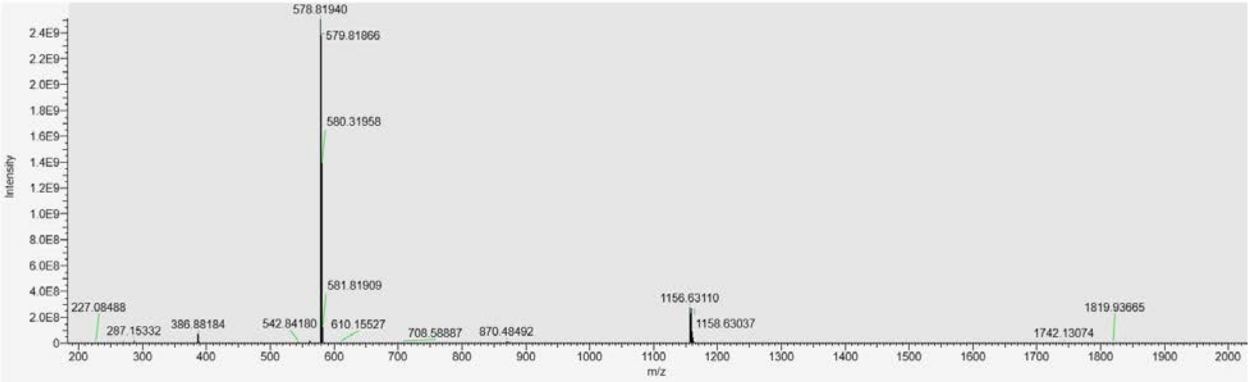

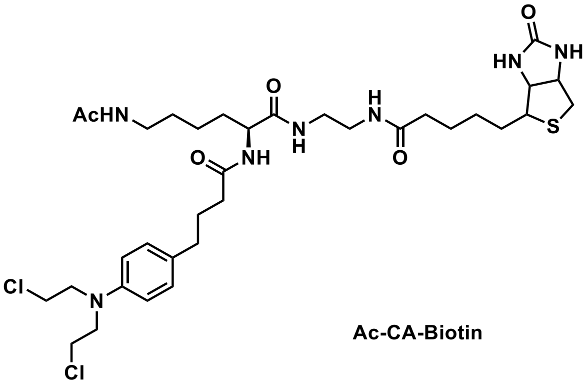

Ac-CA-Biotin

Chemical Formula: $C_{34}H_{53}Cl_2N_7O_5S$
Exact Mass: 741.3206
Molecular Weight: 742.8020

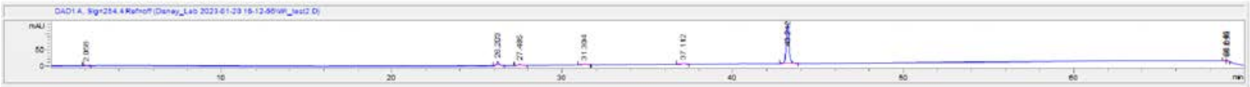

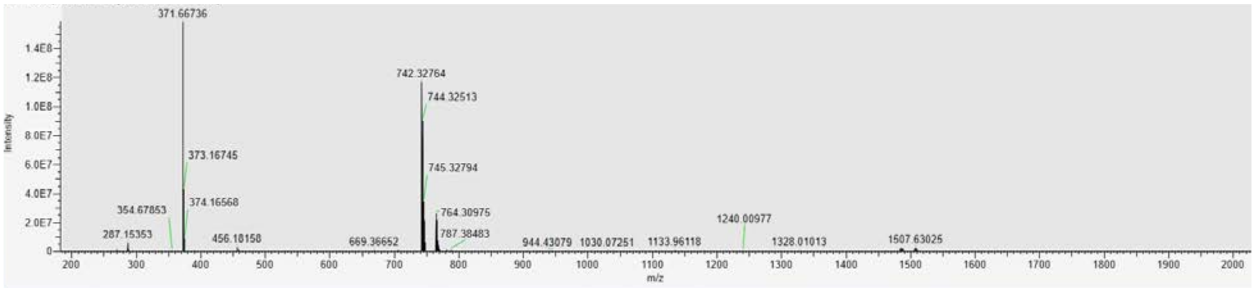

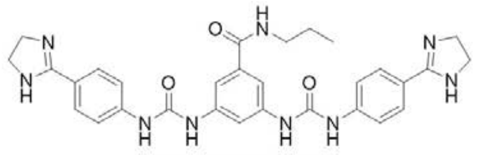

c-Myc-amide binder

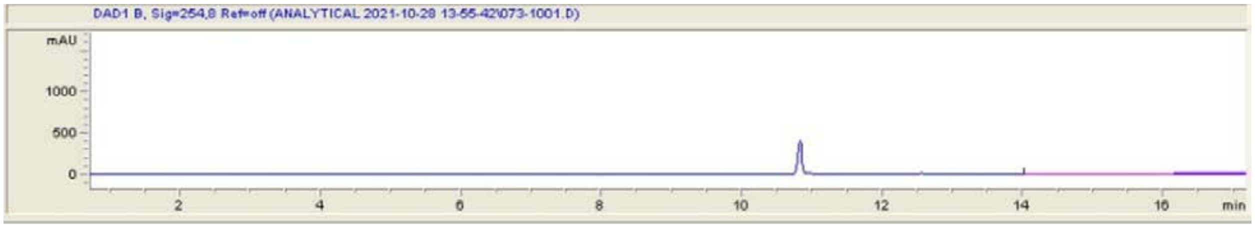

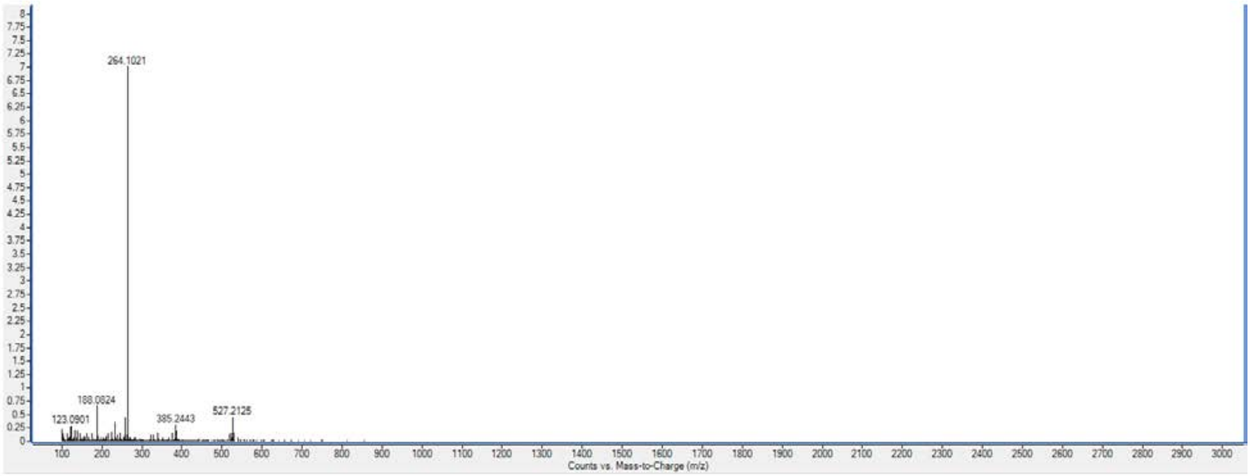

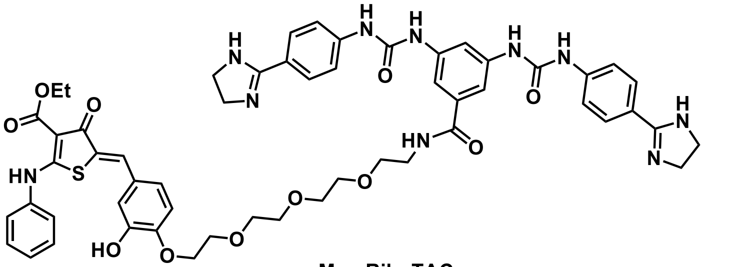

c-Myc-RiboTAC

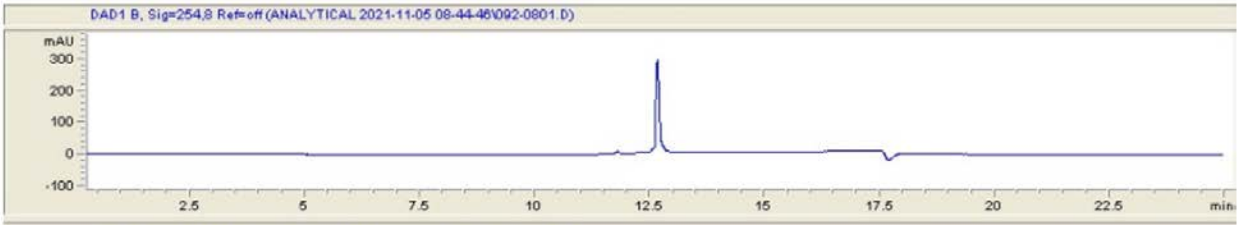

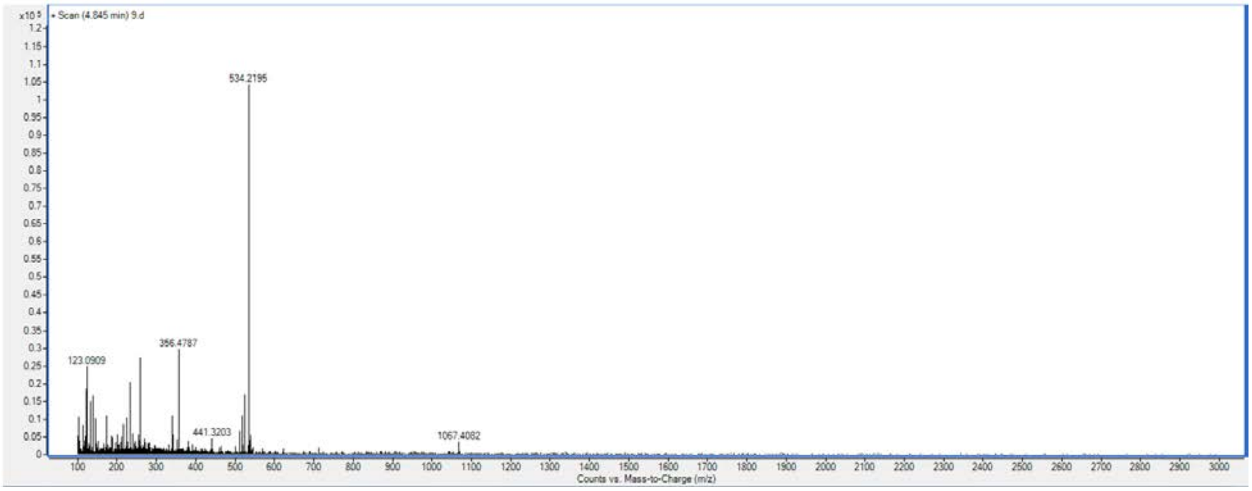

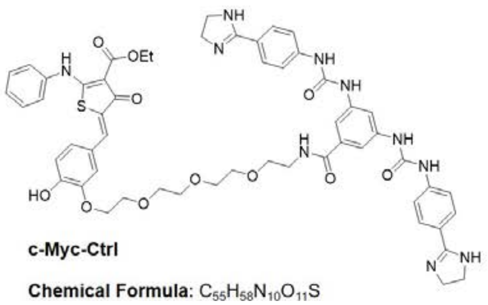

c-Myc-Ctrl

Chemical Formula: $C_{55}H_{58}N_{10}O_{11}S$

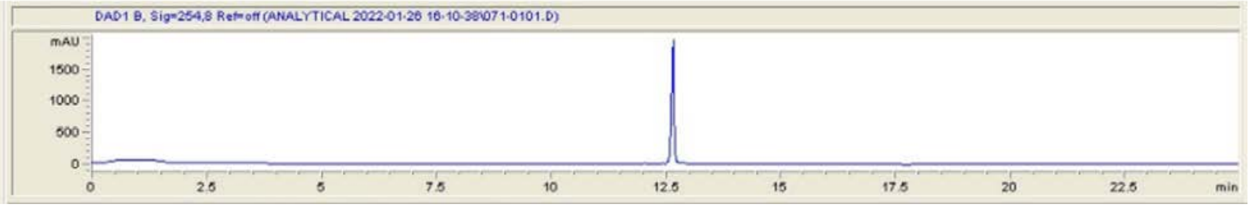

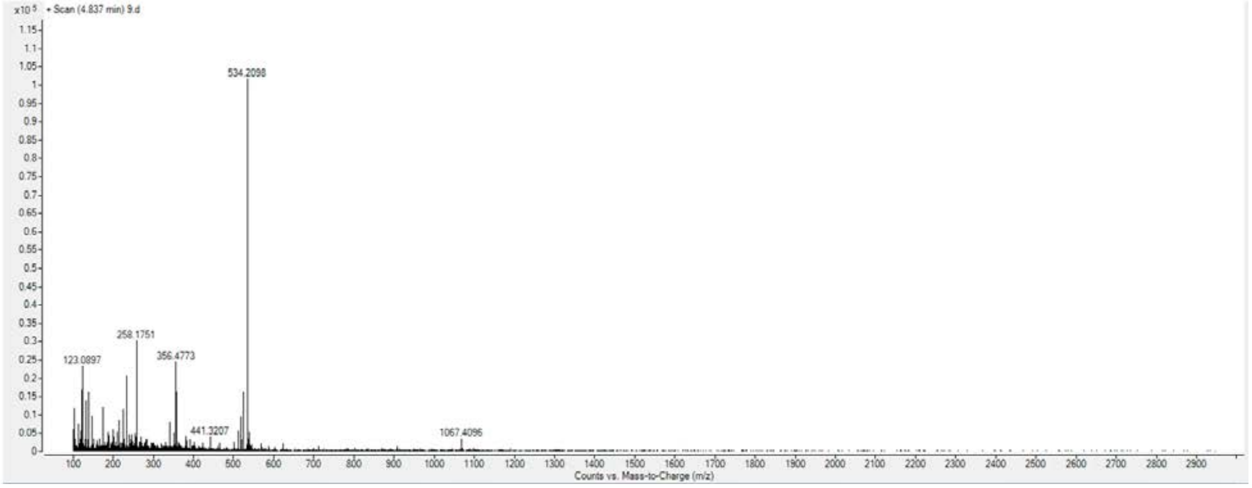

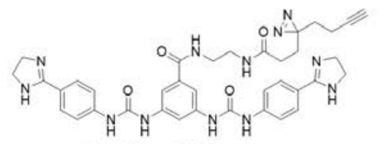

c-Myc-Chem-CLIP

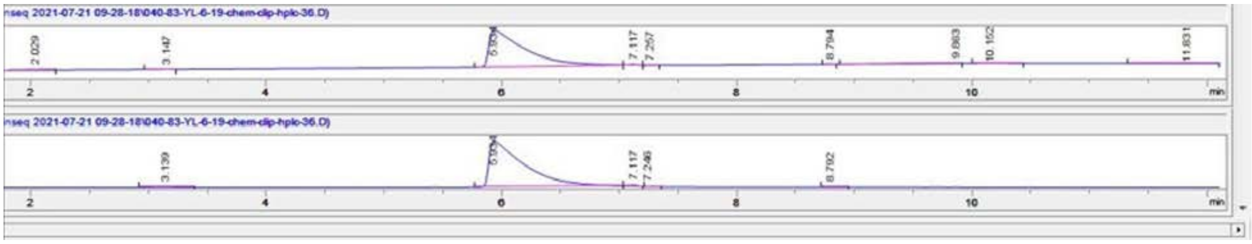

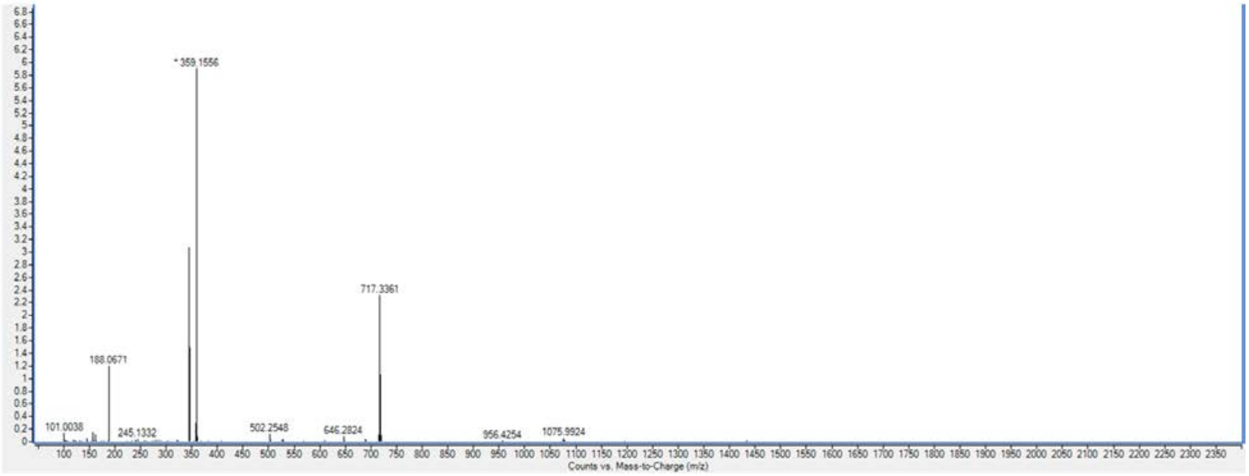

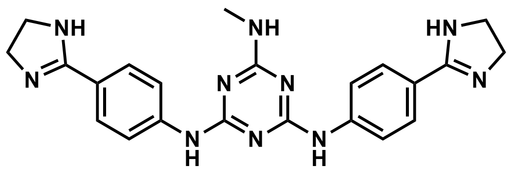

c-Jun-binder

Chemical Formula: $C_{22}H_{24}N_{10}$
Exact Mass: 428.2185
Molecular Weight: 428.5040

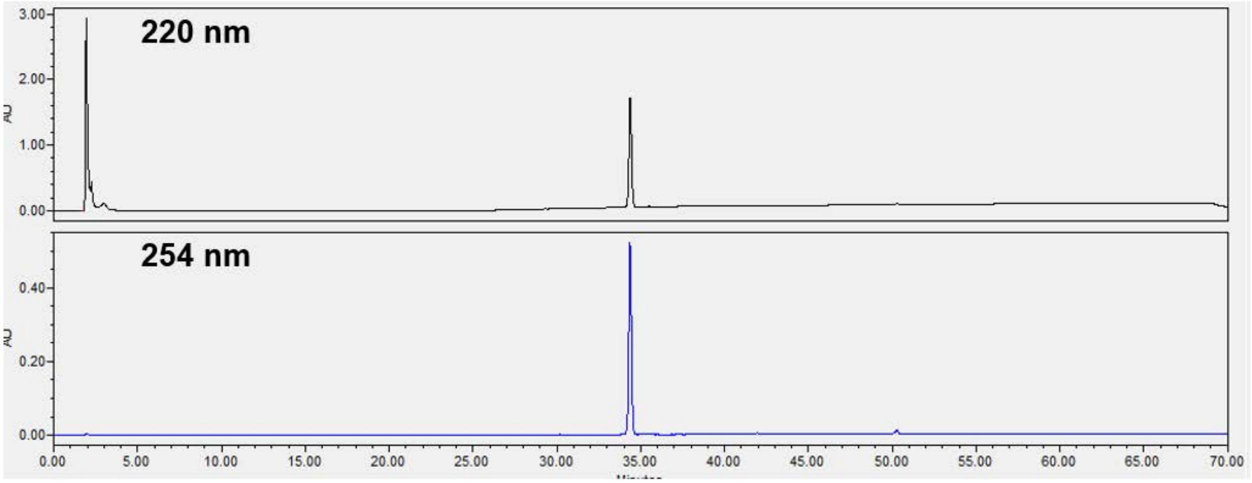

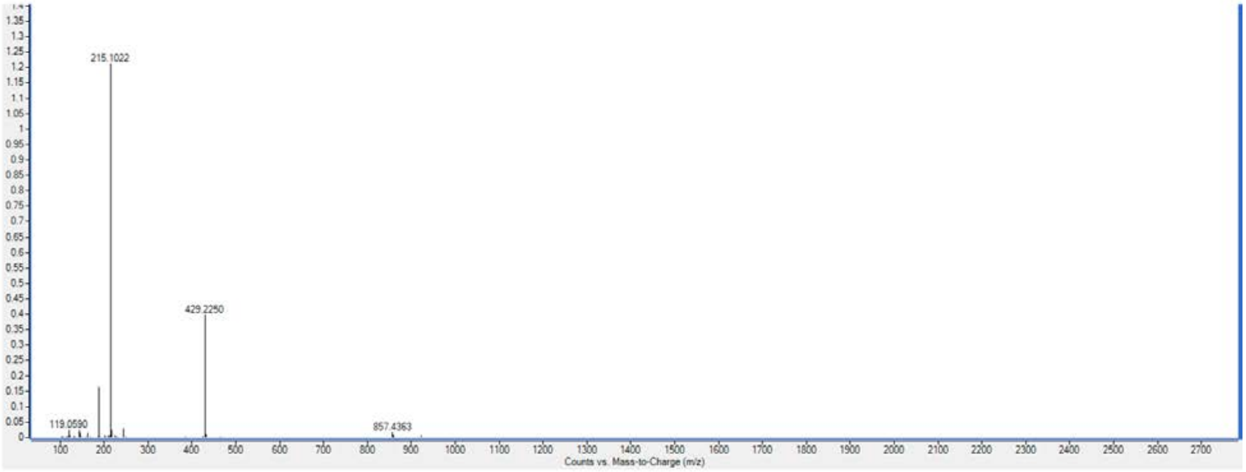

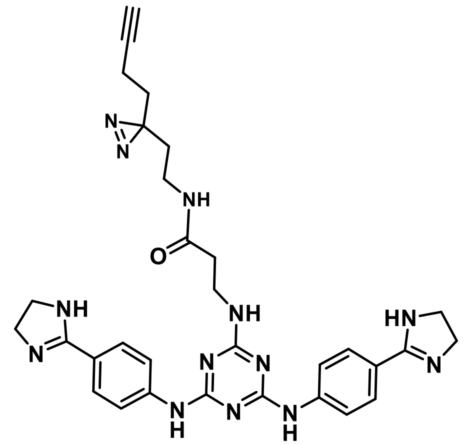

c-Jun-Chem-CLIP

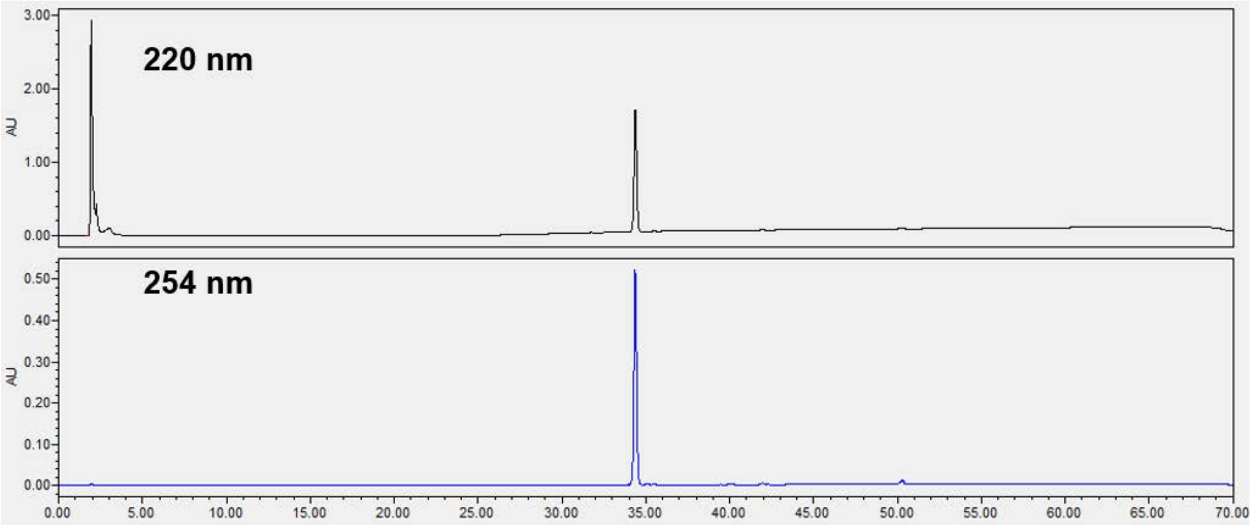

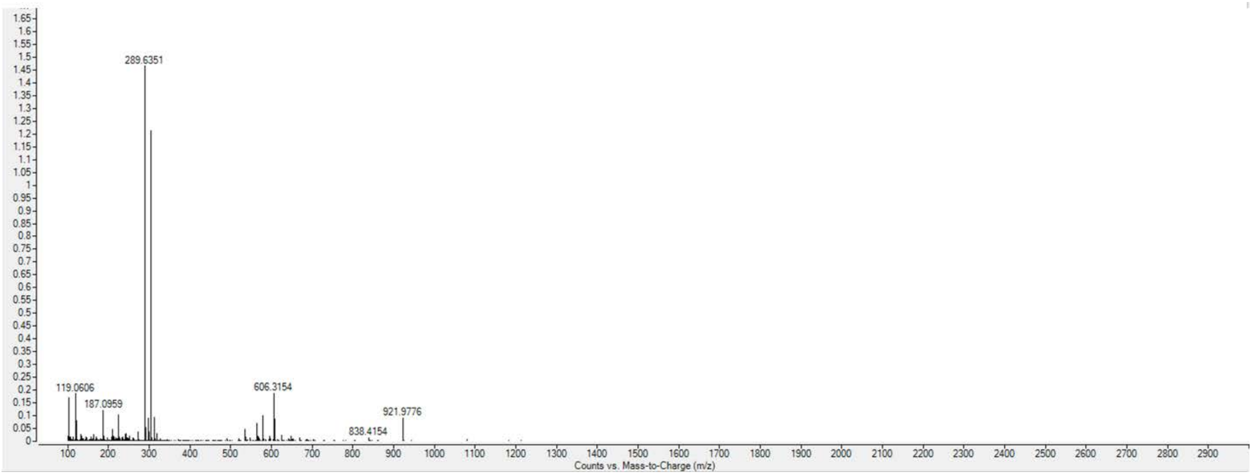

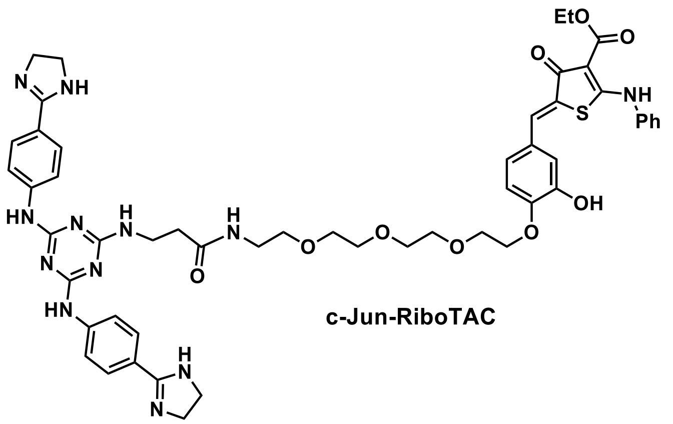

c-Jun-RiboTAC

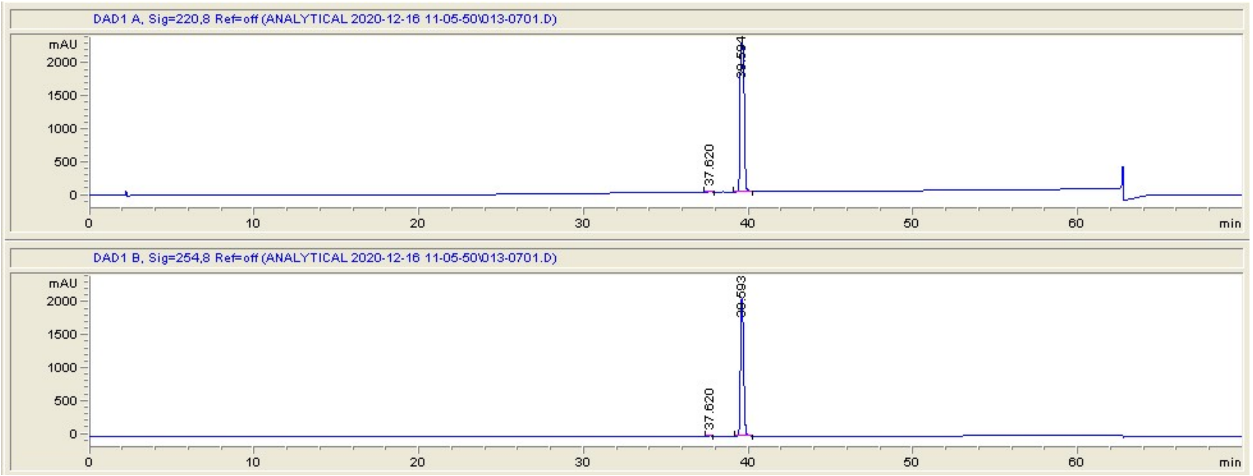

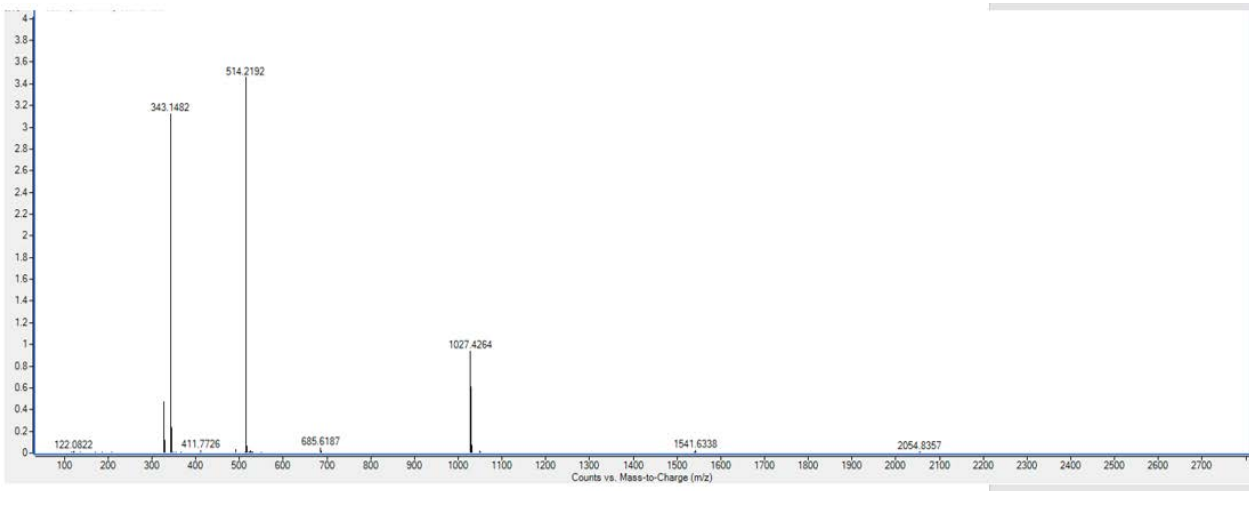

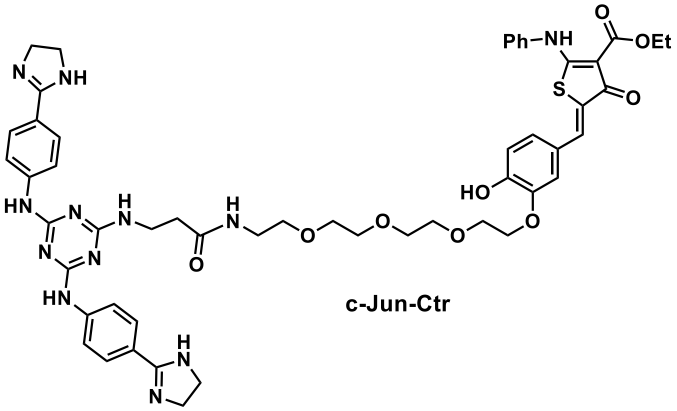
Ph–NH
O
OEt
S
O
HO
O
O
O
O
N
NH
HN
N
N
N
H
N
H
N
O
HN
H
N
N

c-Jun-Ctr

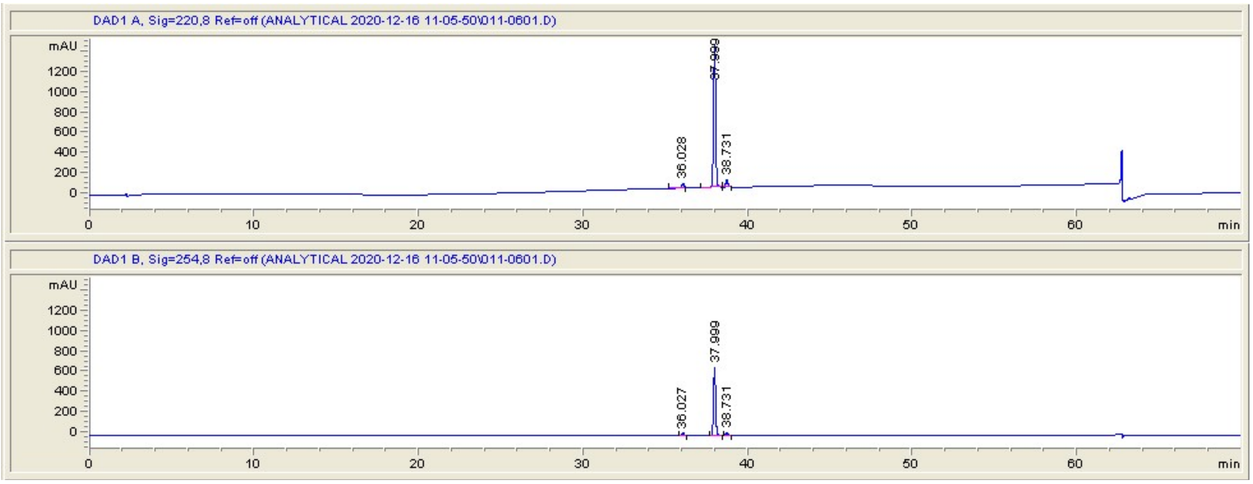
DAD1 A, Sig=220,8 Ref=off (ANALYTICAL 2020-12-16 11-05-50\011-0601.D)
mAU
36.028
37.999
38.731
min
DAD1 B, Sig=254,8 Ref=off (ANALYTICAL 2020-12-16 11-05-50\011-0601.D)
mAU
36.027
37.999
38.731
min

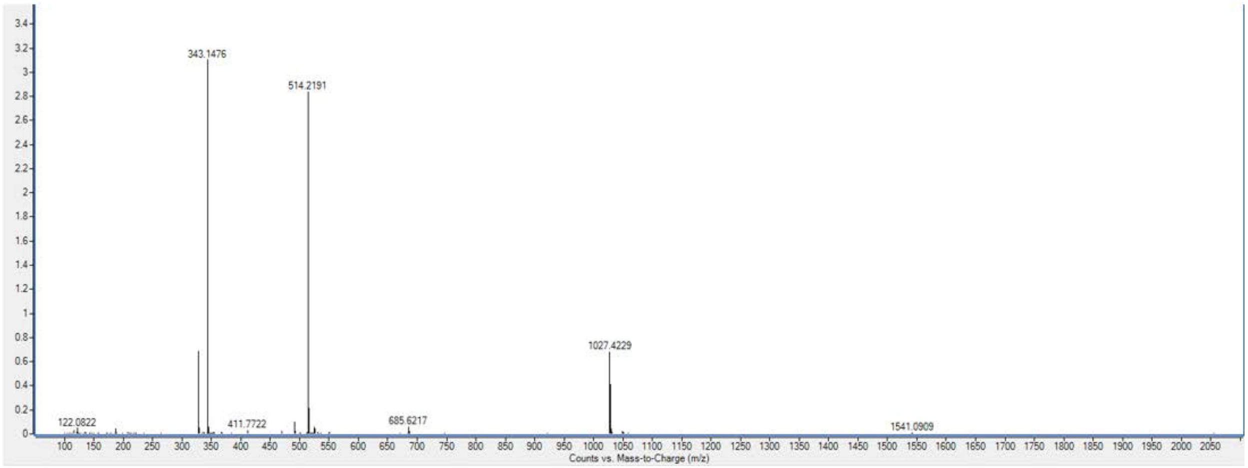
343.1476
514.2191
1027.4229
122.0822
411.7722
685.6217
1541.0909
Counts vs. Mass-to-Charge (m/z)

REFERENCES

1 Costales, M. G. *et al.* Small-molecule targeted recruitment of a nuclease to cleave an oncogenic RNA in a mouse model of metastatic cancer. *Proc. Natl. Acad. Sci. U.S.A.* **117**, 2406-2411 (2020). https://doi.org:10.1073/pnas.1914286117

11 Velagapudi, S. P. *et al.* Approved Anti-cancer drugs target oncogenic non-coding RNAs. *Cell Chem. Biol.* **25**, 1086-1094 (2018). https://doi.org:10.1016/j.chembiol.2018.05.015

13 Velagapudi, S. P. *et al.* Defining RNA-small molecule affinity landscapes enables design of a small molecule inhibitor of an oncogenic concoding RNA. *ACS Cent. Sci.* **3**, 205-216 (2017). https://doi.org:10.1021/acscentsci.7b00009

15 Haniff, H. S. *et al.* Design of a small molecule that stimulates vascular endothelial growth factor A enabled by screening RNA fold–small molecule interactions. *Nat. Chem.* **12**, 952–961 (2020). https://doi.org:10.1038/s41557-020-0514-4

17 Morgan, B. S., Forte, J. E., Culver, R. N., Zhang, Y. & Hargrove, A. E. Discovery of key physicochemical, structural, and spatial properties of RNA-targeted bioactive ligands. *Angew. Chem. Int. Ed. Engl.* **56**, 13498-13502 (2017). https://doi.org:10.1002/anie.201707641

18 Liu, B. *et al.* Analysis of secondary structural elements in human microRNA hairpin precursors. *BMC Bioinformatics* **17**, 112 (2016). https://doi.org:10.1186/s12859-016-0960-6

35 Agarwal, V., Bell, G. W., Nam, J. W. & Bartel, D. P. Predicting effective microRNA target sites in mammalian mRNAs. *eLife* **4**, e05005 (2015). https://doi.org:10.7554/eLife.05005

49 Zengerle, M., Chan, K. H. & Ciulli, A. Selective small molecule induced degradation of the BET bromodomain protein BRD4. *ACS Chem. Biol.* **10**, 1770-1777 (2015). https://doi.org:10.1021/acschembio.5b00216

51 Fernandez, P. C. *et al.* Genomic targets of the human c-Myc protein. *Genes Dev.* **17**, 1115-1129 (2003). https://doi.org:10.1101/gad.1067003

52 Dang, C. V. *et al.* The c-Myc target gene network. *Semin. Cancer Biol.* **16**, 253-264 (2006). https://doi.org:10.1016/j.semcancer.2006.07.014

53 Kim, J., Lee, J.-H. & Iyer, V. R. Global identification of Myc target genes reveals its direct role in mitochondrial biogenesis and its E-box usage in vivo. *PLoS ONE* **3**, e1798 (2008). https://doi.org:10.1371/journal.pone.0001798

59 <https://www.cellsignal.com/products/primary-antibodies/c-myc-d84c12-rabbit-mab/5605>

60 Velagapudi, S. P., Gallo, S. M. & Disney, M. D. Sequence-based design of bioactive small molecules that target precursor microRNAs. *Nat. Chem. Biol*. **10**, 291-297 (2014). https://doi.org:10.1038/Nchembio.1452

61 Zhang, J. H., Chung, T. D. & Oldenburg, K. R. A simple statistical parameter for use in evaluation and validation of high throughput screening assays. *J. Biomol. Screen.* **4**, 67-73 (1999). https://doi.org:10.1177/108705719900400206

62 Nettling, M. et al. DiffLogo: a comparative visualization of sequence motifs. *BMC Bioinformatics* **16**, 387 (2015). https://doi.org:10.1186/s12859-015-0767-x

63 Haniff, H. S., Graves, A. & Disney, M. D. Selective small molecule recognition of RNA base pairs. *ACS Combi. Sci.* **20**, 482-491 (2018). https://doi.org:10.1021/acscombsci.8b00049

64 Velagapudi, S. P., Pushechnikov, A., Labuda, L. P., French, J. M. & Disney, M. D. Probing a 2-aminobenzimidazole library for binding to RNA internal loops via two-dimensional combinatorial screening. *ACS Chem. Biol*. **7**, 1902-1909 (2012). https://doi.org:10.1021/cb300213g

65 Costales, M. G., Matsumoto, Y., Velagapudi, S. P. & Disney, M. D. Small molecule targeted recruitment of a nuclease to RNA. *J. Am. Chem. Soc.* **140**, 6741-6744 (2018). https://doi.org:10.1021/jacs.8b01233

67 Downes, N. L., Laham-Karam, N., Kaikkonen, M. U. & Ylä-Herttuala, S. Differential but complementary HIF1α and HIF2α transcriptional regulation. *Mol. Ther.* **26**, 1735-1745 (2018). https://doi.org:https://doi.org/10.1016/j.ymthe.2018.05.004

68 Dobin, A. et al. STAR: ultrafast universal RNA-seq aligner. *Bioinformatics* **29**, 15-21 (2013). https://doi.org:10.1093/bioinformatics/bts635

69 Liao, Y., Smyth, G. K. & Shi, W. featureCounts: an efficient general purpose program for assigning sequence reads to genomic features. *Bioinformatics* **30**, 923-930 (2014). https://doi.org:10.1093/bioinformatics/btt656

70 Love, M. I., Huber, W. & Anders, S. Moderated estimation of fold change and dispersion for RNA-seq data with DESeq2. *Genome. Biol.* **15**, 550 (2014). https://doi.org:10.1186/s13059-014-0550-8

71 Keyes, A. et al. Light as a catalytic switch for block copolymer architectures: metal–organic insertion/light initiated radical (MILRad) polymerization. *Macromolecules* **51**, 7224-7232 (2018). https://doi.org:10.1021/acs.macromol.8b01719

72 Pan, X. et al. Supramolecular graft copolymers in moderately polar media based on hydrogen-bonded aromatic oligoamide units. *Chem. Commun.* **48**, 9510-9512 (2012). https://doi.org:10.1039/C2CC35004A

73 Af Gennäs, G. B. et al. Design, synthesis, and biological activity of isophthalic acid derivatives targeted to the C1 domain of Protein Kinase C. *J. Med. Chem.* **52**, 3969-3981 (2009). https://doi.org:10.1021/jm900229p
