# Supplementary material for: Programming inactive RNA-binding small molecules into bioactive degraders
Source: Nature. 2023 May 24;618(7963):169–79. doi: 10.1038/s41586-023-06091-8 (PMC10232370; doi:10.1038/s41586-023-06091-8)
Supplement: Supplementary file 2 — Reporting Summary [file 41586_2023_6091_MOESM2_ESM.pdf]

nature portfolio

Corresponding author(s): Frank Glorius, Herbert Waldmann, Matthew D. Disney

Last updated by author(s): Mar 13, 2023

Reporting Summary

Nature Portfolio wishes to improve the reproducibility of the work that we publish. This form provides structure for consistency and transparency in reporting. For further information on Nature Portfolio policies, see our Editorial Policies and the Editorial Policy Checklist.

Statistics

For all statistical analyses, confirm that the following items are present in the figure legend, table legend, main text, or Methods section.

n/a	Confirmed	
☐	☒	The exact sample size (*n*) for each experimental group/condition, given as a discrete number and unit of measurement
☐	☒	A statement on whether measurements were taken from distinct samples or whether the same sample was measured repeatedly
☐	☒	The statistical test(s) used AND whether they are one- or two-sided<br>*Only common tests should be described solely by name; describe more complex techniques in the Methods section.*
☒	☐	A description of all covariates tested
☒	☐	A description of any assumptions or corrections, such as tests of normality and adjustment for multiple comparisons
☐	☒	A full description of the statistical parameters including central tendency (e.g. means) or other basic estimates (e.g. regression coefficient) AND variation (e.g. standard deviation) or associated estimates of uncertainty (e.g. confidence intervals)
☐	☒	For null hypothesis testing, the test statistic (e.g. *F*, *t*, *r*) with confidence intervals, effect sizes, degrees of freedom and *P* value noted<br>*Give P values as exact values whenever suitable.*
☒	☐	For Bayesian analysis, information on the choice of priors and Markov chain Monte Carlo settings
☒	☐	For hierarchical and complex designs, identification of the appropriate level for tests and full reporting of outcomes
☒	☐	Estimates of effect sizes (e.g. Cohen's *d*, Pearson's *r*), indicating how they were calculated

Our web collection on statistics for biologists contains articles on many of the points above.

Software and code

Policy information about availability of computer code

Data collection	No custom code was used to collect data.<br>RT-qPCR data was collected on Applied Biosystem QuantStudio 5 v1.3.0.<br>Western blots were collected on AFP Imaging - Mini-Medical/90.<br>Autoradiographic imaging and ethidium bromide staining imaging was performed by Typhoon FLA9500 variable mode imager.<br>Proteomics data were collected by using a Q Exactive HF-X mass spectrometer.<br>RNA-seq data were collected by using a NextSeq 500 sequencer.<br>Microscale thermophoresis binding assays were measured by using the MST Monolith instrument (NT.115).

Data analysis	No custom code was used for data analysis. General data analysis was completed with Microsoft Excel (Office 365) and GraphPad 8 (v8.0.2). Inforna was used to identify druggable sites within pre-miR-155. The Inforna database can be accessed via the following URL https://rnainforna.com. Access is freely granted for academic users after completion of a software license agreement (https://disney.scripps.ufl.edu/wp-content/uploads/2020/05/software-licensing-agreement-1.pdf). Inforna and R-BIND (https://rbind.chem.duke.edu/) were used to identify known RNA-binding small molecules.<br>Tanimoto coefficients and physicochemcial properties of small molecules were calculated by using Instant JChem v.19.8.0<br>LOGOS analysis was performed by using JMP, v.13.2.1 and DiffLogo, version 3.16, installed on RStudio (v.1.2.5042) with R 3.6.3.<br>Microscale thermophoresis binding assays were analyzed by MO.AffinityAnalysis v1.115<br>Western blots were analyzed with Image J v.1.8.0_112<br>For RNA-seq data analysis, STAR (v2.5.2a) was used for alignment; featureCounts (v2.0.0) was used to extract read counts from bam files; and DESeq2 (v3.13) was used for differential gene expression analysis.<br>Proteomic data analysis was performed by using MaxQuant (v2.3.0.0).

For manuscripts utilizing custom algorithms or software that are central to the research but not yet described in published literature, software must be made available to editors and reviewers. We strongly encourage code deposition in a community repository (e.g. GitHub). See the Nature Portfolio guidelines for submitting code & software for further information.

Data

Policy information about availability of data

All manuscripts must include a data availability statement. This statement should provide the following information, where applicable:

- Accession codes, unique identifiers, or web links for publicly available datasets
- A description of any restrictions on data availability
- For clinical datasets or third party data, please ensure that the statement adheres to our policy

All data and materials are available from the corresponding author upon reasonable request. The data for global proteomics and RNA-seq studies are available on Mendeley Data (DOI:10.17632/xgr83xy8pm.1). The sequencing data for plasmids used in this study are available on Mendeley Data (10.17632/9zgvv67j7s.1). The raw sequencing data are deposited in the Sequence Read Archive (SRA) with BioProject ID: PRJNA914317. The R-Bind database is publicly accessible via the following URL https://rbind.chem.duke.edu. The Inforna database can be accessed via the following URL https://rnainforna.com. Access is freely granted for academic users after completion of a software license agreement (https://disney.scripps.ufl.edu/wp-content/uploads/2020/05/software-licensing-agreement-1.pdf).

Human research participants

Policy information about studies involving human research participants and Sex and Gender in Research.

Reporting on sex and gender	N/A
Population characteristics	N/A
Recruitment	N/A
Ethics oversight	N/A

Note that full information on the approval of the study protocol must also be provided in the manuscript.

Field-specific reporting

Please select the one below that is the best fit for your research. If you are not sure, read the appropriate sections before making your selection.

☒ Life sciences ☐ Behavioural & social sciences ☐ Ecological, evolutionary & environmental sciences

For a reference copy of the document with all sections, see nature.com/documents/nr-reporting-summary-flat.pdf

Life sciences study design

All studies must disclose on these points even when the disclosure is negative.

Sample size	The sample size was chosen to enable measuring statistical significance of differences observed between treated and untreated groups or compound-treated and vehicle-treated groups. No statistical methods were used to predetermine the sample sizes. However, sample sizes were in agreement with many other literature reports that measure changes in miRNA levels, protein levels, etc (see PMID 30116813, 28288108, 25849773, 20354188, 30620551, 35355011, 26030728, 35561226, 25104390, 34826236, and 35641483 as examples).
Data exclusions	There were no data exclusions except for in the case of RT-qPCR when OD260/OD280 < 1.8. These samples were excluded from analysis.
Replication	All experiments were confirmed with multiple replicates as indicated in each figure legend. Plots show all data points; representative Western

Replication	blots, binding curves, histology analyses, and FISH images were provided.
Randomization	All mice were randomly assigned to either the control group or the treatment group. For cellular and in vitro experiments, plate wells were randomly assigned to treatment groups.
Blinding	Investigators were blinded when i) grouping the mice; ii) dosing the mice; iii) harvesting the organs; iv) counting the number of nodules on lung samples; and iv) preparation of histology samples. Investigations were not blinded when i) extracting RNA samples for RT-qPCR and ii) quantifying histology images. All in vitro and cellular samples not blinded as they yield quantitative results that are not subject to investigator bias. It should be noted that these types of experiments are typically unblinded from the literature (see PMID 36725932, 36859550 as examples).

Reporting for specific materials, systems and methods

We require information from authors about some types of materials, experimental systems and methods used in many studies. Here, indicate whether each material, system or method listed is relevant to your study. If you are not sure if a list item applies to your research, read the appropriate section before selecting a response.

Materials & experimental systems

n/a	Involved in the study
☐	☒ Antibodies
☐	☒ Eukaryotic cell lines
☒	☐ Palaeontology and archaeology
☐	☒ Animals and other organisms
☒	☐ Clinical data
☒	☐ Dual use research of concern

Methods

n/a	Involved in the study
☒	☐ ChIP-seq
☐	☒ Flow cytometry
☒	☐ MRI-based neuroimaging

Antibodies

Antibodies used

β-actin primary antibody (Cell Signaling Technology, 3700S)
RNase L primary antibody (Cell Signaling Technology, D4B4J)
anti-rabbit IgG horseradish-peroxidase secondary antibody conjugate (Cell Signaling Technology, 7074S)
anti-mouse IgG horseradish-peroxidase secondary antibody conjugate (Cell Signaling Technology, 7076S)
SOCS1 primary antibody (Cell Signaling Technology, 3950S)
VHL primary antibody (Cell Signaling Technology, 68547S)
c-JUN primary antibody (Cell Signaling Technology, 9165S)
c-Myc primary antibody (Cell Signaling Technology, 5605S)
BRD4 primary antibody (Cell Signaling Technology, 13440S)
GAPDH primary antibody (Cell Signaling Technology, 51332S)

Validation

All antibodies are validated by the manufacturer as below:
β-actin primary antibody: https://www.cellsignal.com/products/primary-antibodies/b-actin-8h10d10-mouse-mab/3700?_=1678131448181&Ntt=3700S&tahead=true

RNase L primary antibody: https://www.cellsignal.com/products/primary-antibodies/rnase-l-d4b4j-rabbit-mab/27281?site-search-type=Products&N=4294956287&Ntt=d4b4j&fromPage=plp

anti-rabbit IgG horseradish-peroxidase secondary antibody conjugate: https://www.cellsignal.com/products/secondary-antibodies/anti-rabbit-igg-hrp-linked-antibody/7074?site-search-type=Products&N=4294956287&Ntt=7074s&fromPage=plp&_requestid=767849

anti-mouse IgG horseradish-peroxidase secondary antibody conjugate: https://www.cellsignal.com/products/secondary-antibodies/anti-mouse-igg-hrp-linked-antibody/7076?site-search-type=Products&N=4294956287&Ntt=7076s&fromPage=plp&_requestid=768047

SOCS1 primary antibody: https://www.cellsignal.com/products/primary-antibodies/socs1-a156-antibody/3950?site-search-type=Products&N=4294956287&Ntt=3950s&fromPage=plp&_requestid=768092

VHL primary antibody: https://www.cellsignal.com/products/primary-antibodies/vhl-antibody/68547

c-JUN primary antibody: https://www.cellsignal.com/products/primary-antibodies/c-jun-60a8-rabbit-mab/9165

c-Myc primary antibody: https://www.cellsignal.com/products/primary-antibodies/c-myc-d84c12-rabbit-mab/5605

BRD4 primary antibody: https://www.cellsignal.com/products/primary-antibodies/brd4-e2a7x-rabbit-mab/13440

GAPDH primary antibody: https://www.cellsignal.com/products/antibody-conjugates/gapdh-d4c6r-mouse-mab-hrp-conjugate/51332

Eukaryotic cell lines

Policy information about cell lines and Sex and Gender in Research

Cell line source(s)	MDA-MB-231 cells (HTB-26, ATCC), HEK293T (CRL-3216, ATCC), HUVEC-Umbilical Vein Endo cells(CC-2517, Lanza), MCF-10A cells (CRL-10317, ATCC), CFPAC-1 (CRL-1918,ATCC), HeLa (CCL-2, ATCC), HEK293T (CRL-3216, ATCC), Namalwa (CRL-1432, ATCC), Raji (CCL-86, ATCC), HL-60 (CCL-240, ATCC), and MIA PaCa-2 (CRL-1420, ATCC). MDA-MB-luc cells were a gift from Prof. Derek Duckett (Moffitt Cancer Center) as reported in Rosenberg et al. Therapeutic Targeting of Casein Kinase 1δ in Breast Cancer. Sci Transl Med. 2015 Dec 16; 7(318): 318ra202.
Authentication	Commercially available cell lines were authenticated by the manufacturer, and a specification sheet was provided by the manufacturer confirming identity. MDA-MB-luc were as reported in Rosenberg et al. Therapeutic Targeting of Casein Kinase 1δ in Breast Cancer. Sci Transl Med. 2015 Dec 16; 7(318): 318ra202. All cell lines were frequently checked by their morphological features to confirm their authentication.
Mycoplasma contamination	All cell lines were tested to be mycoplasma-negative by using PCR Mycoplasma Test Kit I/C (PromoKine, PK-CA91-1024)
Commonly misidentified lines (See ICLAC register)	No commonly misidentified cell lines are used in this study.

Animals and other research organisms

Policy information about studies involving animals; ARRIVE guidelines recommended for reporting animal research, and Sex and Gender in Research

Laboratory animals	Female NOD/SCID mice and C57Bl/6 (5-7 weeks, purchased from Jackson Laboratory) were used in this study.
Wild animals	The study did not involve wild animals.
Reporting on sex	No sex-based design or analysis was performed for this study.
Field-collected samples	The study did not involve samples collected from the field.
Ethics oversight	Scripps Florida Institutional Animal Care and Use Committee approved the protocol.

Note that full information on the approval of the study protocol must also be provided in the manuscript.

Flow Cytometry

Plots

Confirm that:

- [x] The axis labels state the marker and fluorochrome used (e.g. CD4-FITC).
- [x] The axis scales are clearly visible. Include numbers along axes only for bottom left plot of group (a 'group' is an analysis of identical markers).
- [x] All plots are contour plots with outliers or pseudocolor plots.
- [x] A numerical value for number of cells or percentage (with statistics) is provided.

Methodology

Sample preparation	Namalwa (0.4 million cells/mL, 1 mL), HL-60 (0.5 million cells/mL, 1 mL), and Raji (0.7 million cells/mL, 1 mL) were treated with vehicle, c-Myc-RiboTAC, or c-Myc-Ctr for 48 h. Apoptosis was measured by CellEvent Caspase-3/7 Green ReadyProbes Reagent (ThermoFisher, C10423) and DAPI (0.1 µg/ml, Molecular Probe) following the manufacturer's protocol. Dye was directly added to cells in flow cytometry analysis buffer (1x DPBS, 10% (v/v) FBS), and incubated for 20 min at room temperature. For Propidium Iodide cell cycle analyses, cells were harvested and washed in PBS. Cells were fixed for 30 min at 4oC in cold 70% ethanol. Cold 70% ethanol was added drop wise to the pellet while vortexing. Fixed cells were washed twice in PBS. Cells were spun at 850 g and the supernatant discard. Cells were treated the with ribonuclease by adding 50 µl of a 100 µg/ml (1:100 from 10mg/ml, Sigma R-6148) stock of RNase. 200 µl PI was then added prior to analysis (50 µg/ml-from 1:20 of 1mg/ml stock solution, Millipore Sigma, P4170).
Instrument	BD LSRII (BD Biosciences) flow cytometer
Software	BD FACSDiva for sample acquisition and FlowJo for sample analysis

Cell population abundance

The purities of the samples were not determined. As customary in FACS analysis, debris was excluded by evaluating forward scatter vs. side scatter (FSC vs. SSC).

Gating strategy

For Caspase-3/7 Green analyses, cells are gated via FSC vs SSC, singlets are further gated for analyses.
For Propidium Iodide cell cycle analyses, the following gates were used prior to PI quantification.
SSC-A vs FCS-A
SSC-W vs SSC-H
FCS-W vs FCS-H
610/20-W vs 610/20-A= PI singlets

☒ Tick this box to confirm that a figure exemplifying the gating strategy is provided in the Supplementary Information.
